# A Population-Based Human In Vitro Approach to Quantify Inter-Individual Variability in Responses to Chemical Mixtures

**DOI:** 10.3390/toxics10080441

**Published:** 2022-08-01

**Authors:** Lucie C. Ford, Suji Jang, Zunwei Chen, Yi-Hui Zhou, Paul J. Gallins, Fred A. Wright, Weihsueh A. Chiu, Ivan Rusyn

**Affiliations:** 1 Interdisciplinary Faculty of Toxicology, College of Veterinary Medicine and Biomedical Sciences, Texas A&M University, College Station, TX 77843, USA; lford@cvm.tamu.edu (L.C.F.); sjang@cvm.tamu.edu (S.J.); zunwei_chen@hsph.harvard.edu (Z.C.); wchiu@tamu.edu (W.A.C.); 2 Department of Veterinary Physiology and Pharmacology, College of Veterinary Medicine and Biomedical Sciences, Texas A&M University, College Station, TX 77843, USA; 3 Departments of Biological Sciences and Statistics, North Carolina State University, Raleigh, NC 27695, USA; yihui_zhou@ncsu.edu (Y.-H.Z.); fred_wright@ncsu.edu (F.A.W.); 4 Bioinformatics Research Center, North Carolina State University, Raleigh, NC 27695, USA; pjgalli2@ncsu.edu

**Keywords:** population-wide, inter-individual variability, toxicodynamics, chemical mixtures, defined mixtures, human health risk assessment, uncertainty factors, genome-wide association study

## Abstract

Human cell-based population-wide in vitro models have been proposed as a strategy to derive chemical-specific estimates of inter-individual variability; however, the utility of this approach has not yet been tested for cumulative exposures in mixtures. This study aimed to test defined mixtures and their individual components and determine whether adverse effects of the mixtures were likely to be more variable in a population than those of the individual chemicals. The in vitro model comprised 146 human lymphoblastoid cell lines from four diverse subpopulations of European and African descent. Cells were exposed, in concentration–response, to 42 chemicals from diverse classes of environmental pollutants; in addition, eight defined mixtures were prepared from these chemicals using several exposure- or hazard-based scenarios. Points of departure for cytotoxicity were derived using Bayesian concentration–response modeling and population variability was quantified in the form of a toxicodynamic variability factor (TDVF). We found that 28 chemicals and all mixtures exhibited concentration–response cytotoxicity, enabling calculation of the TDVF. The median TDVF across test substances, for both individual chemicals or defined mixtures, ranged from a default assumption (10^1/2^) of toxicodynamic variability in human population to >10. The data also provide a proof of principle for single-variant genome-wide association mapping for toxicity of the chemicals and mixtures, although replication would be necessary due to statistical power limitations with the current sample size. This study demonstrates the feasibility of using a set of human lymphoblastoid cell lines as an in vitro model to quantify the extent of inter-individual variability in hazardous properties of both individual chemicals and mixtures. The data show that population variability of the mixtures is unlikely to exceed that of the most variable component, and that similarity in genome-wide associations among components may be used to accrue additional evidence for grouping of constituents in a mixture for cumulative assessments.

## 1. Introduction

Humans are exposed to a wide variety of chemicals from both dietary and non-dietary sources; therefore, developing approaches for the risk assessment of combined exposures to multiple agents is the pressing challenge in regulatory science [1]. Evaluation of mixtures and complex substances, which are the primary types of combined exposures, is hindered by the challenges of determining their chemical composition [2,3]. Mixtures are categorized into intentional and unintentional types. The former are mixtures with largely known chemical constituents (e.g., paint, food additives, pesticide formulations), while the latter include discharge, coincidental, and environmental mixtures [4]. Discharge mixtures are defined as substances released from a single installation during production, transportation, or waste disposal processes. Coincidental mixtures are those that originate from multiple sources, thus further increasing their complexity. Environmental mixtures are defined as a combination of chemical constituents from multiple sources that occur in various exposure settings (e.g., air pollution) [3].

Due to the chemical complexity of mixtures, there are no harmonized methodologies for their risk assessment; recent efforts by a number of regulatory agencies worldwide aim to devise a standardized approach [3,5,6,7]. Two methods are commonly used for assessing mixtures, the whole mixture and component-based approaches. The whole mixture approach is challenging as it assumes invariable composition of a substance which is not a realistic scenario [8,9,10]; it also requires testing of the whole substance which presents unique analytical challenges if the components are not water soluble [11]. The component-based approach uses exposure and toxicity data from either individual chemicals or groups of chemicals (i.e., component assessment groups) to conduct the assessment. This approach is predicated on the availability of information on the “marker” chemical constituent(s) in a mixture, which is both an analytical (i.e., availability of standards and instrumentation to quantify constituents) and practical (i.e., temporal changes and batch-to-batch variability in composition) challenge [7]. Solutions to address these challenges have been proposed in the areas of both analytical chemistry and in vitro toxicology, still, their implementation has yet to be widely adopted [12].

One of the particularly challenging components of the risk characterization step in assessments of both individual chemicals and mixtures is the estimation of the extent of population variability in toxicity [13]. Considerations of intra-species variability are typically addressed through the use of a default assumption in calculations of the margin of exposure [14]. As an alternative to using the defaults [15], a number of experimental approaches have been recently proposed to derive chemical-specific estimates of human variability [16]; however, few studies have applied these novel experimental models to mixtures. Due to the need to test either numerous components of a mixture or many “whole” mixtures, in vitro approaches using human cells would be most sensible in terms of necessary throughput. Still, only limited options exist for such studies, human lymphoblastoid cell lines [17] and induced pluripotent stem cells (iPSC) [18]. Large sets of chemicals have been tested in lymphoblasts and iPSC-derived cardiomyocytes, thus providing experimental data for chemical-specific assessments [19,20,21]. The examples for mixtures are fewer, with one study evaluating human population variability in toxicity of whole mixtures of pesticides using lymphoblast cells [22].

Opportunities exist to apply population-based human in vitro models, such as lymphoblasts, to whole- and component-based mixtures assessments by testing the validity of dose and variability assumptions using defined (i.e., with known components) mixtures. The aim of this study was to test defined mixtures and their individual components and determine whether adverse effects of the mixtures were likely to be more variable in a population than those of the individual chemicals. Therefore, we hypothesized that a population of lymphoblasts can be used to screen both individual chemicals and defined mixtures to determine the extent of variability of cytotoxic effects across individuals. A panel of 146 lymphoblastoid cell lines from four subpopulations were used to screen 42 individual chemicals and eight defined mixtures in concentration–response. Cytotoxicity was evaluated as the endpoint of interest, from which the points of departure were derived to quantify chemical- and mixture-specific estimates of variability, and to identify potential molecular drivers of variability.

## 2. Experimental Section

### 2.1. Chemicals and Biologicals

RPMI 1640 maintenance media (Cat#11875119) and penicillin-streptomycin (Cat#10378016) were obtained from LifeTechnologies (Grand Island, NY, USA). Premium grade Fetal Bovine Serum (FBS) was obtained from VWR (Cat#97068-085, Radnor, PA, USA). Tissue-culture treated T75 cell culture flasks were obtained from Fisher Scientific (Cat#156499, Waltham, MA, USA). Tissue-culture treated 384-well black/clear bottom plates were obtained from Corning (Cat#3764, Kennebunk, ME, USA) and Greiner Bio-One (Cat#781091, Kremsmünster, Austria). Tissue culture grade dimethyl sulfoxide (DMSO, CAS#67-68-5, Cat#sc-358801) was obtained from Santa Cruz Biotechnology (Dallas, TX, USA). Trypan Blue 0.4% solution was obtained from MilliporeSigma (Cat#T8154-100 ML, Burlington, MA, USA). The CellTiter-Glo assay kit was obtained from Promega (Madison, WI, USA). Tetra-octyl ammonium bromide (TAB, CAS#14866-33-2, Cat# D2438) was obtained from SigmaAldrich (St. Louis, MO, USA). Test chemicals (*n* = 42, Table S1) were from various sources as indicated therein.

### 2.2. Cell Lines

A set of immortalized lymphoblast cell lines was from the Coriell Institute (Camden, NJ, USA; *n* = 146, Table S2). The cells are human B-lymphocytes that were originally collected by the 1000 Genomes Project [23]. The cell lines used herein were from four human subpopulations that represent geographical diversity in their ancestral origins and were also tested in previous studies of environmental mixtures [22]. The populations included were Utah residents with European ancestry (CEU), Tuscans in Italy (TSI), British from England and Scotland (GBR), and Yoruban from Ibadan, Nigeria (YRI). The lymphoblast cell lines were selected to have equal male to female representation.

### 2.3. Cell Culture

For technical reasons, the cell lines were divided into seven testing batches, with equal representation from each subpopulation and sex in each batch. The cells were cultured in T75 flasks at 37 °C with 5% CO_2_ in RPMI 1640 media supplemented with 15% FBS and 1% penicillin-streptomycin. Media was changed every other day. Cell count and viability were assessed during media changes using the Cellometer™ Auto T4 Plus (Nexelom Bioscience, Lawrence, MA, USA). The cells were expanded up to a minimum concentration of 10^6^ cells/mL and viability of ≥85% prior to plating and chemical exposure. Before transfer to 384-well plates for chemical treatments, the cells were centrifuged and re-suspended in the fresh media. Cell density and viability were evaluated and the cells were plated into tissue culture-treated 384-well black/clear bottom plates at a density of 5000 cells/well in 12.5 μL of media. To evaluate experimental reproducibility, each cell line was seeded in three identical 384-well plates and each chemical and mixture were tested on each plate (see Figure S1 for the plate design). After chemical addition, the cells were incubated for 24 h at 37 °C with 5% CO_2_.

### 2.4. Chemicals and Mixtures

The individual chemicals for testing were selected as previously detailed [24] based on the following criteria: (i) chemicals listed on the Agency for Toxic Substances and Disease Registry priority list; (ii) chemicals frequently detected at the U.S. National Priority list sites (also known as Superfund sites); (iii) representation of various chemical classes [pesticides (*n* = 20), high production volume chemicals (HPV) (*n* = 8), heavy metals (*n* = 7), polycyclic aromatic hydrocarbons (PAHs) (*n* = 5), and phthalates (*n* = 2)]; (iv) established human “safe exposure” levels; (v) reverse toxicokinetic and exposure data available on U.S. Environmental Protection Agency (U.S. EPA) dashboard; and (vi) available data from other in vitro studies through ToxCast™/Tox21. Each chemical was first dissolved in 100% DMSO at 20 mM, then, five 10× serial dilutions were prepared with 100% DMSO. Then, each sample was further diluted 100-fold in corresponding cell culture media to yield 2× working solutions in 1% DMSO. Finally, 12.5 μL of 2× solution with chemical/mixture was added to each well with cells using the automated liquid handler in the FLIPR tetra (Molecular Devices, San Jose, CA, USA) for the final concentrations of 100, 10, 1, 0.1, or 0.01 μM, and 0.5% DMSO in each well. The concentration of DMSO in assay wells at 0.5% (*v*/*v*) was consistent with previous reports and by itself had no effect on lymphoblast viability [25].

The mixtures were assembled based on various assumptions to model environmental scenarios as previously detailed [26]. In summary, each chemical was present in every mixture in amounts based on the following assumptions: (i) points of departure (POD) from in vivo studies in experimental animals used for determining regulatory oral non-cancer reference doses (RfD); (ii) the RfD derived from the experimental animal data and converted to oral equivalent dose in humans [27]; (iii) human exposure estimates from ExpoCast [28]; and (iv) in vitro toxicity data (active concentration 50%, AC_50_) from ToxCast/Tox21 [29]. In each assumption, we created two mixtures to represent the median (termed “low”) or upper 95th percentile (termed “high”) to account for the human toxicokinetic variability. The concentration of each chemical in the eight tested mixtures is provided in Table S3. The chemicals were first dissolved in 100% cell culture-grade DMSO at a concentration of 20 mM each and then aliquoted at different proportions to create stocks for each mixture. All mixtures were then tested using five 10× serial dilutions to generate concentration–response data. For testing, stocks were further diluted 200× with cell culture media as detailed above for the chemicals.

### 2.5. Cell Viability Screening

After the 24 h incubation with the individual chemicals or mixtures, intracellular ATP concentration was measured as a marker for cell viability. Microplates were equilibrated at room temperature for 30 min and the CellTiter-Glo reagent (25 μL) was added in equal volumes to each well. The plates were first mixed for two minutes on a plate shaker to induce cell lysis and then incubated at room temperature for 10 min in the dark. The luminescence signal was read using a FLIPR Tetra and Screenworks 4.0 (Molecular Devices).

### 2.6. Assessment of Experimental Reproducibility

Experimental reproducibility was evaluated both within and among plates. Within each plate, two chemicals were tested as intra-plate replicates: 2,4,5-trichlorophenol and mercuric chloride. First, raw luminescence counts in each experimental well were normalized to the average of the wells containing vehicle (0.5% DMSO). Then, normalized values at each tested concentration were used to assess Pearson correlation coefficient and associated *p*-values were calculated for both chemicals (Table S4). Inter-plate reproducibility was evaluated using data from each cell line that were screened on three individual plates. Normalized values for each well from two out of three plates (randomly selected) were used to calculate Pearson correlation coefficient and the corresponding *p*-values (Table S5). Both tests were performed for each cell line.

### 2.7. Derivation of Chemical and Mixture-Specific Concentration–Response Profiles

After normalization and quality control (coefficient of variability <20% for vehicle-treated wells and 100% cytotoxicity in wells exposed to 100 μM tetra-octyl ammonium bromide), a concentration–response model was fit for each chemical and mixture using hierarchical Bayesian random effects Hill models as described in [30]. For each chemical or mixture and individual cell line, it was assumed that the concentration–response data follow a “downward” Hill model [30,31] where response *y* as a function of concentration *x* follows the equation:

$$y=y_{0}\left(1-\frac{{\left(\frac{x}{x_{0}}\right)}^{n}}{1+{\left(\frac{x}{x_{0}}\right)}^{n}}\right)+\epsilon$$

The variable $y_{0}$ is the baseline value, $x_{0}$ is the concentration at a 50% decrease from the baseline response (EC_50_), n is the Hill coefficient, and ϵ is the residual error. Fitting of $y_{0}$ was needed to address “drifting” baseline values even after normalization via negative controls. To prevent anomalous fits with very shallow concentration–response curves, the Hill coefficient was restricted to be ≥1, as noted previously [26]. All parameters, after natural log-transformation, were assumed to have normal random effects so that individuals in the population were distributed normally given population mean and standard deviation hyperparameters. Prior distributions for hyperparameters were normal for population means and half-normal for population standard deviations. The error ϵ between estimated and actual values was assumed to follow a scaled Student’s *t* distribution with scale parameter σ where ϵ/σ has a standard Student’s *t* distribution, with ν=5 degrees of freedom, to be robust for outliers [30].

We used the Markov chain Monte Carlo (MCMC) algorithm to sample the posterior distribution using the R Stan package [32]. Four chains were set for each chemical or mixture and each chain initially contained 4000 iterations. The first half iterations for each chain were used as warm-up data and discarded, with the rest used for inference. In order to assess convergence indicating the adequacy of sampling, the potential scale reduction factor $\hat{R}$ was used to compare between- and within-chain variability, with values of $\hat{R}$ ≤ 1.2 considered adequately converged [33]. Chain length was doubled until convergence, with no dataset requiring more than 16,000 iterations.

### 2.8. Derivation of Chemical and Mixture-Specific Point of Departure (POD) and Toxicodynamic Variability Factor (TDVF_05_)

For the point of departure (POD), the effective concentration at which a 10% change in cytotoxicity in comparison to vehicle (EC_10_) was selected as the benchmark response based on the US EPA guidance for dose–response modeling and determination of the point-of-departure values [34], and consistent with previously published work using in vitro model and cytotoxicity endpoints [19,30,31]. The EC_10_ was calculated for each individual *i* using the equation $EC_{10,i}=x_{0,i}{\left(0.1/0.9\right)}^{1/n_{i},}$, where $x_{0,i}$ and $n_{i}$ are the individual-specific values (MCMC samples) for the EC_50_ and the Hill parameter, respectively. A chemical or mixture was considered “inactive” if the population median EC_10_ > 3× the maximum tested concentration (100 μM for individual chemicals, see Table S3 for mixtures), and further corroborated by visual inspection of the concentration–response data.

For the analysis of inter-individual variability in toxicodynamic response, the toxicodynamic variability factor at 5% (TDVF_05_) is defined as the ratio of the POD for the median individual to the POD for the most sensitive 5th percentile individual: TDVF_05_ = EC_10,median_/EC_10,5th percentile_. Given the EC_10_ values for all individuals in the population, the median and 5th percentile quantiles across individuals were calculated, with their ratio set equal to the TDVF_05_. This procedure was repeated for each MCMC sample, so that the result is a distribution representing the uncertainty in the TDVF_05_ for each chemical or mixture. The default uncertainty factor for toxicodynamic variability is generally considered to be 10^1/2^, TDVF = 3.16 [35], which was used as a comparison benchmark.

### 2.9. Genome-Wide Association Mapping

Genome-wide association mapping of the EC_10_ values could be performed because genotypes for these cell lines are available from the 1000 Genomes resource (www.internationalgenome.org/data/ (accessed on 1 June 2022)). While the statistical power for mapping of toxicity as a complex trait is low for the current dataset, we conducted this analysis as a proof of principle for exploration, and the results were recorded for replication or inclusion in future meta-analyses. Genotypes were recoded as the number of copies of the minor allele (0, 1, 2). Due to the limited sample size, ~1.3 million single nucleotide polymorphisms (SNPs) with minor allele frequency (MAF) ≥0.05 were used for the mapping analysis. A standard linear regression model for additive allele effects was employed, using sex and three genotype-based principal components as covariates. Batch effects were examined by genotype principal components, with no discernable effects (results not shown). SNP associations were evaluated using the t-statistic *p*-value for the SNP coefficient in the linear model. Quantile–quantile plots of association showed no evidence of significance inflation (Figure S2). Manhattan plots and regional visualizations were produced using the LocusZoom software (locuszoom.org) and by reference to the UCSC Genome Browser (https://genome.ucsc.edu/ (accessed on 1 June 2022)). We used *p* < 10^−5^ as indicative of suggestive significance, and *p* < 5 × 10^−8^ as genome-wide significant [36].

Although the use of genotype principal components as covariates in linear models is well established [37], for completeness we also used an alternate mixed-linear-model association (MLMA) method [38]. First, we estimated the genetic relatedness between pairs of individuals using the SNPs and generated a genetic relationship matrix. We analyzed the individuals from the populations of European descent (*n* = 106) and of African descent (*n* = 40) separately, adjusting for relatedness and including sex and principal components as covariates. Then we ran a meta-analysis by weighting z-scores according to the relative sample sizes from each ancestry group to generate final SNP *p*-values (Figure S3 and Table S6).

## 3. Results

### 3.1. Population Variability Screening of Chemicals and Mixtures in Human Lymphoblasts

**Figure 1:**
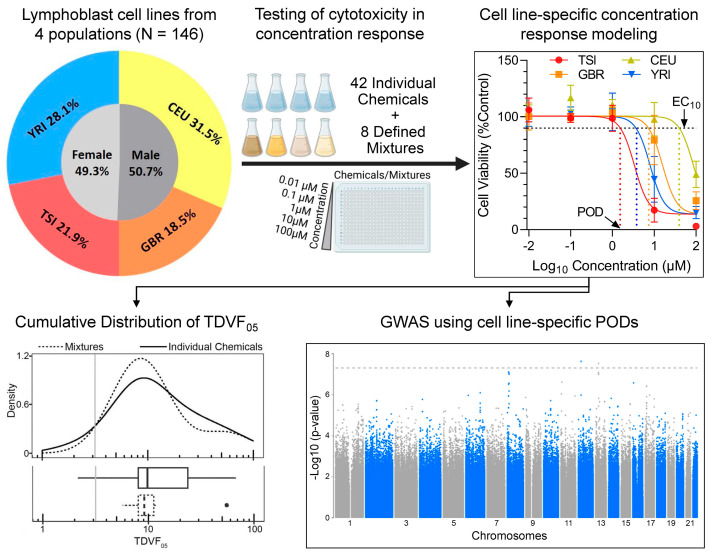
Experimental design for population-based human in vitro toxicity assessments of environmental chemicals and mixtures. A sample of 146 human lymphoblast cell lines from four subpopulations with equal male to female representation were used in this study. All cell lines were exposed to 42 chemicals and eight environmental mixtures in concentration–response to derive points of departure (PODs) which were then used to calculate the TDVF_05_ and conduct GWAS analyses.

This study tested inter-individual variability in the effects of 42 chemicals and eight defined mixtures using a population-based human in vitro model. We used cells from a total of 146 donors representing four subpopulations, three from European descent and one African descent, balanced for sex ratio. Figure 1 shows the overall study design and data analysis pipelines. Both chemicals and mixtures were tested in concentration–response from which EC_10_ values were derived as the PODs, these were used to calculate the TDVF_05_ and conduct genome-wide association analysis.

**Figure 2:**
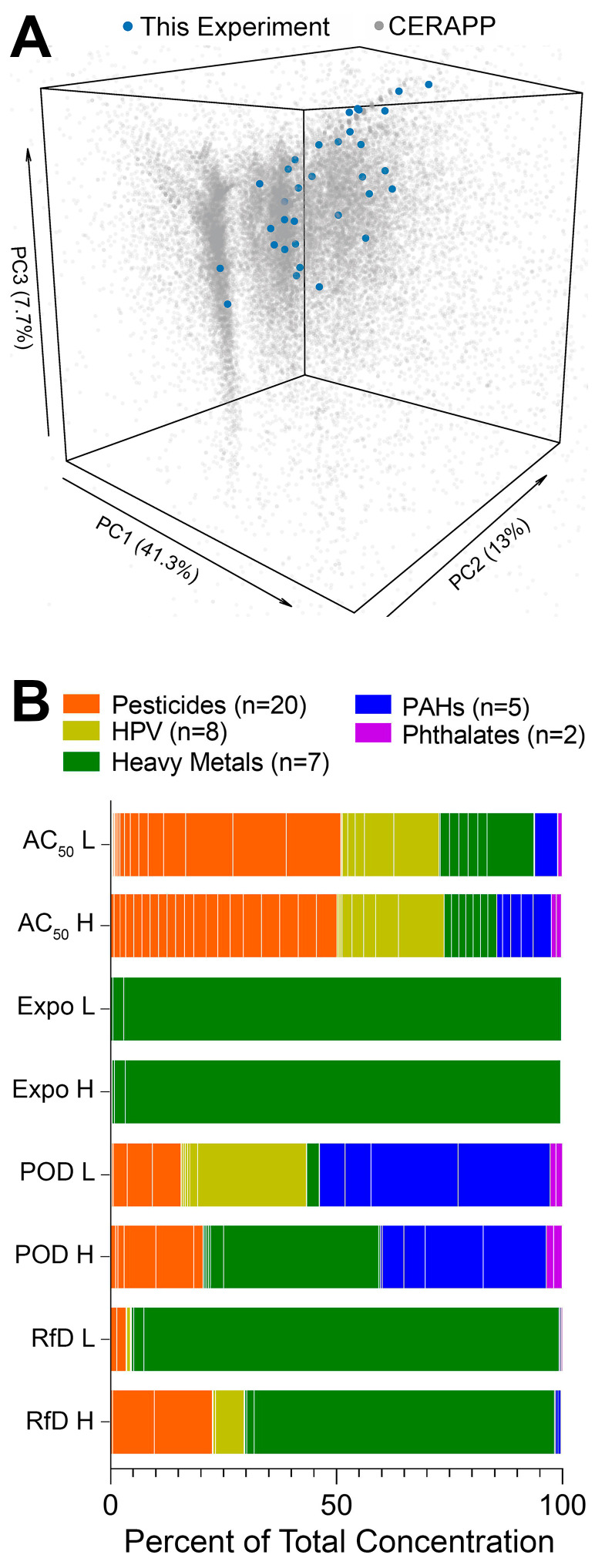
Diversity in the chemical structure of selected chemicals and defined mixture composition. (**A**) Principal component analysis (PCA) plot demonstrating the structural diversity of the chemicals tested in this study as compared to the representative environmental compound set included in the Collaborative Estrogen Receptor Activity Prediction Project (CERAPP, *n* = 32,464) [39]. We used 48 chemical descriptors (after omitting NA or constant descriptors) calculated using the open-source Chemistry Development Kit (implemented in the R package *rcdk*) [40] to illustrate the spatial distribution of both datasets. Principal component analysis was conducted using *prcomp* from the R *stats* package, and visualization was conducted with the R *plot3D* package. The axes were truncated at the 95% confidence interval of each principal component. (**B**) Defined mixtures boxplots of the percent of total concentration for each individual chemical across eight defined mixtures. The boxplots are arranged by individual chemicals and chemical classes [orange = pesticides (*n* = 20), yellow = HPV (*n* = 8), green = heavy metals (*n* = 7), blue = PAHs (*n* = 5), purple = phthalates (*n* = 2)].

Compounds selected for this study spanned a number of chemical classes (e.g., pesticides, high production volume chemicals, heavy metals, polycyclic aromatic hydrocarbons, and phthalates) to be largely representative of the structural diversity of the chemicals with documented use and exposure potential across various US EPA chemical product categories [39]. Specifically, the principal components analysis plot (Figure 2A) shows that the chemicals tested herein are distributed across the compendium of chemicals selected by the Collaborative Estrogen Receptor Activity Prediction Project (*n* = 32,464 chemicals) [39]. Due to the difference in assumptions for defined mixture preparation (see Section 2. Experimental), concentrations of the individual chemicals in each mixture varied widely (Figure 2B). For example, the ToxCast database mixtures were most balanced in terms of relative composition, but exposure-based mixtures were dominated by the presence of zinc chloride. Overall, we aimed to represent very diverse mixture scenarios and the variability in composition was by design.

**Figure 3:**
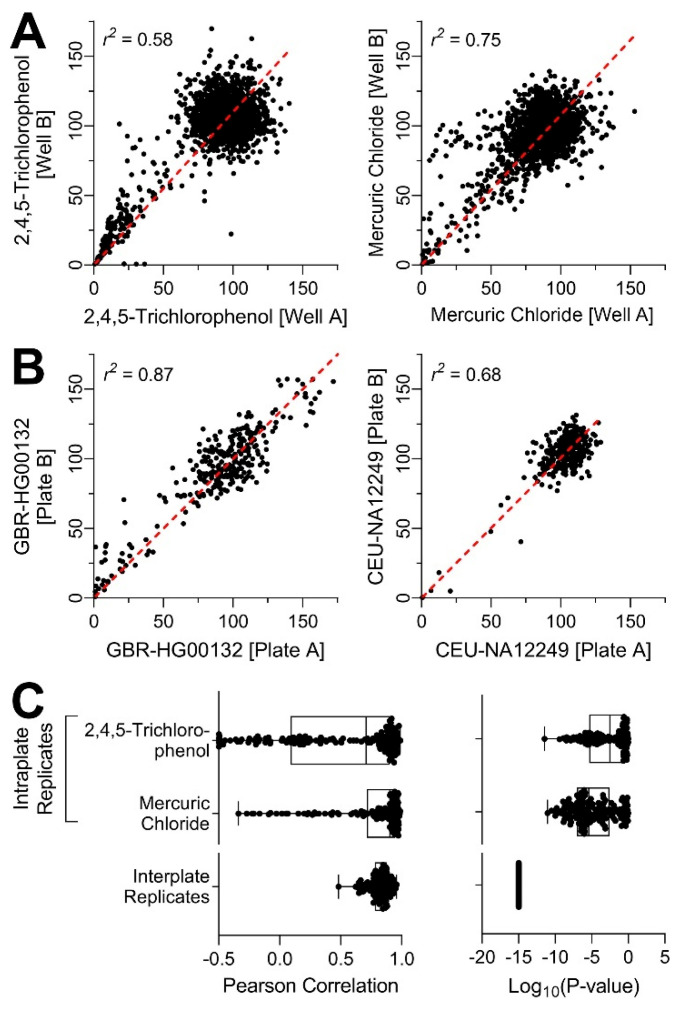
Technical reproducibility of in vitro cell viability assays across all donors. (**A**) Intra-plate reproducibility correlation plots of the two intra-plate replicate substances: 2,4,5-trichlorophenol and mercuric chloride, correlations include the entire concentration–response profile for all cell lines tested. (**B**) Inter-plate reproducibility correlation plots. Of the three plate replicates available, two were randomly selected for correlation analysis. Representative plots are shown for the most susceptible cell line GBR-HG00132 (i.e., highest variability across all chemical-specific PODs, left) and the most resistant cell line CEU-NA12249 (i.e., lowest variability across all PODs, right). (**C**) Distribution of Pearson correlation coefficients and the corresponding *p*-values for intra- and inter-plate replicates across all cell lines.

In order to ensure that this complex (in terms of the number of cell lines tested and variables interrogated) experiment was technically sound, we first evaluated intra- and inter-plate concordance (Figure 3). Experimental reproducibility was assessed by including chemicals that were screened as intra-plate replicates, as well as testing the chemicals and mixtures on a number of independent plates for each donor. Both chemicals that were screened as intra-plate replicates, mercuric chloride and 2,4,5-trichlorophenol were without effect across many donors; however, in the lines that had an effect, intra-plate replicates were significantly correlated (Figure 3A,C). The inter-plate reproducibility was even higher (Figure 3B,C). While correlations were greater in the individuals that were susceptible to the effects of tested compounds (e.g., GBR-HG00132) because of a wider spread of responses to individual treatments, even in resistant individuals (e.g., CEU-NA12249) the correlation coefficients were highly significant. Overall, experimental reproducibility was demonstrated by both the intra-plate replicates and the inter-plate replicates thus, increasing confidence in the follow-up analyses.

### 3.2. Concentration–Response Analyses of the Effects of Chemicals and Mixtures

**Figure 4:**
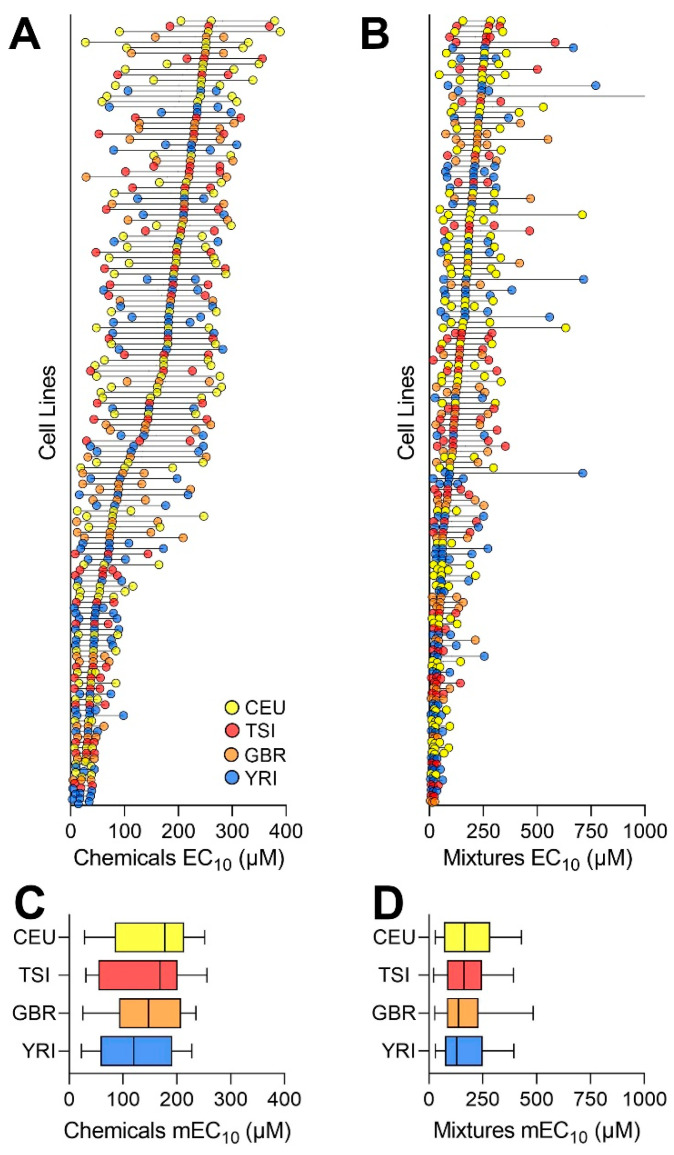
Cell-line specific distribution of chemical and mixture PODs. Distribution of the PODs (EC_10_ values) for all chemicals (**A**) and mixtures (**B**) arranged by each cell line’s median value. Colors represent the subpopulations (see inset). Each line is the inter-quartile range of the PODs, with the middle dot representing the median value. Overall distribution of median POD values for chemicals (**C**) and mixtures (**D**) across each subpopulation. Box-and-whisker plots show the interquartile range (boxes) and the 10th and 90th percentile (whiskers) of the distribution for each of the four subpopulations (colors are identical to those in panel (**A**)).

As indicated by the EC_10_ POD values (see Table S7 for raw data, Table S8 for EC_10_ values, and Figure S4 for the concentration–response curves), a wide range of susceptibility to the individual chemicals (28 out of 42 had cytotoxic effects in at least one cell line) was observed across tested cell lines (Figure 4A), consistent with our previous observations [19,25]. The median EC_10_ values across all cell lines ranged from 14.6 μM to 261 μM. The intra-cell variability was lower in cell lines that were more susceptible. With respect to the mixtures, we observed a similar phenomenon, with the median EC_10_ values across all cell lines ranging from 12.8 to 282 μM (Figure 4B). There were highly significant correlations in sensitivity of the cell lines to chemicals and mixtures (*r*^2^ = 0.73, *p* < 0.001). Interestingly, no subpopulation was more susceptible than the others to either individual chemicals (Figure 4C) or mixtures (Figure 4D).

**Figure 5:**
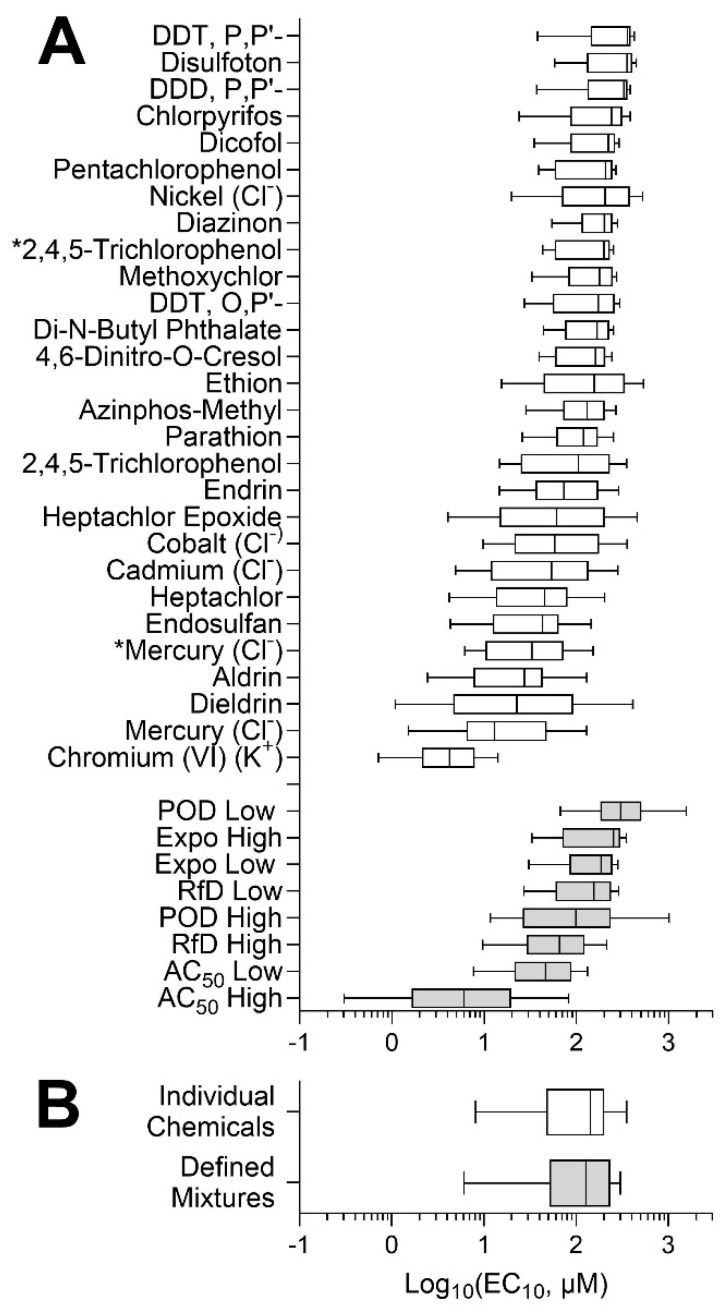
Concentration–response analysis of the inter-individual variability in chemical and mixture effects across all lymphoblast cell lines. (**A**) Distribution of the PODs for individual chemical (white) and defined mixtures (grey) is shown as box (interquartile range)-and-whiskers (5th and 95th percentiles) plots. Asterisks (*) denote compounds that were technical on-plate replicates. (**B**) Overall distribution of PODs for chemicals and defined mixtures.

Figure 5A shows the inter-individual variability in response to each active chemical and mixture, using the median posterior POD estimate for each individual. Wide variability was observed in each case with the full range of PODs from about 10-fold (e.g., disulfoton) to as much as 1000-fold (e.g., dieldrin). Of the 28 chemicals that had cytotoxic effects, 17 of them were pesticides, whereas none of the PAHs exhibited activity in the lymphoblasts, indicating one potential limitation of this in vitro model (e.g., lacking metabolism to cytotoxic moieties). The two chemicals with the lowest EC_10_ were heavy metals, mercuric chloride (median EC_10_ of 12.9 μM) and potassium chromate (median EC_10_ of 4.21 μM). Among mixtures, the “AC_50_ high” was the most cytotoxic mixture, as expected because it contained chemical concentrations at the upper 95th percentile of AC_50_ values across ToxCast assays; it was also the mixture with the largest variability across all cells. Overall, the median and distribution of PODs for individual chemicals and environmental mixtures were similar (Figure 5B).

### 3.3. Analysis of the Inter-Individual Variability in Effects of Chemicals and Mixtures

**Figure 6:**
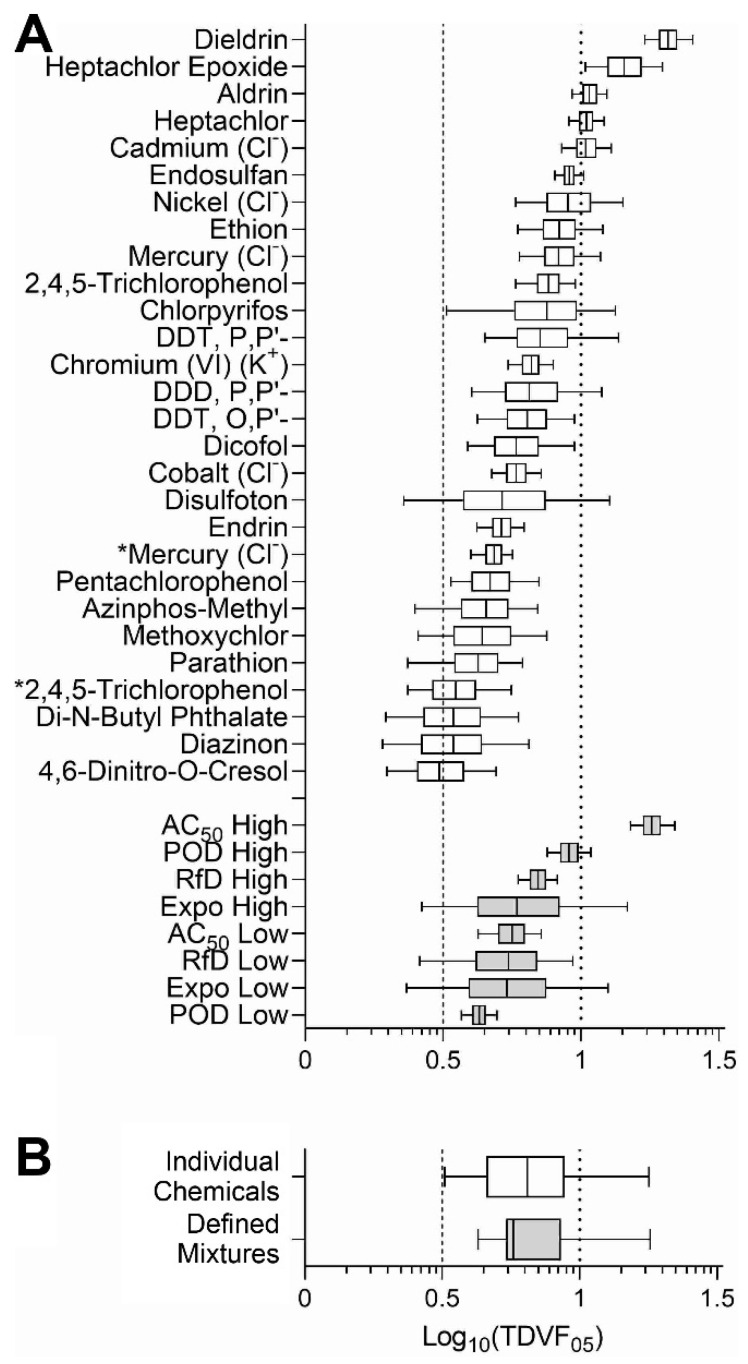
Distribution of TDVF_05_ for chemicals and mixtures across all cell lines. (**A**) Distribution of TDVF_05_ values for individual chemicals and mixtures is shown as box (interquartile range)-and-whiskers (5th and 95th percentiles) plots. Asterisks (*) denote compounds that were technical on-plate replicates. (**B**) Overall distribution of the TDVF_05_ for chemicals and mixtures. The vertical dashed line represents the default toxicodynamic variability factor of 10^1/2^ and the vertical dotted line represents a default total variability factor of 10.

It was previously shown that inter-individual variability is chemical-specific, both in human clinical studies [41] and in human in vitro models [19,21], often exceeding the default assumption of 10. Similar chemical-specific variability was observed in our study (Figure 5); therefore, we next quantified the range of the variability in the form of the toxicodynamic variability factor 5% (TDVF_05_) defined as the ratio of the POD (i.e., EC_10_ values) for the median individual to the POD for the most sensitive 5th percentile individual. Figure 6A shows the uncertainty distribution (based on posterior MCMC samples) of TDVF_05_ values for the active chemicals and all mixtures (see Table S9 for the median and range values). Most of the chemical and mixtures variability was within the default total inter-individual variability factor for toxicokinetics and toxicodynamics combined of 10, but all exceeded the default TDVF_05_ of 10^1/2^. The median TDVF_05_ for both chemicals and mixtures were similar (Figure 6B). For both chemicals and mixtures, there was significant inverse Pearson correlation (−0.79 and −0.41, respectively) between EC_10_ and TDVF_05_.

### 3.4. Genome-Wide Association Study (GWAS) Analyses

**Figure 7:**
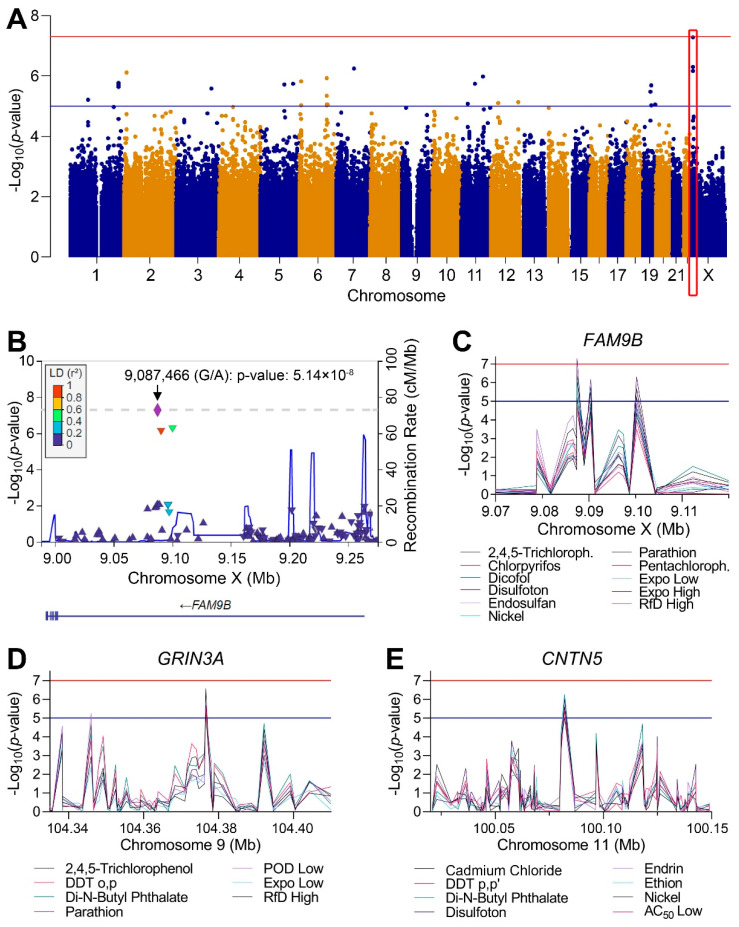
GWAS of population variability in cytotoxicity (covariate-corrected linear regression, see all raw data for each test chemical and mixture in Table S6). (**A**) A plot of the GWAS results for disulfoton (selected as a representative chemical) shows all analyzed SNPs arranged by their genomic position and corresponding -log10(p). Highlighted box (in red) demonstrates a locus that corresponds to the *FAM9B* gene. (**B**) A zoomed-in plot of the most significant region from A. (**C**) Plots of significance for SNPs in the 50kb region surrounding the *FAM9B* gene on chromosome X for chemicals and mixtures with overlapping GWAS results. (**D**,**E**) Plots similar to C for *GRIN3A* gene on chromosome 9 and *CNTN5* gene on chromosome 11. Blue horizontal line at 10^−5^ indicates the significance threshold for suggestive association for the cytotoxicity phenotype and the red horizontal line at 5 × 10^−8^ represents the corrected genome-wide significance threshold for all SNPs and phenotypes.

The design of the study and the use of these cell lines with available genotypes made it possible to conduct exploratory analysis of genome-wide associations between cytotoxicity (EC_10_ values) and polymorphisms. Linear regression GWAS analyses with correction for ancestry were performed for each chemical with effects and for mixtures, with no evidence of inflation of significance due to ancestry variations (Figures S2, S3 and S5). Multiple candidate genes with associations that were either suggestive (*p* < 10^−5^) or significant (*p* < 5 × 10^−8^) of an association relationship between polymorphic loci and susceptibility to cytotoxicity were identified. Figure 7A shows an example genome-wide plot for disulfoton. A large number of loci exceed the suggestive threshold and one region located on chromosome X reached the significant threshold (Table S10). Figure 7B shows a zoomed-in plot of the 0.25 Mb region where the most significant association was recorded, the locus encodes for the Family with Sequence Similarity 9 Member B (*FAM9B*) gene. The most significant polymorphism in this locus were in linkage disequilibrium with a number of other polymorphisms. Interestingly, the same region was identified with suggestive associations for a number of other chemicals and mixtures (Figure 7C), among those were other pesticides and exposure-based mixtures. Similarly, high concordance among chemicals that “hit” the same locus were observed for a number of other genomic regions (Table S10), two additional examples are shown for the Glutamate Ionotropic Receptor NMDA Type Subunit 3A (*GRIN3A*) on chromosome 9 (Figure 7D) and Contactin 5 (*CNTN5*) gene on chromosome 11 (Figure 7E). The SNPs, *p*-values, and regression coefficients (beta values), are provided in Tables S6 and S11, respectively.

Results from the alternate mixed model approach were similar to the linear regression analyses, with most analyses showing rank correlation >0.9 between the *p*-value of the two approaches Figures S3 and S5. However, the mixed model approach posed somewhat greater challenges in appropriate control of genomic significance inflation (genomic control lambda [42] outside the range 0.9–1.1), and so we consider these results to be used as auxiliary information.

## 4. Discussion

For most chemicals, both individually and as components in mixtures, there are little if any empirical data on the degree of human inter-individual variability or its genetic underpinnings [43]. Estimation of the degree of inter-individual variability in the population is a required step in assessment of human health hazards from chemicals [44], it is also desired in higher tier mixture assessments [45]. Options to fill data gaps in refining inter-individual variability estimates for specific substances, rather than relying on default assumptions, exist using either modeling or experimentation. The former is most developed for estimating toxicokinetic variability and is largely based on the knowledge of polymorphisms and associated functional heterogeneity of xenobiotic metabolism genes in the human population [46,47,48,49]. Whilst most toxicokinetic models are designed to evaluate single chemicals, the applications of these models to combined exposures and mixtures have also been demonstrated [50,51]. Population-based models to collect chemical-specific data through new experiments comprise both human and animal cell-based options, as well as genetically-diverse and -defined panels of rodents [16]. Studies in multi-strain panels of mice are time- and resource-consuming and are impractical as a tool for collecting experimental data on a large number of chemicals, or on whole mixtures. In vitro-based models are now widely used to provide both hazard and mode of action information [52,53,54,55]; still, few cell-based assays that are now routinely used in the large-scale toxicity screening programs are designed to address inter-individual variability [56]. Importantly, the availability of the genetically-diverse and -defined renewable sources of human cells, such as lymphoblasts from the HapMap [57] and 1000 Genomes [58] projects, enables in vitro toxicity testing at the population scale. Indeed, the feasibility of the in vitro population-based approach has been demonstrated for hundreds of chemicals [19,21,59] and several mixtures [22]. Therefore, we aimed to extend the use of novel human population-based in vitro models for characterization of inter-individual variability to understand differences between individual chemicals and mixtures.

Mixture assessments are far more complex than those for the individual chemicals; risk assessors are primarily concerned with characterization of the chemical composition of mixtures and data availability on their components of concern. Still, the inter-individual variability does factor into the outcome of these assessments at the risk characterization step where the margin of exposure or hazard index is typically derived based on the default assumptions [3,7,45]. A previous study with the in vitro model identical to that used herein showed that toxicodynamic variability could be experimentally-derived for two defined mixtures of pesticides [22]. These data were informative for comparing not only the potencies, but also the variability of two mixtures tested, thus providing useful data for risk characterization. However, the previous study was essentially a whole mixture investigation and was not suitable for a component-based assessment of the variability because it did not test the components of those mixtures. Indeed, experimental studies of chemical mixtures are few in spite of the agreement that cell-based testing may hold promise in reducing uncertainties in assessment of the health effects of mixtures [60]. The lack of such data is largely due to combinatorial complexity of creating defined mixtures for experimental testing; this formidable challenge has been addressed through studies of binary [61,62] to more multi-factorial [26,63] mixtures.

We reason that our study is a valuable case example for how a population-based human in vitro model could be applied in the assessment of mixtures. First, we show that for the diverse chemicals that were tested in lymphoblast cell lines, these cells, while limited in their metabolic capacity, had comparable ranges of cytotoxicity EC_10_ values to those in different human iPSC-based models (Figure S6). In the case of the defined mixtures, the cytotoxic range across the lymphoblast cell lines was more conservative in comparison to the other in vitro models; however, the overall range in cytotoxicity overlapped across various in vitro models (excluding iPSC-derived neurons and endothelial cells). These findings are important because lymphoblast cells are far easier to culture than iPSC-based models and they can be passaged (e.g., amplified), while the former are terminally differentiated cells. We suggest that large-scale initial screening studies can be done in lymphoblasts without sacrificing the sensitivity for establishing conservative hazard estimates, while also providing very useful information on the potential inter-individual variability. Population size for future screening studies that may not need to involve genetic mapping will be informative with samples sizes of as few as ~20 individuals; follow-up experiments, if needed to establish high confidence in chemical-specific variability estimates, may be performed using sample sizes of ~50–100 [30].

Second, we confirm previous observations [22] that the variability estimates from this in vitro model are close to default for most compounds tested, yet the high-variability chemicals and mixtures can be flagged. This finding is reassuring as it provides additional empirical evidence to human in vivo data from clinical and epidemiological studies [64], and data from population-based in vitro studies with human lymphoblasts [19], as well as other cell types such as fibroblasts [59] and iPSC-derived cardiomyocytes [31]. Furthermore, our finding that variability estimates for the mixtures were in range with those of the individual chemicals suggests that the variability may not be additive or multiplicative in the context of combined exposures. This finding needs to be verified with a formal analysis of variability reconstruction. Future work will focus on using the dose–response data from the mixtures to evaluate concentration addition and independent action assumptions and determine if dose reconstruction can be used to quantify the variability in addition to calculating the PODs for the mixtures. Further, the individual chemical data can potentially be used to reconstruct other mixtures as well as determine chemical-specific TDVF_05_ that can be used in other assessments.

Although lymphoblasts lack robust metabolic capacity, they are proliferative cells and can serve as a sensible in vitro model when considering effects of the chemicals that may cause adverse effects by disturbing key toxicity pathways such as cell cycle regulation, DNA repair, and membrane transport [17]. Indeed, the genome-wide association analyses identified a number of candidate susceptibility loci in these pathways. It is noteworthy that a large number of these loci were concordant across multiple chemicals and mixtures, this indicates potential similarity in downstream cell signaling pathways that may have been perturbed by the tested compounds.

On the one hand, the similarities in loci of susceptibility may be used as additional information for grouping chemicals for mixture “assessment groups” [7]. Specifically, the guidance on scientific criteria for grouping chemicals into assessment groups for human risk assessment of combined exposure to multiple chemicals calls for the use of “mechanistic information on toxicity as the gold standard where available (i.e., common mode of action or adverse outcome pathway) through a structured weight of evidence approach.” We propose that similarity in the susceptibility genes may be a reasonable indication of similarity of the mechanisms of toxicity in absence of other data.

On the other hand, the candidate genes are also of interest because they indicate common pathways of susceptibility that may also be of importance in vivo. For example, the genome-wide association analysis identified *FAM9B*, *CNTN5*, and *GRIN3A* as common putative susceptibility loci across eleven, eight, and seven chemicals or mixtures, respectively. Even though these candidate genes lack evidence regarding their potential role in toxicity pathways, the information available on their general function is highly relevant to cell stress responses. The *FAM9B* gene plays a role in regulating cell division as it codes for a protein in the synaptonemal complex that plays a critical role in meiosis in germ cells [65]. *FAM9B* and related genes have been shown to be upregulated in tumor cells [66] and also induced by DNA damaging agents [67]. *CNTN5* is a protein coding gene that functions to mediate cell adhesion interactions during neural development and has been linked to inflammation [68]. Recently, it was identified as a potential pharmacogene that may affect drug clearance [69], albeit the association has not been independently verified. *GRIN3A* is a gene encoding an ion channel that is critical during neural development [70]. A study of organophosphates toxicity in mice identified increased expression of the *GRIN3A* gene after treatments with chlorpyrifos and diazinon [71]. Another candidate gene, *FANCA*, was a suggestive locus for susceptibility to a number of heavy metals including cadmium chloride, cobalt chloride, and mercuric chloride. Interestingly, the *FANCA* gene is involved in DNA replication, has high expression in lymphoblasts, and is rich in cysteine residues which have a strong affinity for heavy metals [72]. The *FANCA* gene polymorphisms are known to affect the cell’s capacity for inter-strand crosslinking in the process of DNA replication [73]. We emphasize that the GWAS findings, for this limited sample size, should be interpreted with caution; still, the results provided herein can be used by future researchers for replication or inclusion in meta-analyses.

This study has a number of limitations. Specifically, this model system is reflective of acute high-dose treatments that may not be indicative of the potential repeat exposure effects. Recapitulating chronic exposure or repeat dosing regimens presents a challenge for in vitro models in general, and it has been suggested that studies that aim to characterize the toxicodynamics of a response need to not only be designed in terms of concentration–response, but also include repeat exposures [74]. Another limitation is that the likely route of exposure to these chemicals and mixtures was not incorporated into the experimental design. In addition, for the chemicals thar require metabolic activation (e.g., polycyclic aromatic hydrocarbons), any in vitro model that lacks metabolism may be insufficient unless additional measures are taken [75]. However, to address these limitations, future work can study quantitative in vitro to in vivo extrapolation and use in vitro data coupled with preexisting exposure data to calculate oral doses that can be compared to chronic exposure scenarios while addressing the relevant routes of exposure [76]. The unknown composition of mixtures, health effects, and exposure assessment present challenges in mixtures risk assessment. In particular, understanding and screening realistic exposure scenarios has recently become more feasible with the increased availability and accessibility to monitor and model environmental exposures [77]. As more exposure data becomes available, these concepts of defined mixtures can be applied for toxicity screenings to conduct region-specific exposure assessments. Furthermore, while this study included a number of chemical classes, it is still limited in representing other important classes of toxicants and mixtures. Still, future work can apply a population-based approach to test mixtures of other chemicals of concern.

In summary, our data shows that population variability of the mixtures is unlikely to exceed that of the most variable component, but further work using additivity models is needed to better understand the extent to which population variability of mixtures can be reconstructed from variability in their components. Additionally, we found that similarity in genome-wide associations among components may be informative for mechanism-based grouping of constituents in a mixture for cumulative assessments. Overall, this study demonstrates that a human population-based in vitro model of lymphoblastoid cell lines is a reasonable approach to quantify inter-individual variability and can also be applied to mixtures, thus potentially reducing uncertainties associated with complex exposure scenarios, and helping to fill a critical gap in cumulative risk assessment.

## Data Availability

All pertinent data are included in Supplementary Materials. Table S6 is available at https://doi.org/10.5281/zenodo.6784584.

## Supplementary Materials

The following are available online at https://www.mdpi.com/article/10.3390/toxics10080441/s1, Figure S1: Plate design layout, Figure S2: QQplots, Figure S3: Manhattan plots with mixed model analyses, Figure S4: Cell-line specific concentration–response curves, Figure S5: Chemical and mixture-specific Manhattan plot visualizations, Figure S6: Cytotoxicity comparisons for chemicals and mixtures across various in vitro models, Table S1: Chemical identification information, Table S2: Individual lymphoblast cell line identification, Table S3: Defined mixture information, Table S4: Pearson correlation coefficients for intraplate replicates across all cell lines, Table S5: Pearson correlation coefficients for interplate replicates for all cell lines, Table S6: SNP ID and corresponding *p*-values for chemicals (with effect) and all mixtures, Table S7: Chemical and mixture raw and normalized cytotoxicity data across all cell lines, Table S8: Median EC_10_ values for individual chemicals and mixtures, Table S9: Chemical and mixtures toxicodynamic variability factor summary data, Table S10: Genome-wide association study candidate genes, corresponding *p*-values, Table S11: Genome-wide association study SNPs with beta coefficients.

## Abbreviations

AC_50_ high/low, active concentration 50%; CEU, Utah residents with European ancestry; *CNTN5*, Contactin 5; DMSO, dimethyl sulfoxide; EC_10_, effective concentration at which a 10% change in cytotoxicity in comparison to vehicle; Expo high/low, exposure; *FAM9B*, family with Sequence Similarity 9 Member B; *FANCA*, Fanconi Anemia Complementation Group A; GBR, British from England and Scotland; *GRIN3A*, Glutamate Ionotropic Receptor NMDA Type Subunit 3A; GWAS, genome-wide association study; HPV, high-production volume chemicals; iPSC, induced pluripotent stem cells; LCLs, lymphoblast cell lines; MAF, minor allele frequency; MCMC, Markov chain Monte Carlo; PAHs, polycyclic aromatic hydrocarbons; POD, point of departure; QQplots, quantile-quantile plots; RfD high/low, reference dose; SNPs, single nucleotide polymorphisms; TDVF, toxicodynamic variability factor; TSI, Tuscans in Italy; YRI, Yoruban from Ibadan, Nigeria.
