# Supplementary material for: A Population-Based Human In Vitro Approach to Quantify Inter-Individual Variability in Responses to Chemical Mixtures
Source: Toxics. 2022 Aug 1;10(8):441. doi: 10.3390/toxics10080441 (PMC9413237; doi:10.3390/toxics10080441)
Supplement: Supplementary file 1 [file toxics-10-00441-s001.zip › Figure S2 QQ Plots.pdf]

2,4,5-TRICHLOROPHENOL

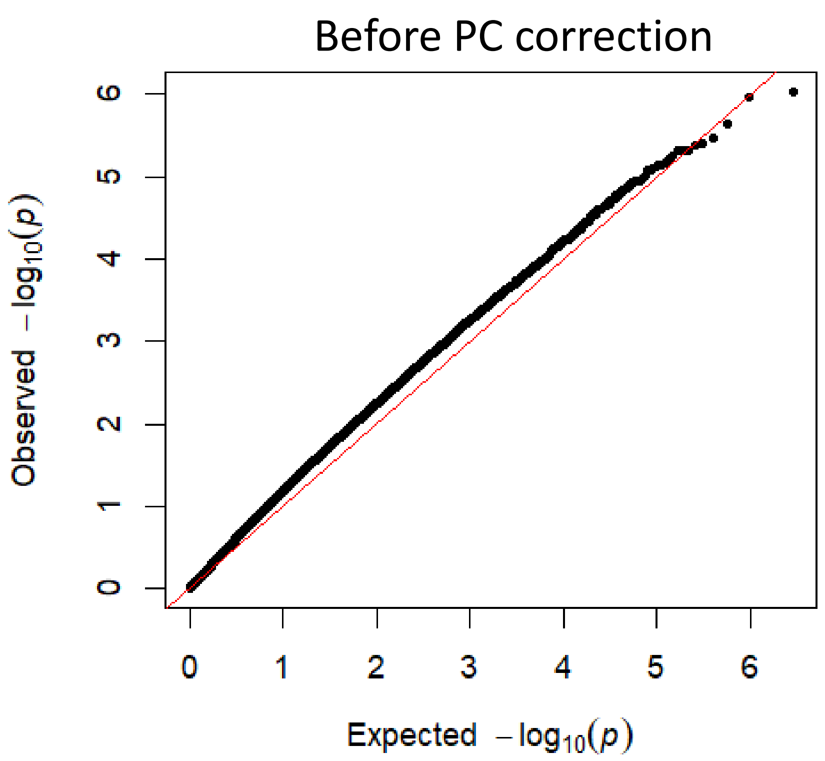

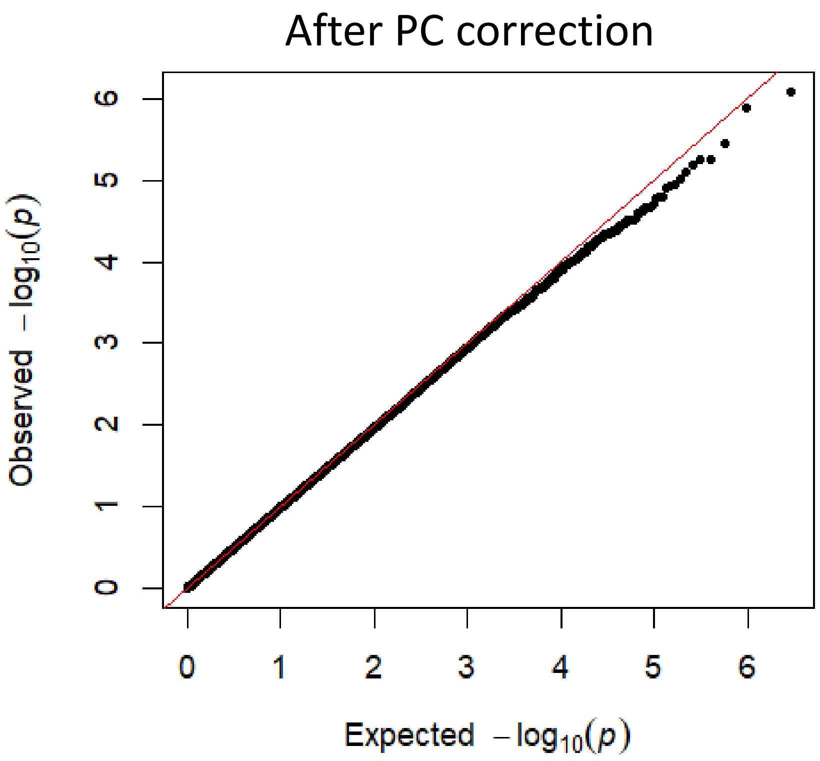

2,4,5-TRICHLOROPHENOL-2

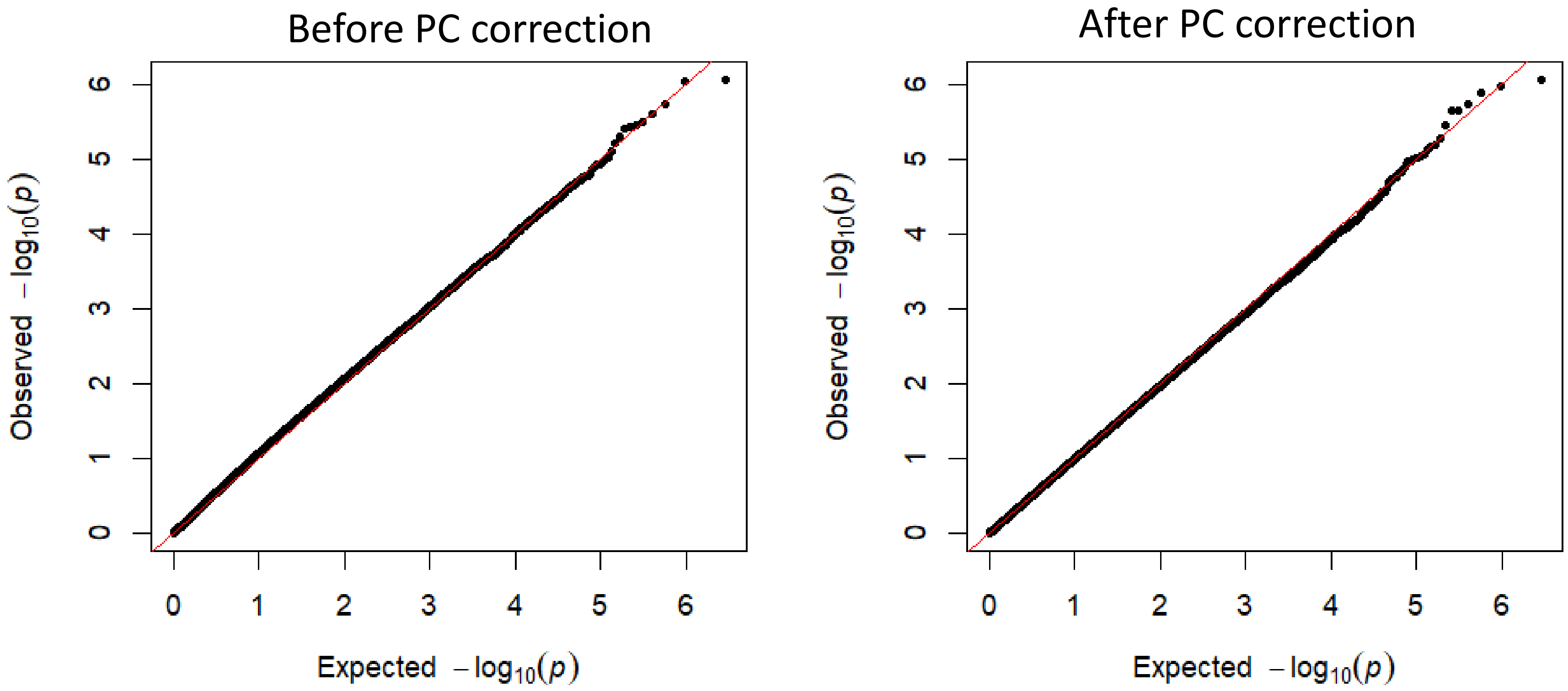

4,6-DINITRO-O-CRESOL

Before PC correction

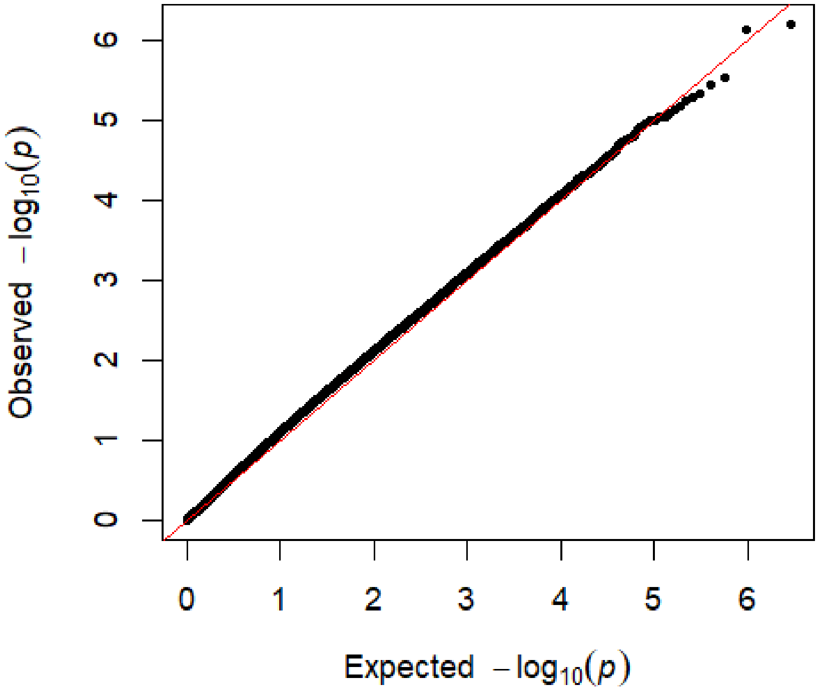

After PC correction

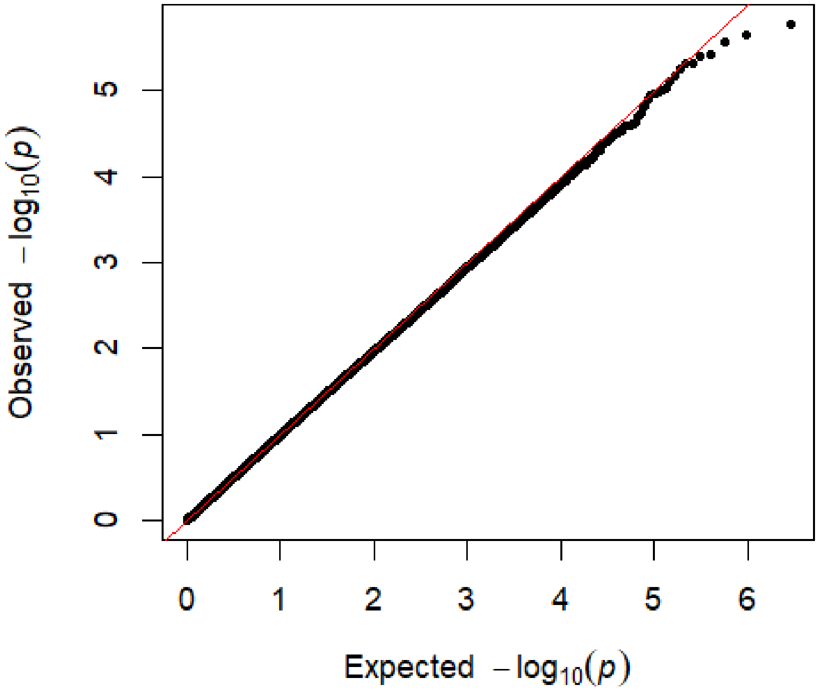

ALDRIN

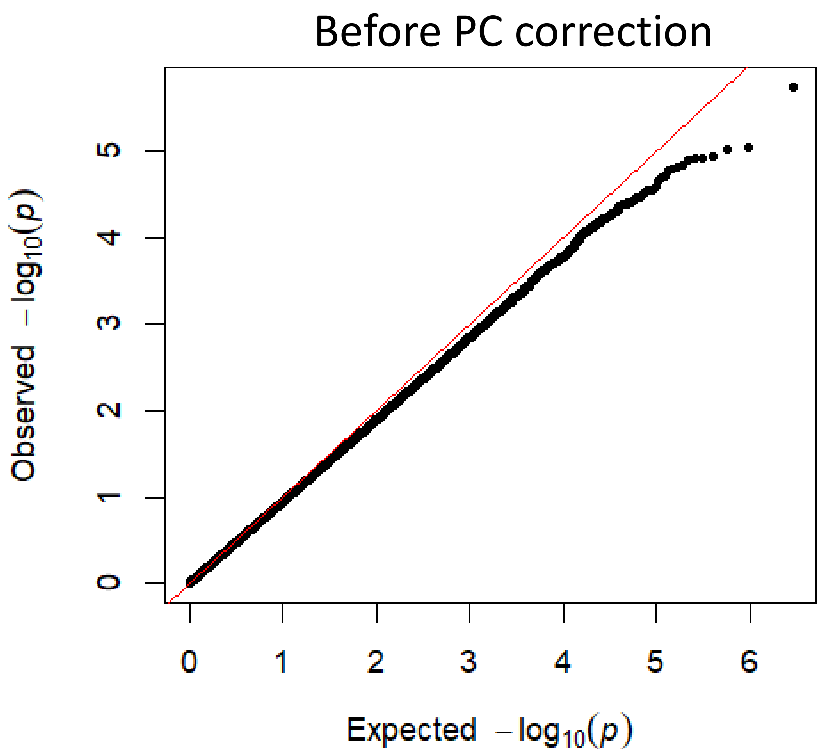
Before PC correction
Observed $-\log_{10}(p)$
0
1
2
3
4
5
0
1
2
3
4
5
6
Expected $-\log_{10}(p)$

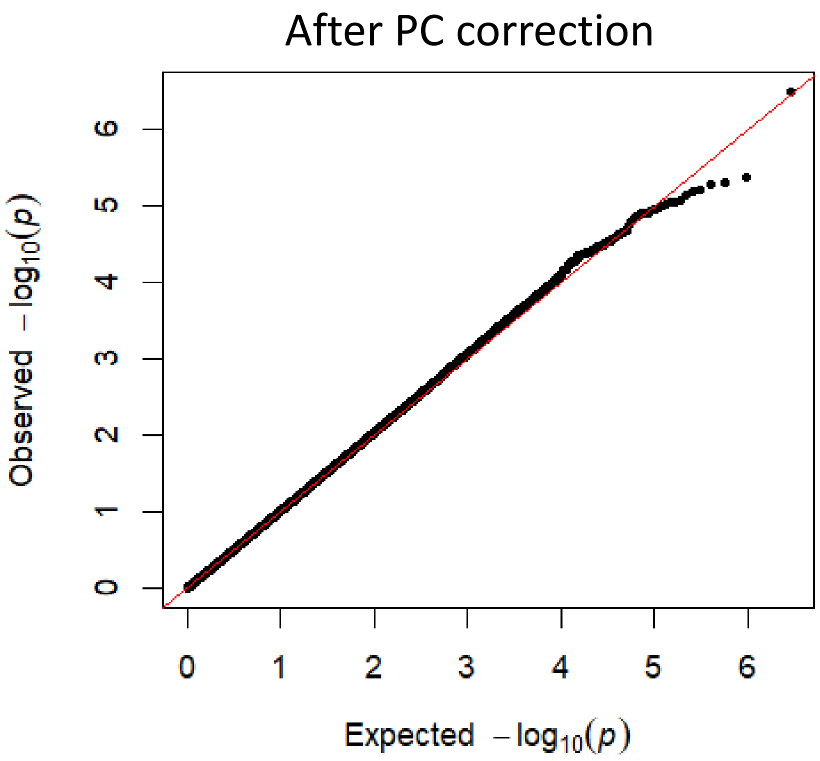
After PC correction
Observed $-\log_{10}(p)$
0
1
2
3
4
5
6
0
1
2
3
4
5
6
Expected $-\log_{10}(p)$

AZINPHOS-METHYL

Before PC correction

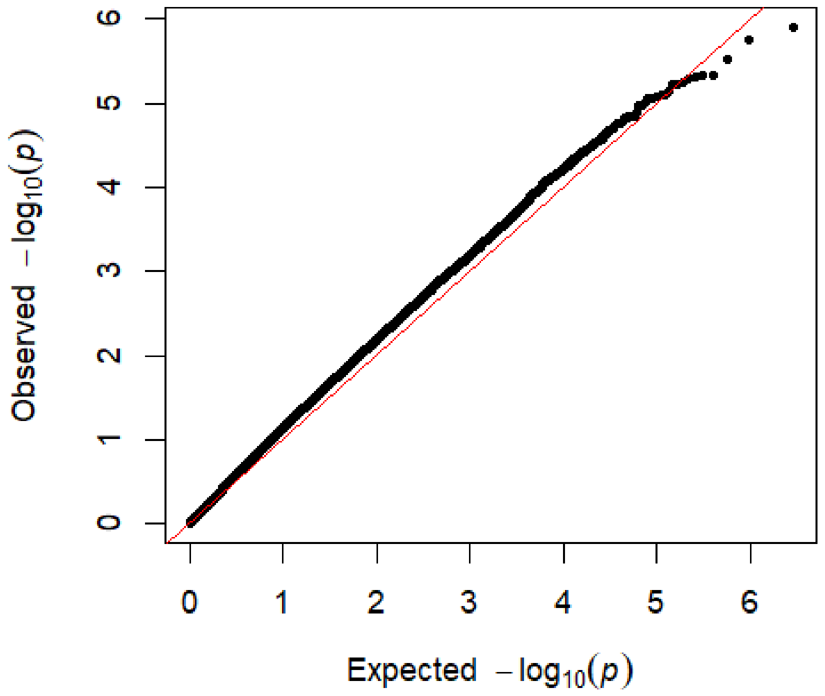

After PC correction

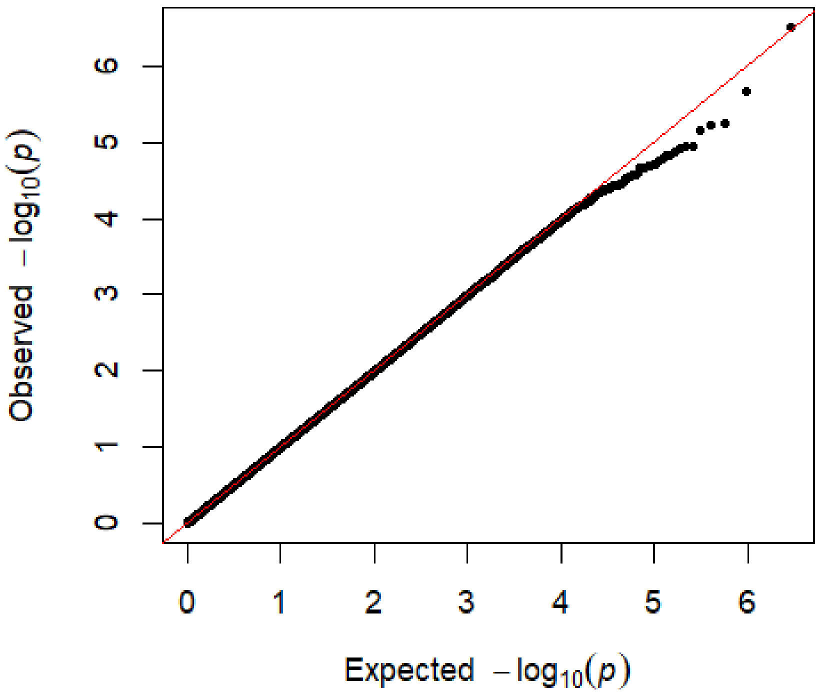

CADMIUM(Chloride)

Before PC correction

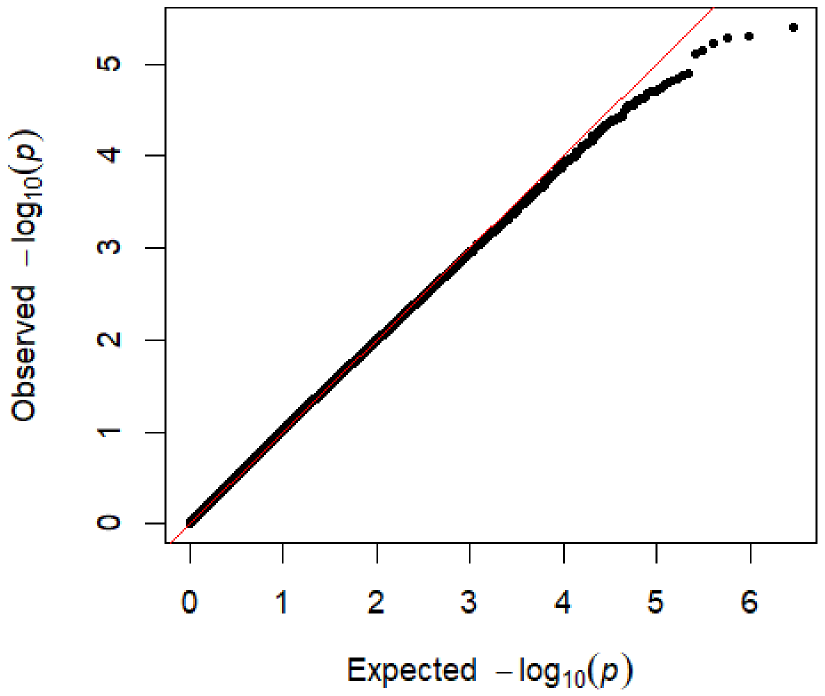

After PC correction

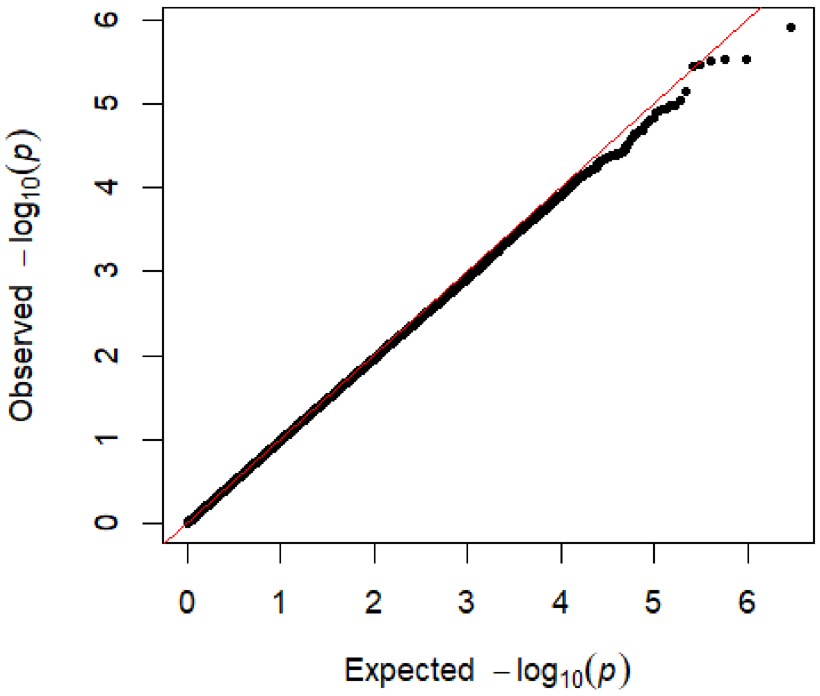

CHLORPYRIFOS

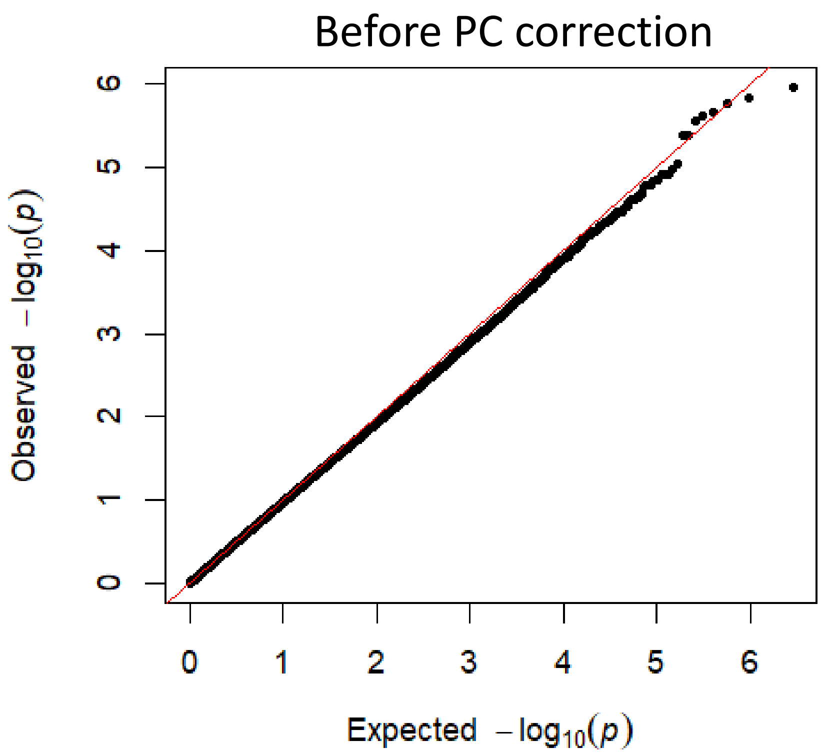

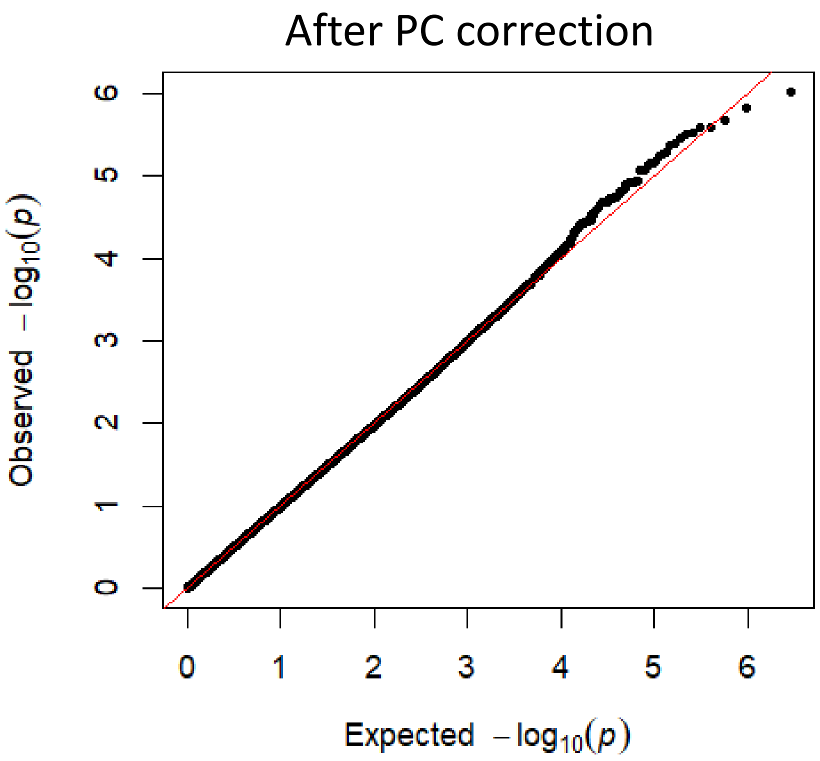

COBALT

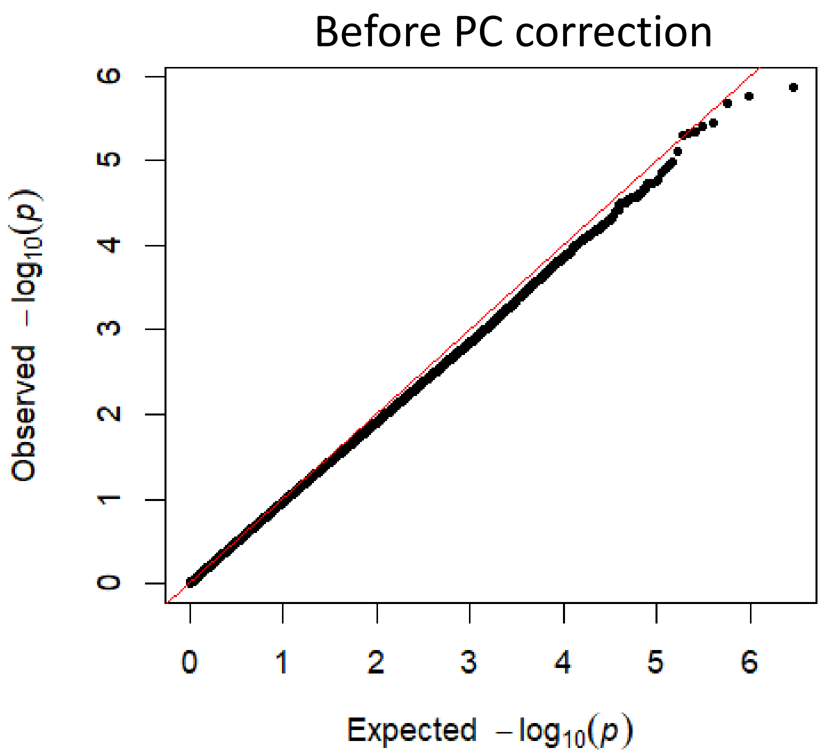
Before PC correction
Observed $-\log_{10}(p)$
0
1
2
3
4
5
6
Expected $-\log_{10}(p)$
0
1
2
3
4
5
6

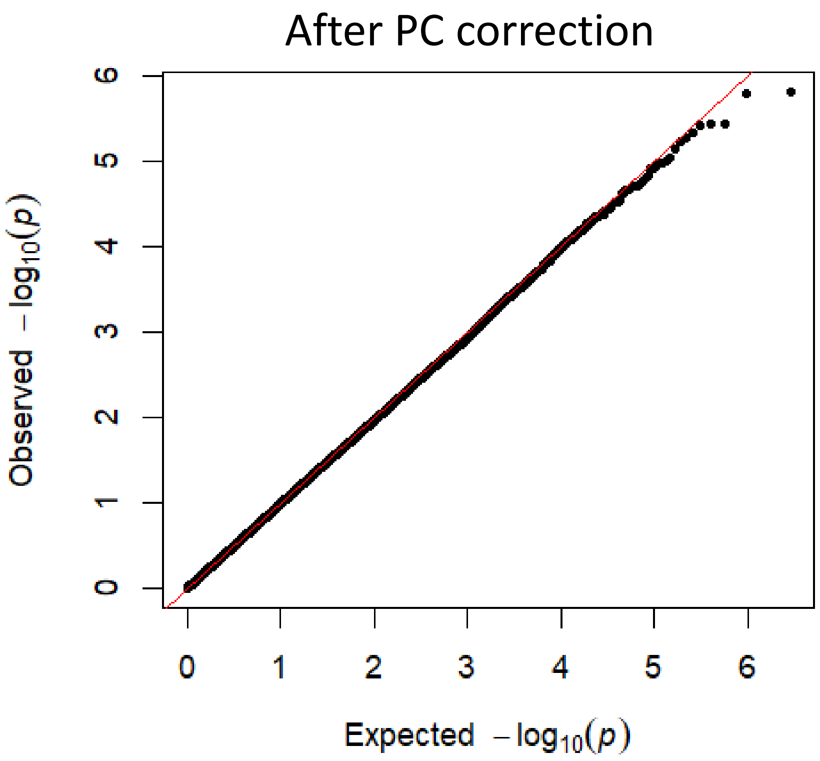
After PC correction
Observed $-\log_{10}(p)$
0
1
2
3
4
5
6
Expected $-\log_{10}(p)$
0
1
2
3
4
5
6

DDD, P,P'-

Before PC correction

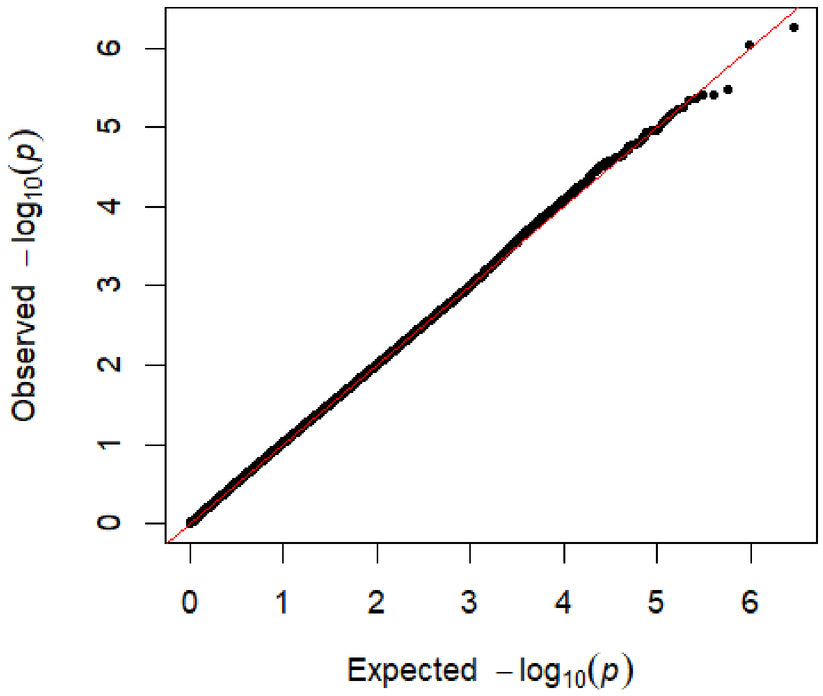

After PC correction

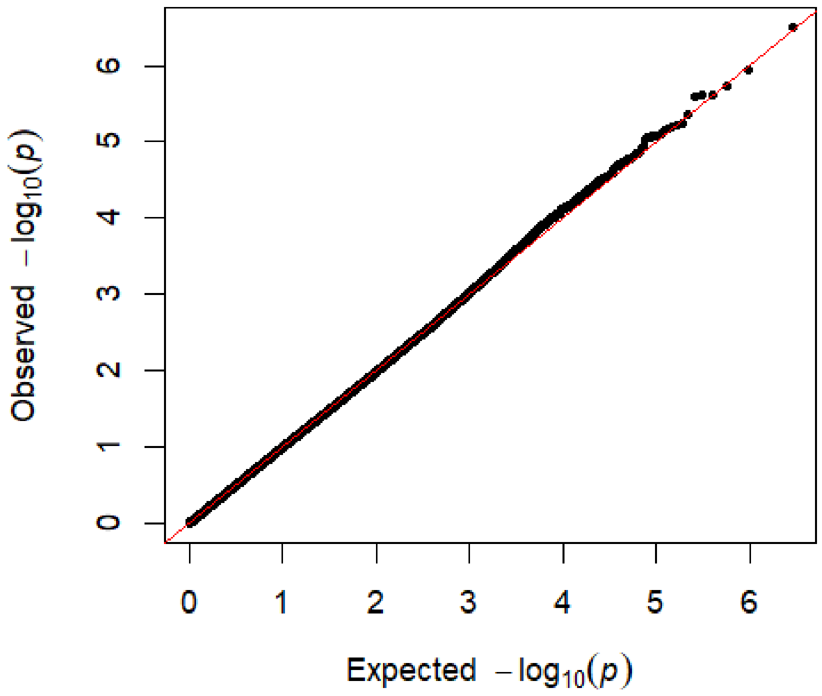

DDT, O,P'-

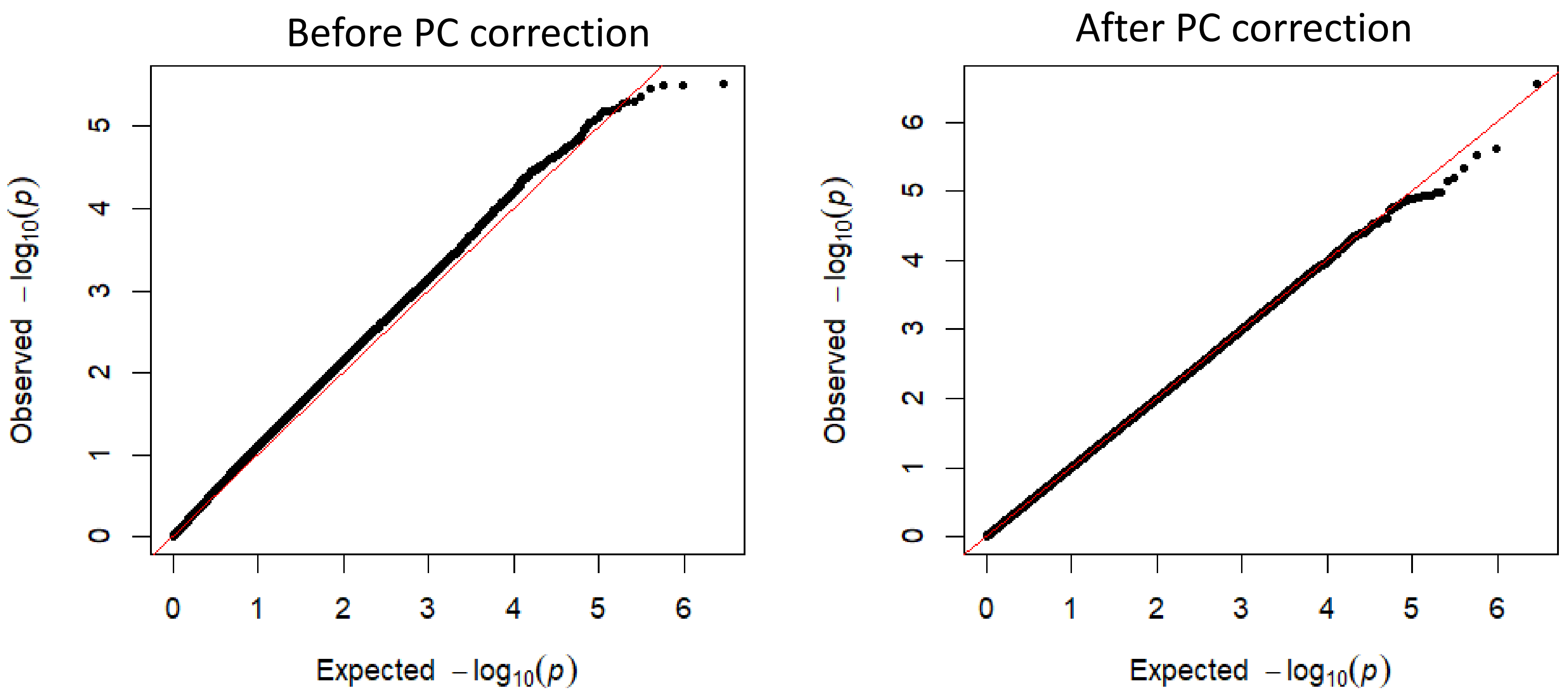
Before PC correction
After PC correction
Observed − $\log_{10}(p)$
Expected − $\log_{10}(p)$
0
1
2
3
4
5
6

DDT, P,P'-

Before PC correction

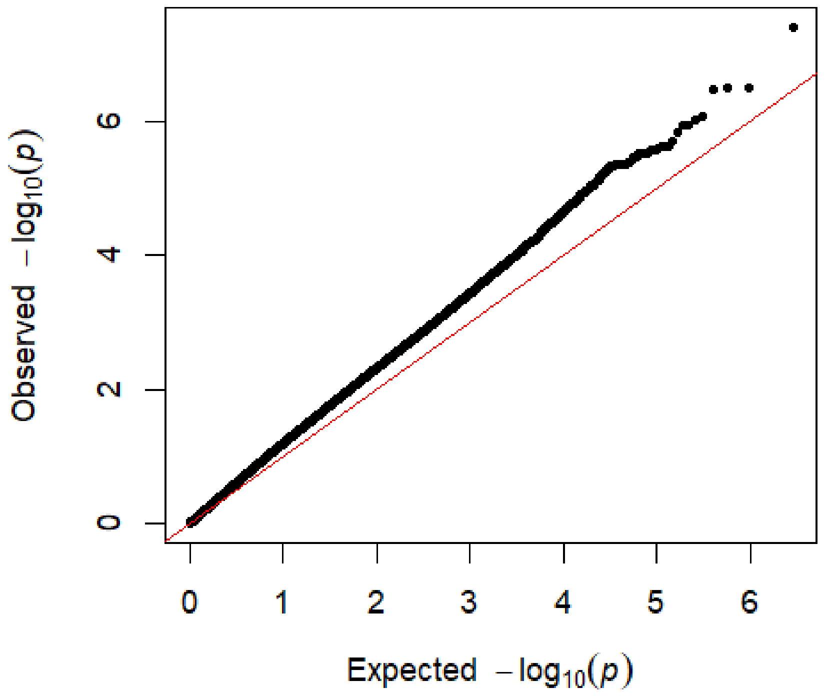

After PC correction

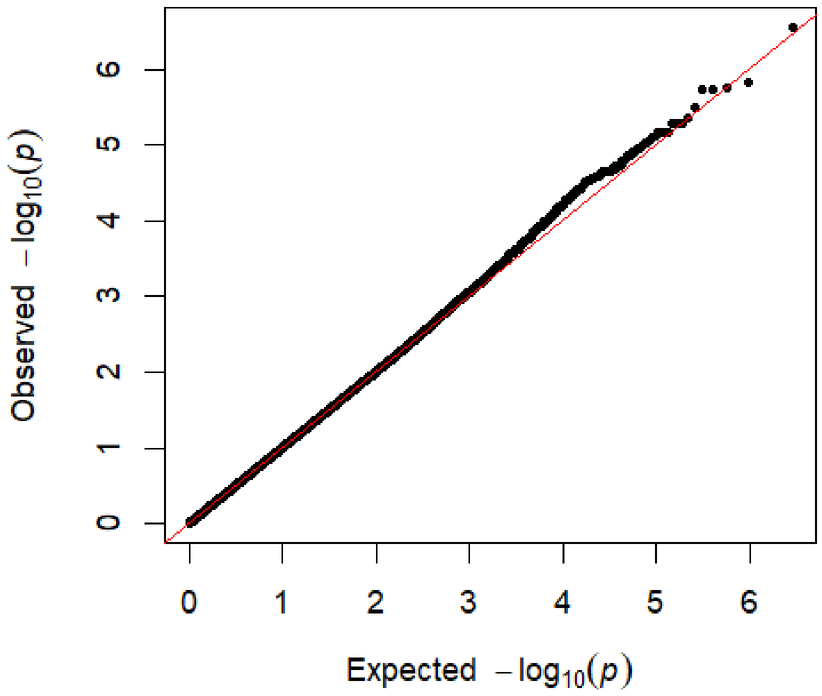

DI-N-BUTYL PHTHALATE

Before PC correction

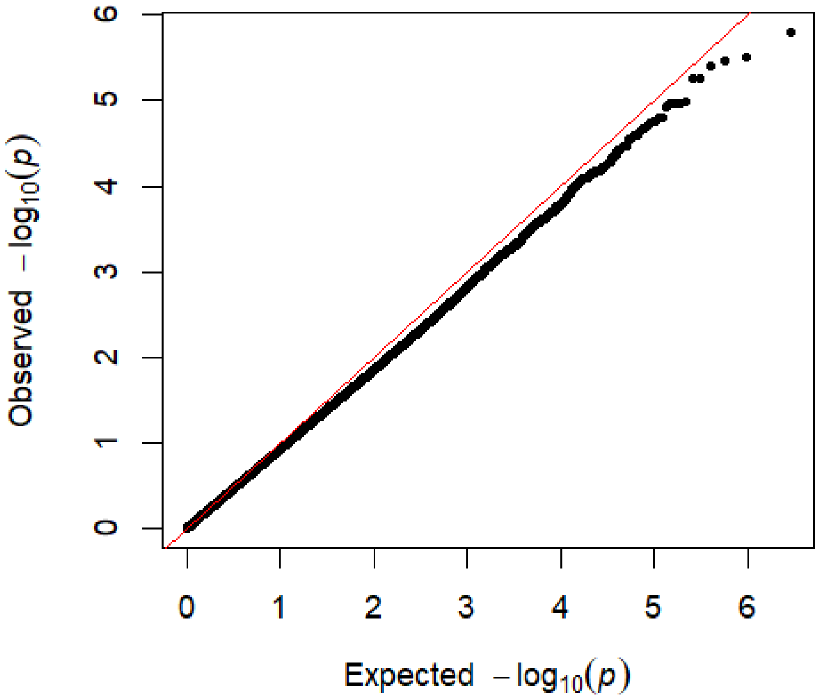

After PC correction

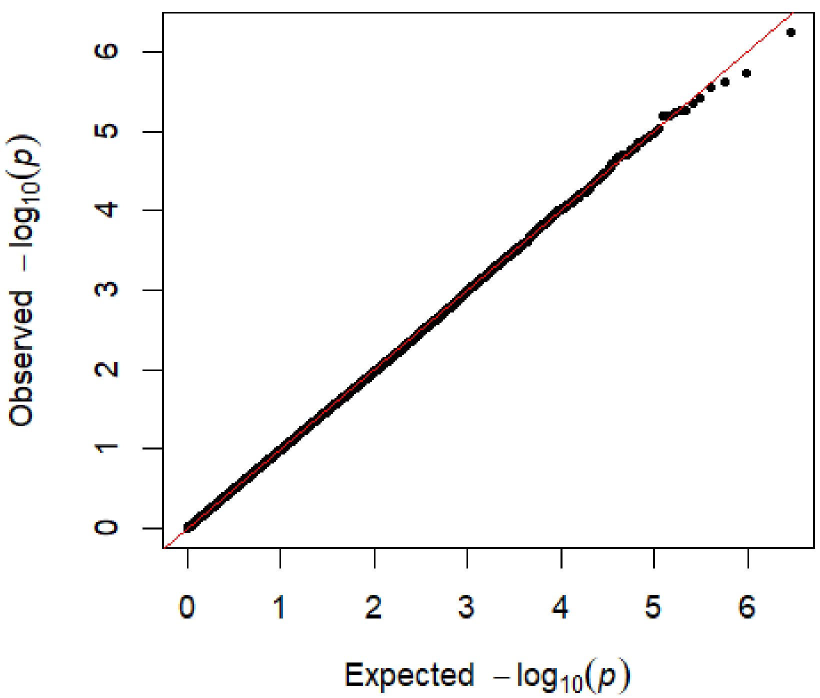

DIAZINON

Before PC correction

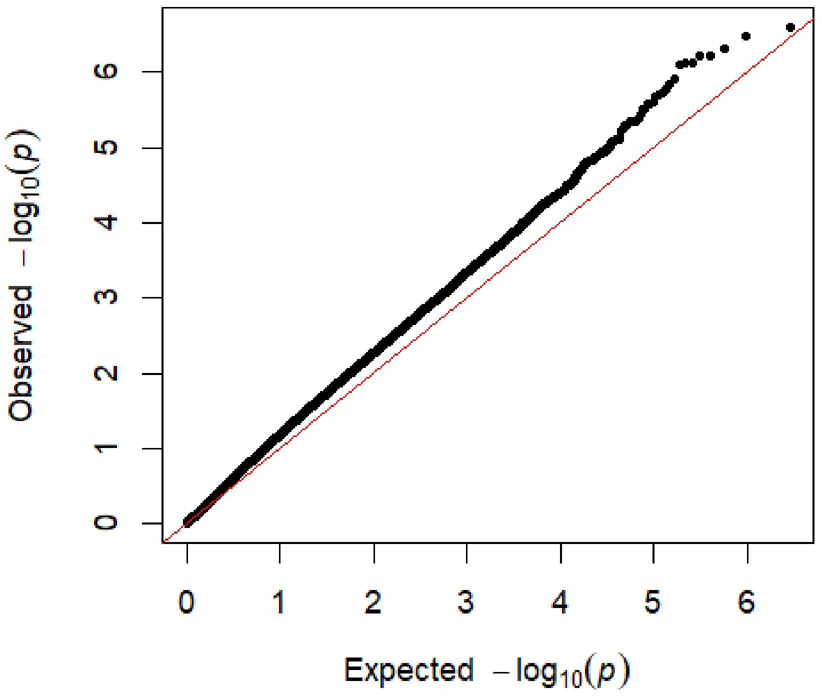

After PC correction

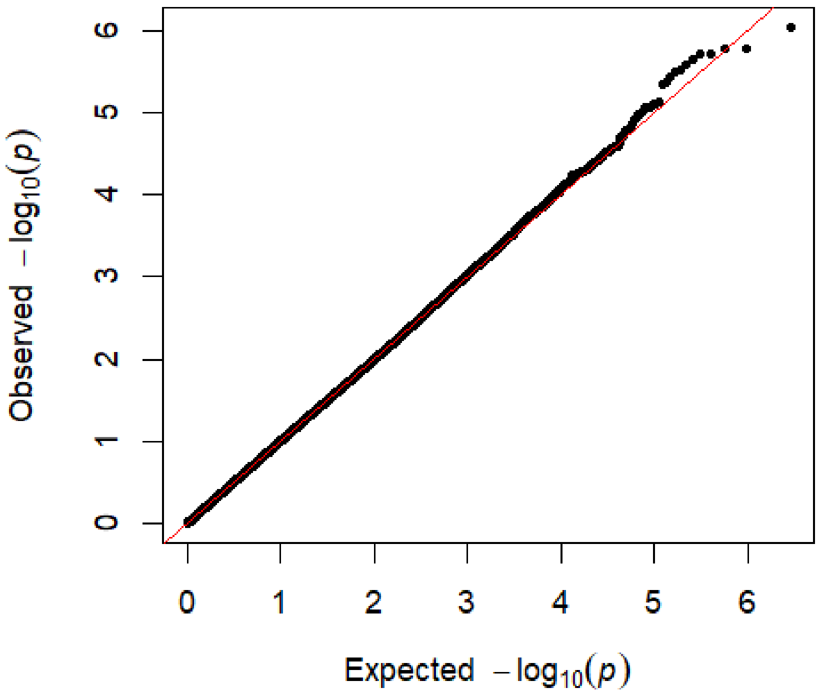

DICOFOL

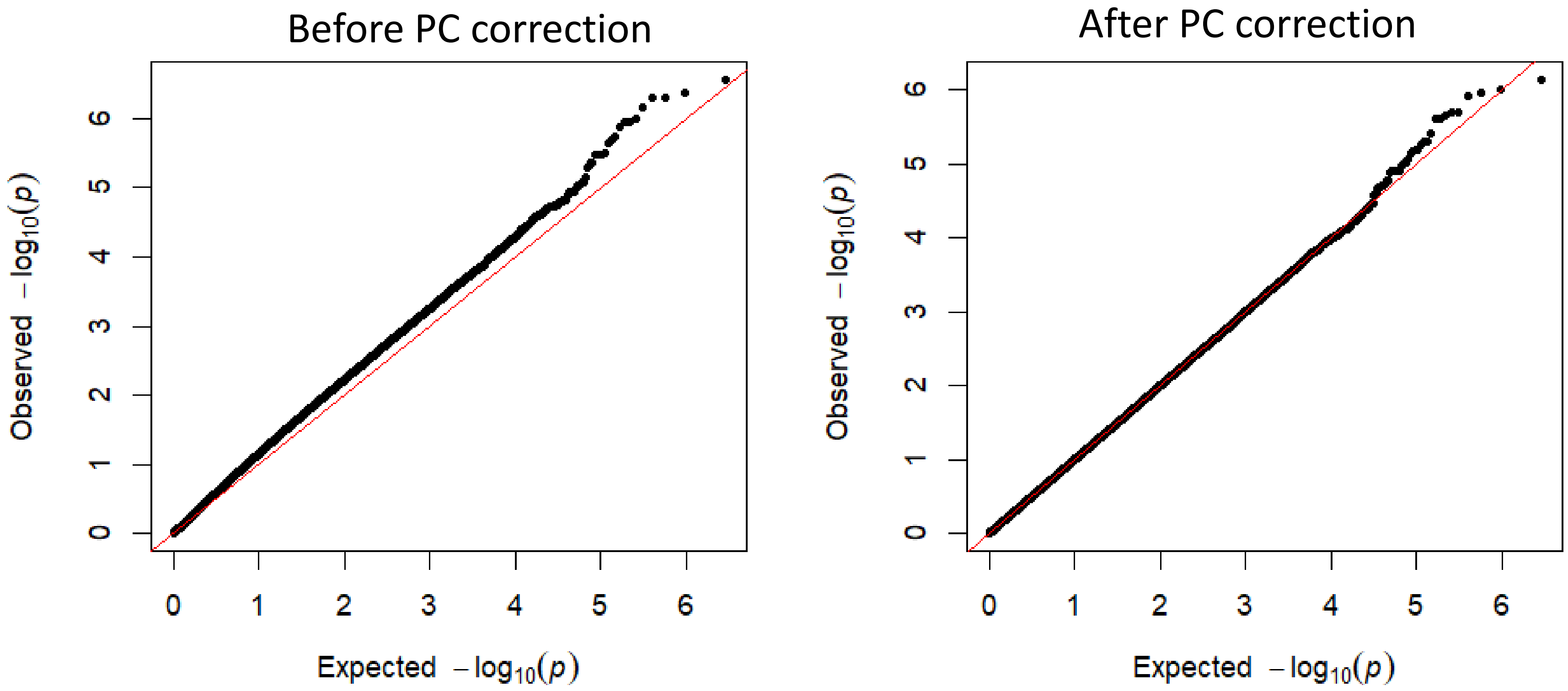
Before PC correction
After PC correction
Observed −log10(p)
Expected −log10(p)
0
1
2
3
4
5
6

DIELDRIN

Before PC correction

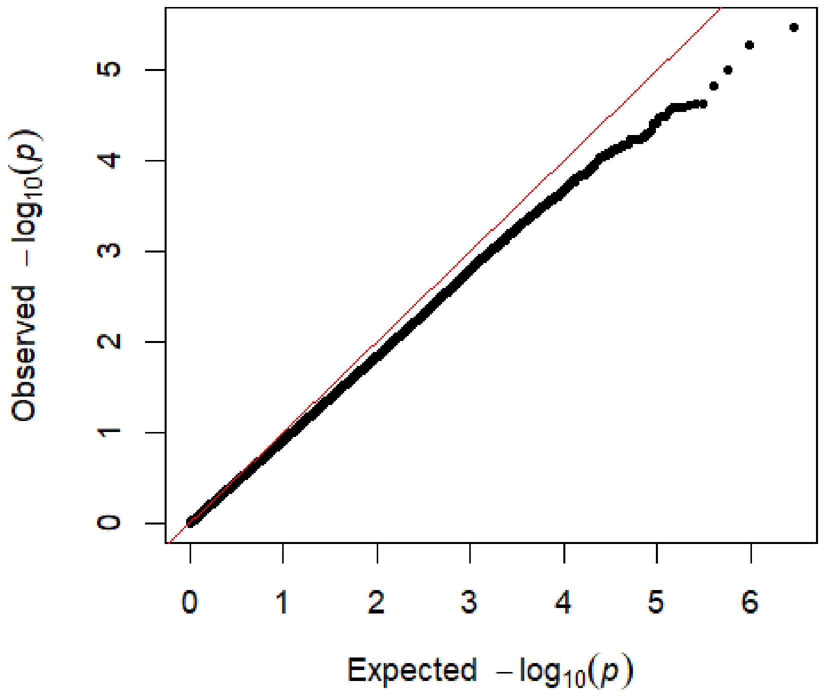

After PC correction

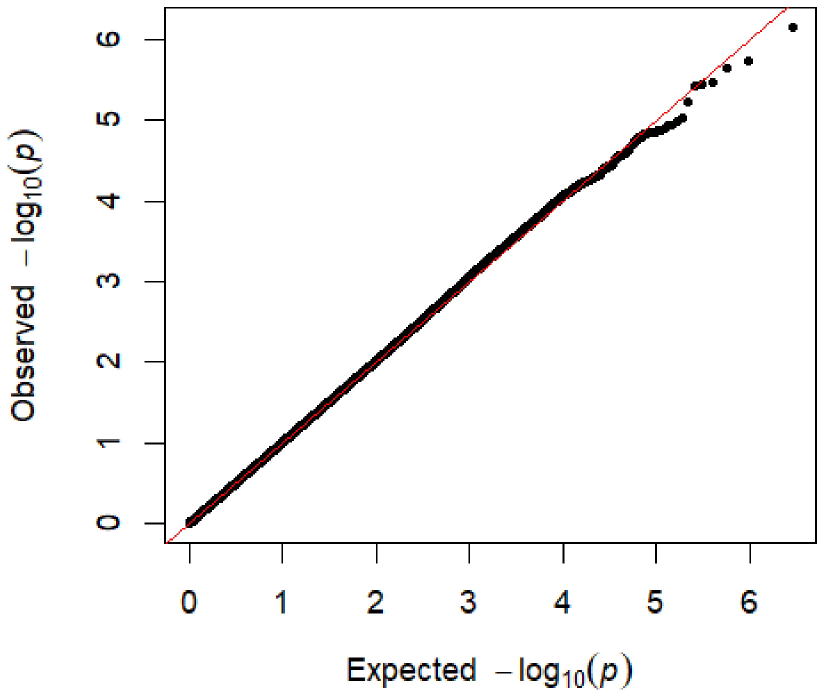

DISULFOTON

Before PC correction

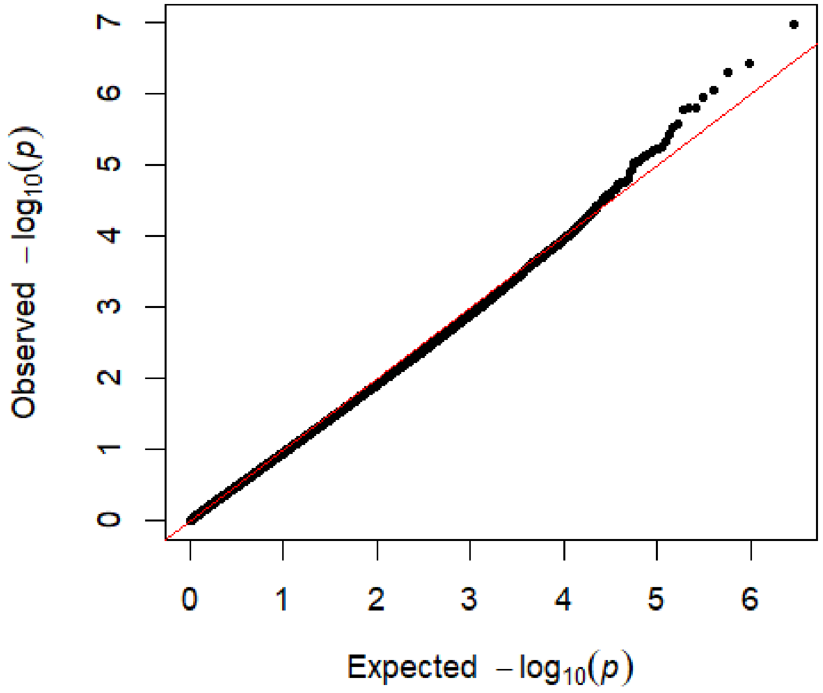

After PC correction

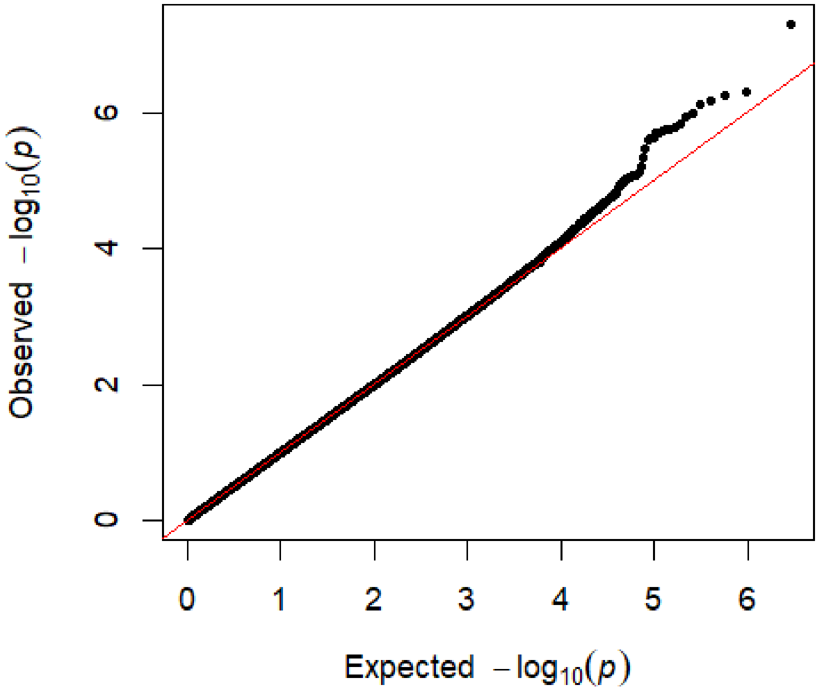

ENDOSULFAN

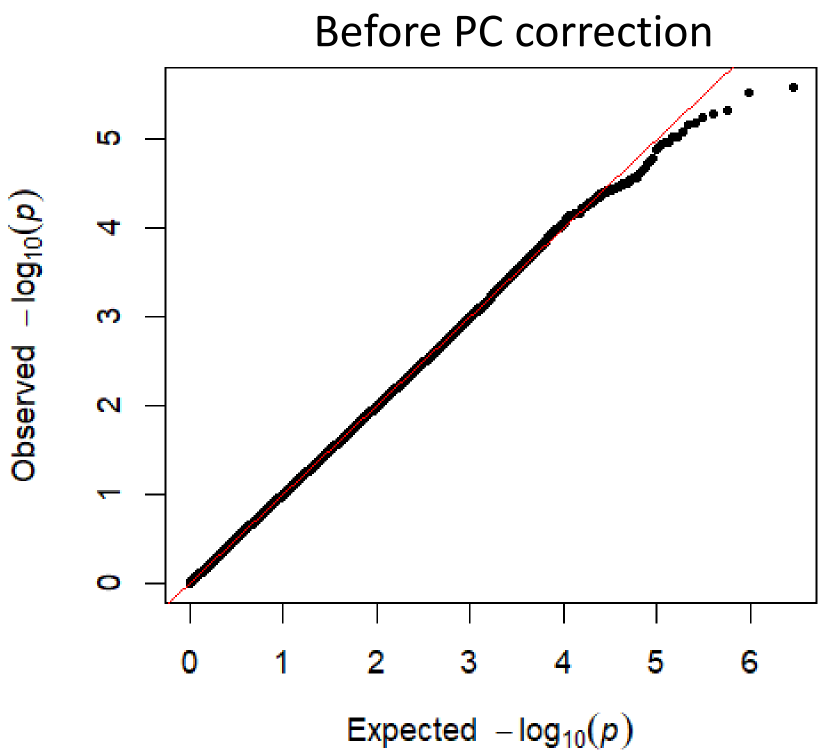

Before PC correction
Observed −log10(p)
0
1
2
3
4
5
Expected −log10(p)
0
1
2
3
4
5
6

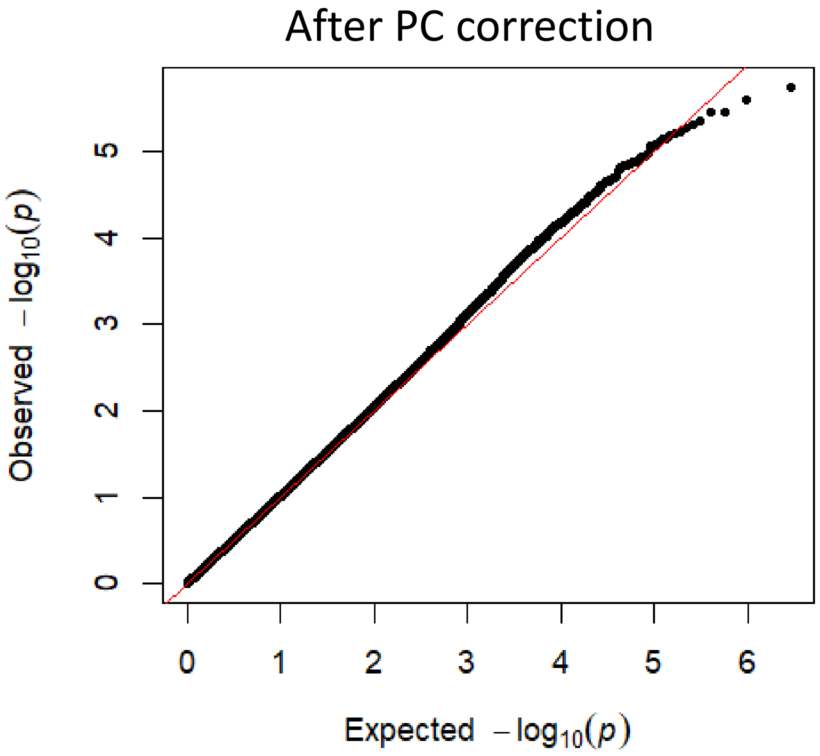

After PC correction
Observed −log10(p)
0
1
2
3
4
5
Expected −log10(p)
0
1
2
3
4
5
6

ENDRIN

Before PC correction

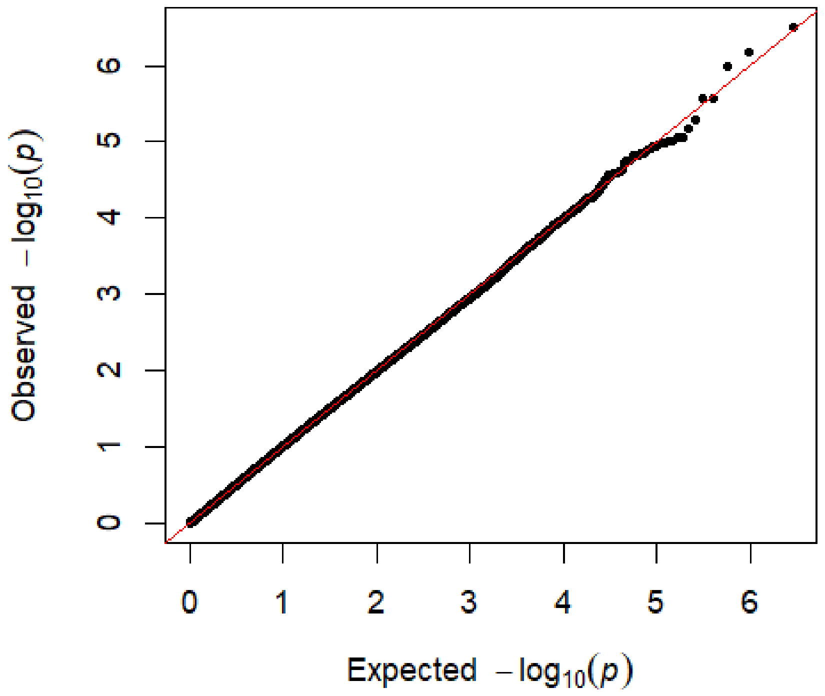

After PC correction

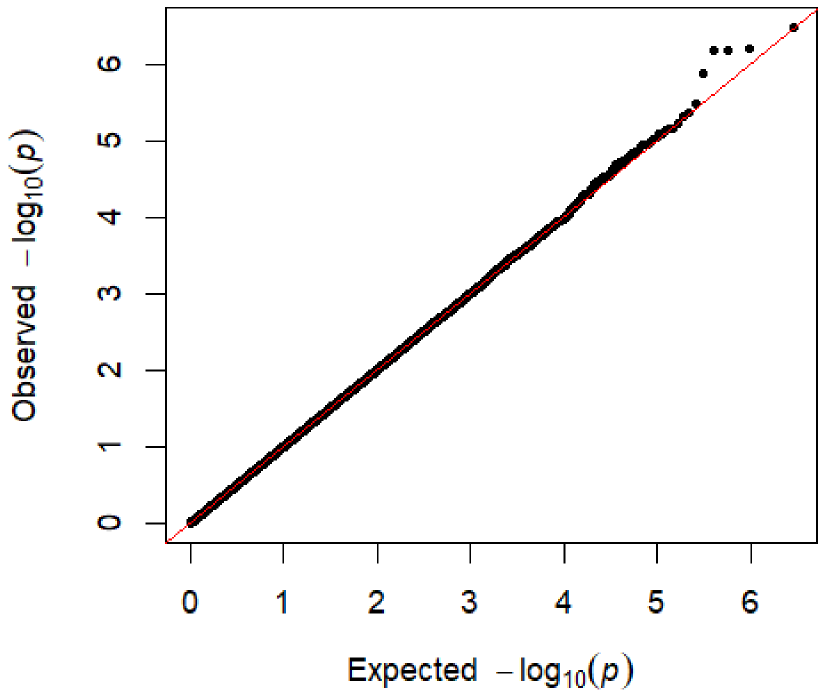

ETHION

Before PC correction

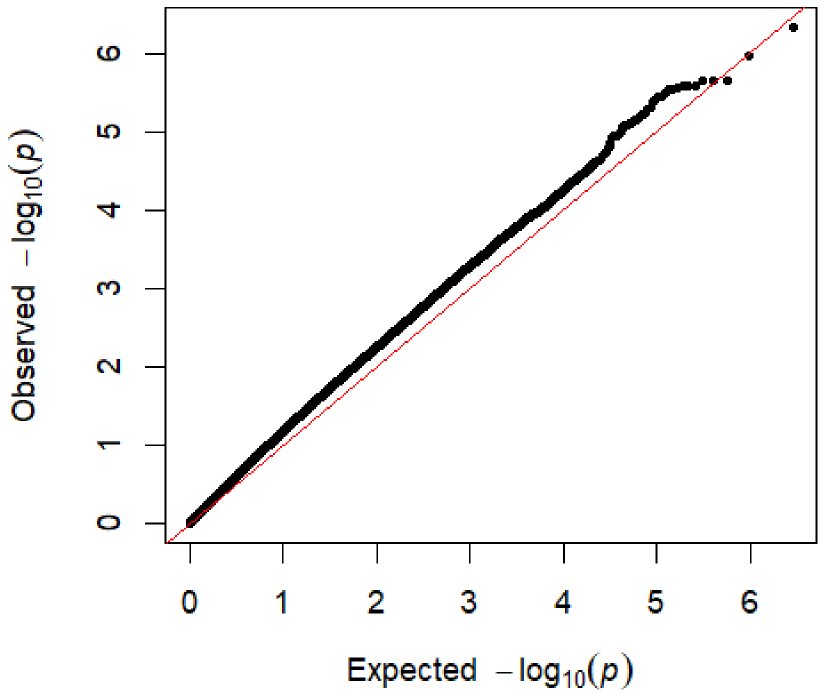

After PC correction

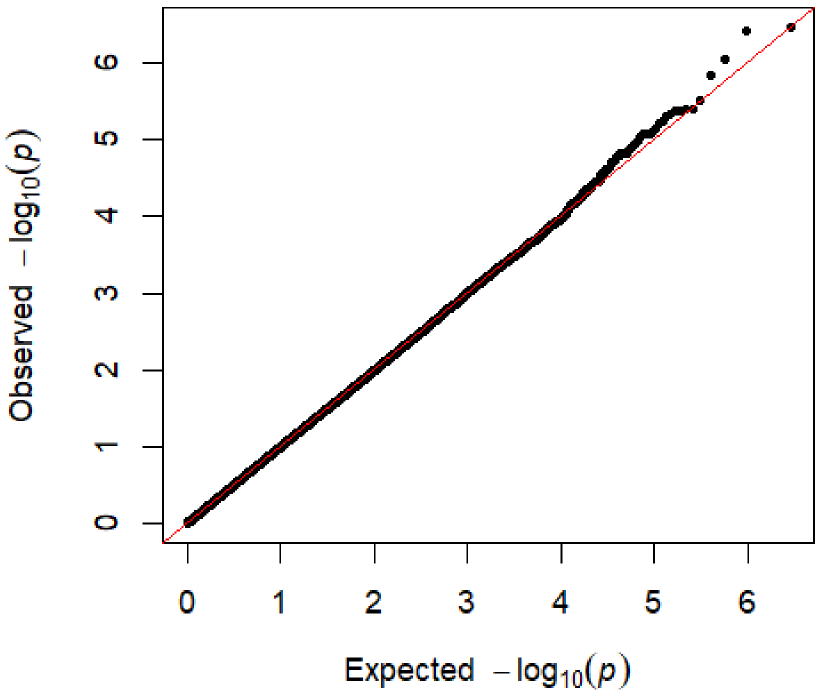

HEPTACHLOR

Before PC correction

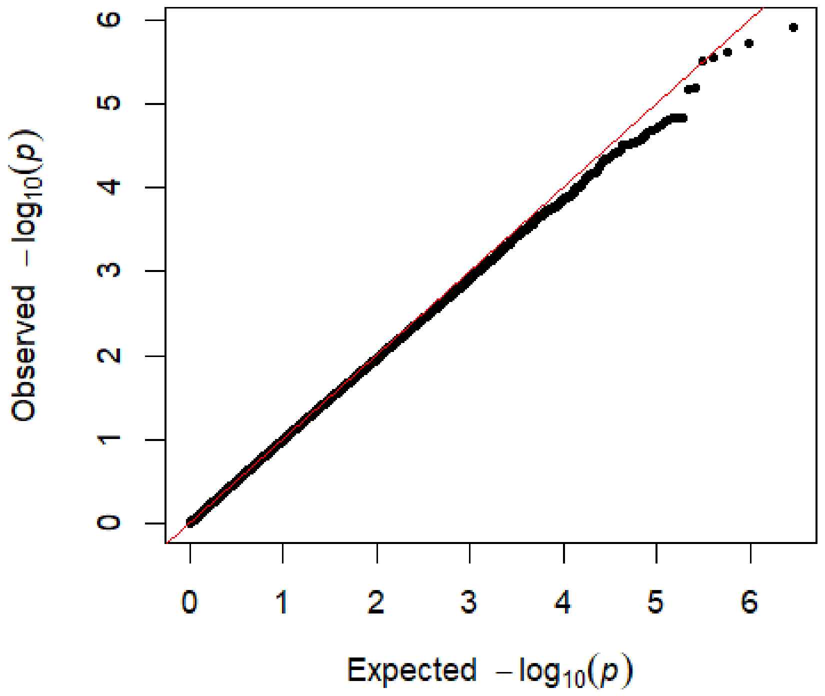

After PC correction

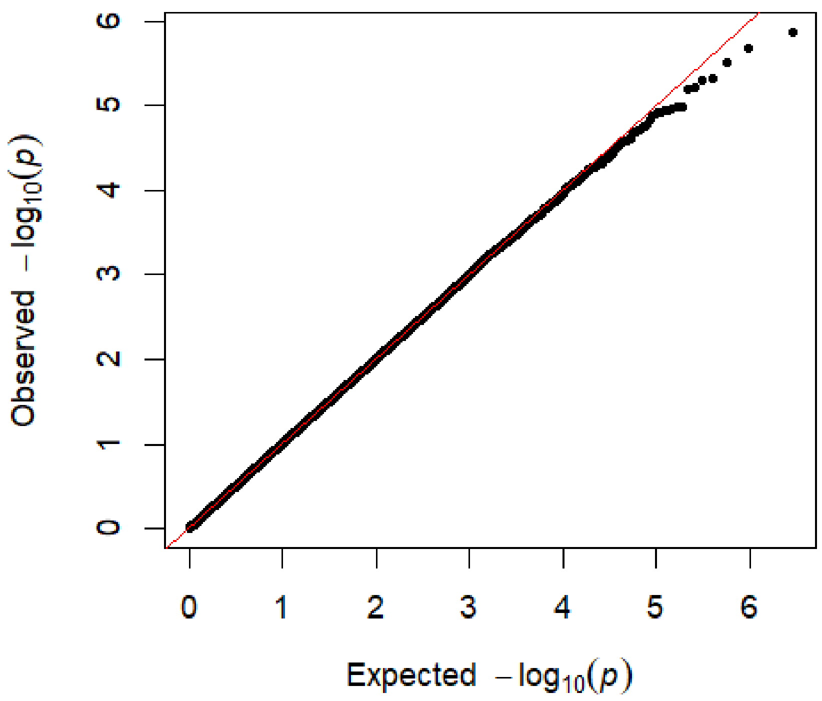

HEPTACHLOR EPOXIDE

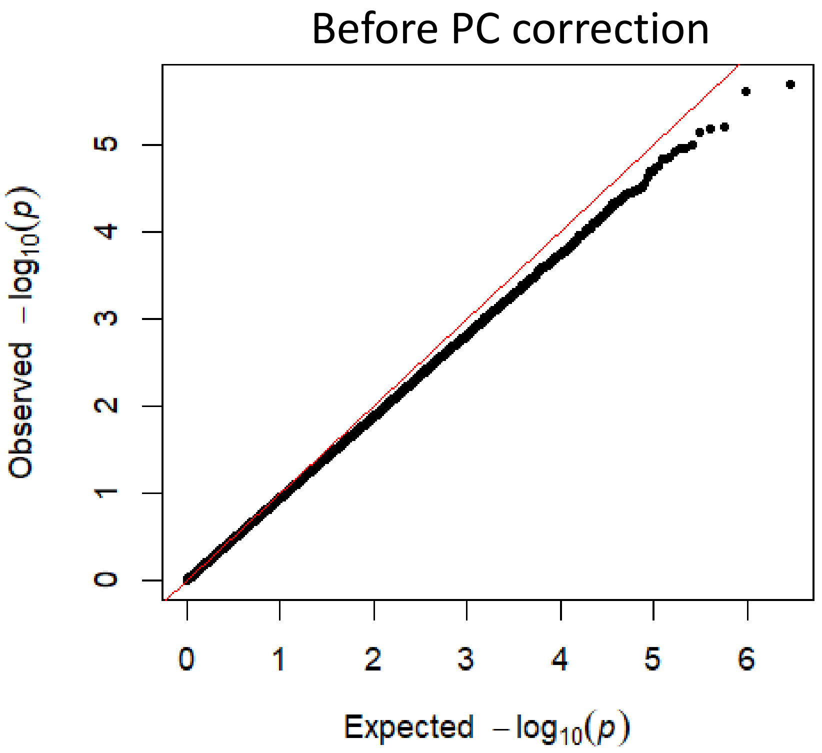

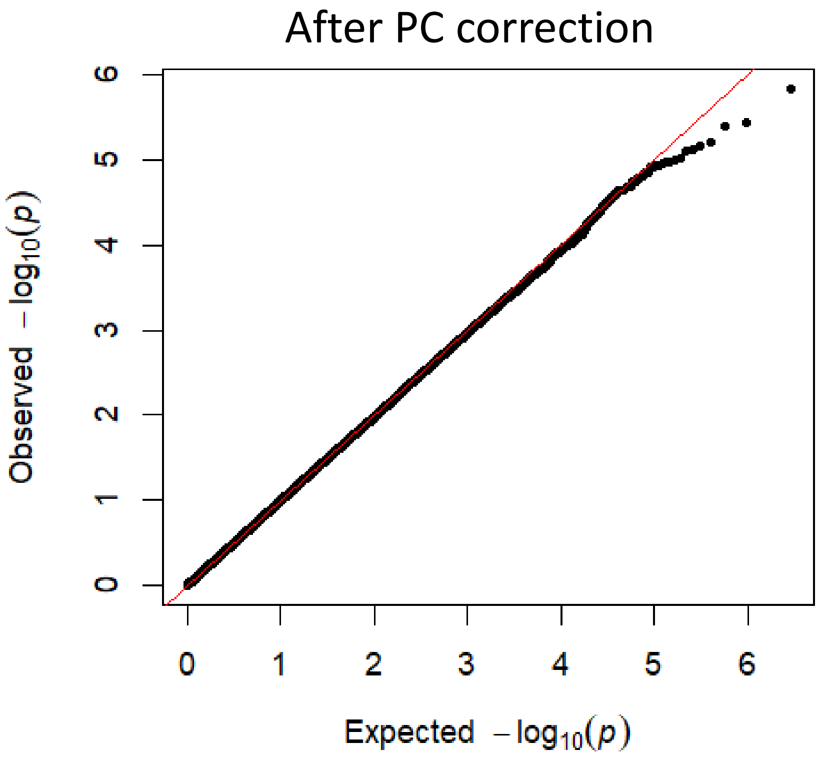

MERCURIC CHLORIDE

Before PC correction

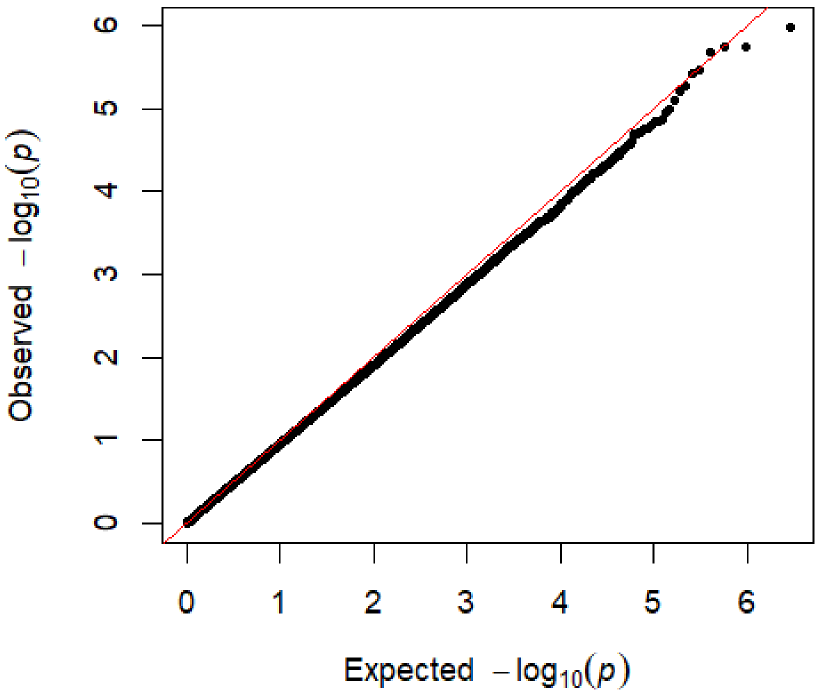

After PC correction

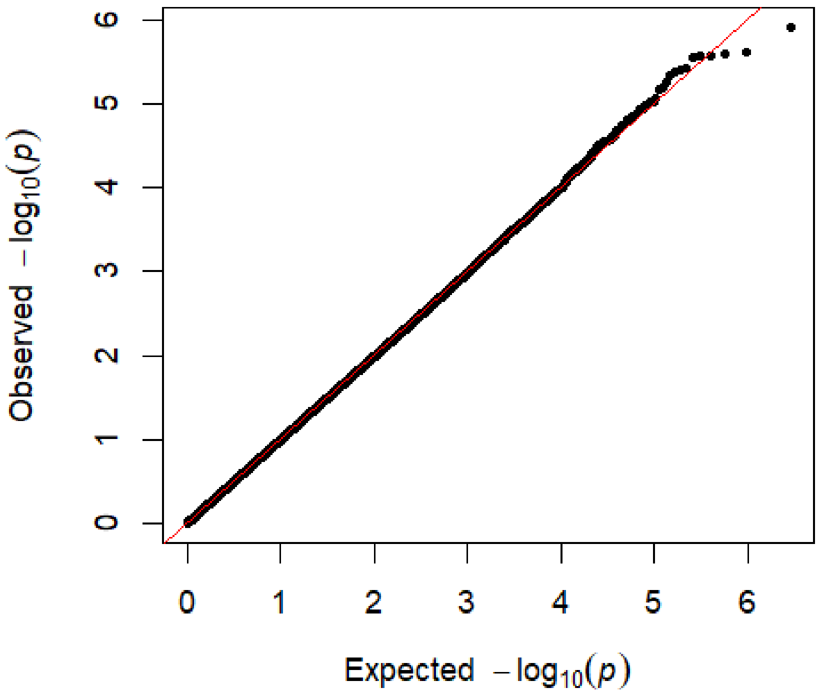

MERCURIC CHLORIDE-2

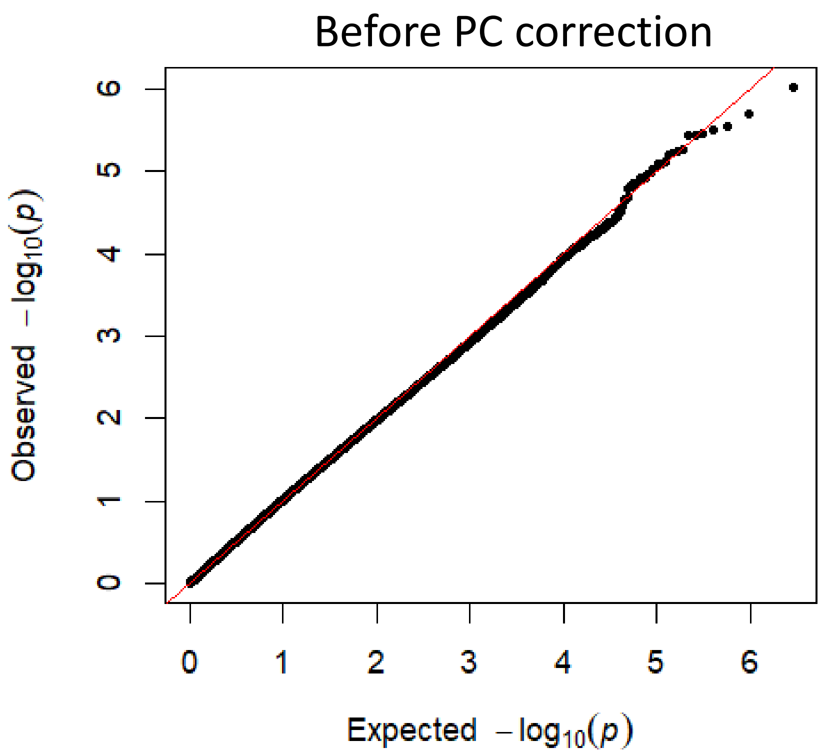

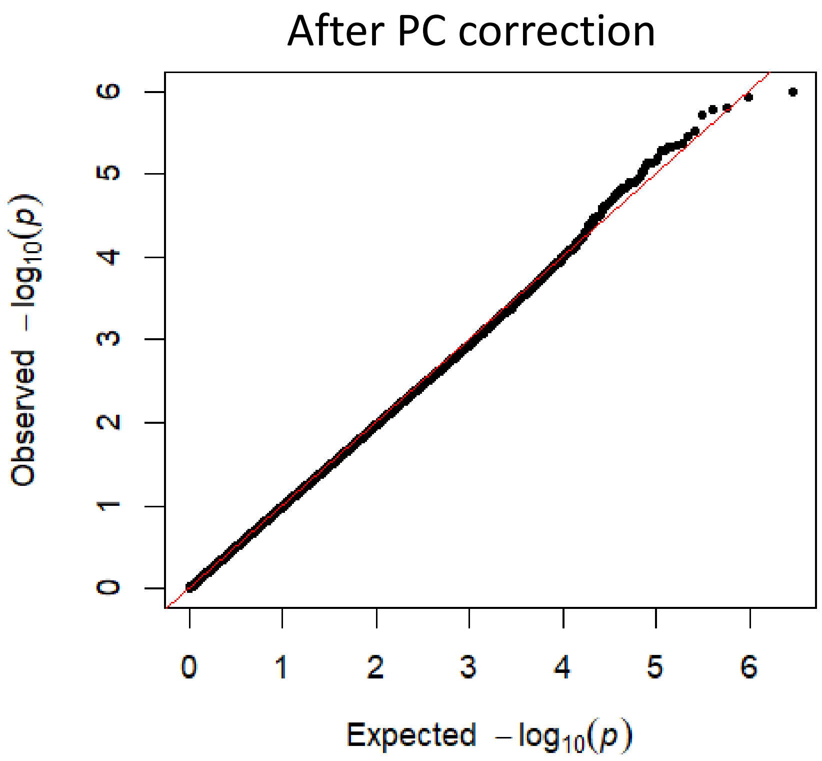

METHOXYCHLOR

Before PC correction

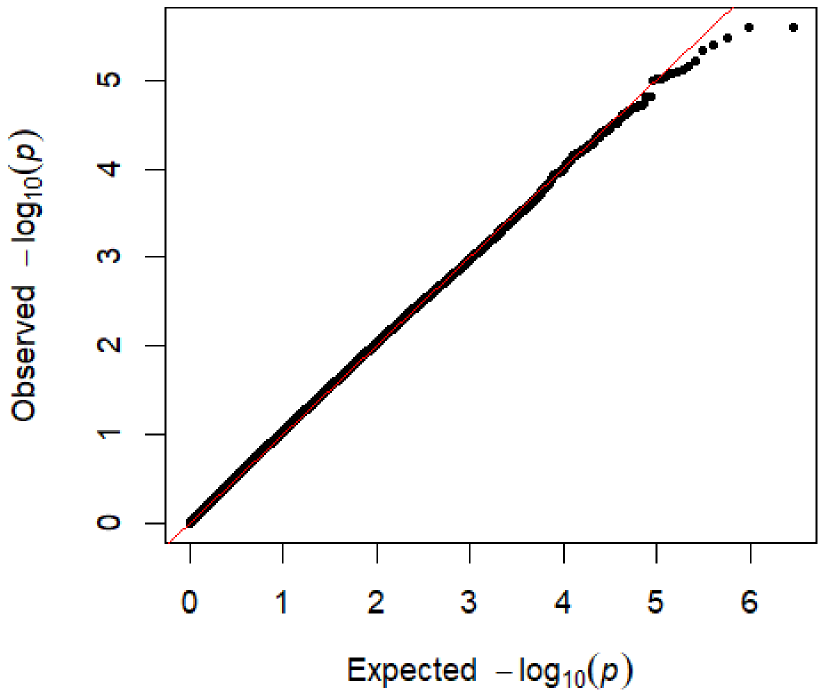

After PC correction

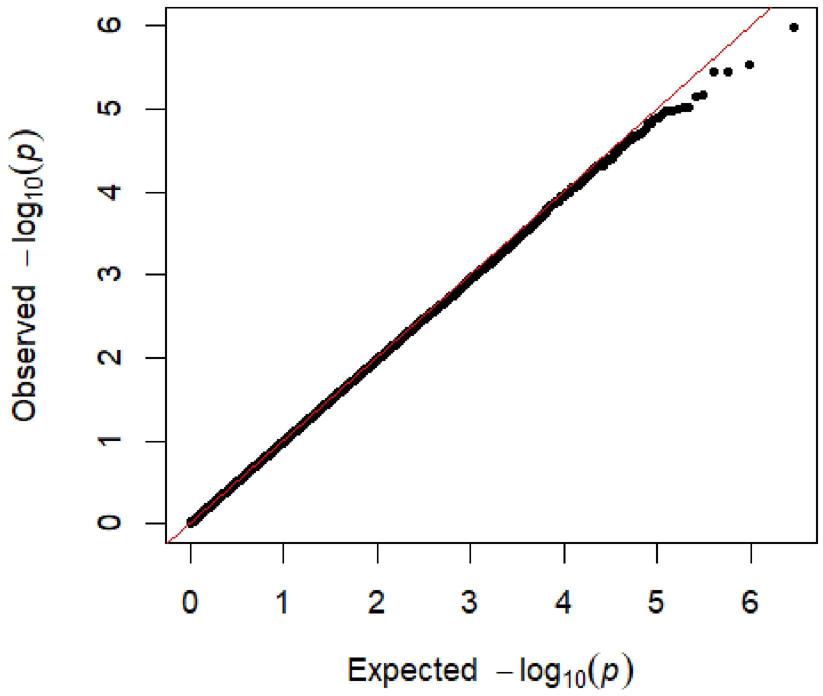

NICKEL

Before PC correction

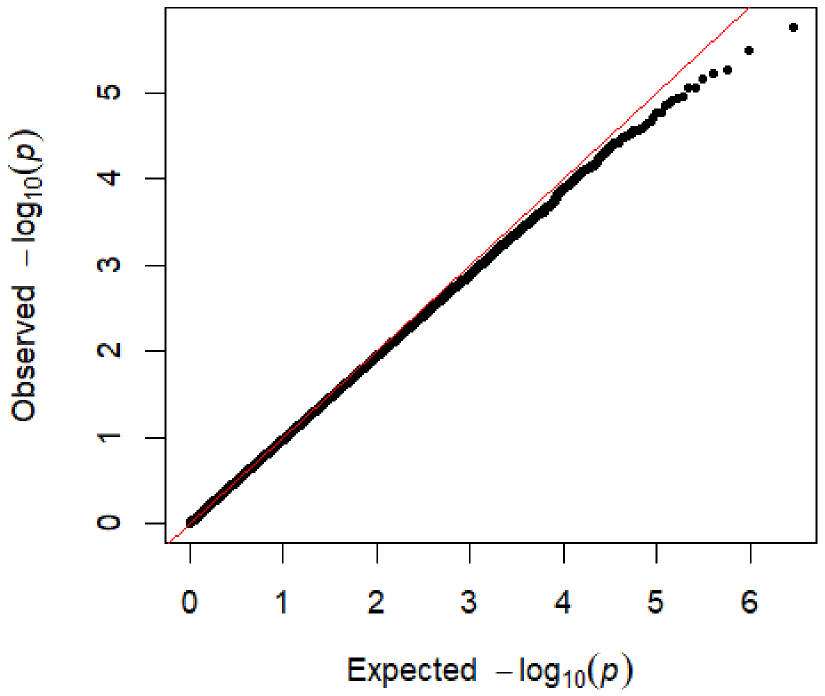

After PC correction

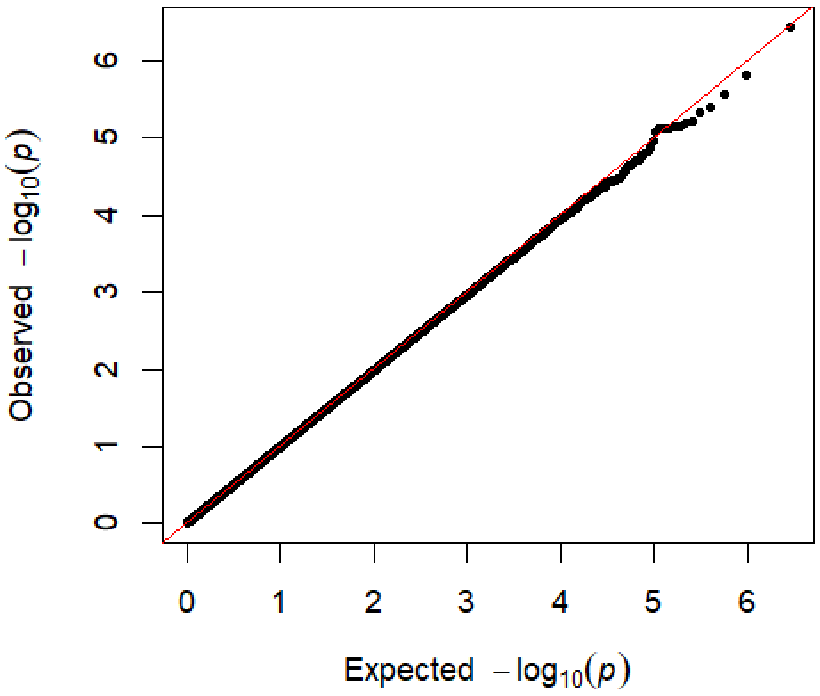

PARATHION

Before PC correction

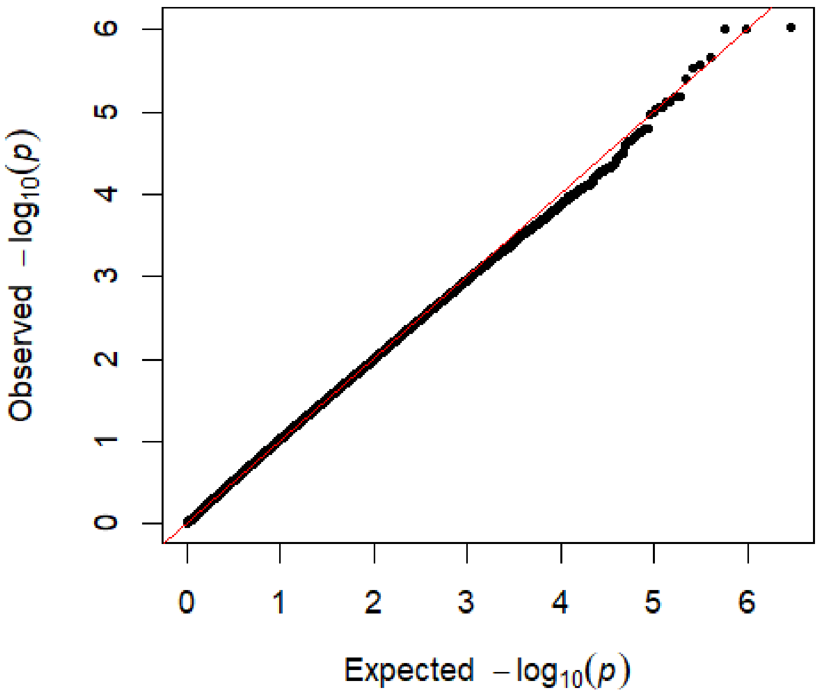

After PC correction

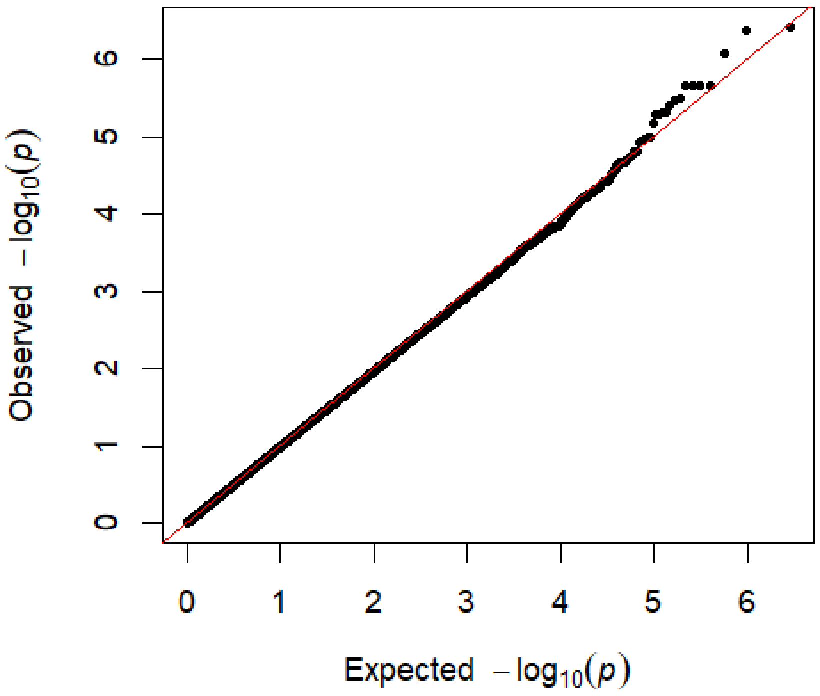

PENTACHLOROPHENOL

Before PC correction

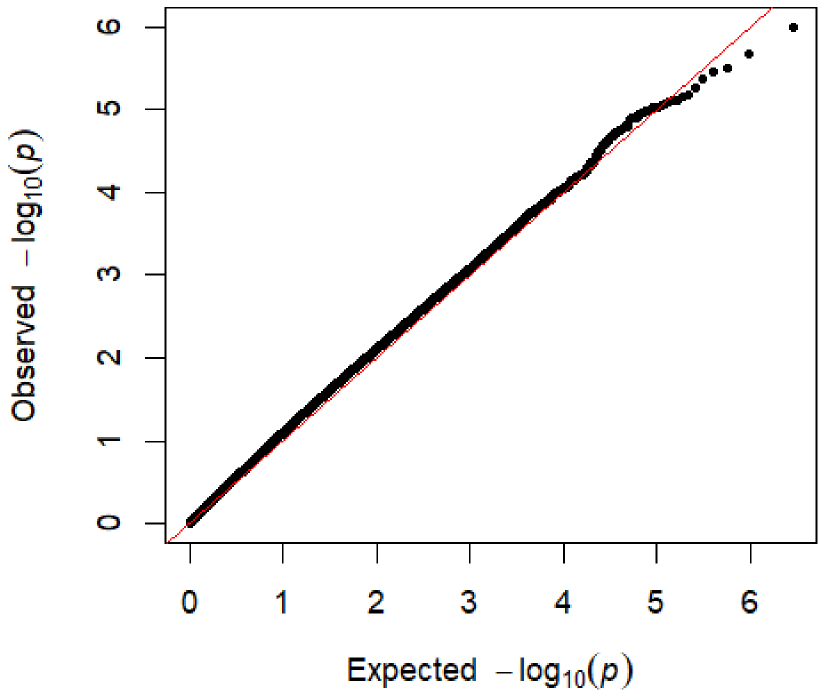

After PC correction

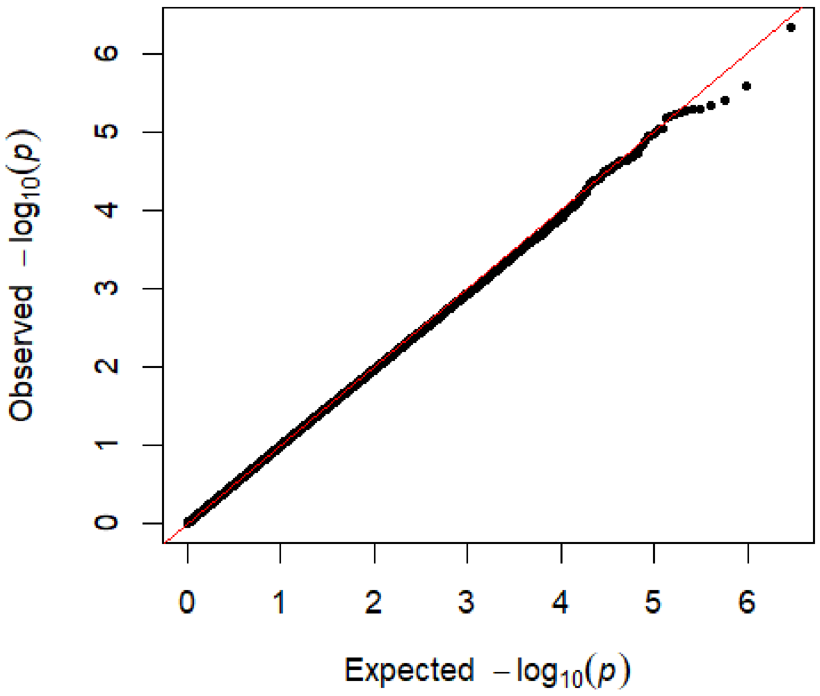

Potassium Chromate

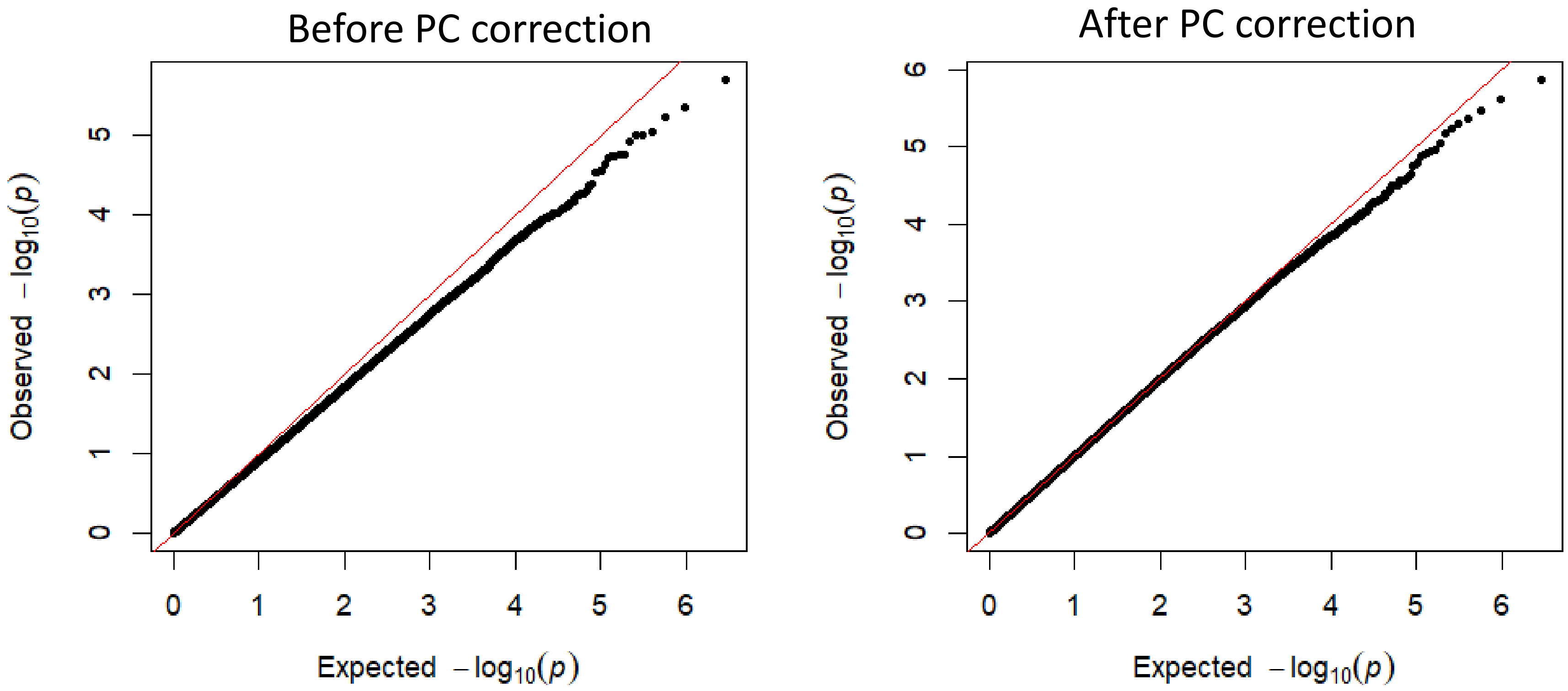

AC50-Low

Before PC correction

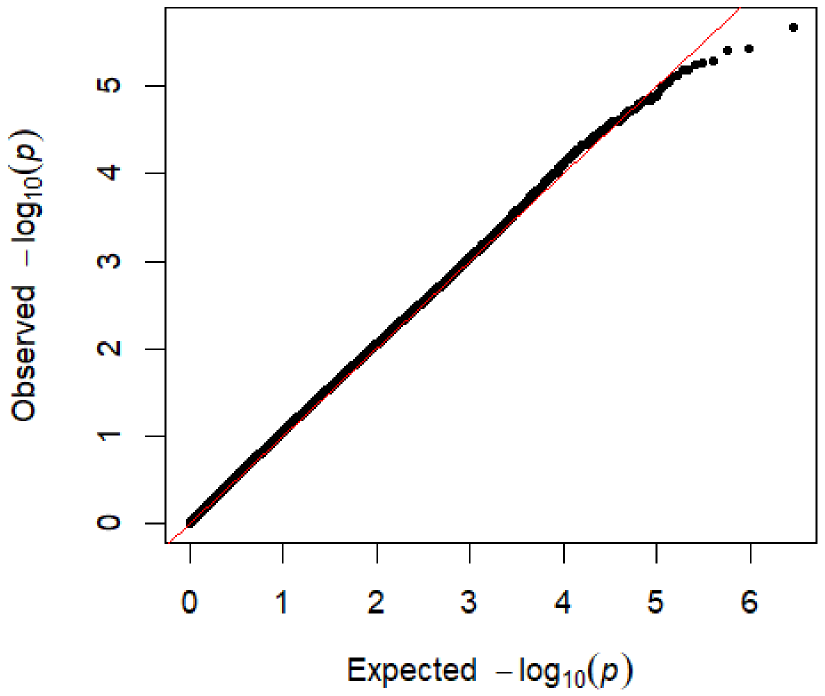

After PC correction

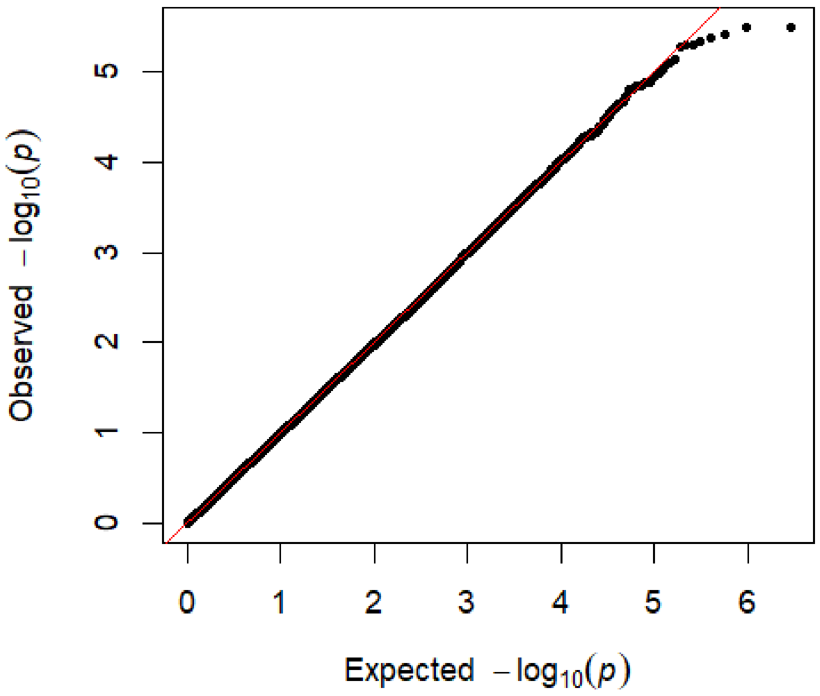

AC50-High

Before PC correction

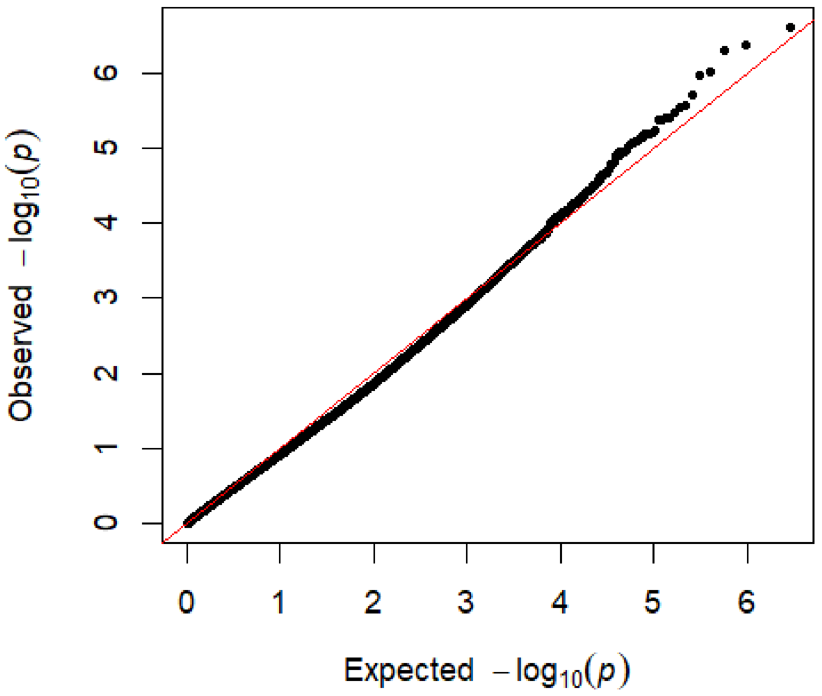

After PC correction

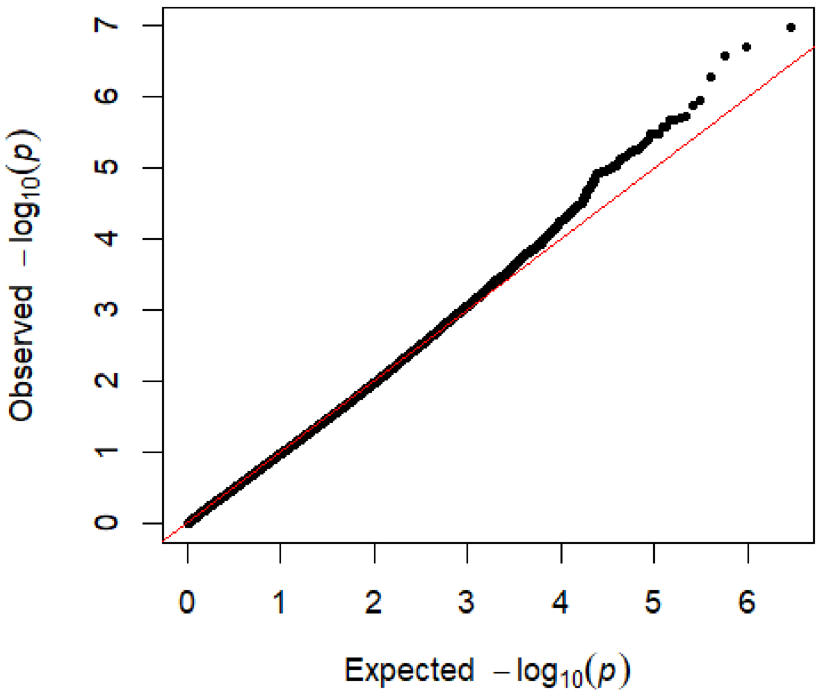

POD-Low

Before PC correction

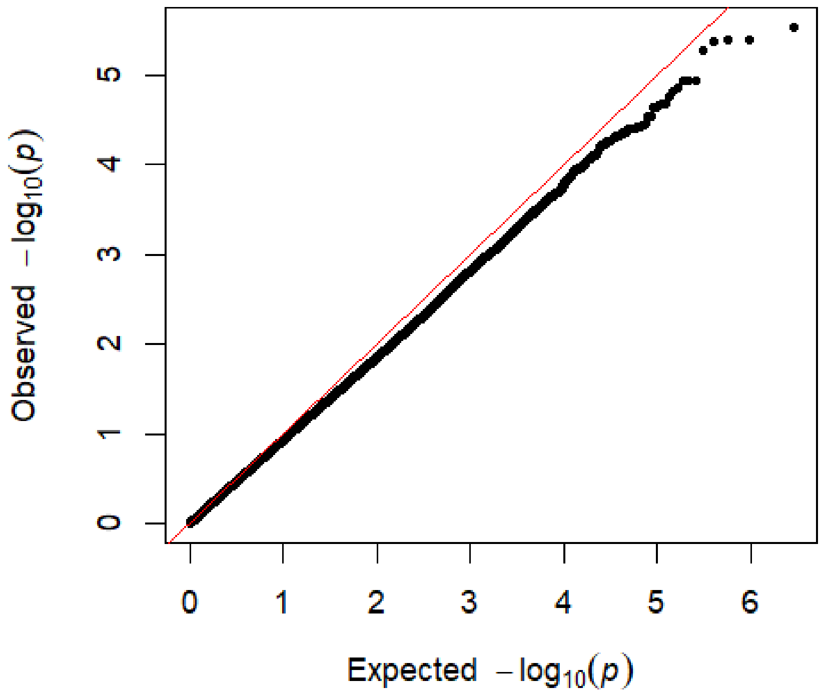

After PC correction

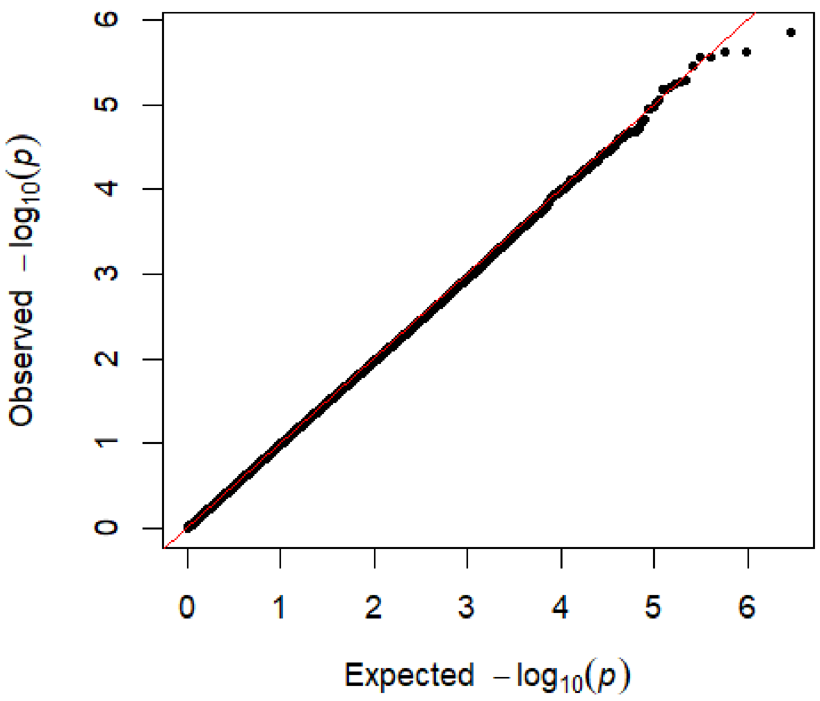

POD-High

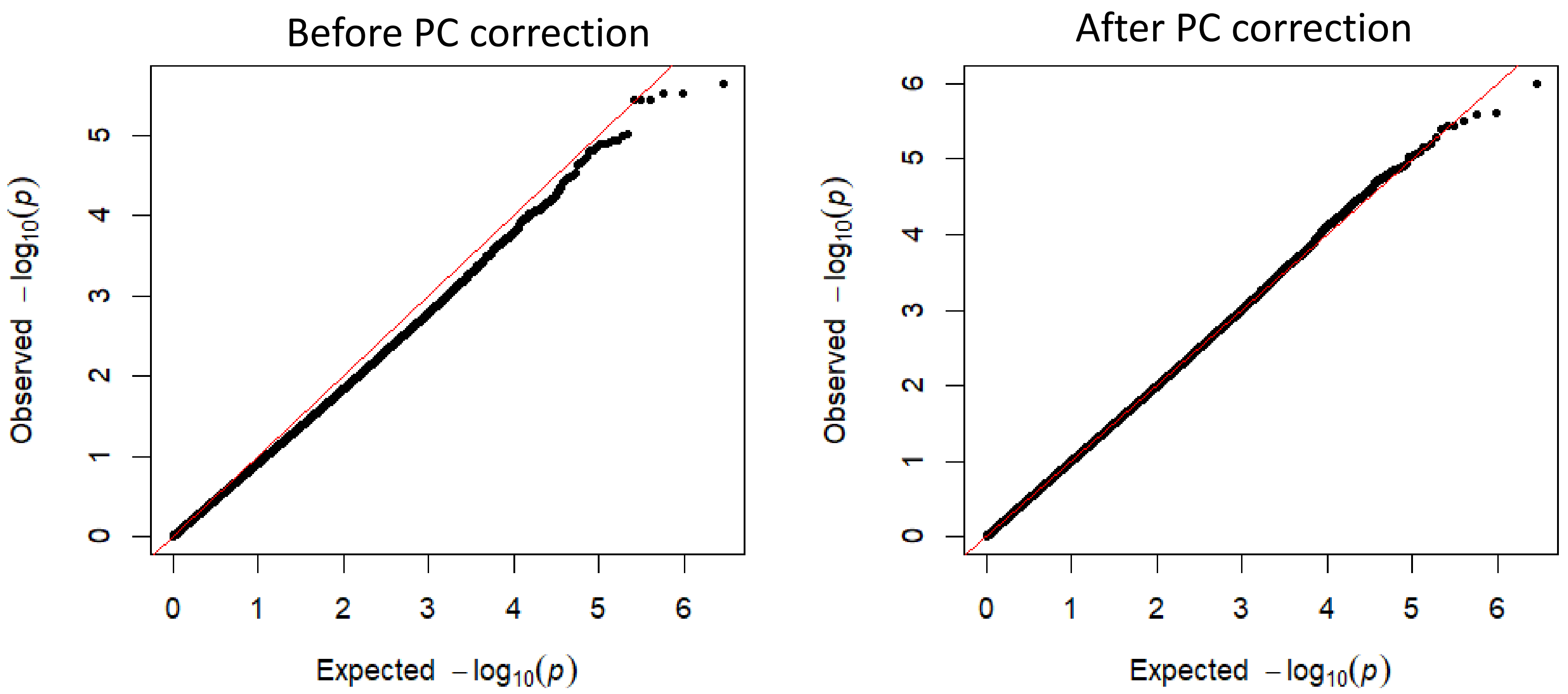
Before PC correction
After PC correction
Observed $-\log_{10}(p)$
Expected $-\log_{10}(p)$
0
1
2
3
4
5
6

Expo-Low

Before PC correction

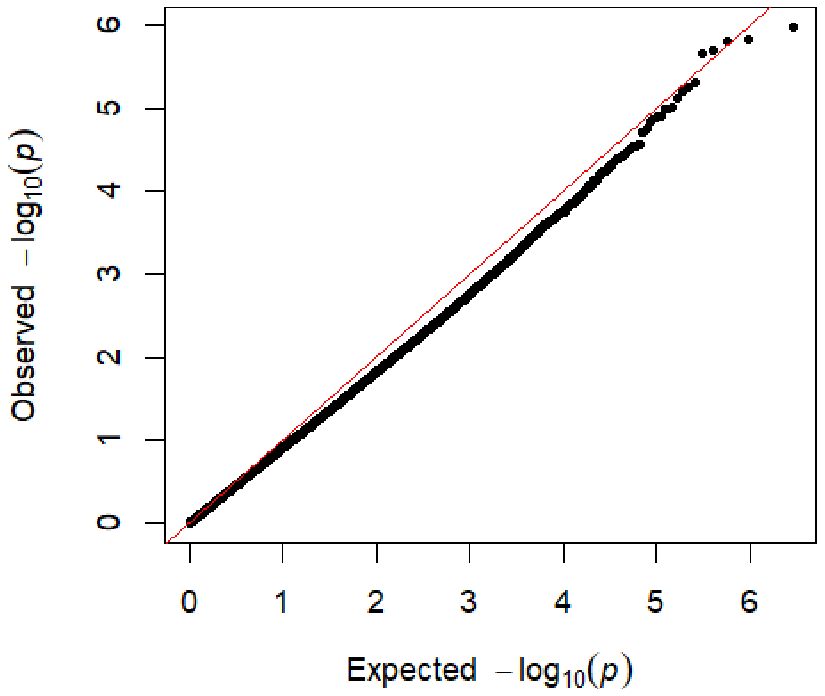

After PC correction

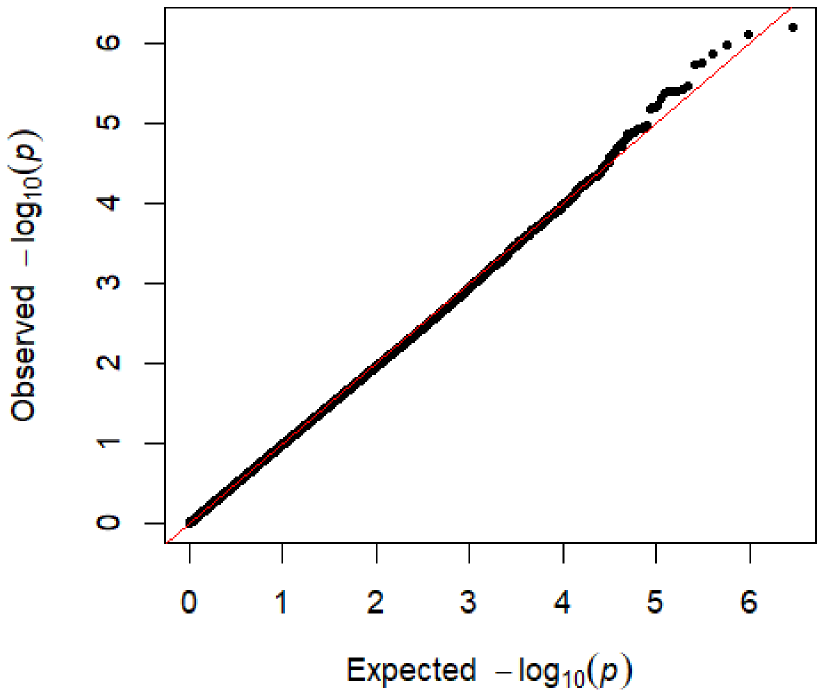

Expo-High

Before PC correction

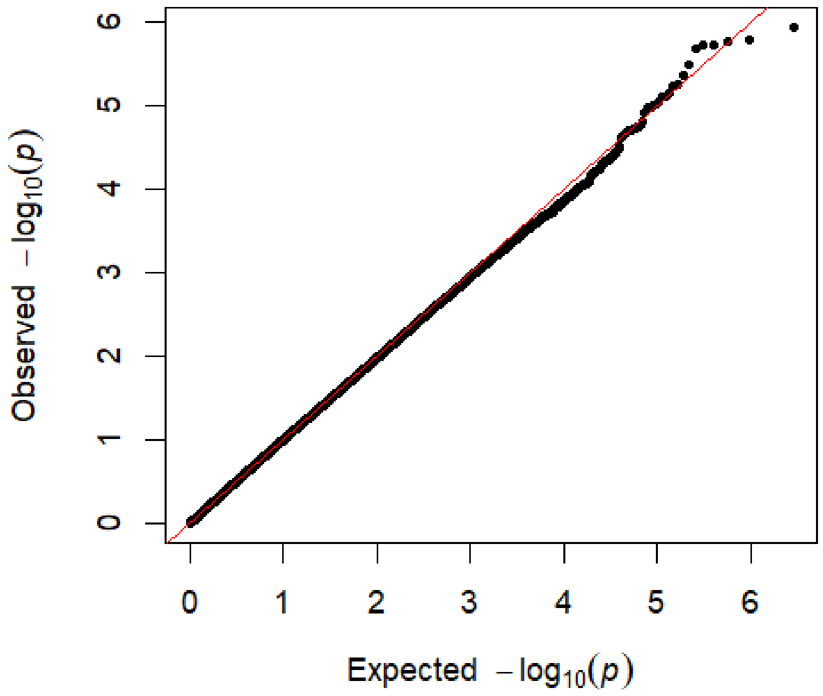

After PC correction

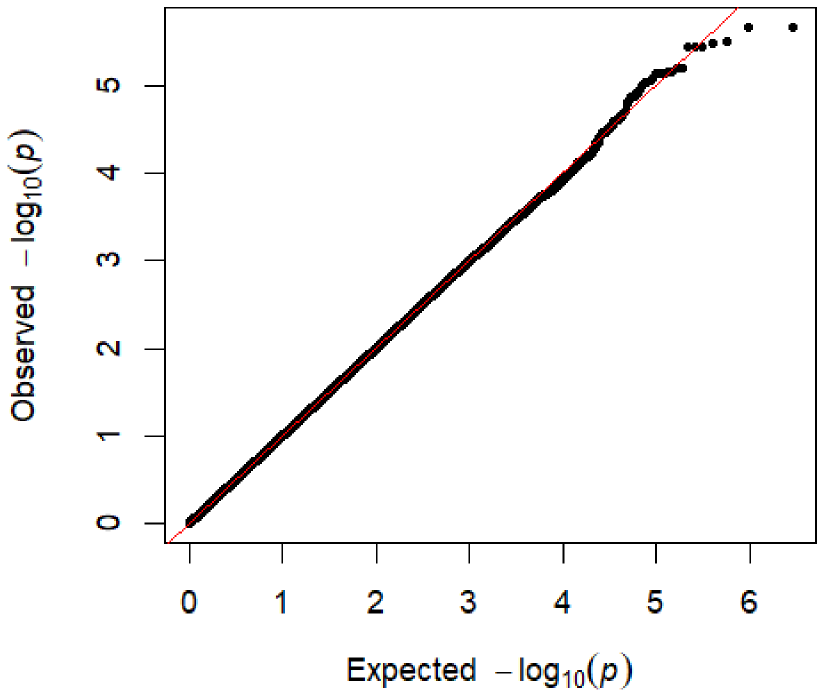

RfD-Low

Before PC correction

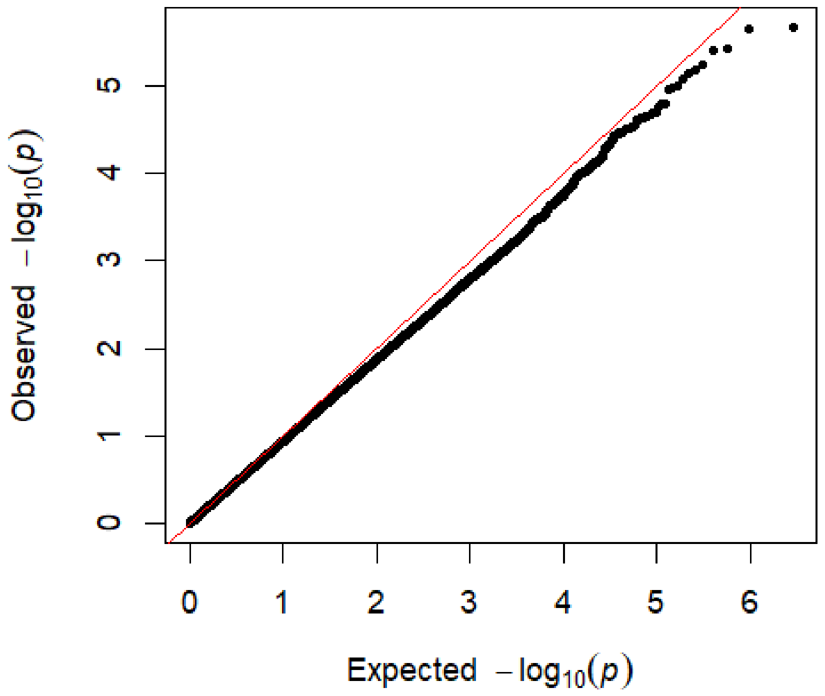

After PC correction

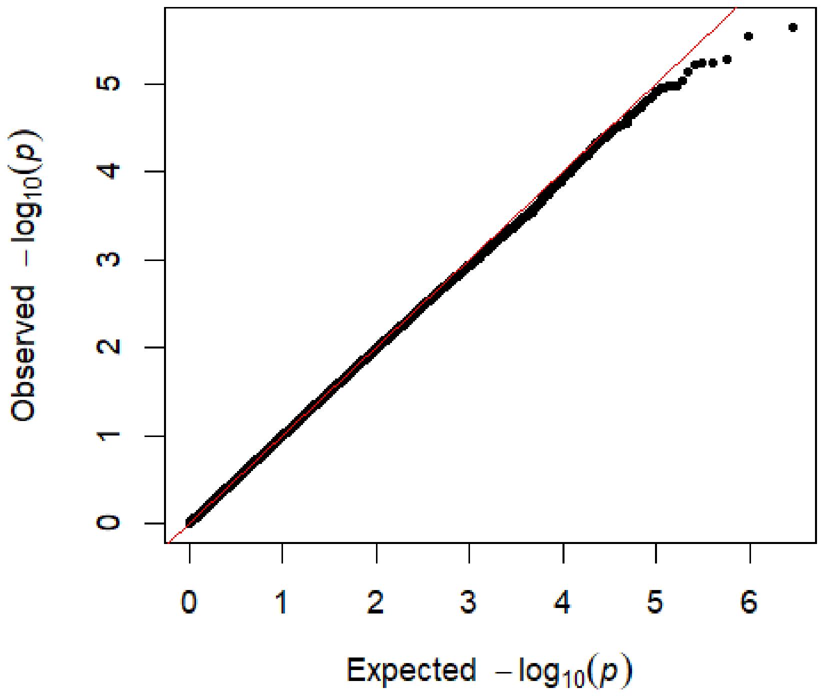

RfD-High

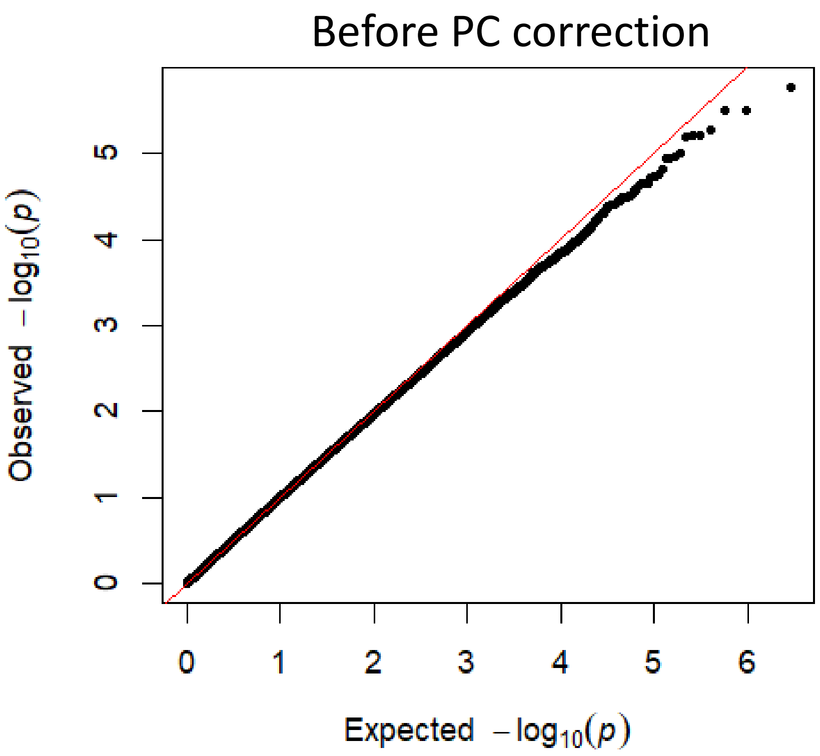
Before PC correction
Observed $-\log_{10}(p)$
Expected $-\log_{10}(p)$
0
1
2
3
4
5
6

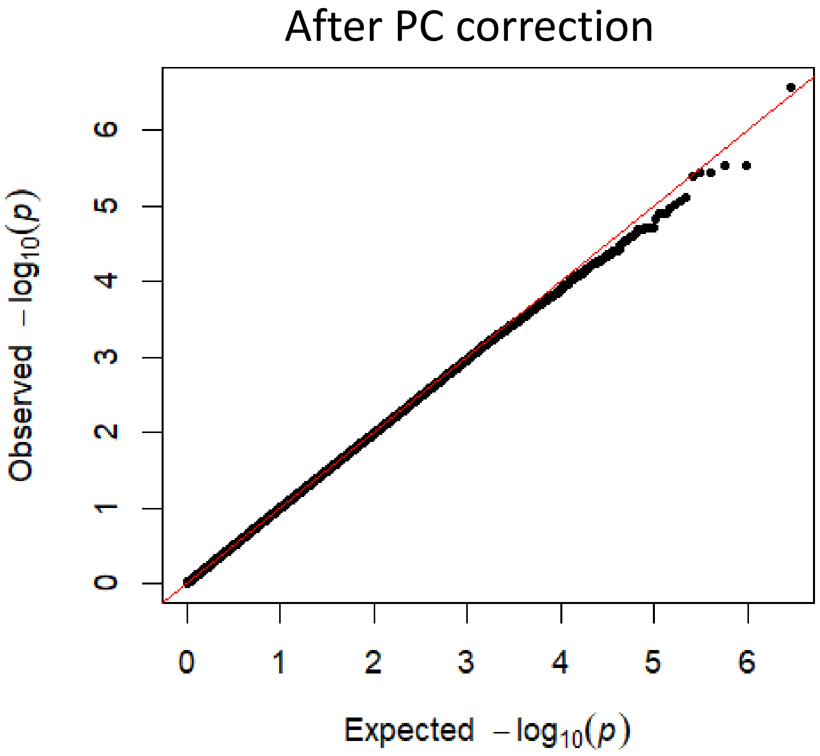
After PC correction
Observed $-\log_{10}(p)$
Expected $-\log_{10}(p)$
0
1
2
3
4
5
6
