# Supplementary material for: A Population-Based Human In Vitro Approach to Quantify Inter-Individual Variability in Responses to Chemical Mixtures
Source: Toxics. 2022 Aug 1;10(8):441. doi: 10.3390/toxics10080441 (PMC9413237; doi:10.3390/toxics10080441)
Supplement: Supplementary file 1 [file toxics-10-00441-s001.zip › Figure S3 Manhattan Plot with Mixed Model Analyses.pdf]

2,4,5-TRICHLOROPHENOL

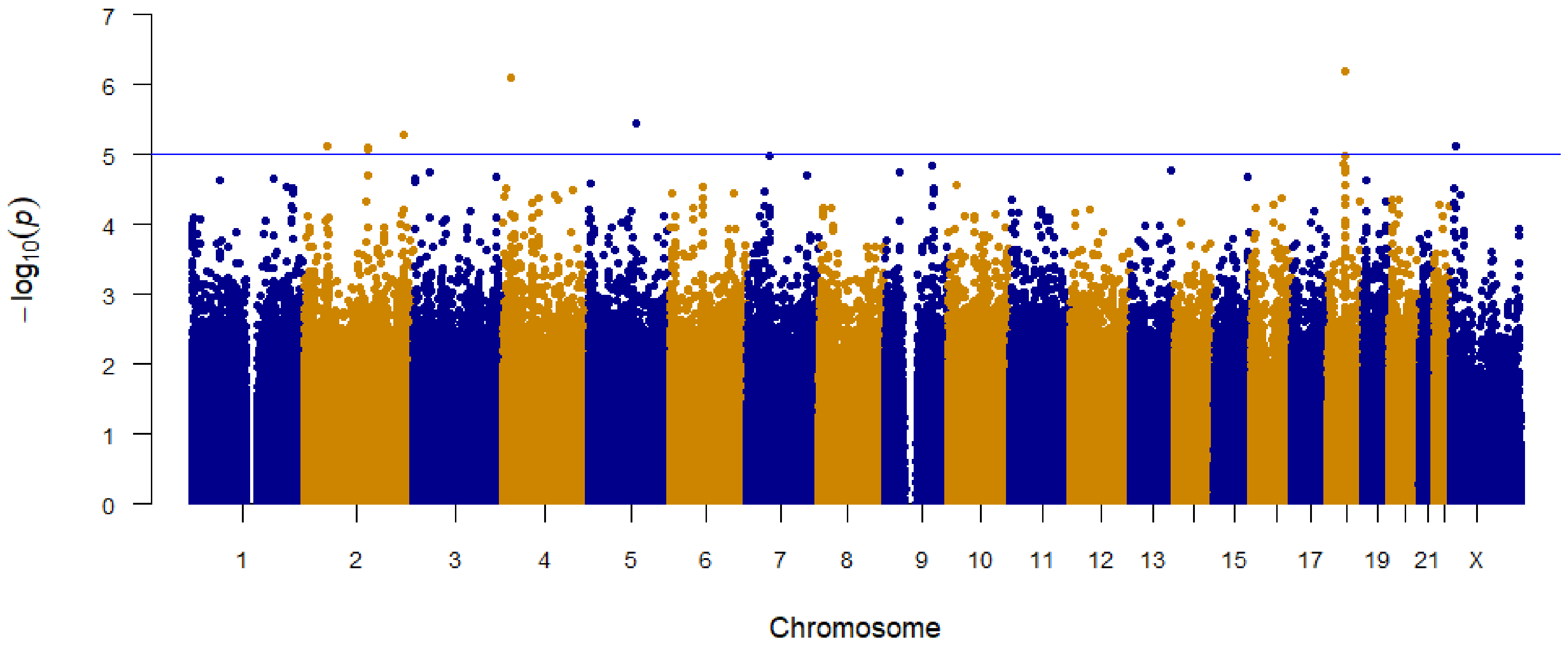

lambda=1.09

2,4,5-TRICHLOROPHENOL-2

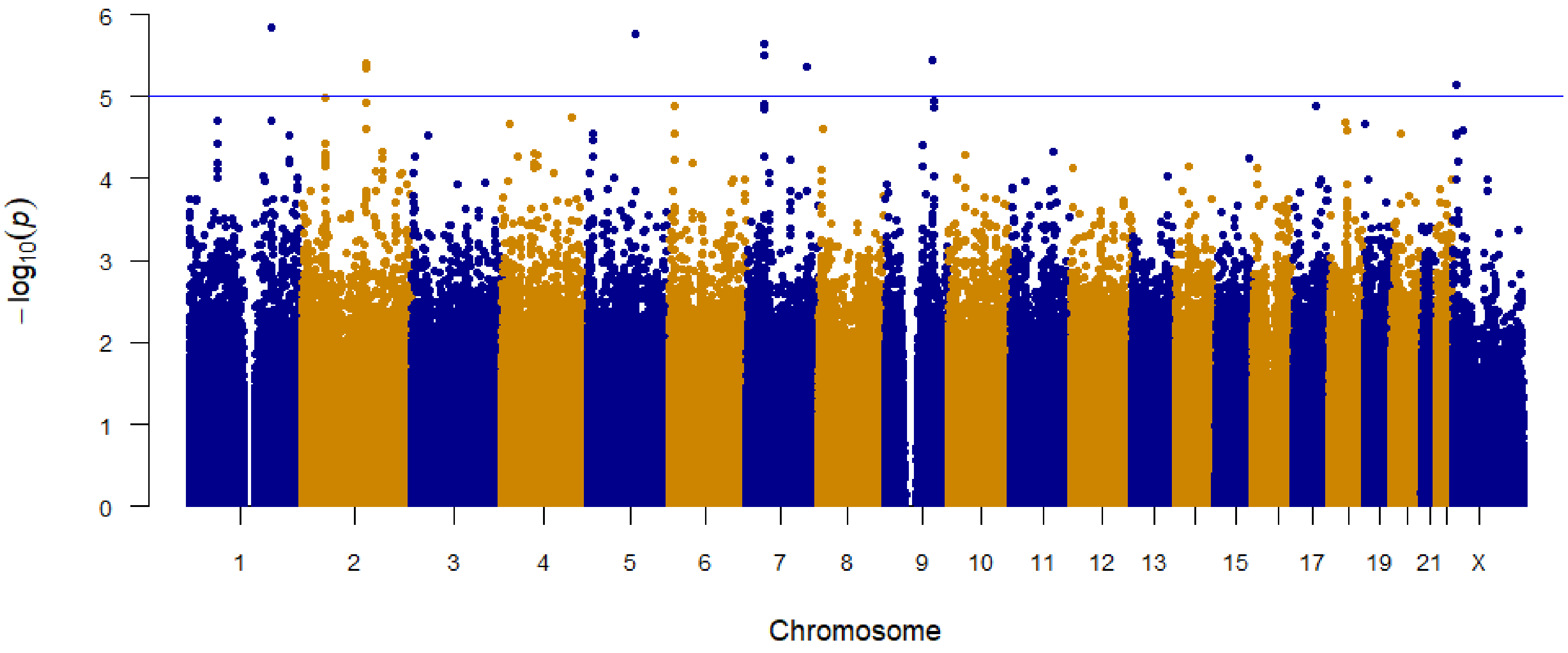

lambda=1.02

4,6-DINITRO-O-CRESOL

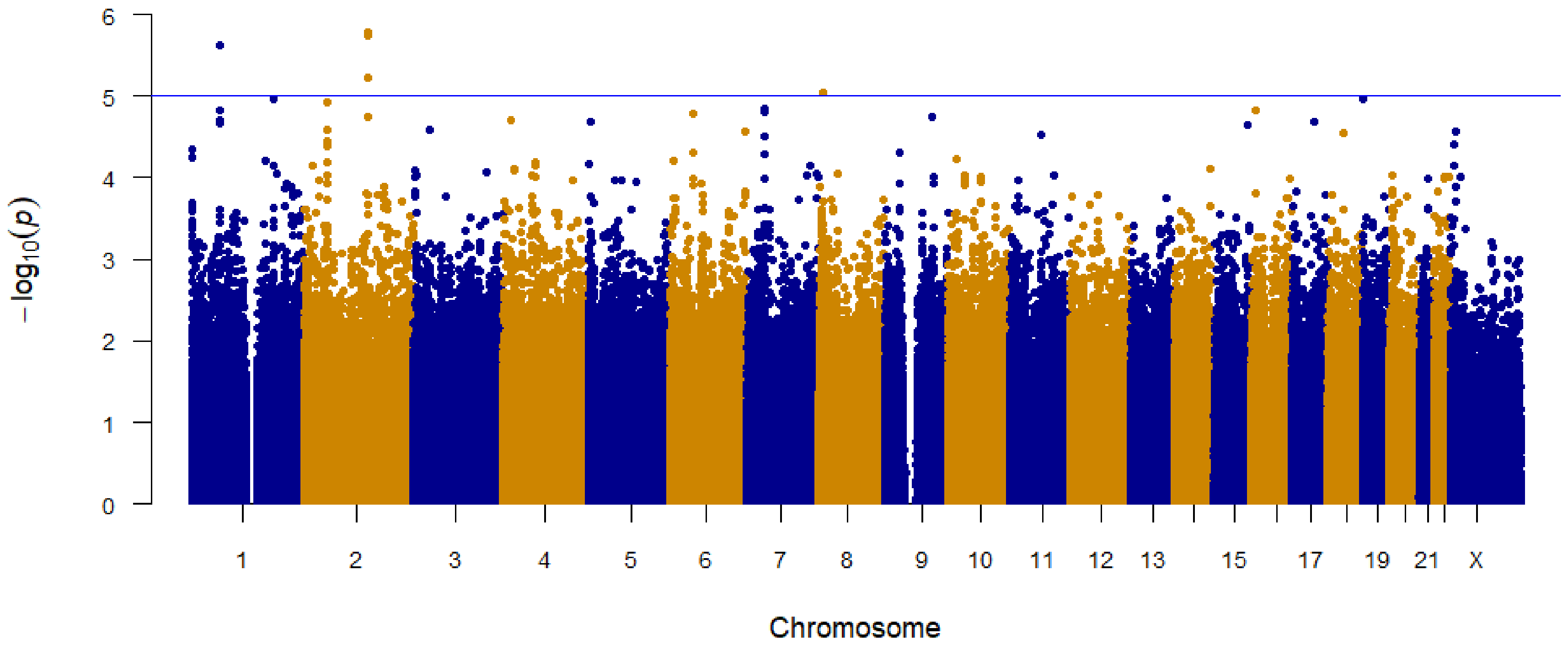

lambda=1.00

ALDRIN

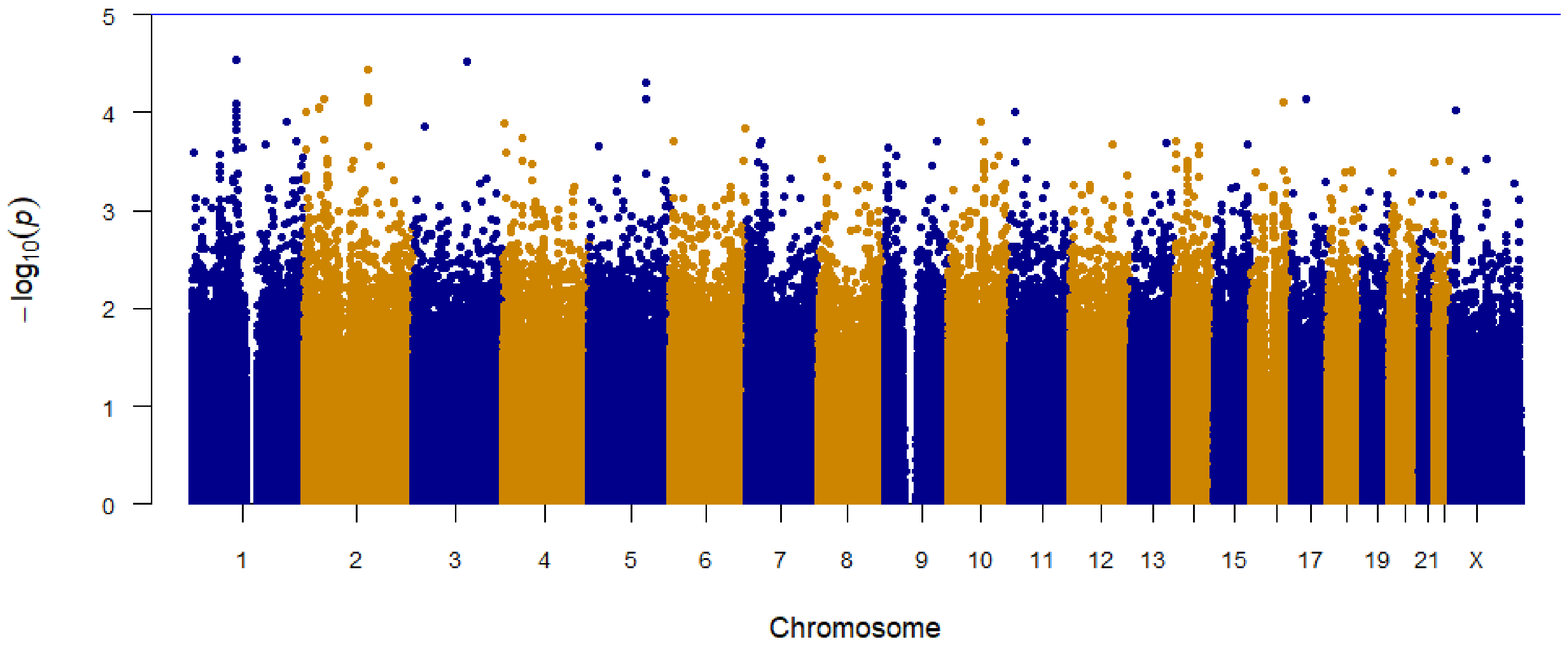

lambda=0.84

AZINPHOS-METHYL

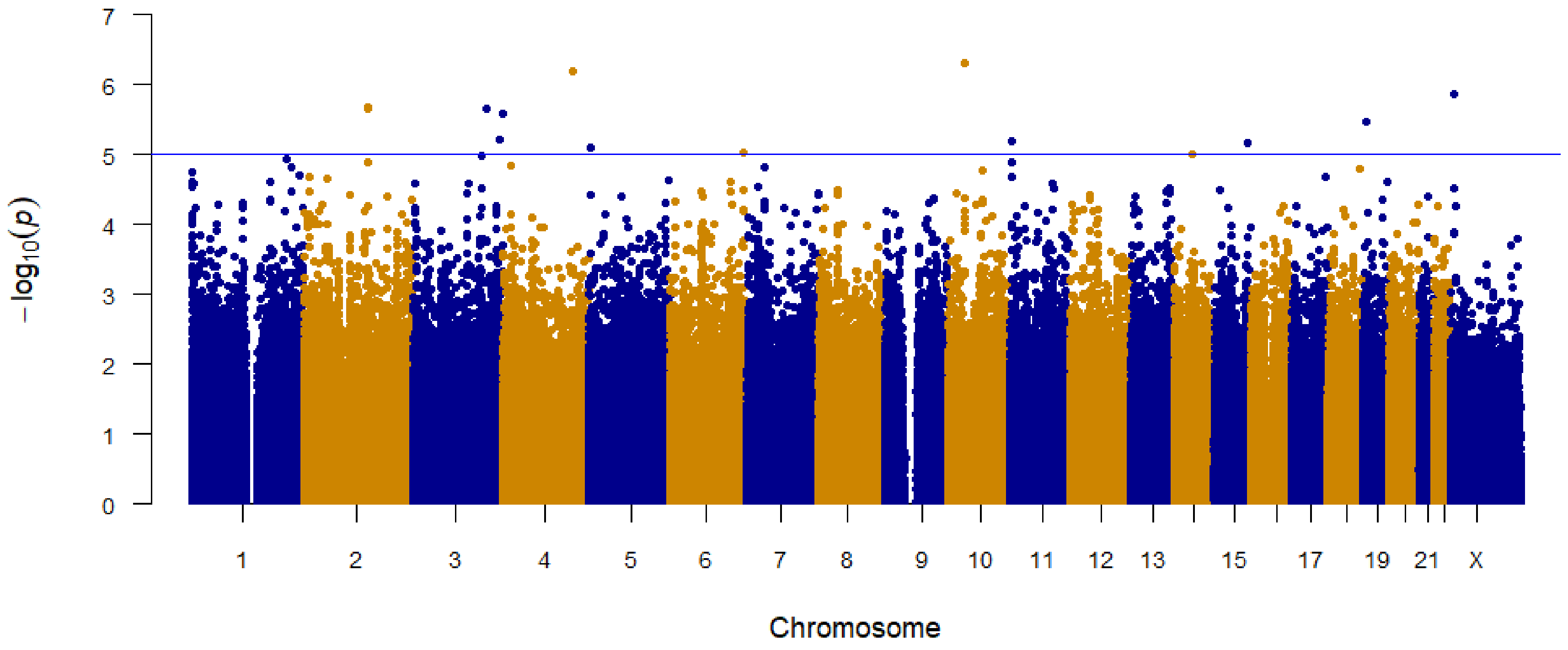

lambda=1.08

CADMIUM(Chloride)

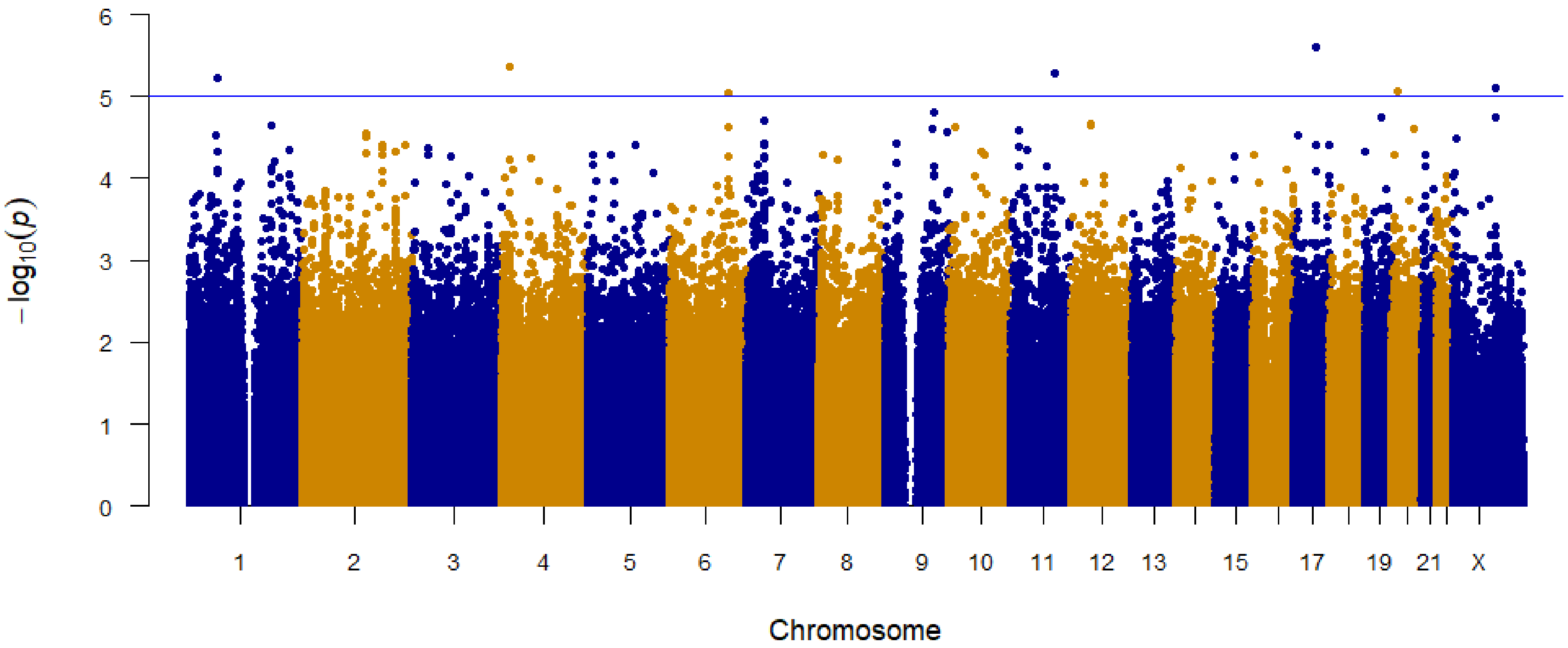

lambda=1.02

CHLORPYRIFOS

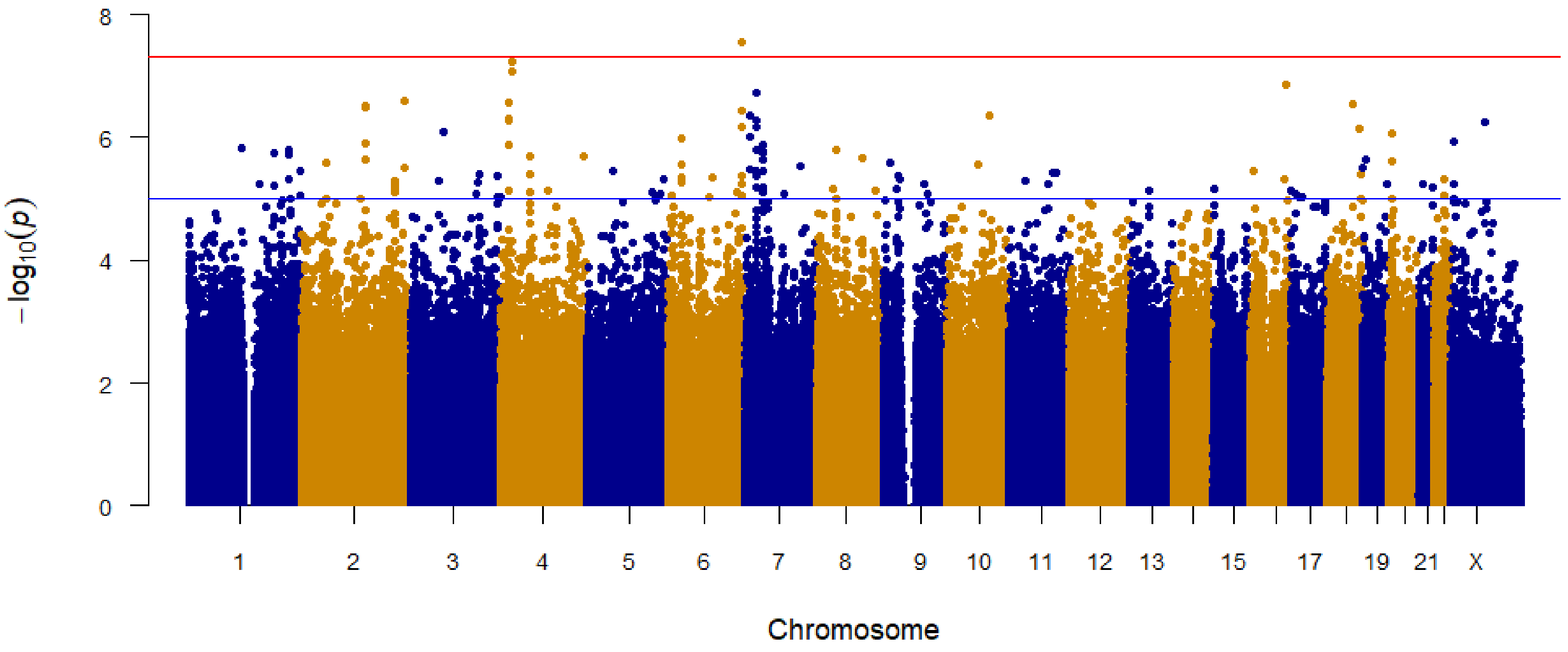

lambda=1.32

COBALT

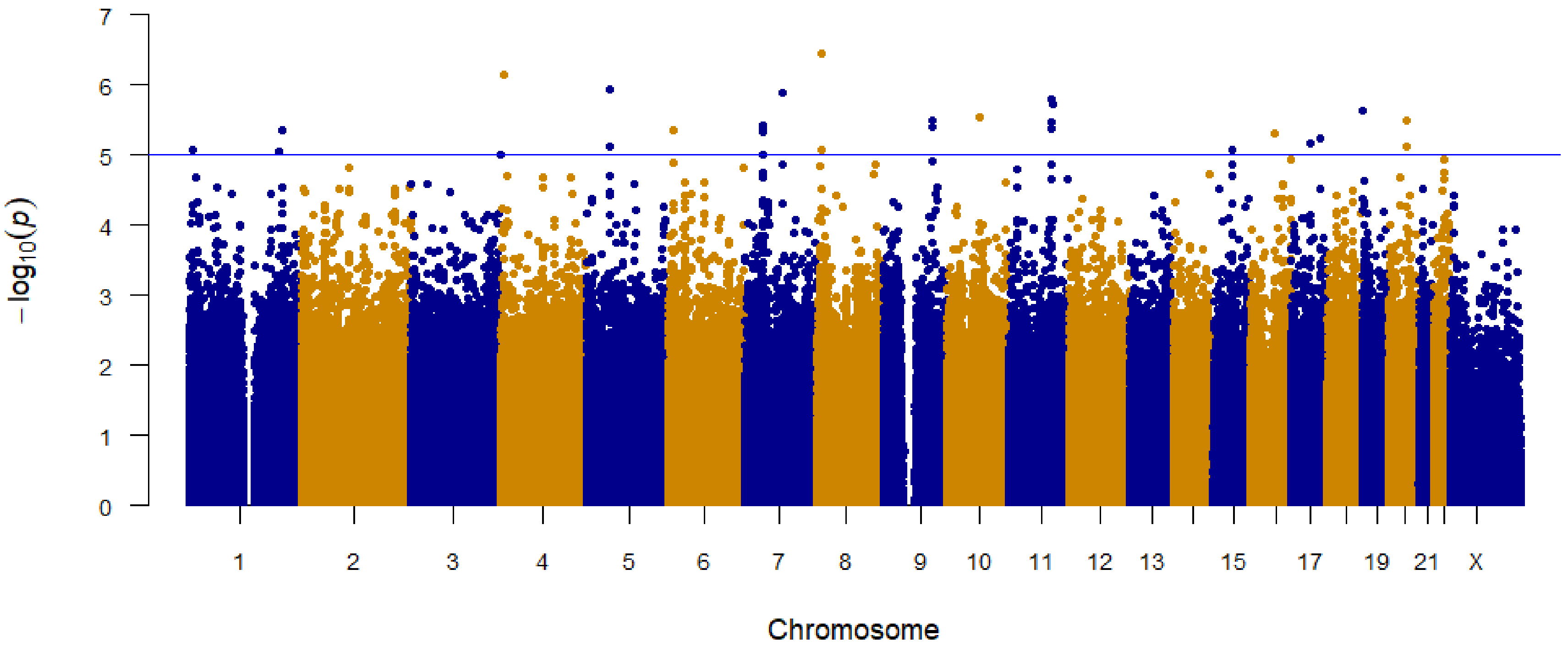

lambda=1.12

DDD, P,P'-

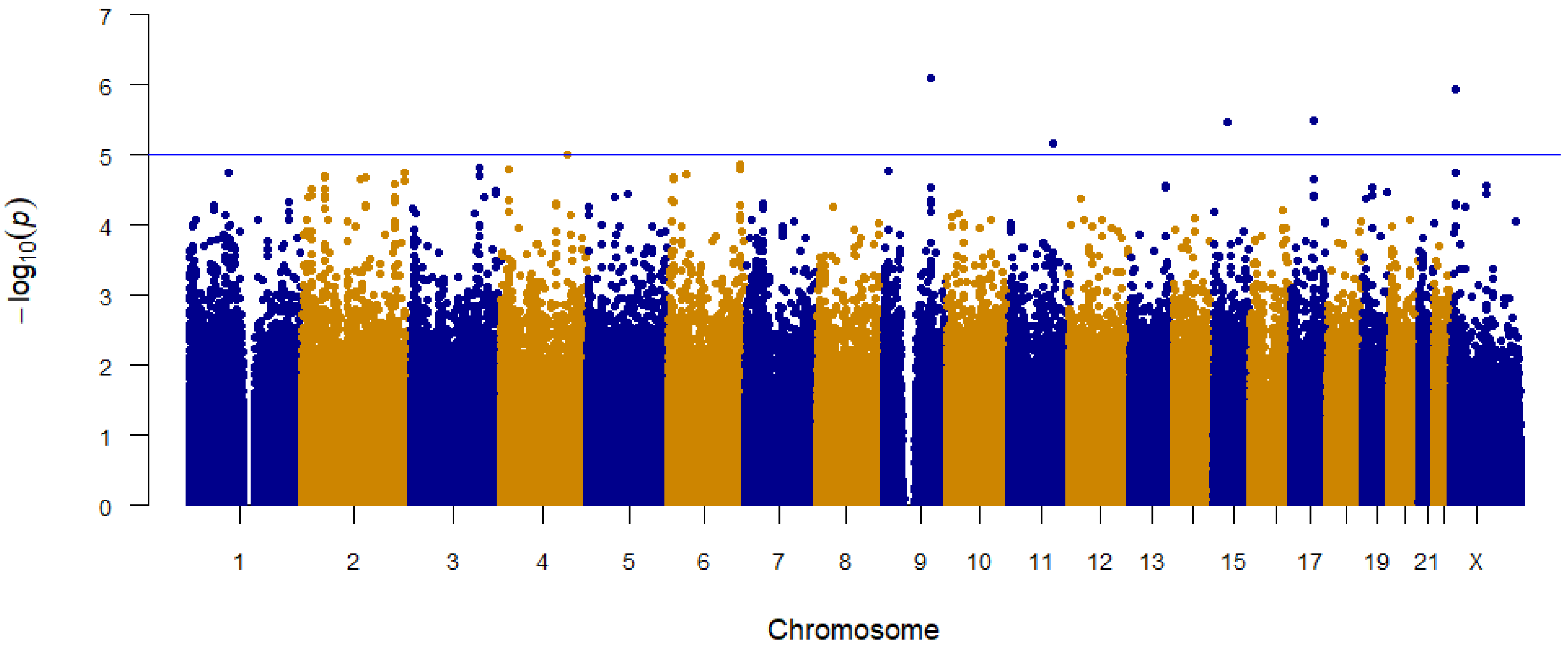

lambda=0.97

DDT, O,P'-

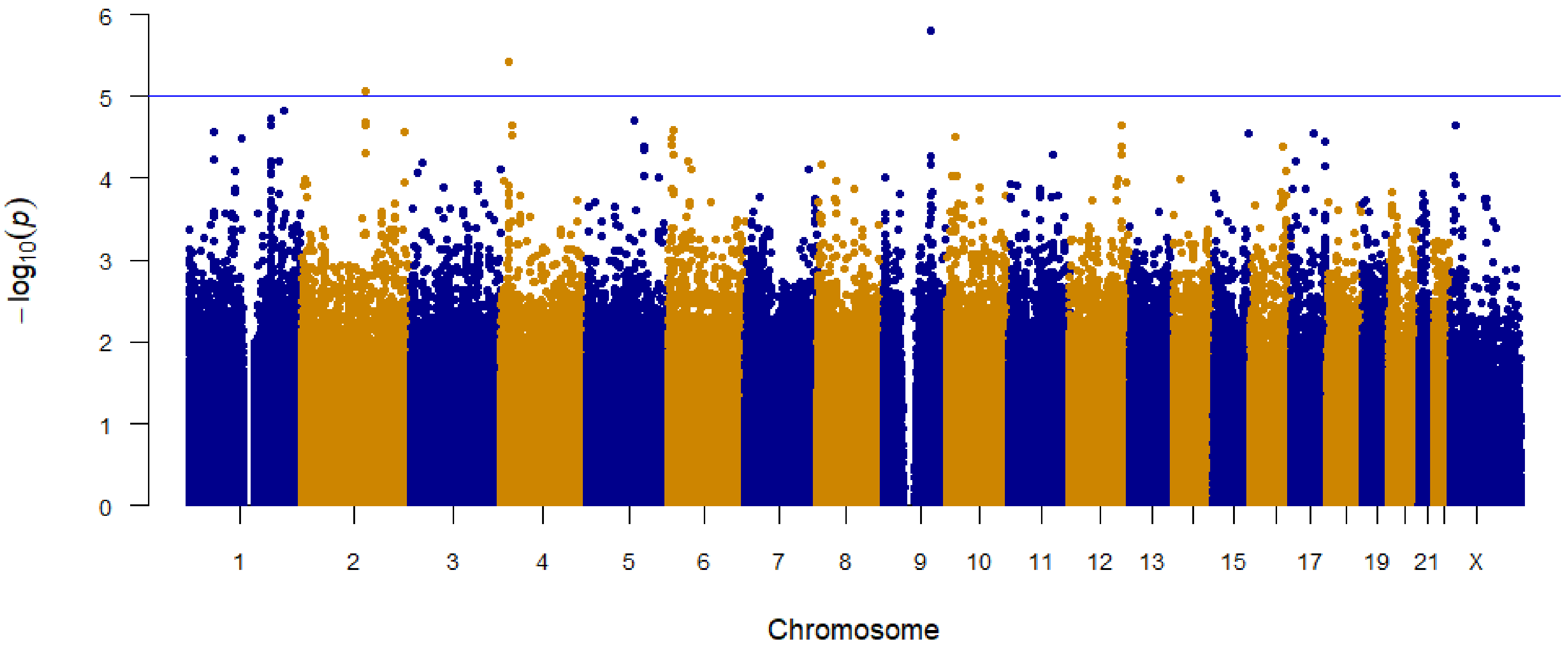

lambda=0.97

DDT, P,P'-

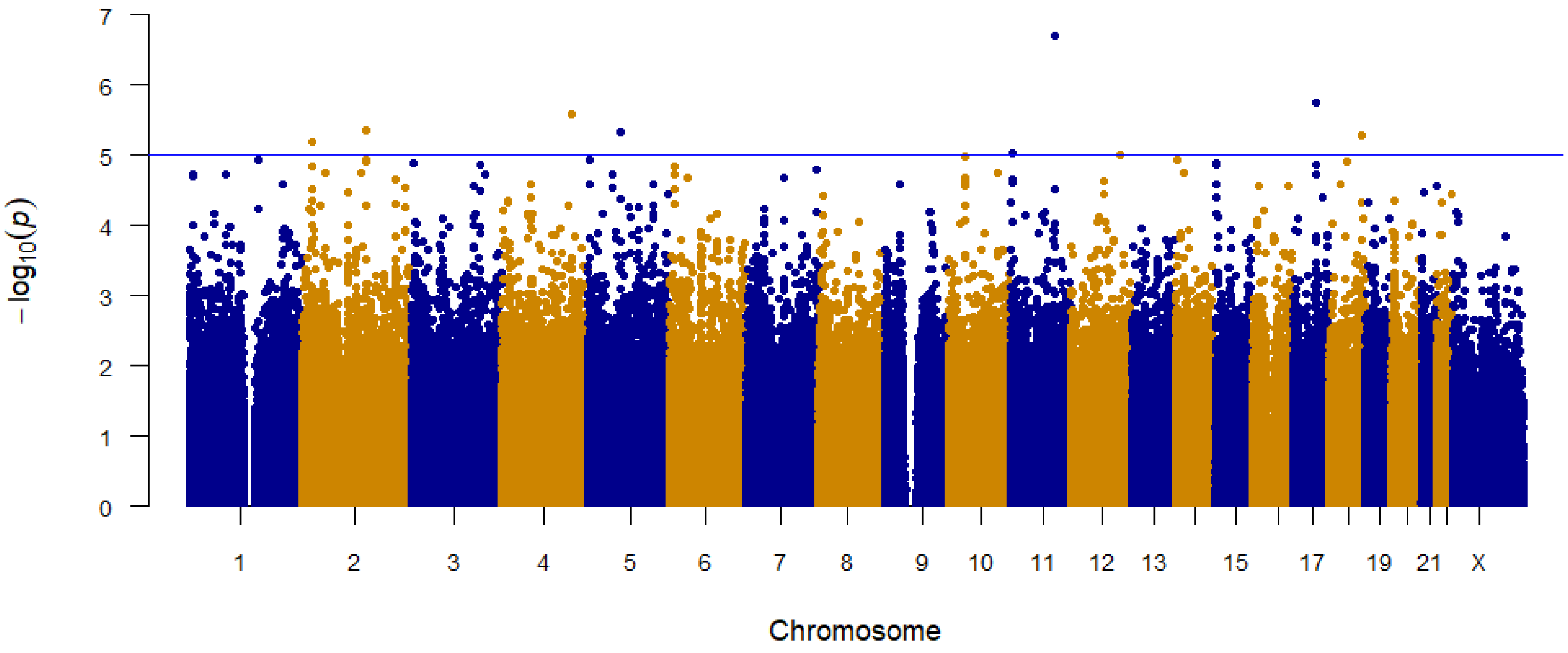

lambda=1.00

DI-N-BUTYL PHTHALATE

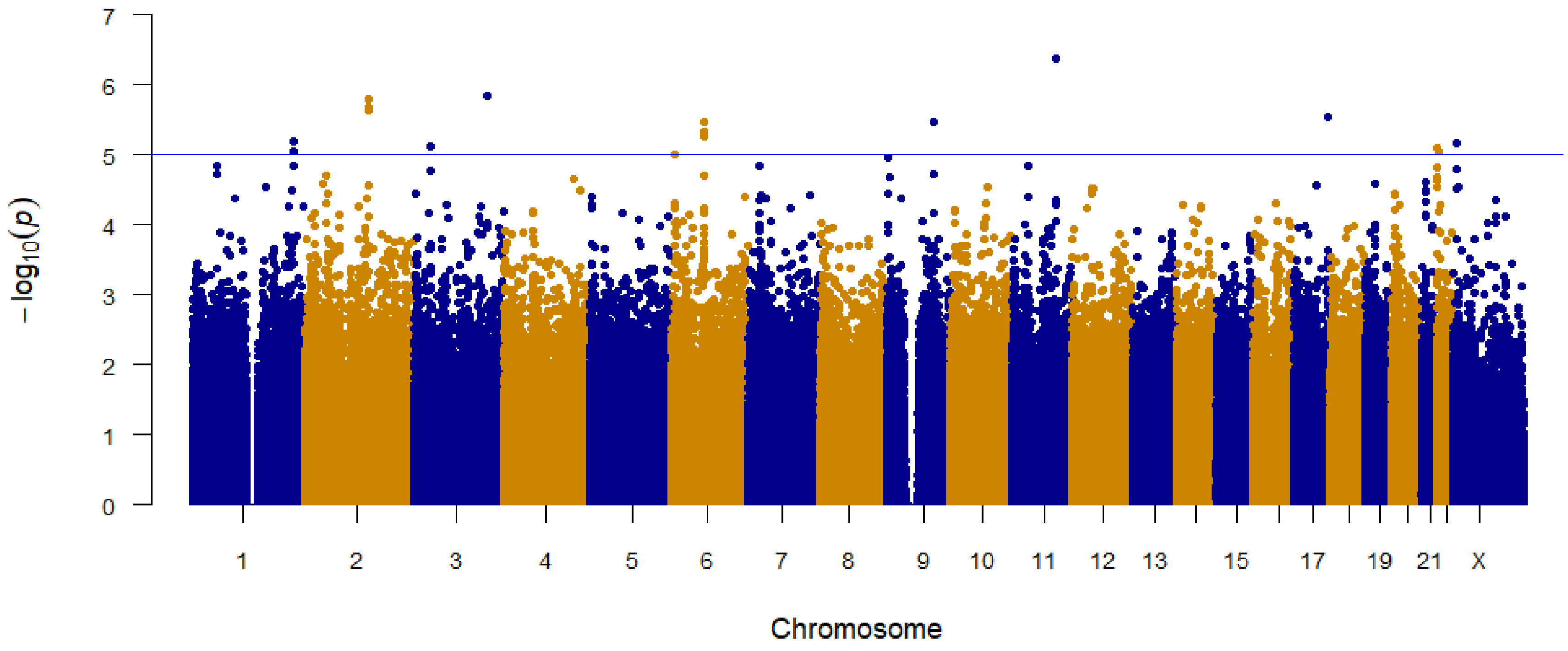

lambda=1.05

DIAZINON

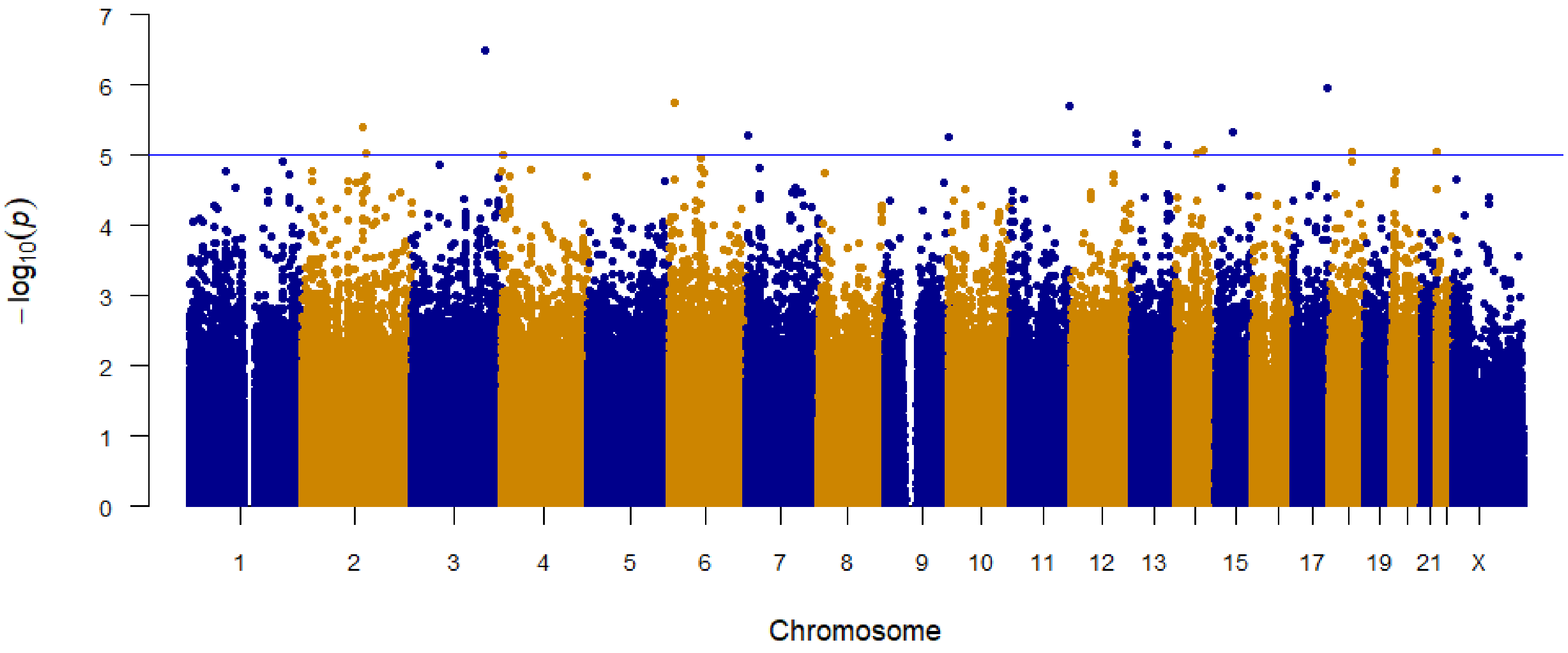

lambda=1.09

DICOFOL

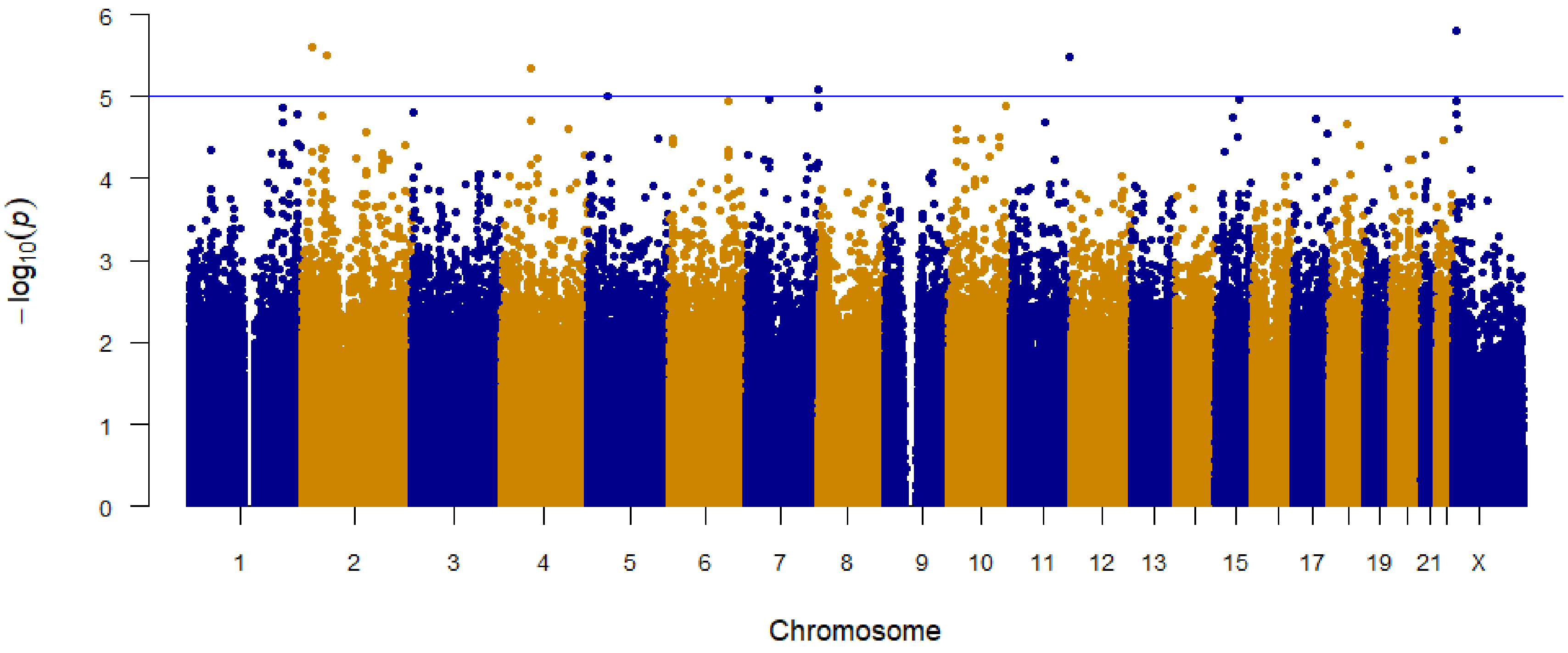

lambda=1.03

DIELDRIN

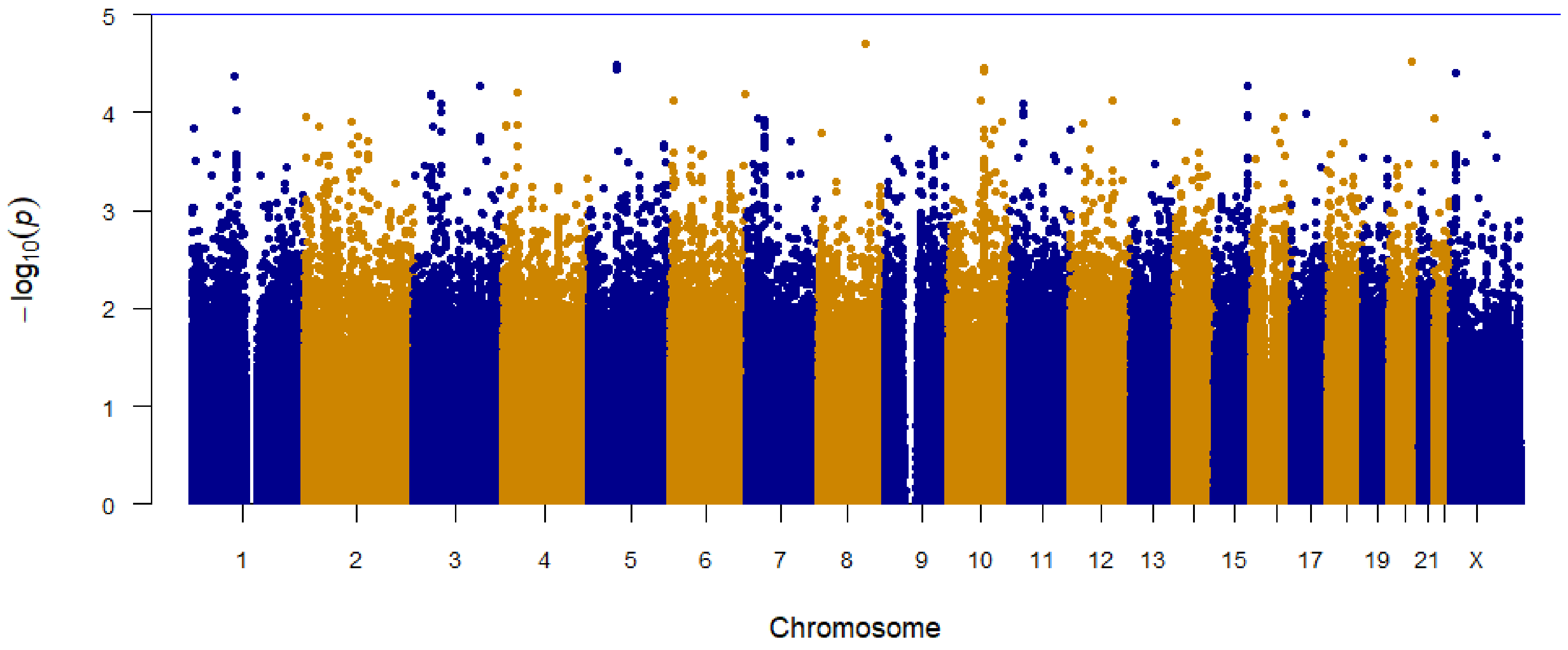

lambda=0.86

DISULFOTON

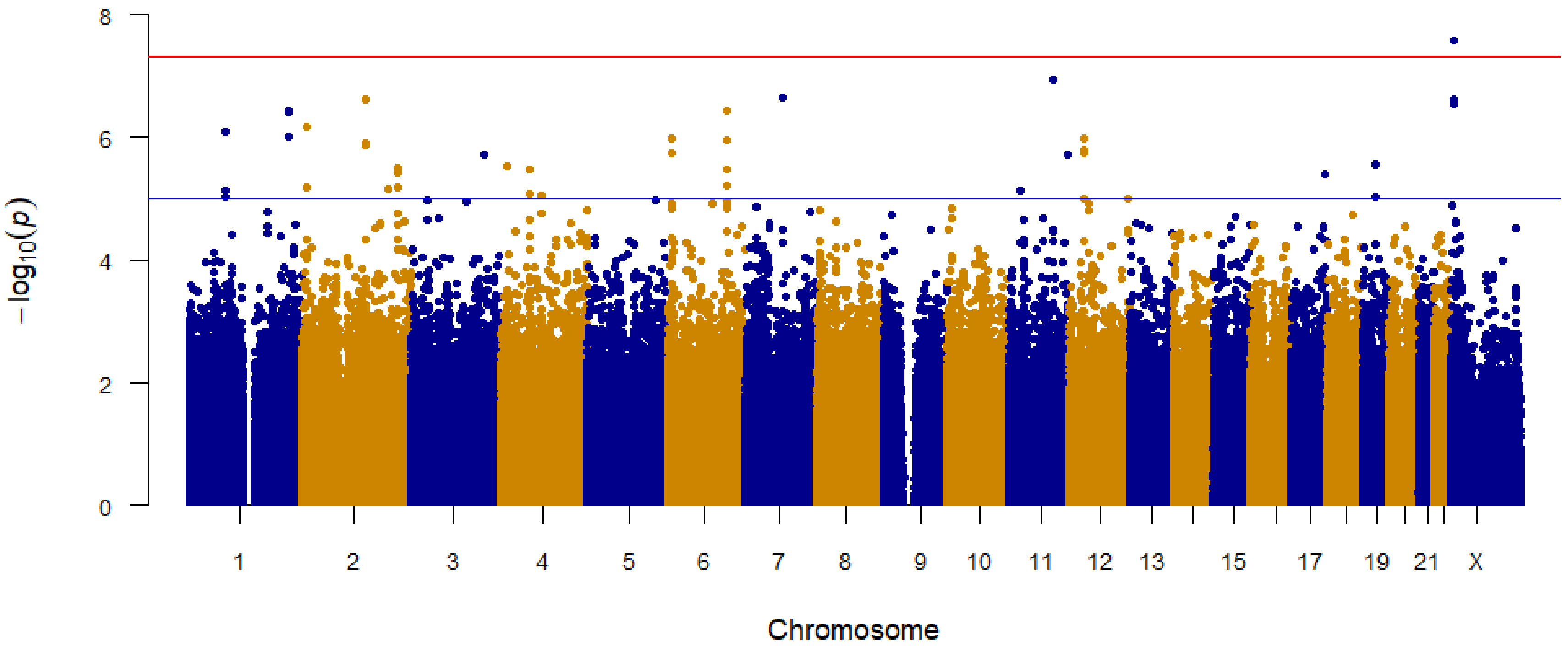

lambda=1.12

ENDOSULFAN

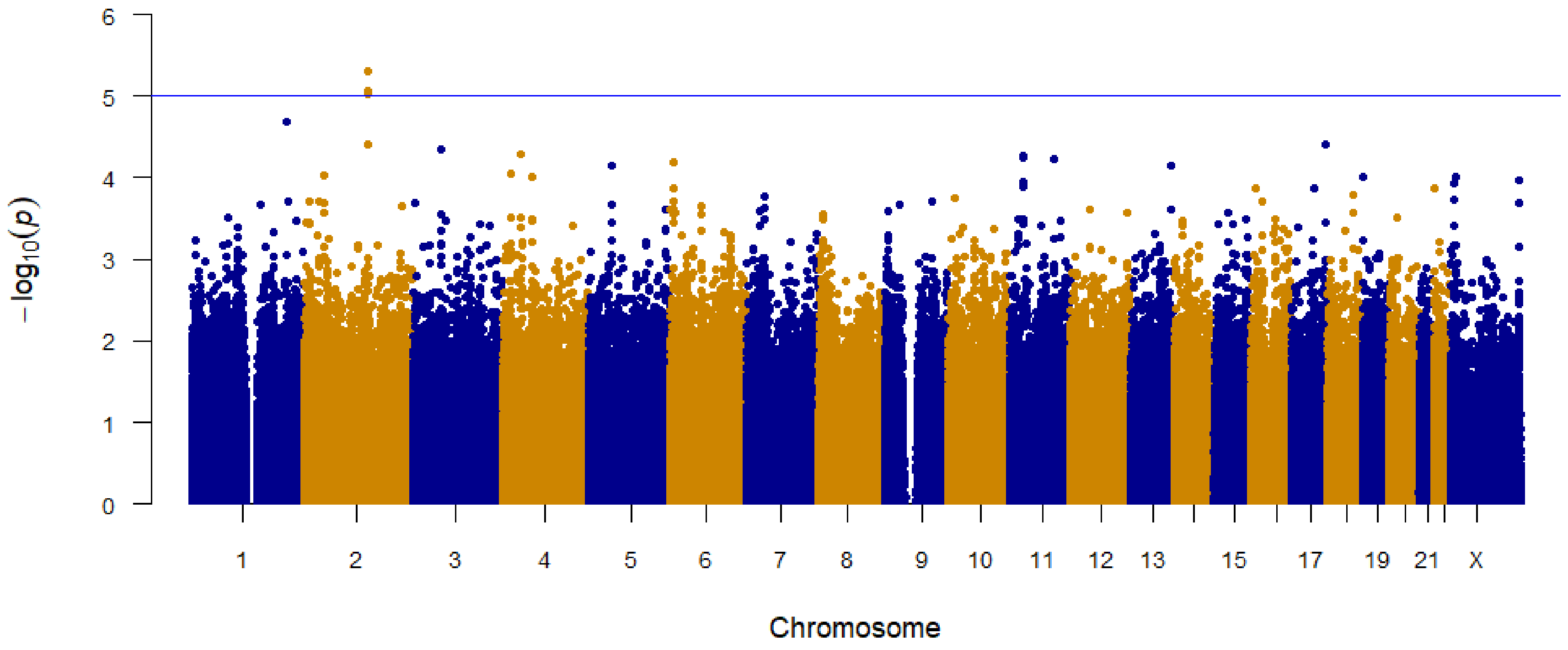

lambda=0.82

ENDRIN

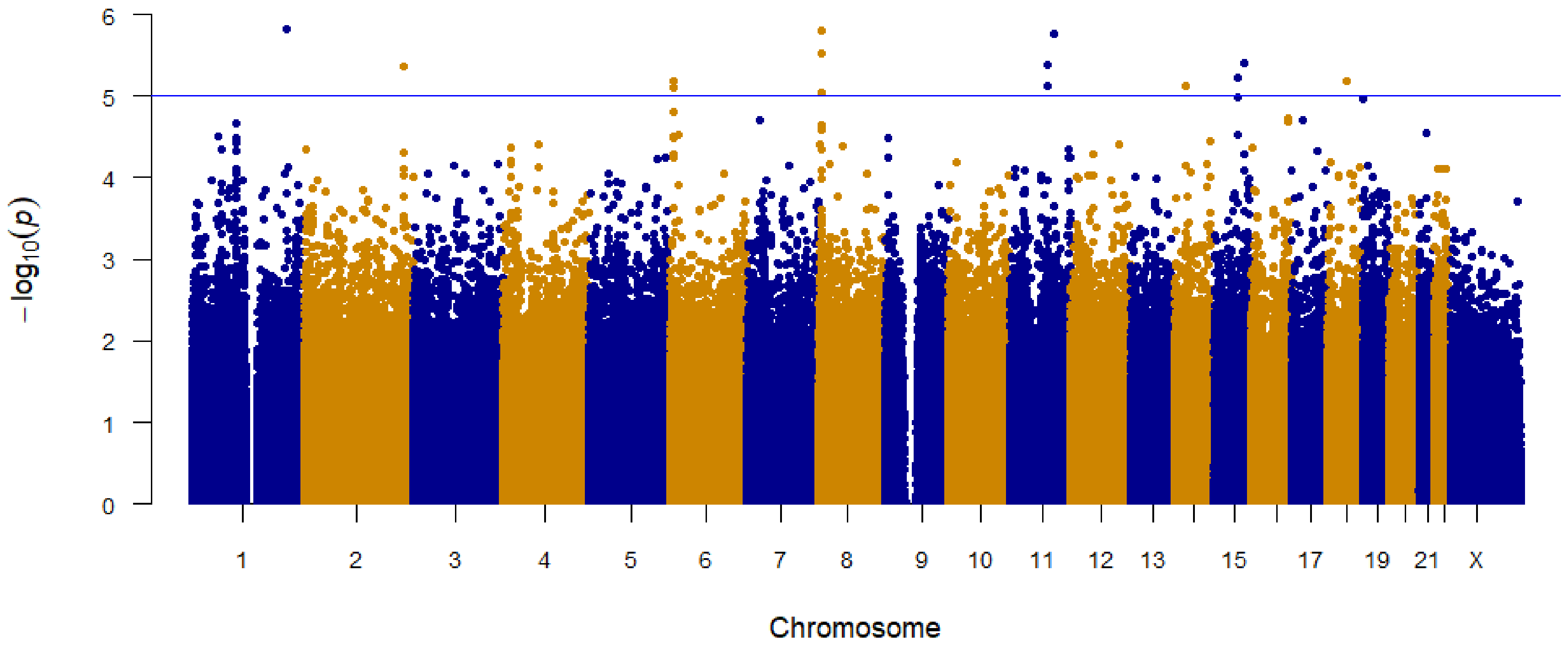

lambda=1.02

ETHION

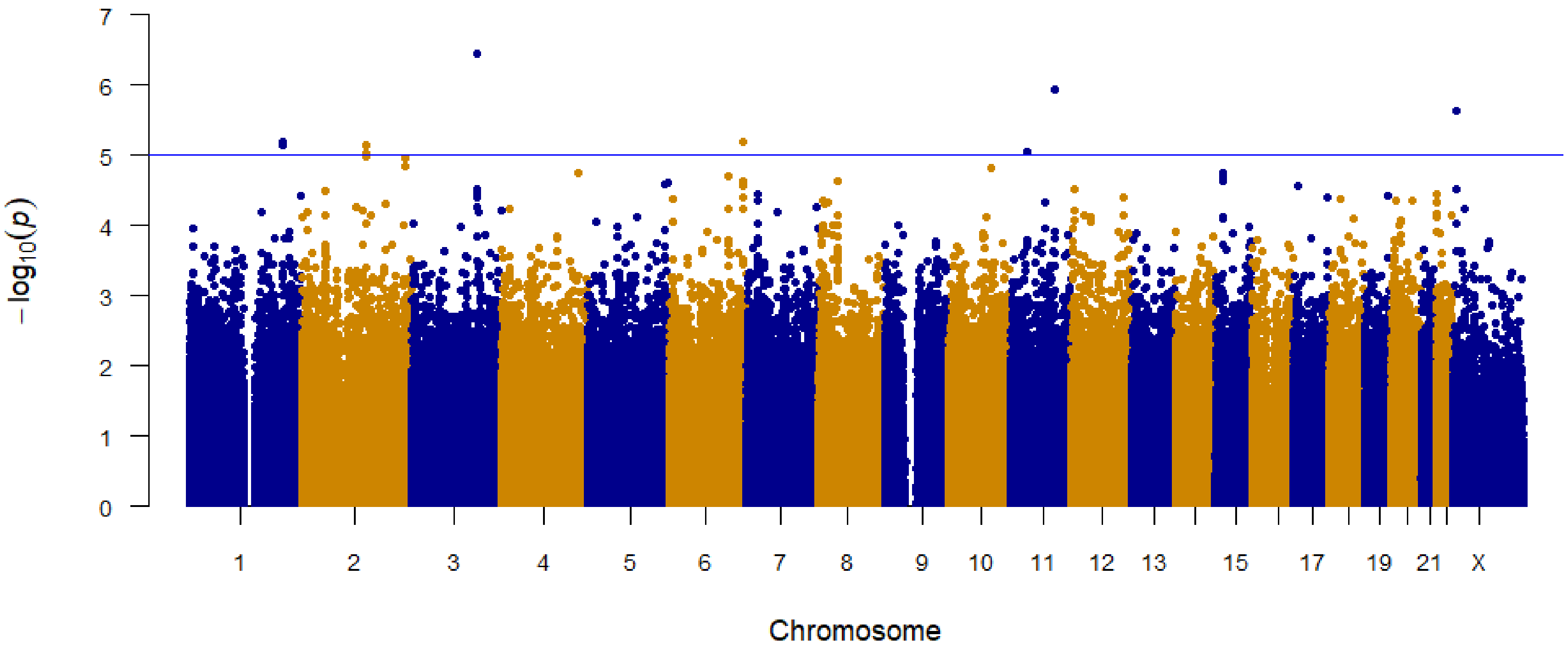

lambda=1.01

HEPTACHLOR

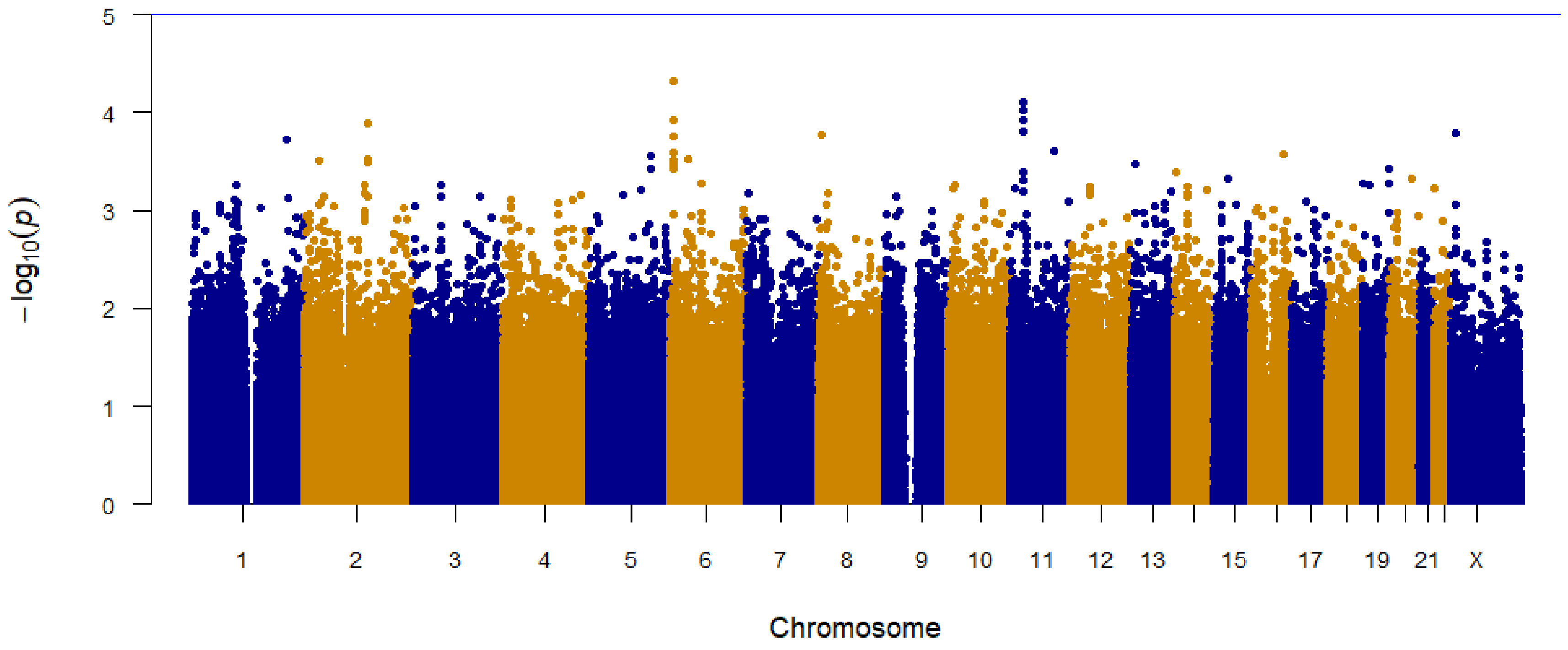

lambda=0.72

HEPTACHLOR EPOXIDE

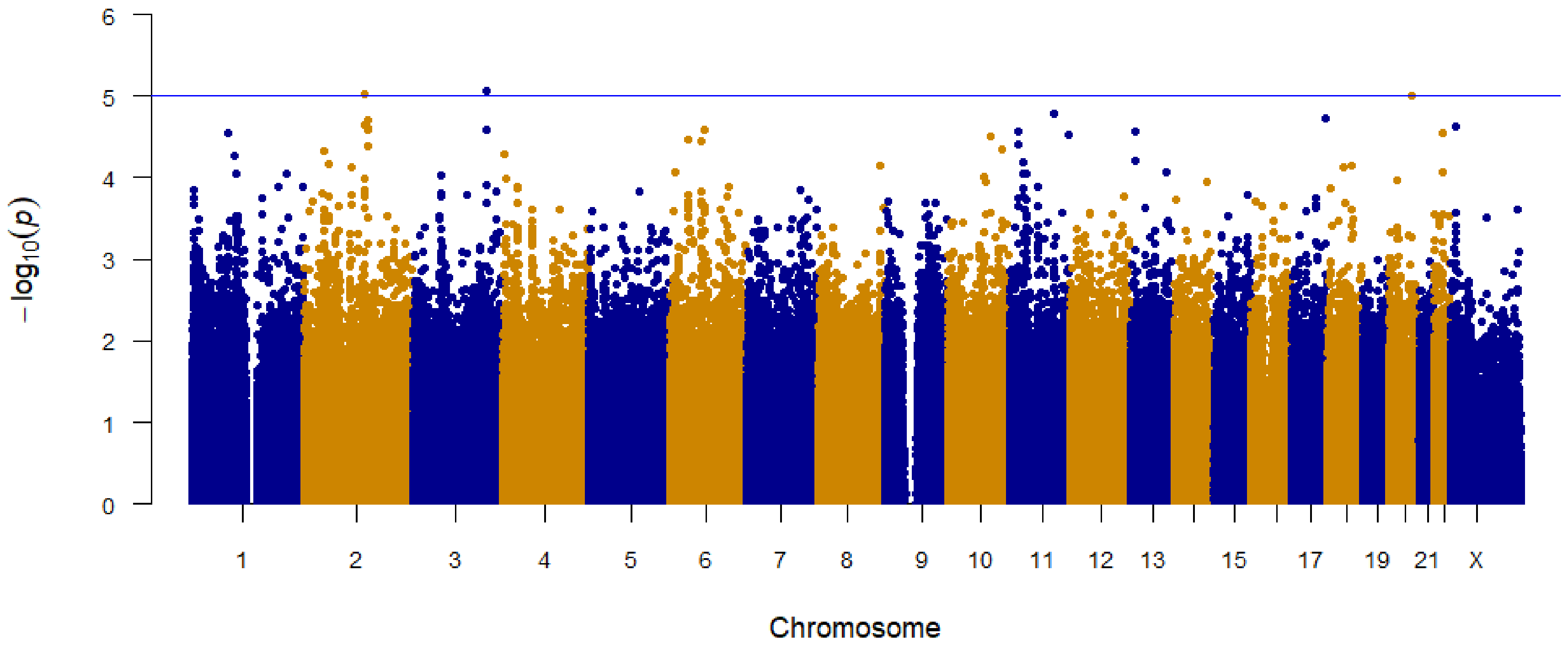

lambda=0.89

MERCURIC CHLORIDE

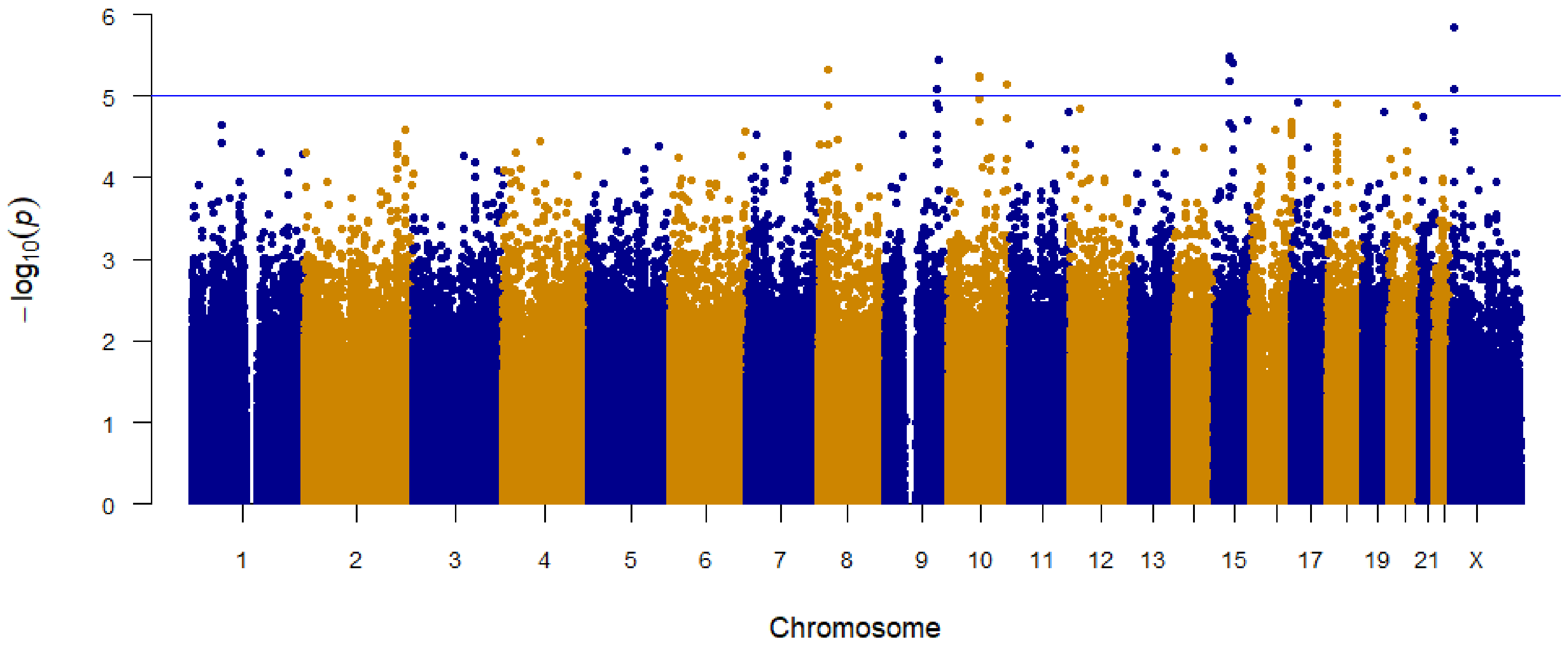

lambda=1.04

MERCURIC CHLORIDE-2

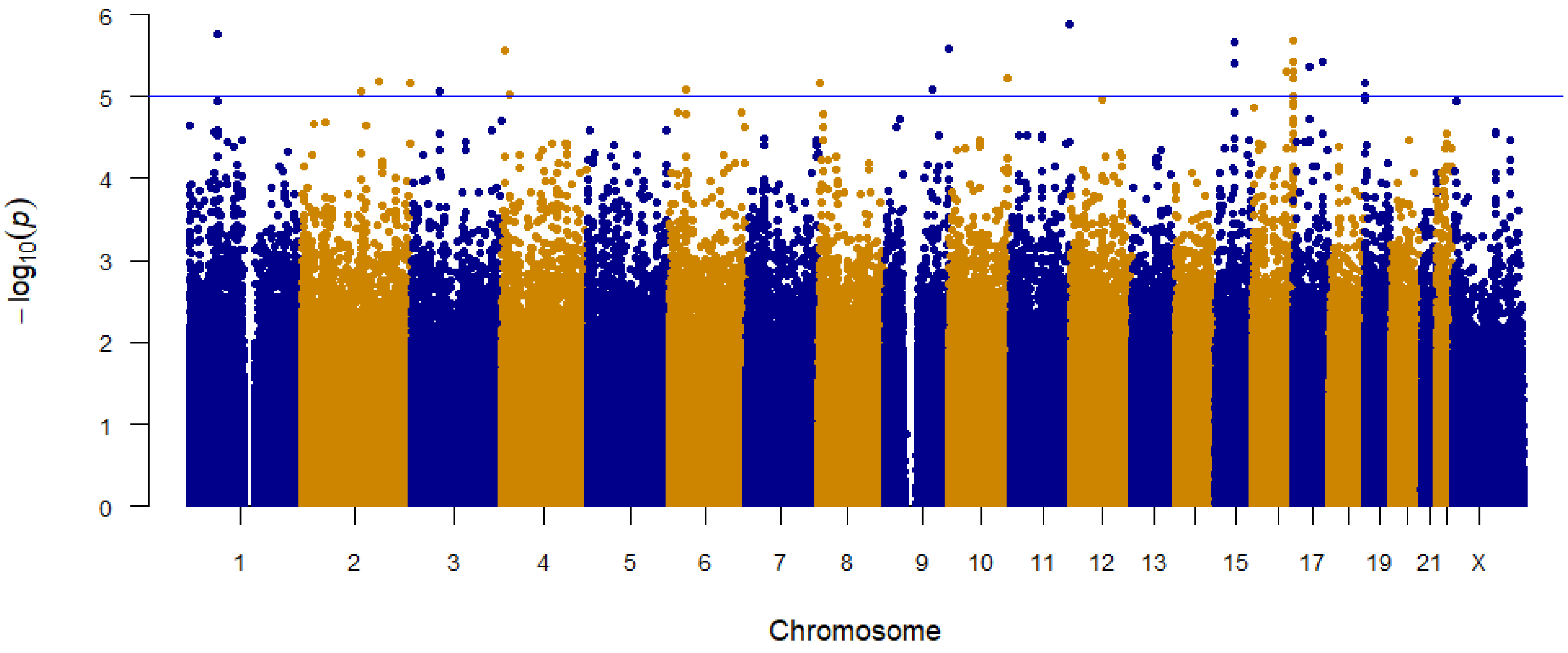

lambda=1.14

METHOXYCHLOR

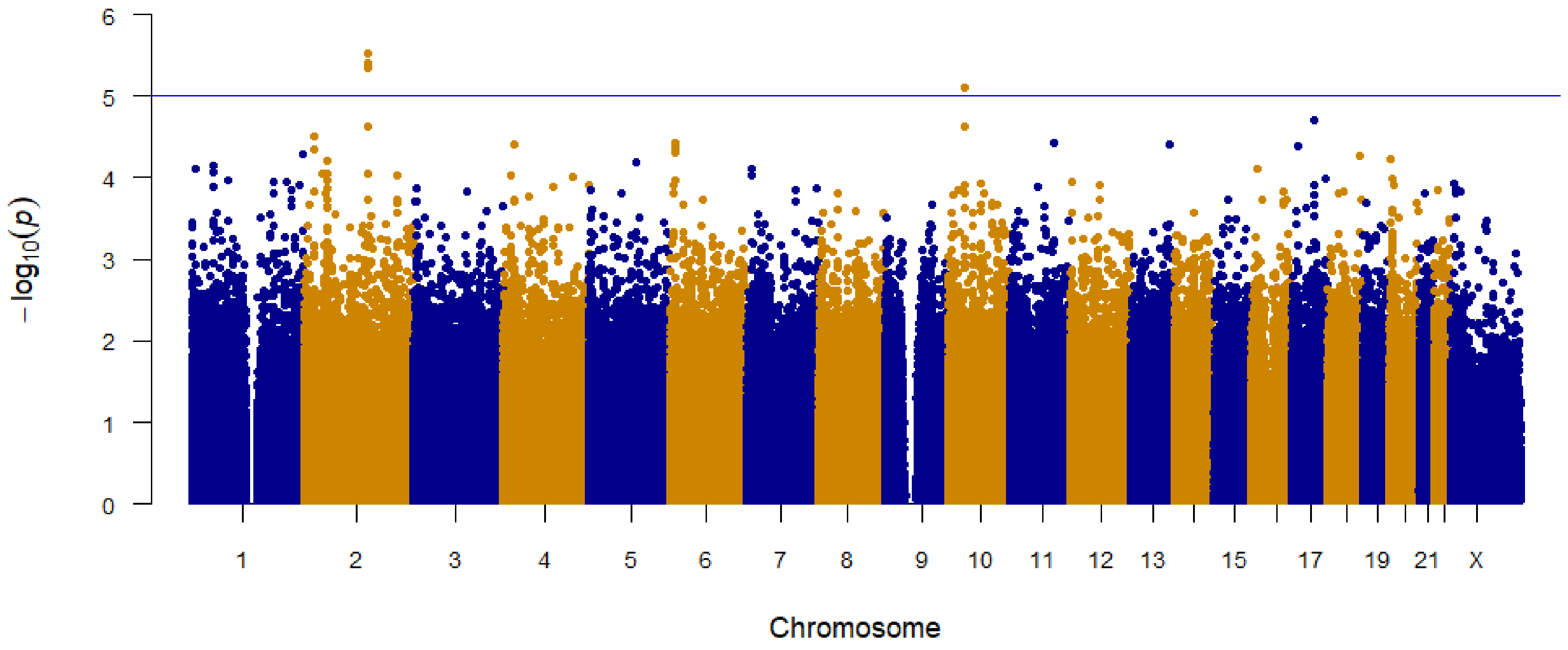

lambda=0.93

NICKEL

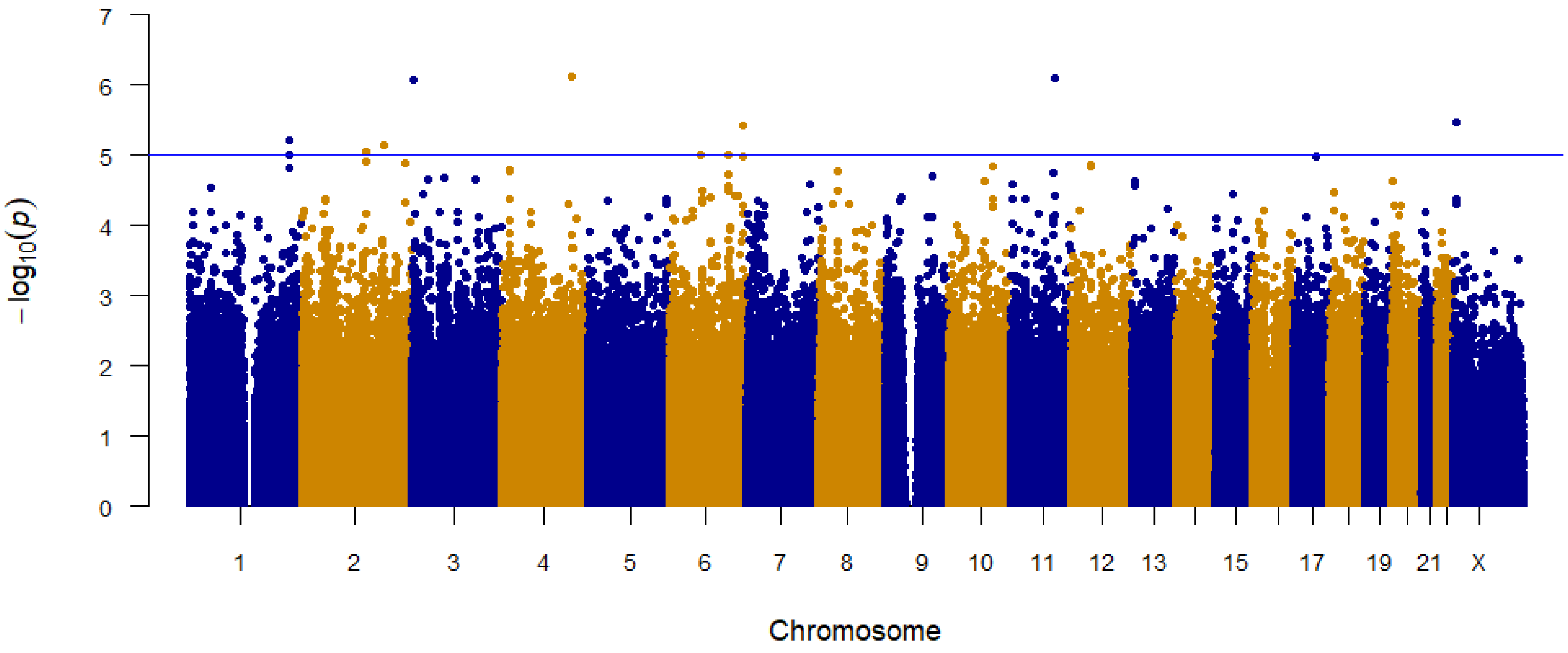

lambda=1.06

PARATHION

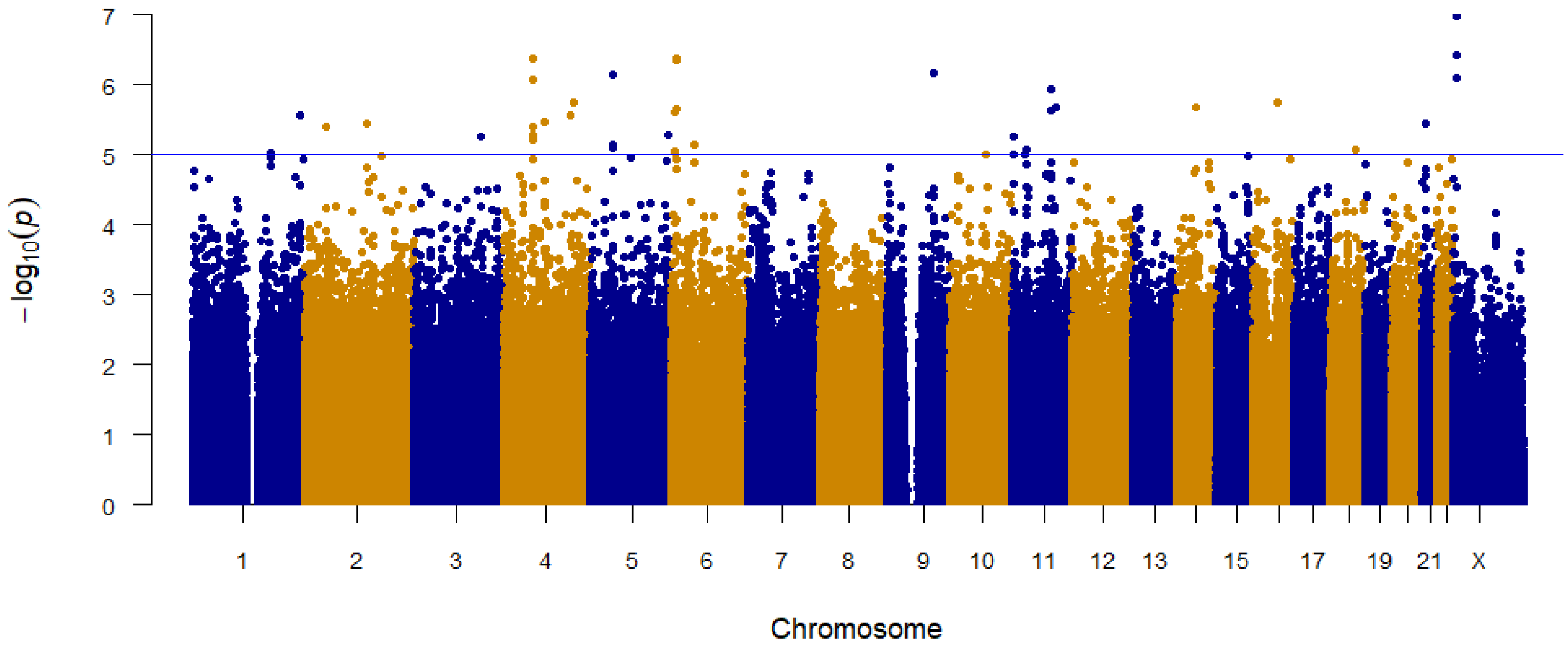

lambda=1.19

PENTACHLOROPHENOL

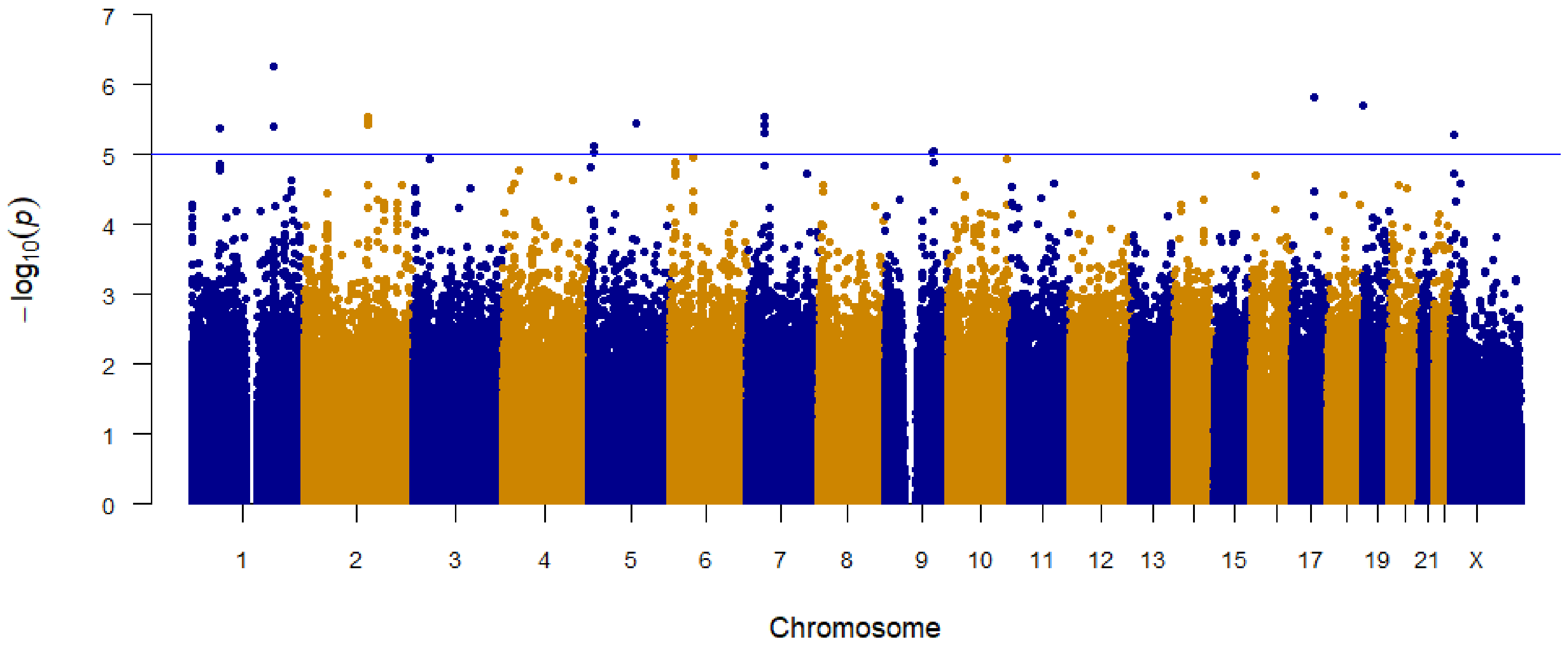

lambda=1.07

Potassium Chromate

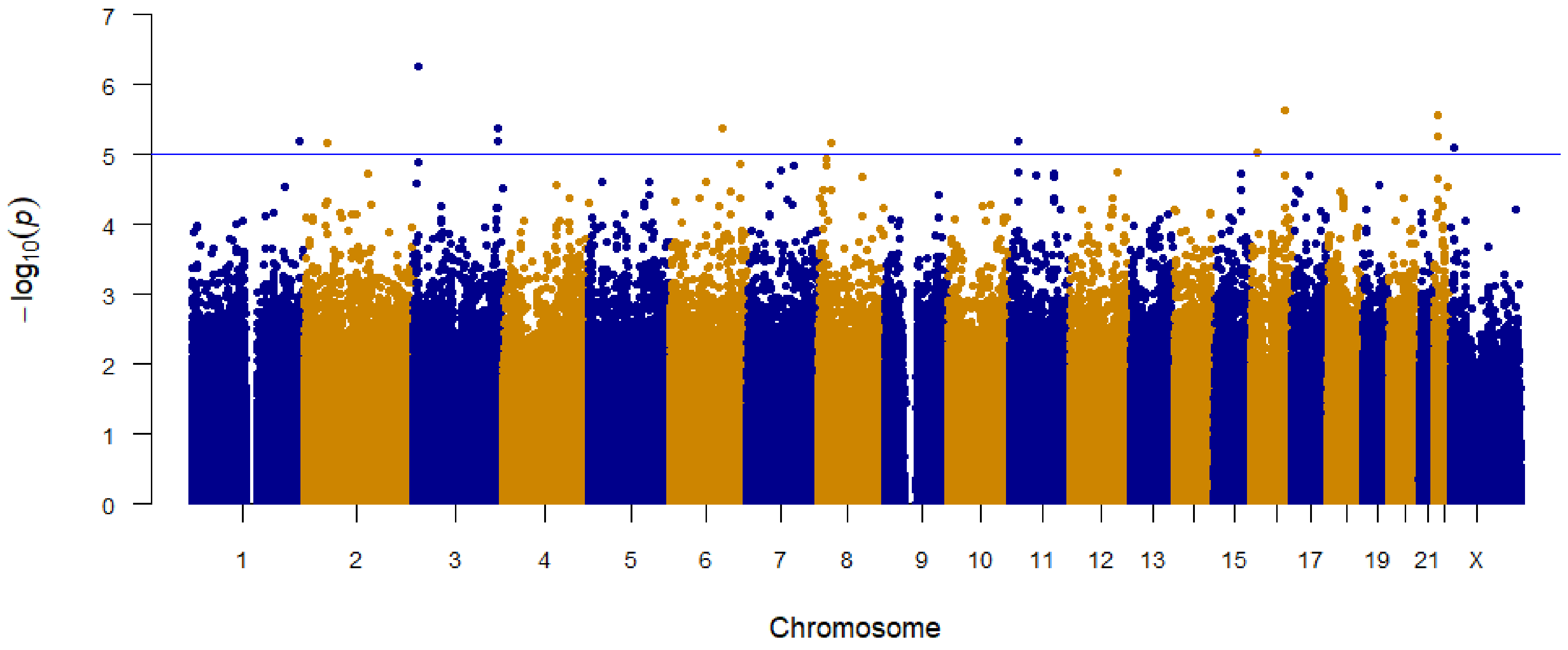

lambda=1.11

AC50-Low

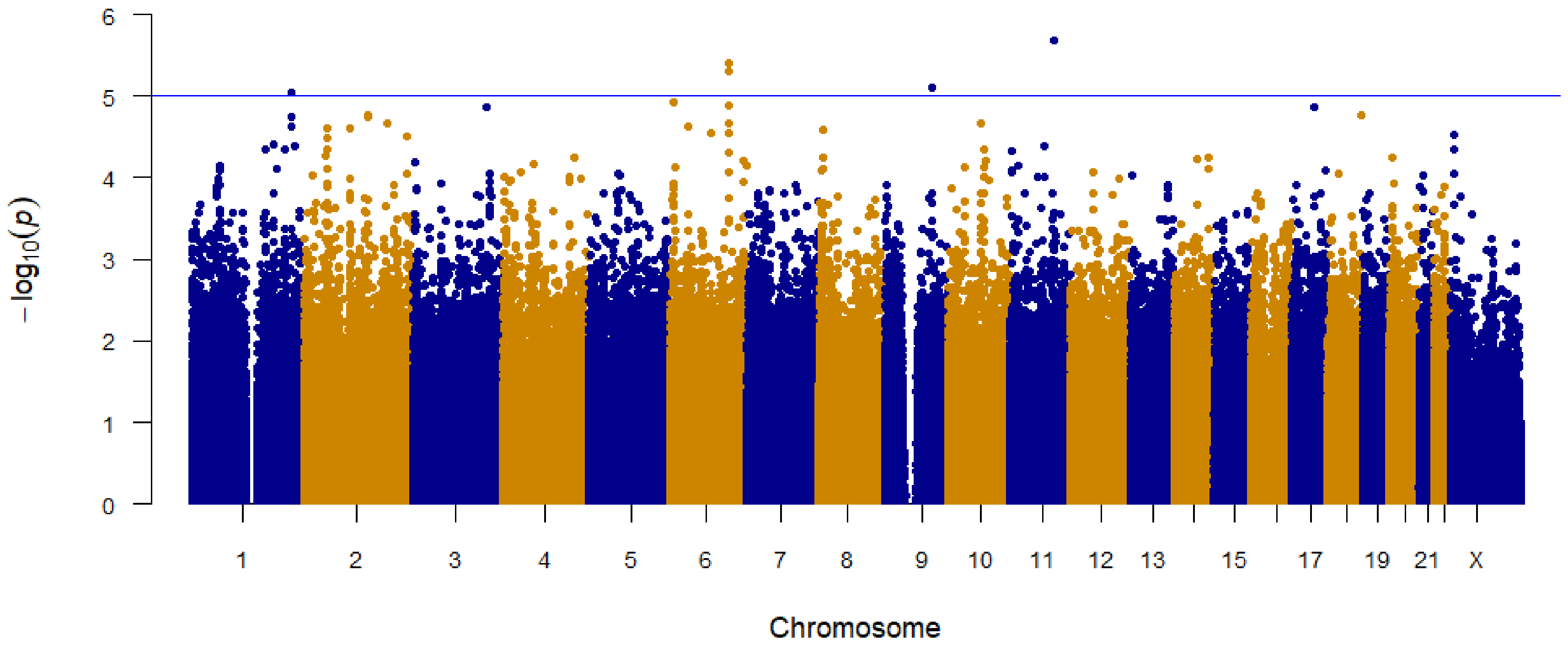

lambda=0.97

AC50-High

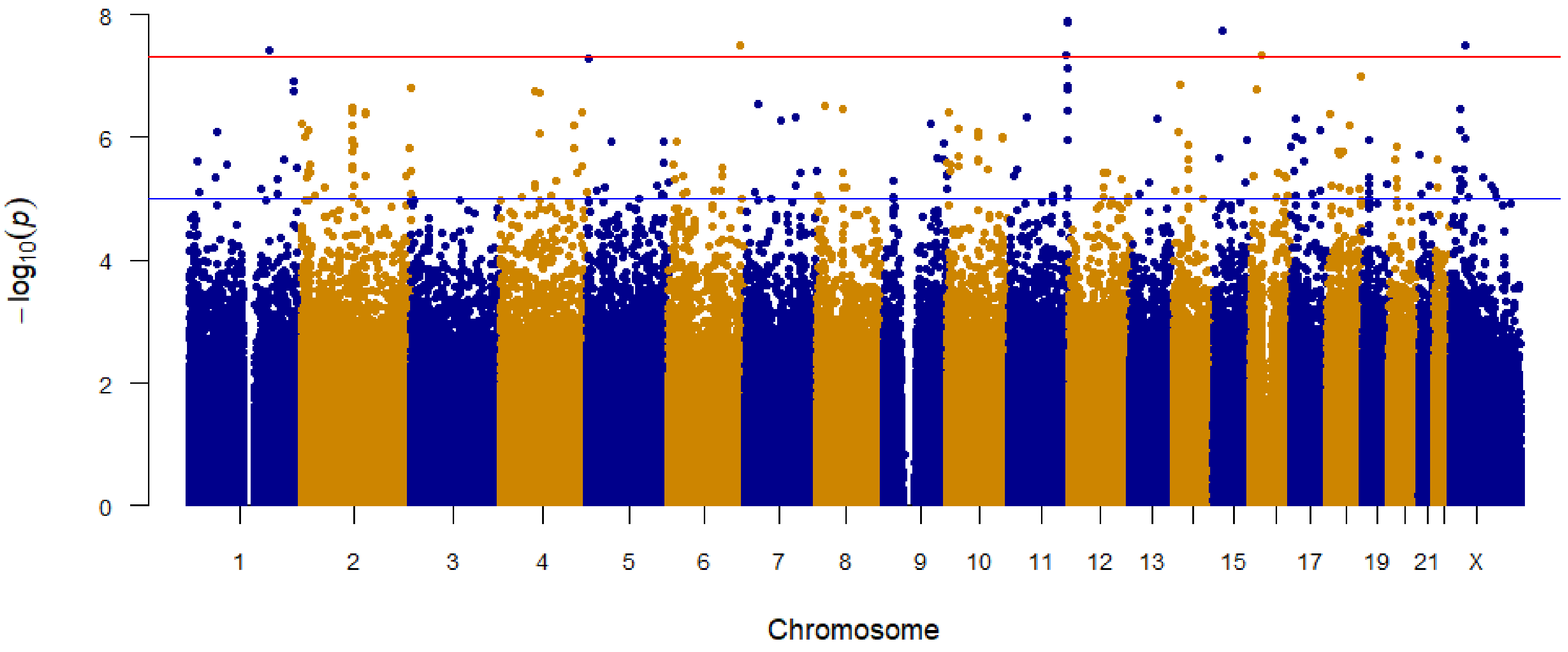

lambda=1.30

POD-Low

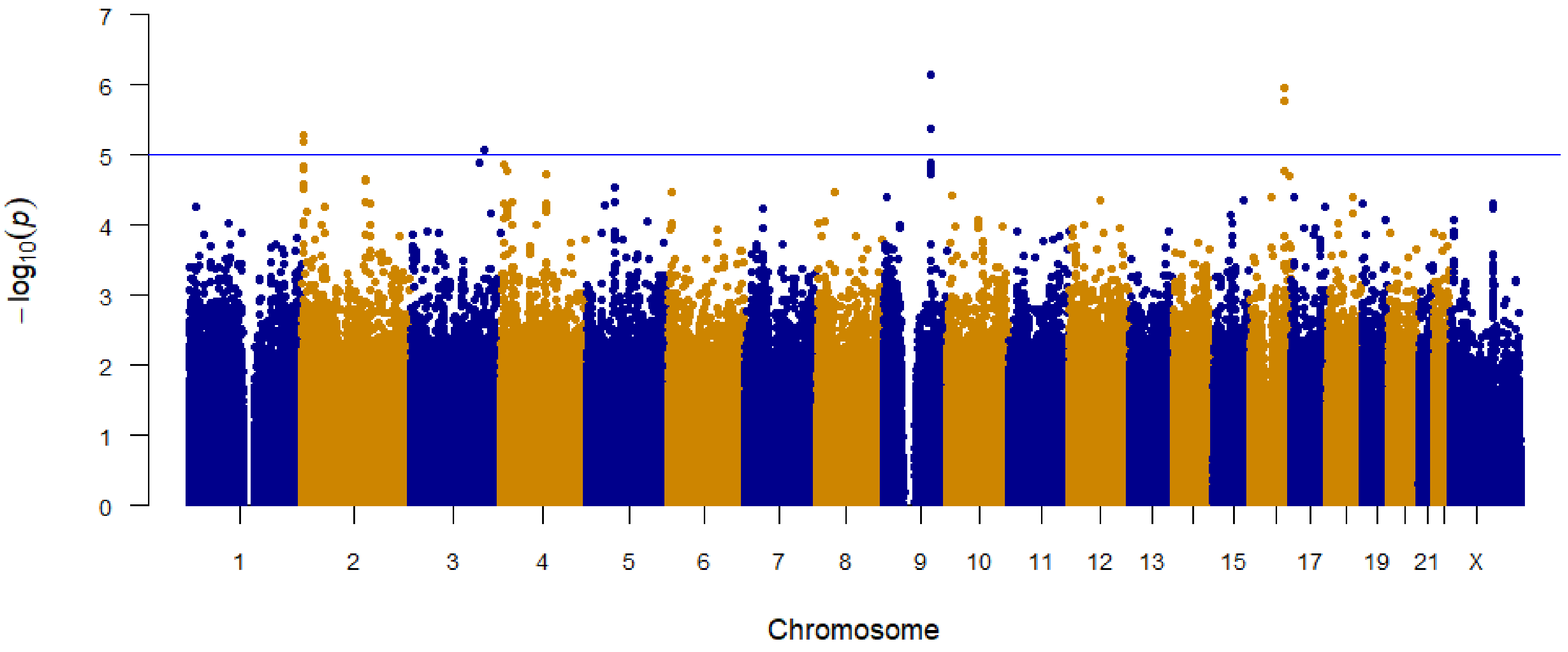

lambda=0.97

POD-High

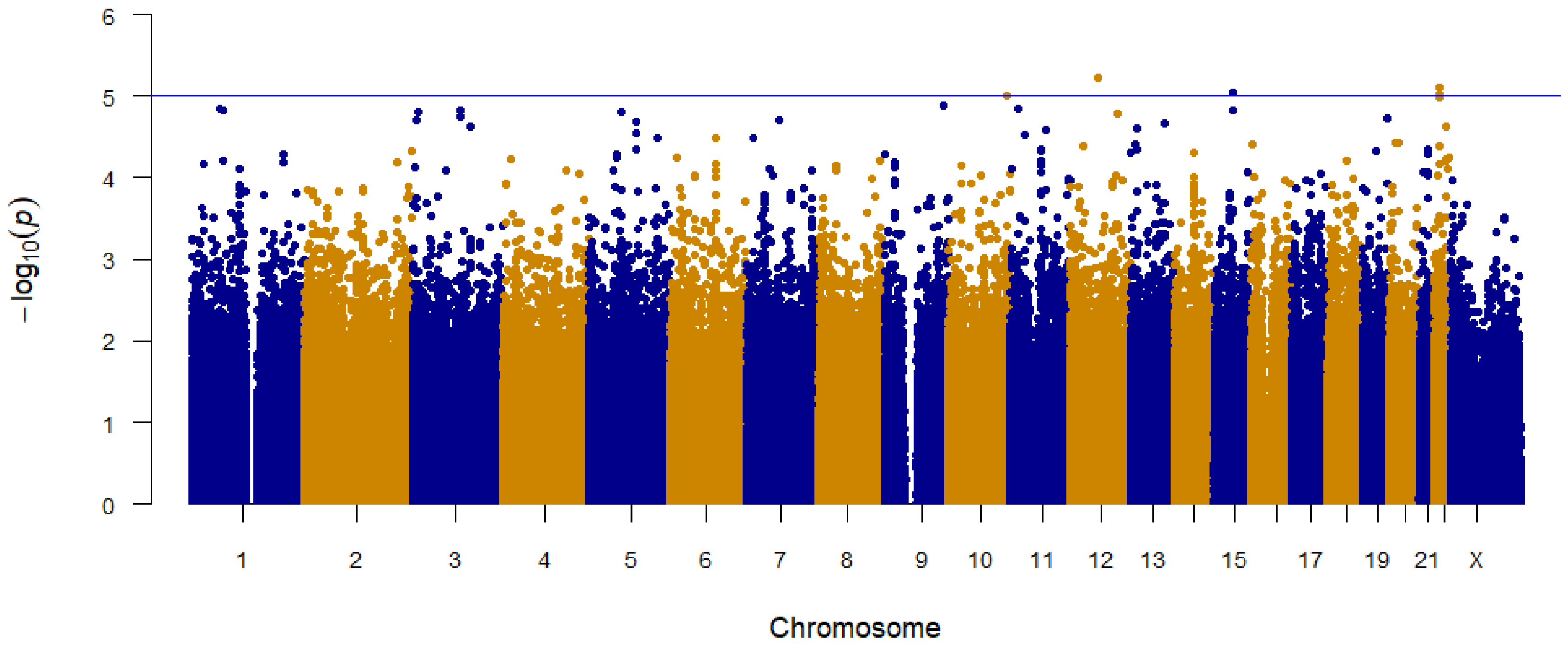

lambda=0.99

Expo-Low

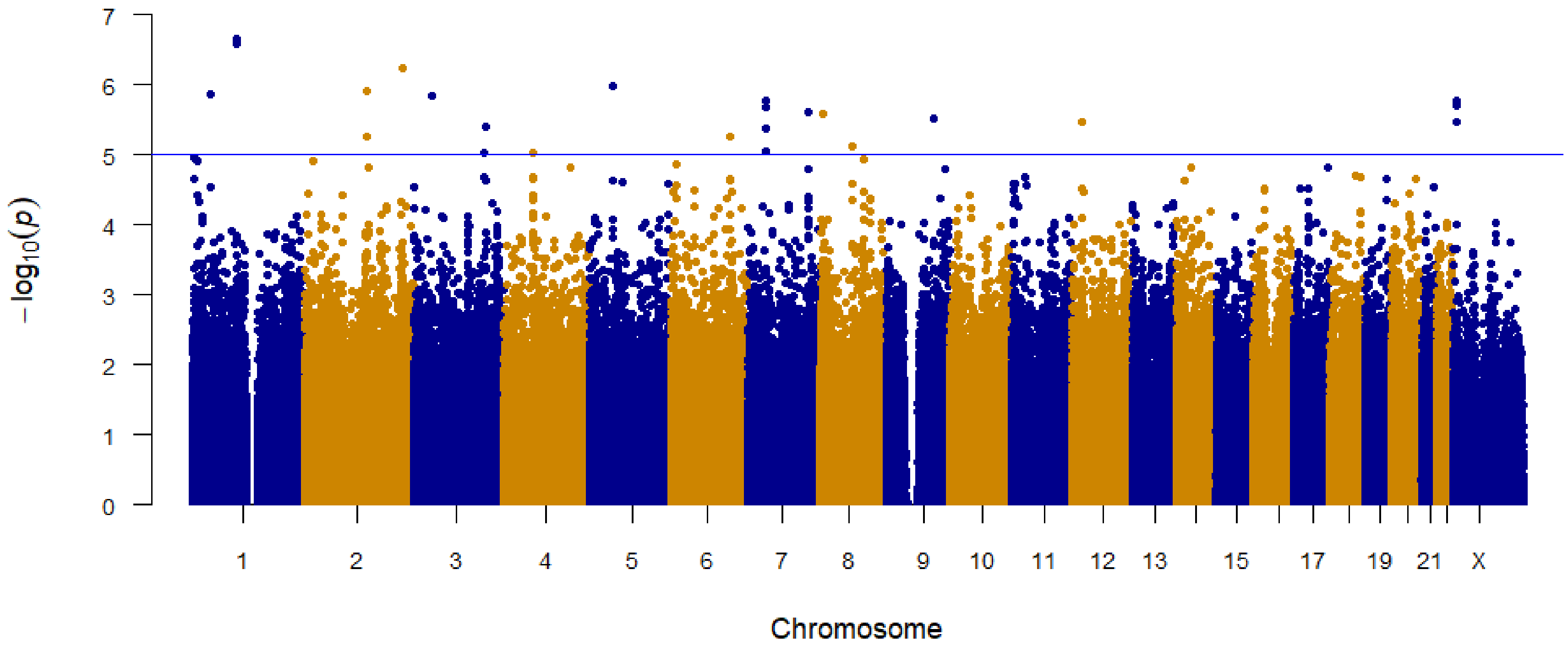

lambda=1.09

Expo-High

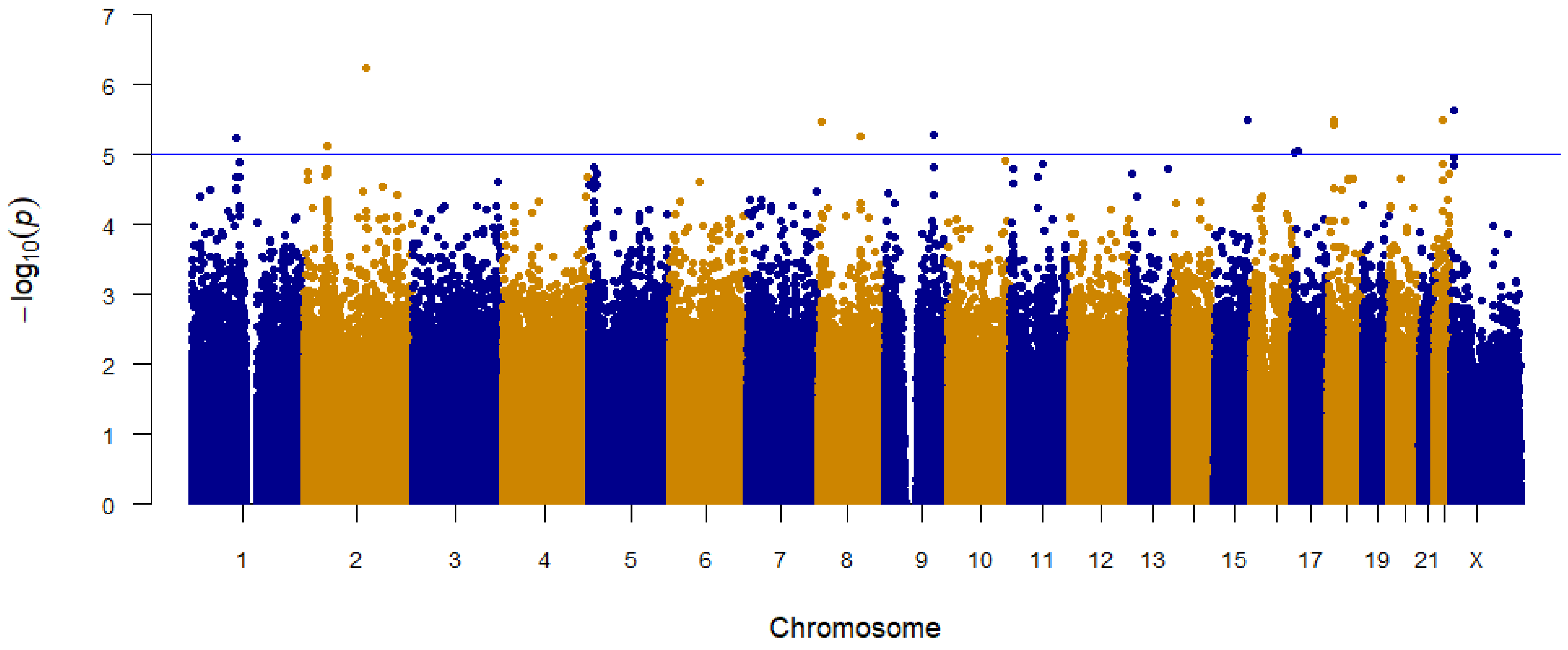

lambda=1.09

RfD-Low

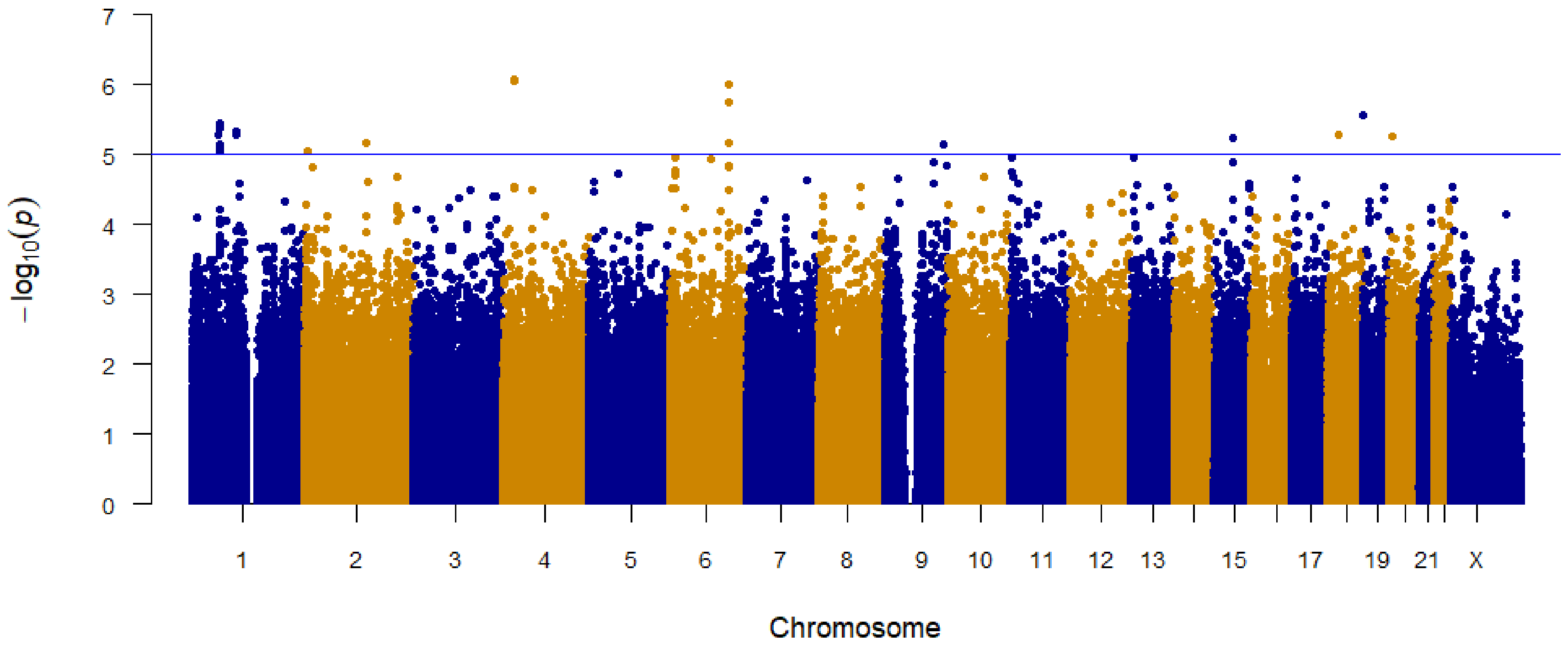

lambda=1.11

RfD-High

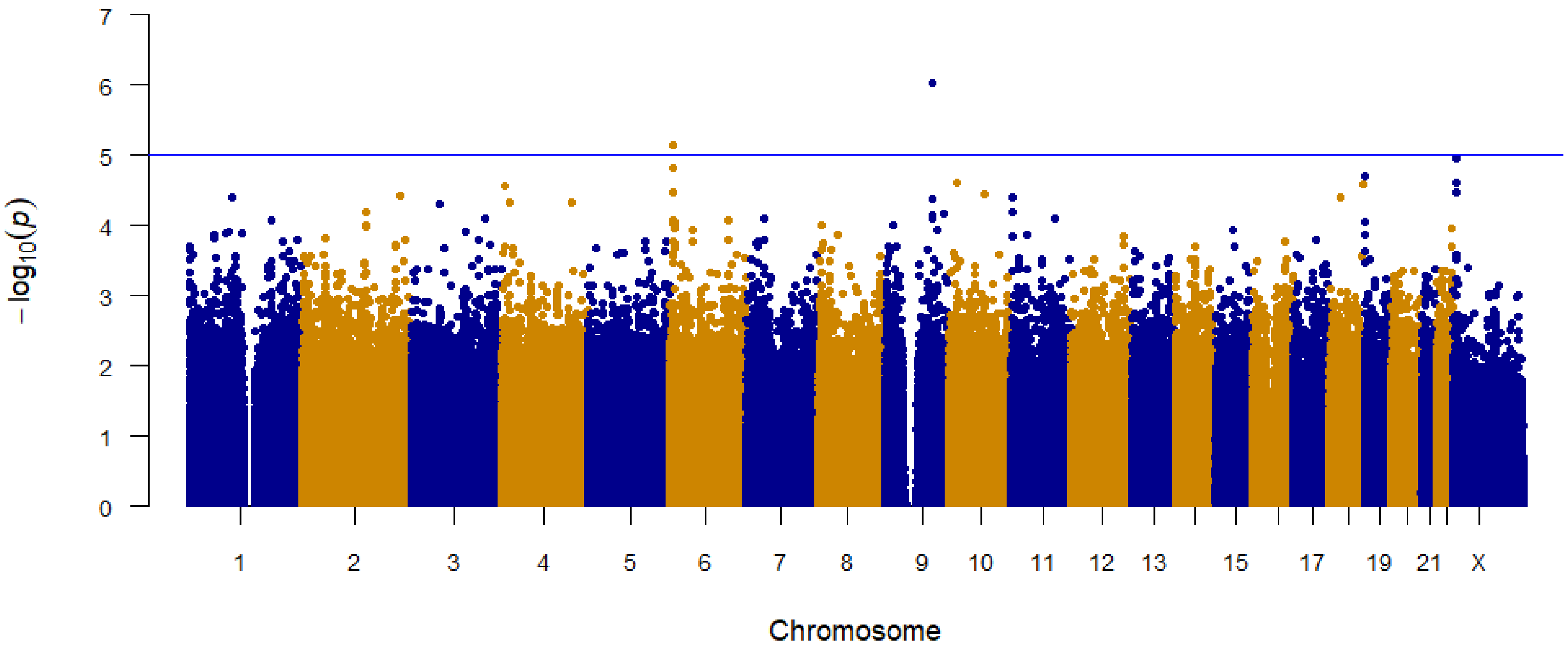

lambda=0.94
