# Supplementary material for: A Population-Based Human In Vitro Approach to Quantify Inter-Individual Variability in Responses to Chemical Mixtures
Source: Toxics. 2022 Aug 1;10(8):441. doi: 10.3390/toxics10080441 (PMC9413237; doi:10.3390/toxics10080441)
Supplement: Supplementary file 1 [file toxics-10-00441-s001.zip › Figure S4 Cell-line specific concentration-response curves.pdf]

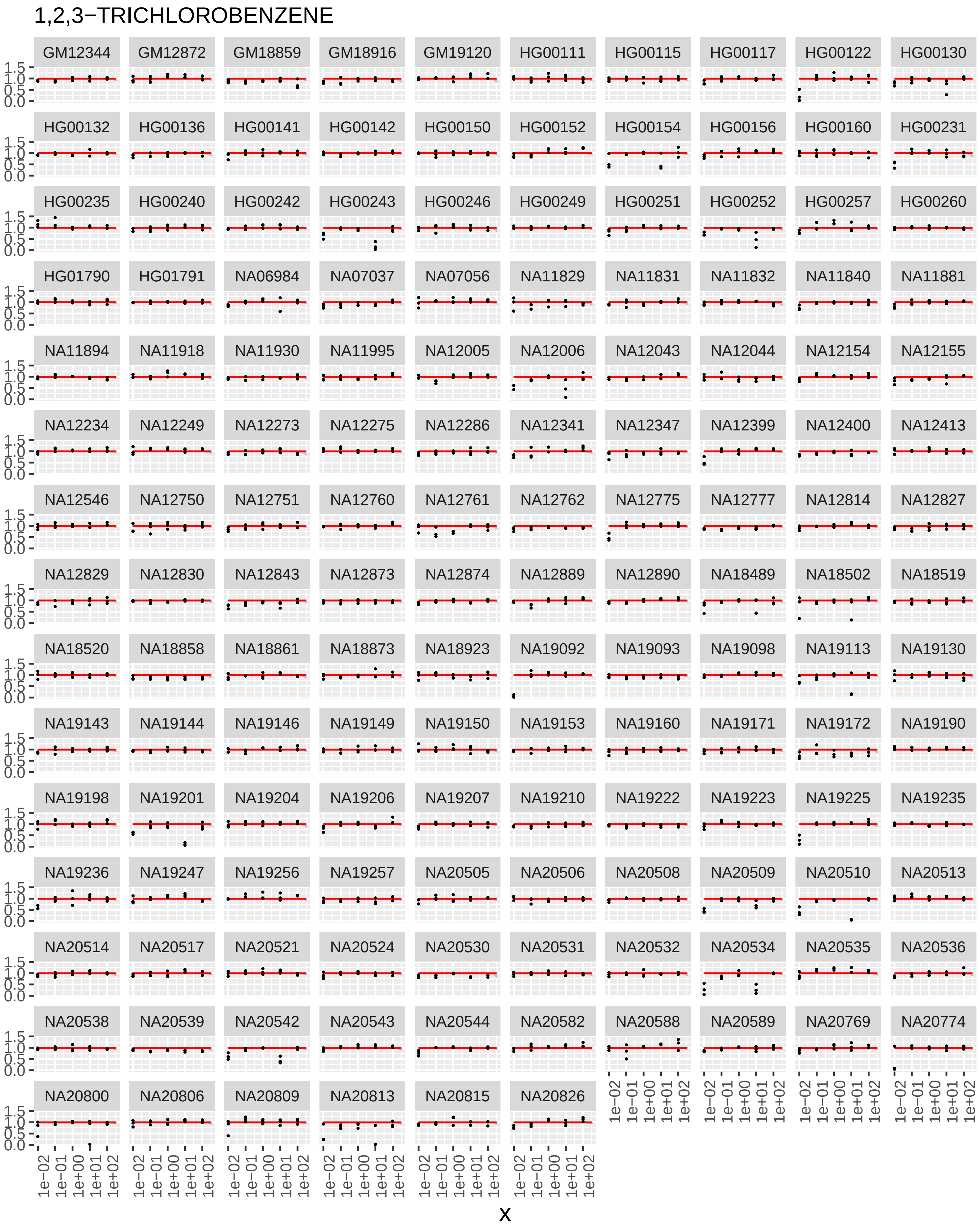
1,2,3−TRICHLOROBENZENE
GM12344 GM12872 GM18859 GM18916 GM19120 HG00111 HG00115 HG00117 HG00122 HG00130
HG00132 HG00136 HG00141 HG00142 HG00150 HG00152 HG00154 HG00156 HG00160 HG00231
HG00235 HG00240 HG00242 HG00243 HG00246 HG00249 HG00251 HG00252 HG00257 HG00260
HG01790 HG01791 NA06984 NA07037 NA07056 NA11829 NA11831 NA11832 NA11840 NA11881
NA11894 NA11918 NA11930 NA11995 NA12005 NA12006 NA12043 NA12044 NA12154 NA12155
NA12234 NA12249 NA12273 NA12275 NA12286 NA12341 NA12347 NA12399 NA12400 NA12413
NA12546 NA12750 NA12751 NA12760 NA12761 NA12762 NA12775 NA12777 NA12814 NA12827
NA12829 NA12830 NA12843 NA12873 NA12874 NA12889 NA12890 NA18489 NA18502 NA18519
NA18520 NA18858 NA18861 NA18873 NA18923 NA19092 NA19093 NA19098 NA19113 NA19130
NA19143 NA19144 NA19146 NA19149 NA19150 NA19153 NA19160 NA19171 NA19172 NA19190
NA19198 NA19201 NA19204 NA19206 NA19207 NA19210 NA19222 NA19223 NA19225 NA19235
NA19236 NA19247 NA19256 NA19257 NA20505 NA20506 NA20508 NA20509 NA20510 NA20513
NA20514 NA20517 NA20521 NA20524 NA20530 NA20531 NA20532 NA20534 NA20535 NA20536
NA20538 NA20539 NA20542 NA20543 NA20544 NA20582 NA20588 NA20589 NA20769 NA20774
NA20800 NA20806 NA20809 NA20813 NA20815 NA20826
1.5 1.0 0.5 0.0
1e−02 1e−01 1e+00 1e+01 1e+02
x

2,4,5−TRICHLOROPHENOL

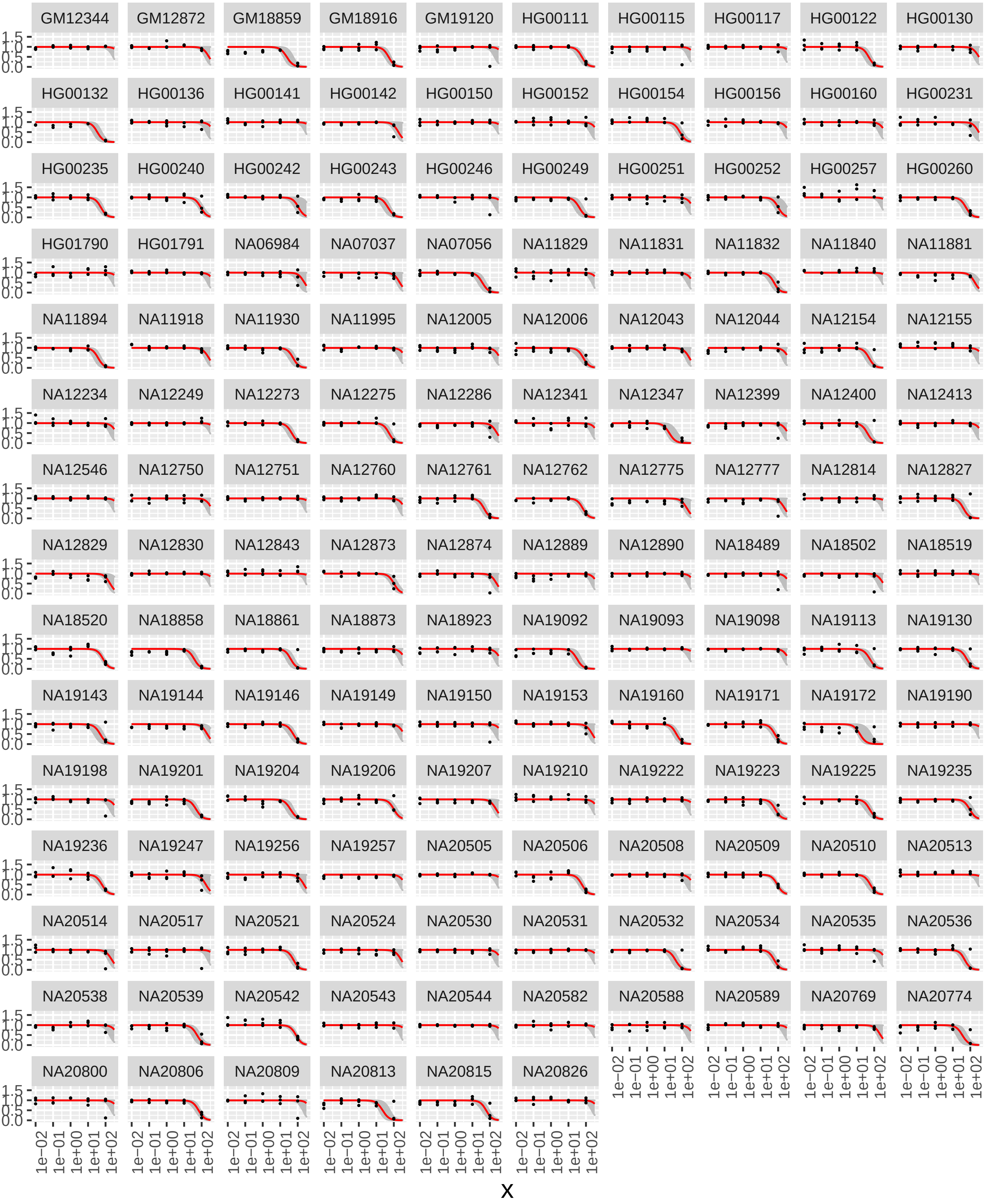

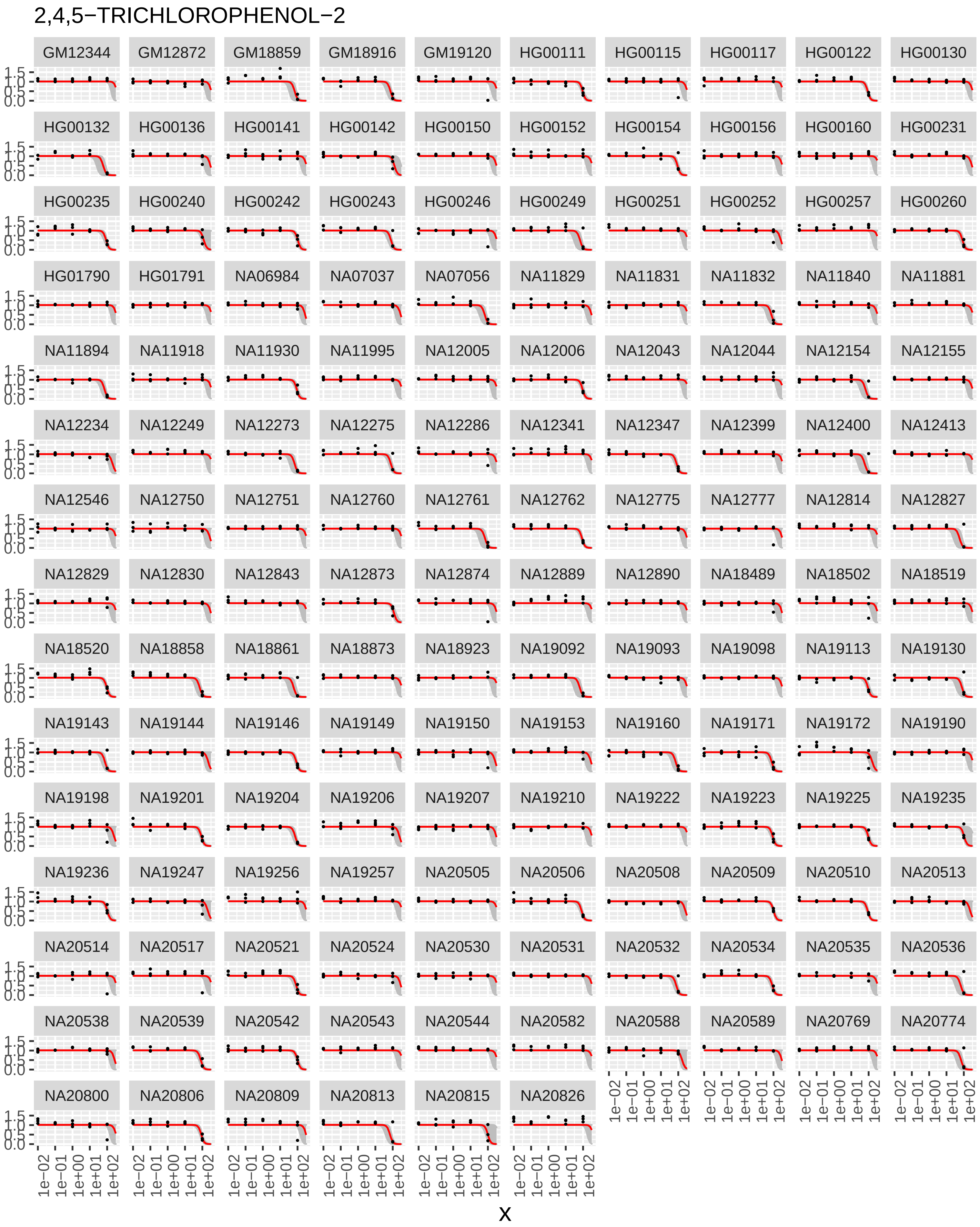
2,4,5−TRICHLOROPHENOL−2
GM12344 GM12872 GM18859 GM18916 GM19120 HG00111 HG00115 HG00117 HG00122 HG00130
HG00132 HG00136 HG00141 HG00142 HG00150 HG00152 HG00154 HG00156 HG00160 HG00231
HG00235 HG00240 HG00242 HG00243 HG00246 HG00249 HG00251 HG00252 HG00257 HG00260
HG01790 HG01791 NA06984 NA07037 NA07056 NA11829 NA11831 NA11832 NA11840 NA11881
NA11894 NA11918 NA11930 NA11995 NA12005 NA12006 NA12043 NA12044 NA12154 NA12155
NA12234 NA12249 NA12273 NA12275 NA12286 NA12341 NA12347 NA12399 NA12400 NA12413
NA12546 NA12750 NA12751 NA12760 NA12761 NA12762 NA12775 NA12777 NA12814 NA12827
NA12829 NA12830 NA12843 NA12873 NA12874 NA12889 NA12890 NA18489 NA18502 NA18519
NA18520 NA18858 NA18861 NA18873 NA18923 NA19092 NA19093 NA19098 NA19113 NA19130
NA19143 NA19144 NA19146 NA19149 NA19150 NA19153 NA19160 NA19171 NA19172 NA19190
NA19198 NA19201 NA19204 NA19206 NA19207 NA19210 NA19222 NA19223 NA19225 NA19235
NA19236 NA19247 NA19256 NA19257 NA20505 NA20506 NA20508 NA20509 NA20510 NA20513
NA20514 NA20517 NA20521 NA20524 NA20530 NA20531 NA20532 NA20534 NA20535 NA20536
NA20538 NA20539 NA20542 NA20543 NA20544 NA20582 NA20588 NA20589 NA20769 NA20774
NA20800 NA20806 NA20809 NA20813 NA20815 NA20826
1.5 1.0 0.5 0.0
1e−02 1e−01 1e+00 1e+01 1e+02
x

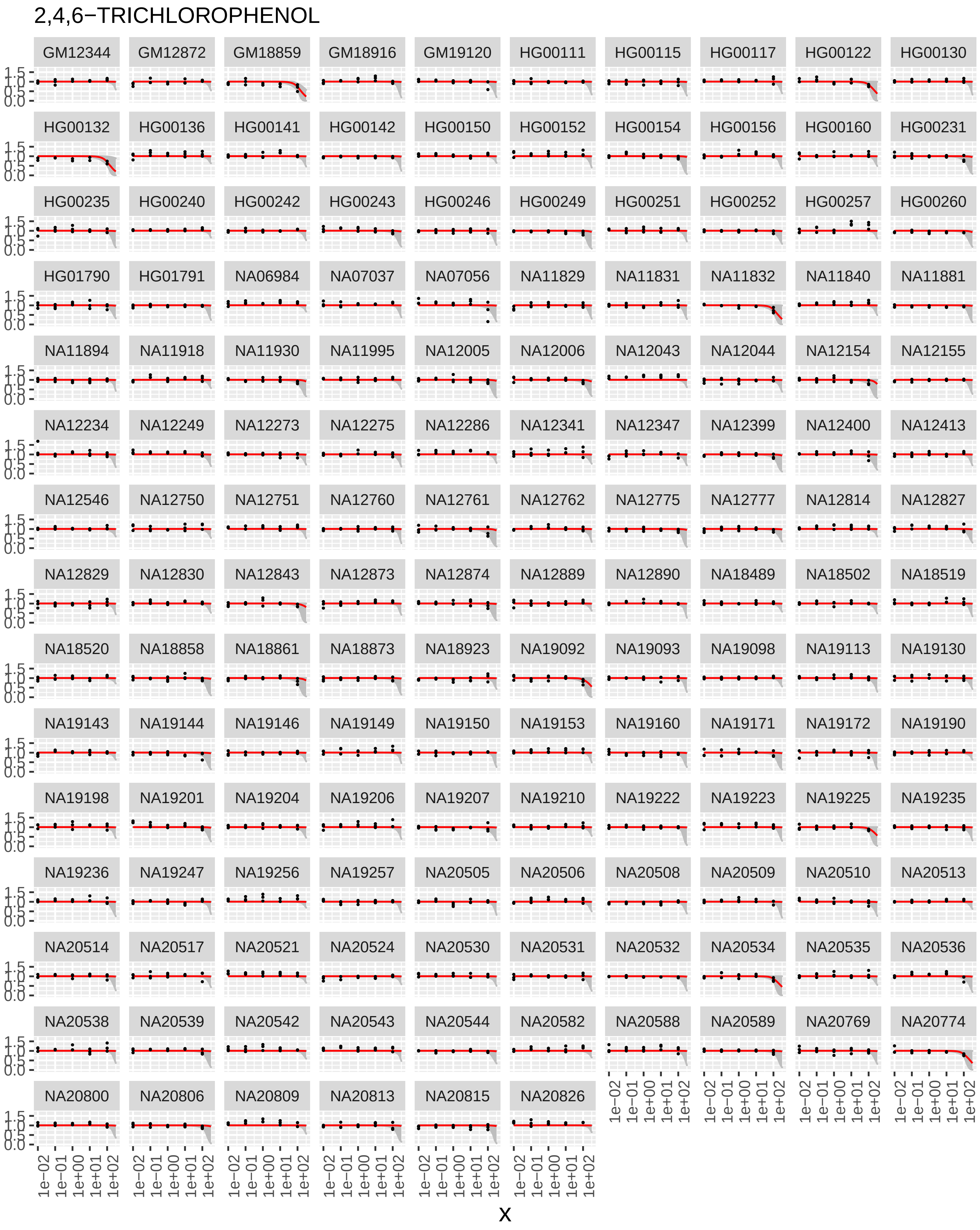
2,4,6−TRICHLOROPHENOL
GM12344
GM12872
GM18859
GM18916
GM19120
HG00111
HG00115
HG00117
HG00122
HG00130
HG00132
HG00136
HG00141
HG00142
HG00150
HG00152
HG00154
HG00156
HG00160
HG00231
HG00235
HG00240
HG00242
HG00243
HG00246
HG00249
HG00251
HG00252
HG00257
HG00260
HG01790
HG01791
NA06984
NA07037
NA07056
NA11829
NA11831
NA11832
NA11840
NA11881
NA11894
NA11918
NA11930
NA11995
NA12005
NA12006
NA12043
NA12044
NA12154
NA12155
NA12234
NA12249
NA12273
NA12275
NA12286
NA12341
NA12347
NA12399
NA12400
NA12413
NA12546
NA12750
NA12751
NA12760
NA12761
NA12762
NA12775
NA12777
NA12814
NA12827
NA12829
NA12830
NA12843
NA12873
NA12874
NA12889
NA12890
NA18489
NA18502
NA18519
NA18520
NA18858
NA18861
NA18873
NA18923
NA19092
NA19093
NA19098
NA19113
NA19130
NA19143
NA19144
NA19146
NA19149
NA19150
NA19153
NA19160
NA19171
NA19172
NA19190
NA19198
NA19201
NA19204
NA19206
NA19207
NA19210
NA19222
NA19223
NA19225
NA19235
NA19236
NA19247
NA19256
NA19257
NA20505
NA20506
NA20508
NA20509
NA20510
NA20513
NA20514
NA20517
NA20521
NA20524
NA20530
NA20531
NA20532
NA20534
NA20535
NA20536
NA20538
NA20539
NA20542
NA20543
NA20544
NA20582
NA20588
NA20589
NA20769
NA20774
NA20800
NA20806
NA20809
NA20813
NA20815
NA20826
1.5
1.0
0.5
0.0
1e−02
1e−01
1e+00
1e+01
1e+02
x

2,4−DINITROPHENOL

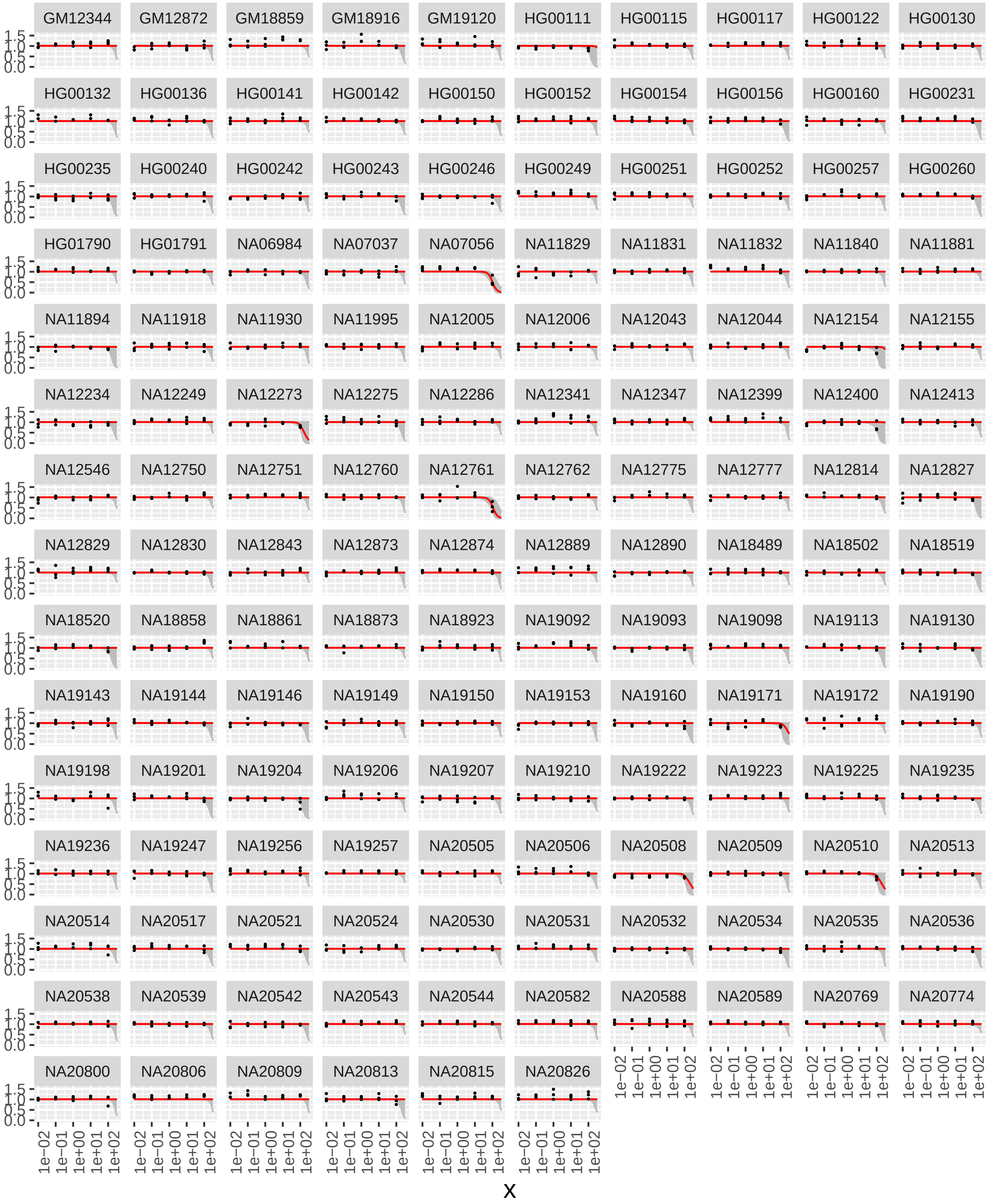

2,4−DINITROTOLUENE

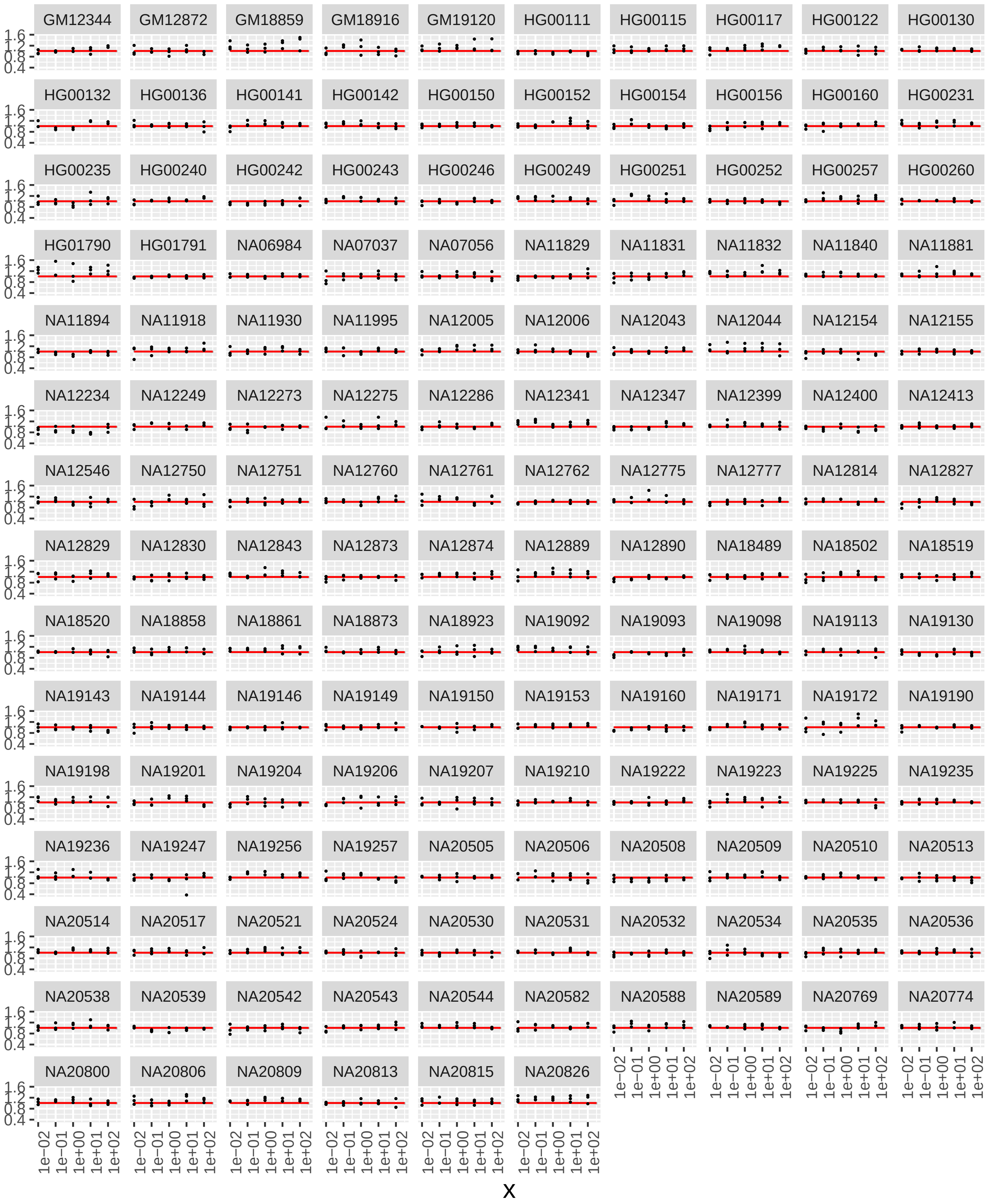

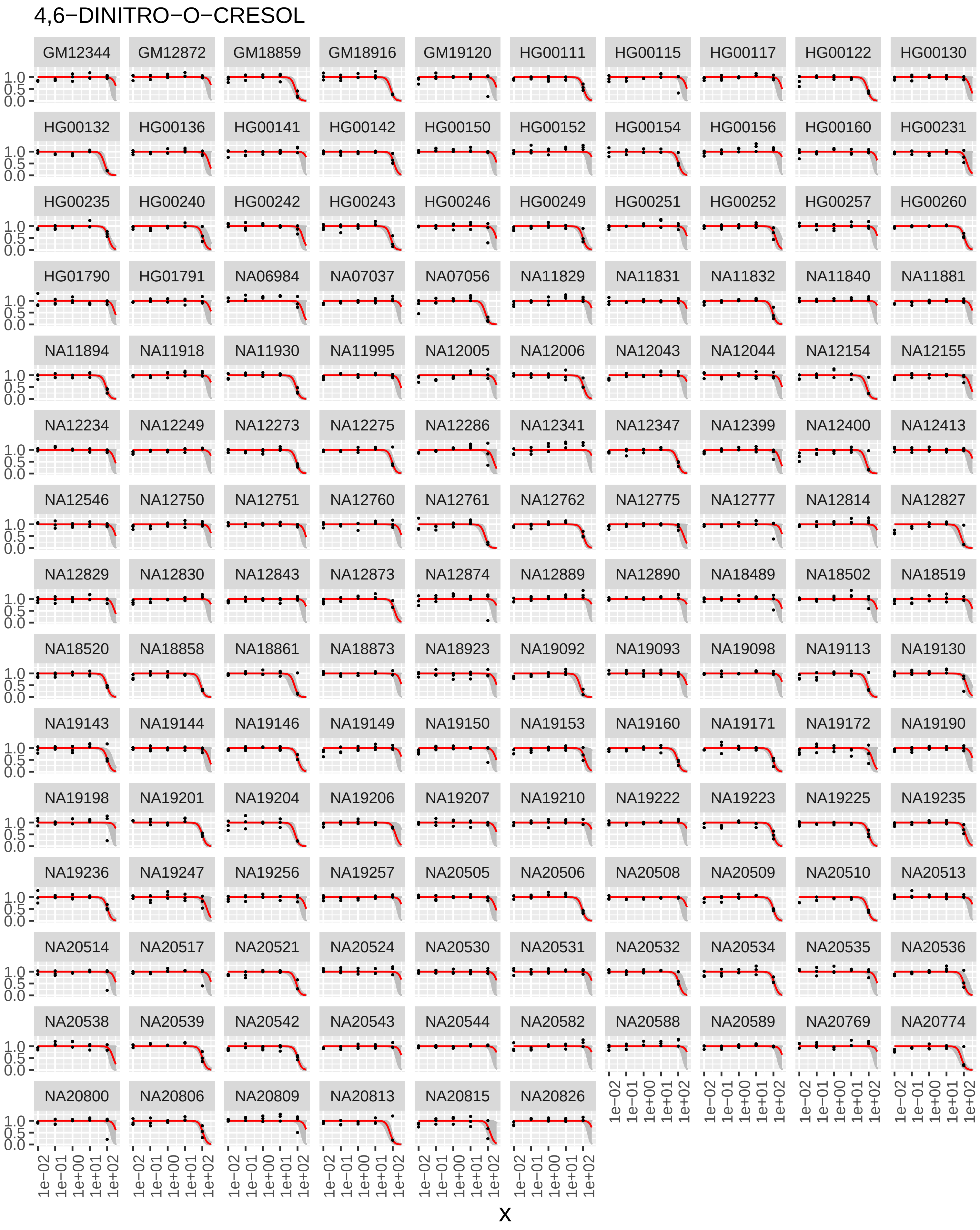
4,6−DINITRO−O−CRESOL
GM12344
GM12872
GM18859
GM18916
GM19120
HG00111
HG00115
HG00117
HG00122
HG00130
HG00132
HG00136
HG00141
HG00142
HG00150
HG00152
HG00154
HG00156
HG00160
HG00231
HG00235
HG00240
HG00242
HG00243
HG00246
HG00249
HG00251
HG00252
HG00257
HG00260
HG01790
HG01791
NA06984
NA07037
NA07056
NA11829
NA11831
NA11832
NA11840
NA11881
NA11894
NA11918
NA11930
NA11995
NA12005
NA12006
NA12043
NA12044
NA12154
NA12155
NA12234
NA12249
NA12273
NA12275
NA12286
NA12341
NA12347
NA12399
NA12400
NA12413
NA12546
NA12750
NA12751
NA12760
NA12761
NA12762
NA12775
NA12777
NA12814
NA12827
NA12829
NA12830
NA12843
NA12873
NA12874
NA12889
NA12890
NA18489
NA18502
NA18519
NA18520
NA18858
NA18861
NA18873
NA18923
NA19092
NA19093
NA19098
NA19113
NA19130
NA19143
NA19144
NA19146
NA19149
NA19150
NA19153
NA19160
NA19171
NA19172
NA19190
NA19198
NA19201
NA19204
NA19206
NA19207
NA19210
NA19222
NA19223
NA19225
NA19235
NA19236
NA19247
NA19256
NA19257
NA20505
NA20506
NA20508
NA20509
NA20510
NA20513
NA20514
NA20517
NA20521
NA20524
NA20530
NA20531
NA20532
NA20534
NA20535
NA20536
NA20538
NA20539
NA20542
NA20543
NA20544
NA20582
NA20588
NA20589
NA20769
NA20774
NA20800
NA20806
NA20809
NA20813
NA20815
NA20826
1.0
0.5
0.0
1e−02
1e−01
1e+00
1e+01
1e+02
x

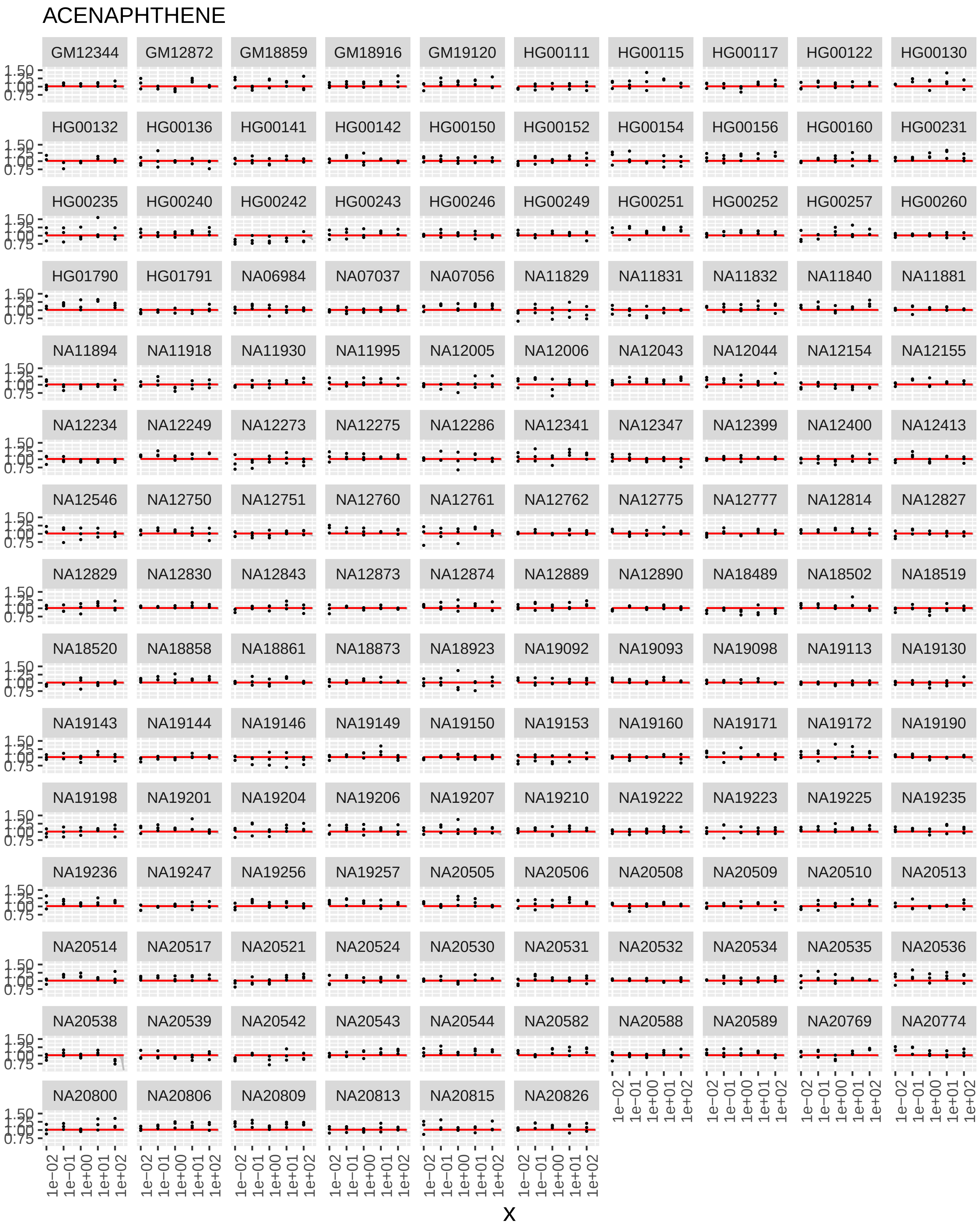
ACENAPHTHENE
GM12344
GM12872
GM18859
GM18916
GM19120
HG00111
HG00115
HG00117
HG00122
HG00130
HG00132
HG00136
HG00141
HG00142
HG00150
HG00152
HG00154
HG00156
HG00160
HG00231
HG00235
HG00240
HG00242
HG00243
HG00246
HG00249
HG00251
HG00252
HG00257
HG00260
HG01790
HG01791
NA06984
NA07037
NA07056
NA11829
NA11831
NA11832
NA11840
NA11881
NA11894
NA11918
NA11930
NA11995
NA12005
NA12006
NA12043
NA12044
NA12154
NA12155
NA12234
NA12249
NA12273
NA12275
NA12286
NA12341
NA12347
NA12399
NA12400
NA12413
NA12546
NA12750
NA12751
NA12760
NA12761
NA12762
NA12775
NA12777
NA12814
NA12827
NA12829
NA12830
NA12843
NA12873
NA12874
NA12889
NA12890
NA18489
NA18502
NA18519
NA18520
NA18858
NA18861
NA18873
NA18923
NA19092
NA19093
NA19098
NA19113
NA19130
NA19143
NA19144
NA19146
NA19149
NA19150
NA19153
NA19160
NA19171
NA19172
NA19190
NA19198
NA19201
NA19204
NA19206
NA19207
NA19210
NA19222
NA19223
NA19225
NA19235
NA19236
NA19247
NA19256
NA19257
NA20505
NA20506
NA20508
NA20509
NA20510
NA20513
NA20514
NA20517
NA20521
NA20524
NA20530
NA20531
NA20532
NA20534
NA20535
NA20536
NA20538
NA20539
NA20542
NA20543
NA20544
NA20582
NA20588
NA20589
NA20769
NA20774
NA20800
NA20806
NA20809
NA20813
NA20815
NA20826
1.50
1.25
1.00
0.75
1e−02
1e−01
1e+00
1e+01
1e+02
x

ALDRIN

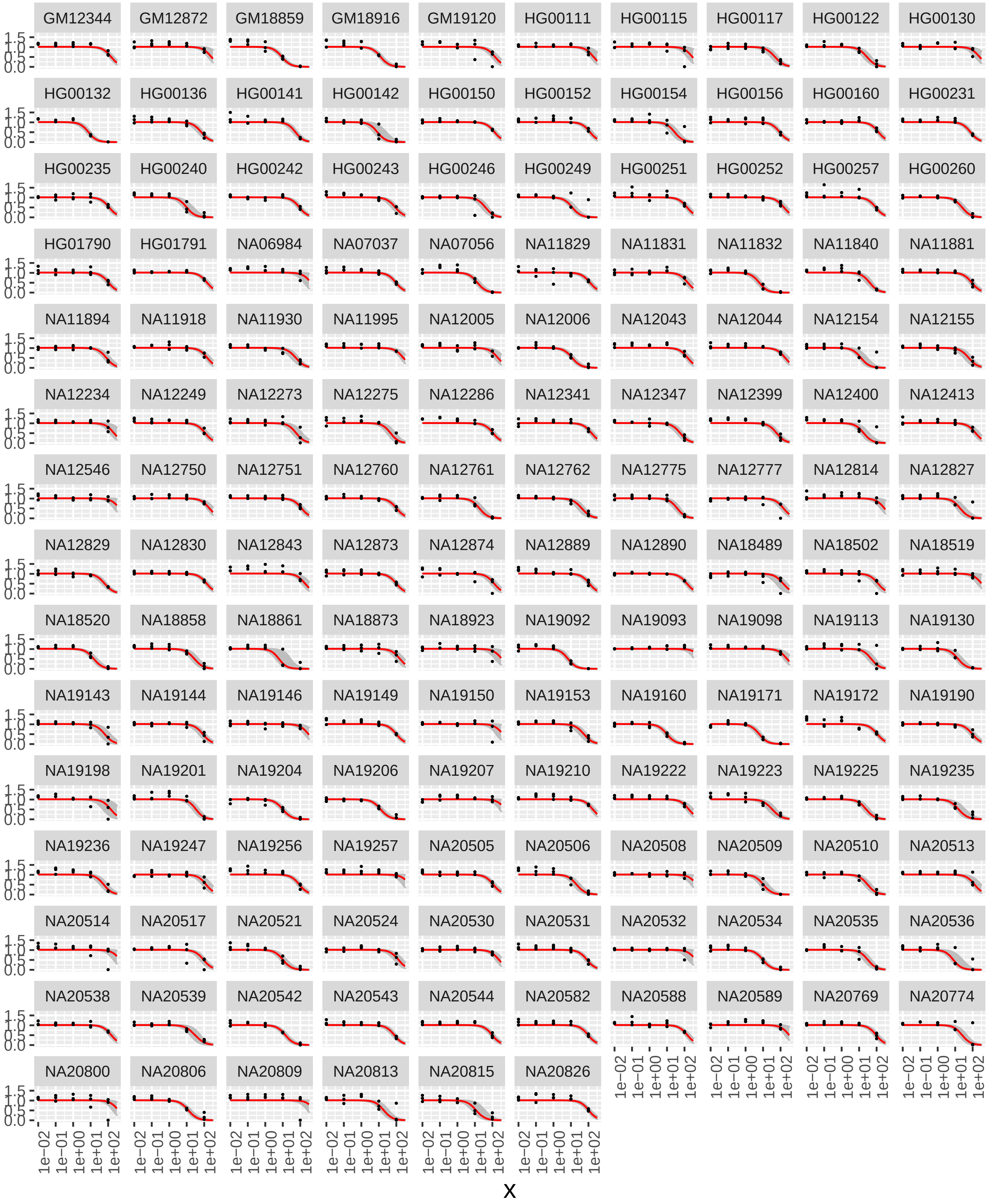
GM12344
GM12872
GM18859
GM18916
GM19120
HG00111
HG00115
HG00117
HG00122
HG00130
HG00132
HG00136
HG00141
HG00142
HG00150
HG00152
HG00154
HG00156
HG00160
HG00231
HG00235
HG00240
HG00242
HG00243
HG00246
HG00249
HG00251
HG00252
HG00257
HG00260
HG01790
HG01791
NA06984
NA07037
NA07056
NA11829
NA11831
NA11832
NA11840
NA11881
NA11894
NA11918
NA11930
NA11995
NA12005
NA12006
NA12043
NA12044
NA12154
NA12155
NA12234
NA12249
NA12273
NA12275
NA12286
NA12341
NA12347
NA12399
NA12400
NA12413
NA12546
NA12750
NA12751
NA12760
NA12761
NA12762
NA12775
NA12777
NA12814
NA12827
NA12829
NA12830
NA12843
NA12873
NA12874
NA12889
NA12890
NA18489
NA18502
NA18519
NA18520
NA18858
NA18861
NA18873
NA18923
NA19092
NA19093
NA19098
NA19113
NA19130
NA19143
NA19144
NA19146
NA19149
NA19150
NA19153
NA19160
NA19171
NA19172
NA19190
NA19198
NA19201
NA19204
NA19206
NA19207
NA19210
NA19222
NA19223
NA19225
NA19235
NA19236
NA19247
NA19256
NA19257
NA20505
NA20506
NA20508
NA20509
NA20510
NA20513
NA20514
NA20517
NA20521
NA20524
NA20530
NA20531
NA20532
NA20534
NA20535
NA20536
NA20538
NA20539
NA20542
NA20543
NA20544
NA20582
NA20588
NA20589
NA20769
NA20774
NA20800
NA20806
NA20809
NA20813
NA20815
NA20826
0.0
0.5
1.0
1.5
1e−02
1e−01
1e+00
1e+01
1e+02
x

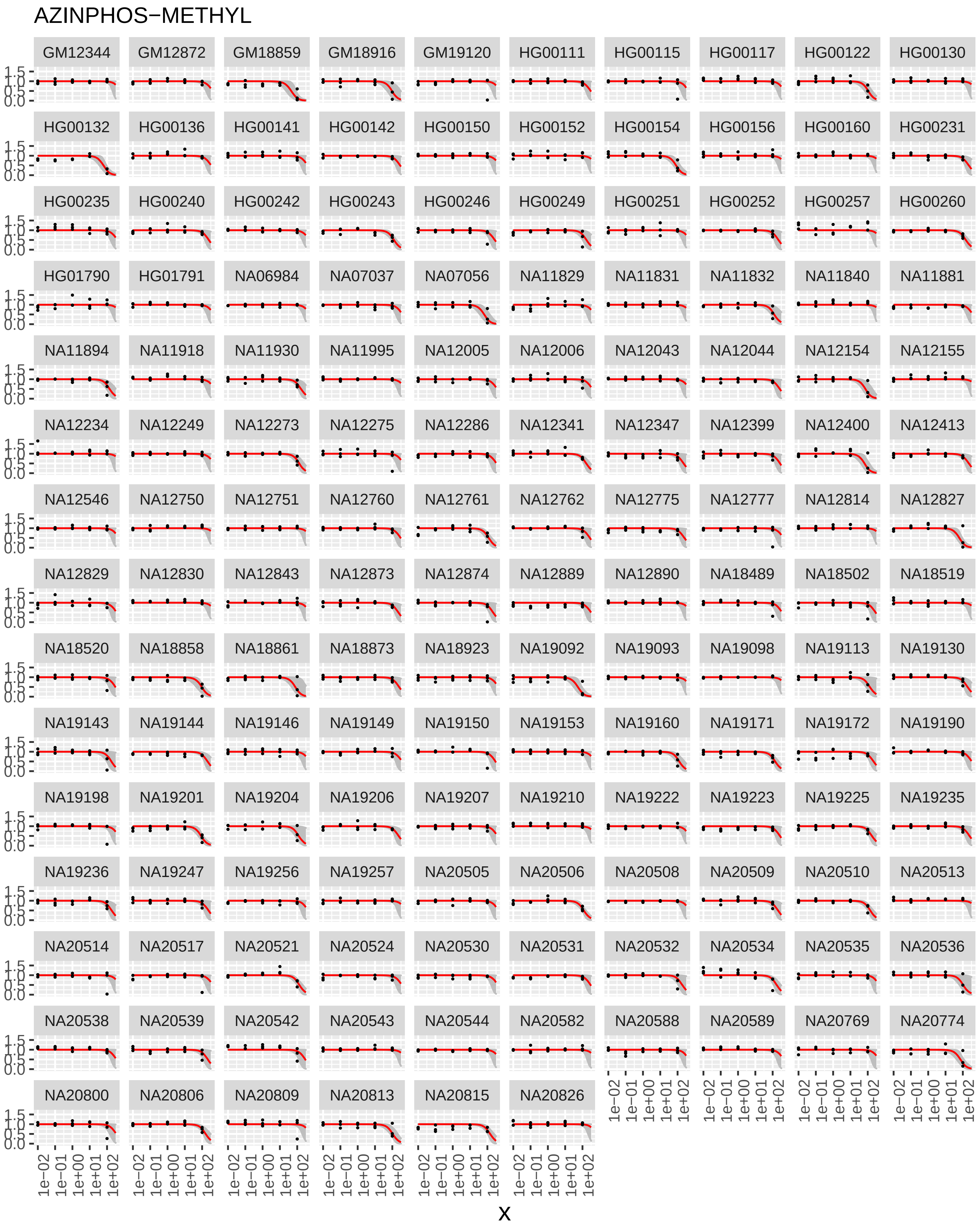
AZINPHOS−METHYL
GM12344 GM12872 GM18859 GM18916 GM19120 HG00111 HG00115 HG00117 HG00122 HG00130
HG00132 HG00136 HG00141 HG00142 HG00150 HG00152 HG00154 HG00156 HG00160 HG00231
HG00235 HG00240 HG00242 HG00243 HG00246 HG00249 HG00251 HG00252 HG00257 HG00260
HG01790 HG01791 NA06984 NA07037 NA07056 NA11829 NA11831 NA11832 NA11840 NA11881
NA11894 NA11918 NA11930 NA11995 NA12005 NA12006 NA12043 NA12044 NA12154 NA12155
NA12234 NA12249 NA12273 NA12275 NA12286 NA12341 NA12347 NA12399 NA12400 NA12413
NA12546 NA12750 NA12751 NA12760 NA12761 NA12762 NA12775 NA12777 NA12814 NA12827
NA12829 NA12830 NA12843 NA12873 NA12874 NA12889 NA12890 NA18489 NA18502 NA18519
NA18520 NA18858 NA18861 NA18873 NA18923 NA19092 NA19093 NA19098 NA19113 NA19130
NA19143 NA19144 NA19146 NA19149 NA19150 NA19153 NA19160 NA19171 NA19172 NA19190
NA19198 NA19201 NA19204 NA19206 NA19207 NA19210 NA19222 NA19223 NA19225 NA19235
NA19236 NA19247 NA19256 NA19257 NA20505 NA20506 NA20508 NA20509 NA20510 NA20513
NA20514 NA20517 NA20521 NA20524 NA20530 NA20531 NA20532 NA20534 NA20535 NA20536
NA20538 NA20539 NA20542 NA20543 NA20544 NA20582 NA20588 NA20589 NA20769 NA20774
NA20800 NA20806 NA20809 NA20813 NA20815 NA20826
1.5
1.0
0.5
0.0
1e−02 1e−01 1e+00 1e+01 1e+02
x

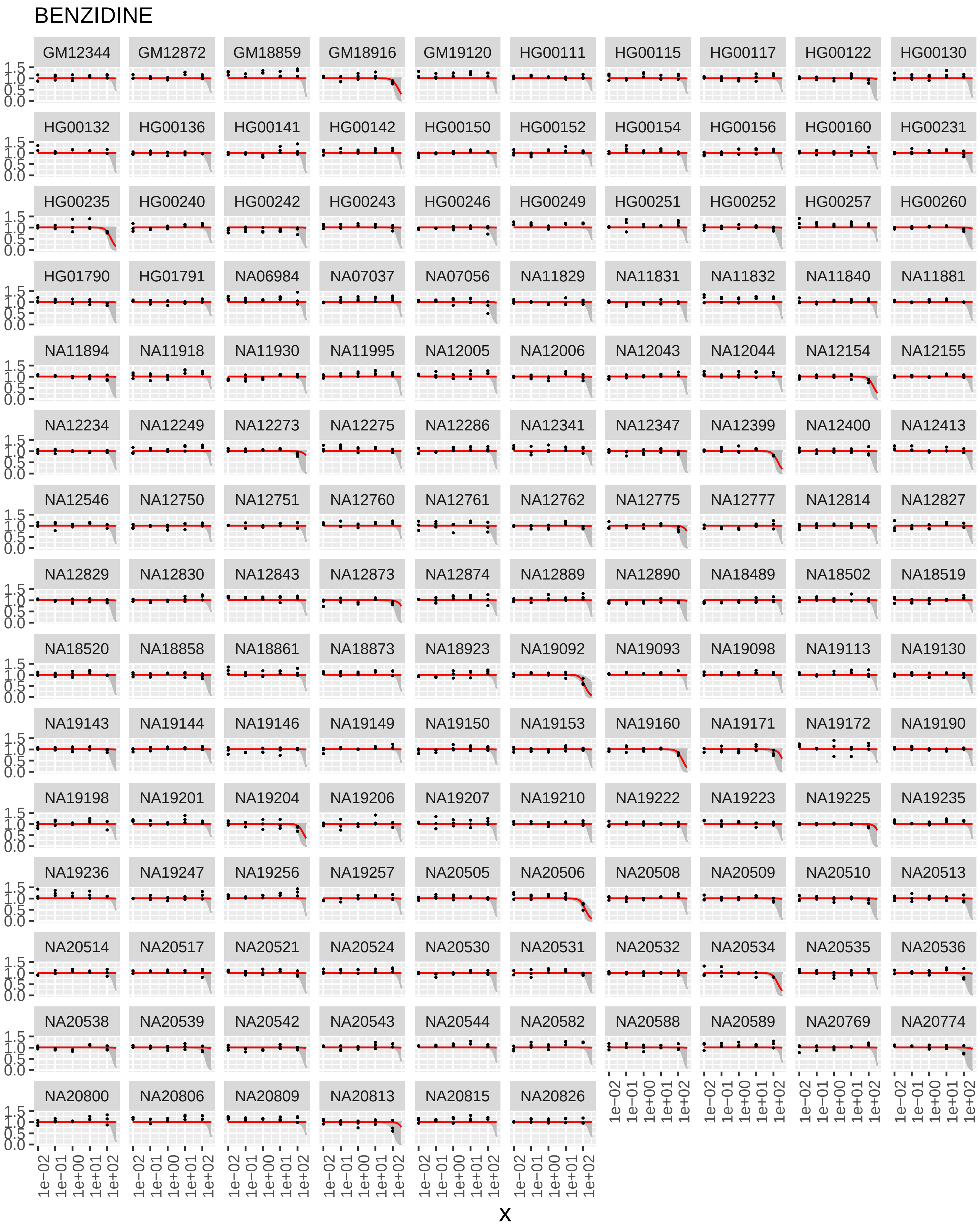
BENZIDINE
GM12344
GM12872
GM18859
GM18916
GM19120
HG00111
HG00115
HG00117
HG00122
HG00130
HG00132
HG00136
HG00141
HG00142
HG00150
HG00152
HG00154
HG00156
HG00160
HG00231
HG00235
HG00240
HG00242
HG00243
HG00246
HG00249
HG00251
HG00252
HG00257
HG00260
HG01790
HG01791
NA06984
NA07037
NA07056
NA11829
NA11831
NA11832
NA11840
NA11881
NA11894
NA11918
NA11930
NA11995
NA12005
NA12006
NA12043
NA12044
NA12154
NA12155
NA12234
NA12249
NA12273
NA12275
NA12286
NA12341
NA12347
NA12399
NA12400
NA12413
NA12546
NA12750
NA12751
NA12760
NA12761
NA12762
NA12775
NA12777
NA12814
NA12827
NA12829
NA12830
NA12843
NA12873
NA12874
NA12889
NA12890
NA18489
NA18502
NA18519
NA18520
NA18858
NA18861
NA18873
NA18923
NA19092
NA19093
NA19098
NA19113
NA19130
NA19143
NA19144
NA19146
NA19149
NA19150
NA19153
NA19160
NA19171
NA19172
NA19190
NA19198
NA19201
NA19204
NA19206
NA19207
NA19210
NA19222
NA19223
NA19225
NA19235
NA19236
NA19247
NA19256
NA19257
NA20505
NA20506
NA20508
NA20509
NA20510
NA20513
NA20514
NA20517
NA20521
NA20524
NA20530
NA20531
NA20532
NA20534
NA20535
NA20536
NA20538
NA20539
NA20542
NA20543
NA20544
NA20582
NA20588
NA20589
NA20769
NA20774
NA20800
NA20806
NA20809
NA20813
NA20815
NA20826
1.5
1.0
0.5
0.0
1e−02
1e−01
1e+00
1e+01
1e+02
x

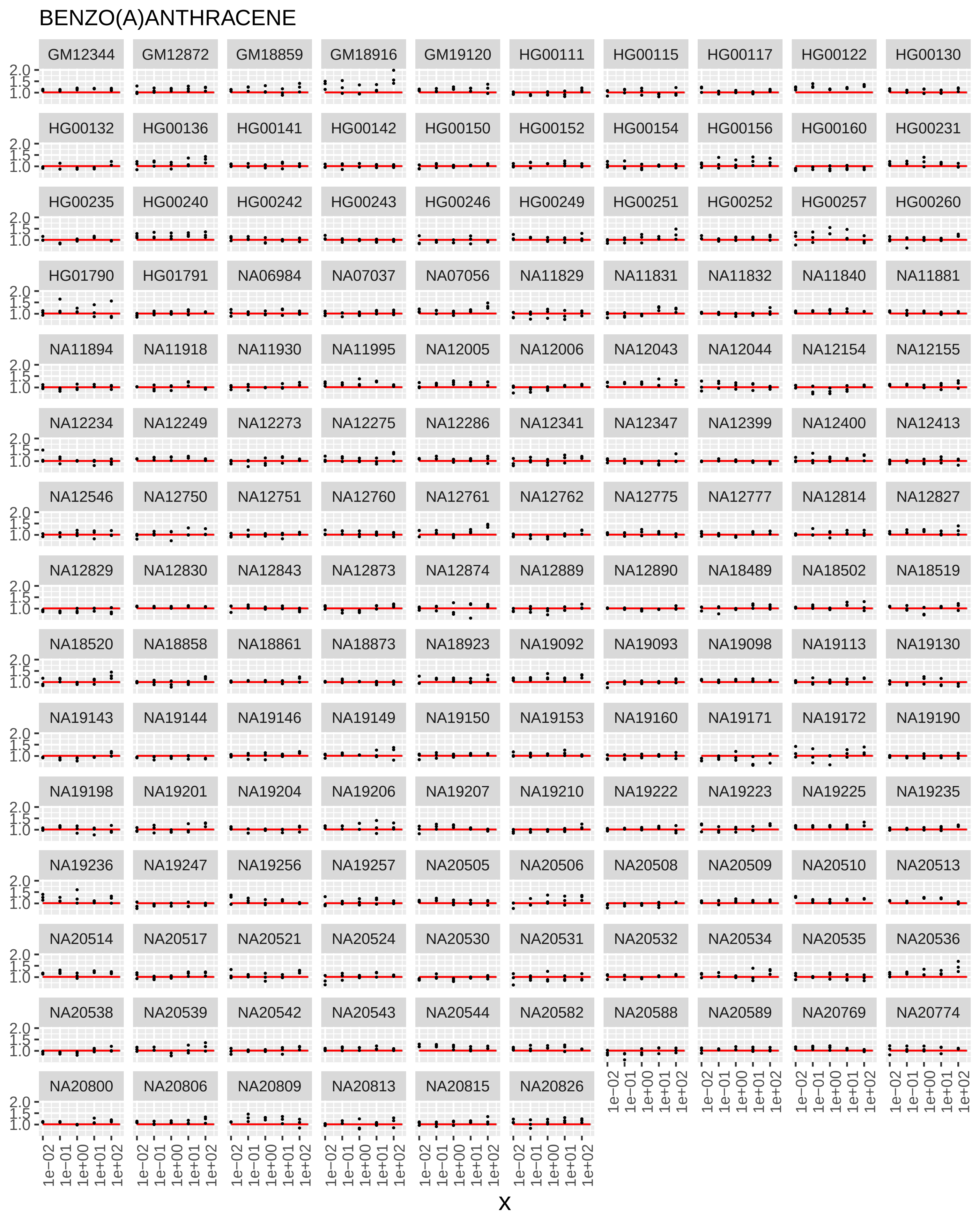
BENZO(A)ANTHRACENE
GM12344
GM12872
GM18859
GM18916
GM19120
HG00111
HG00115
HG00117
HG00122
HG00130
HG00132
HG00136
HG00141
HG00142
HG00150
HG00152
HG00154
HG00156
HG00160
HG00231
HG00235
HG00240
HG00242
HG00243
HG00246
HG00249
HG00251
HG00252
HG00257
HG00260
HG01790
HG01791
NA06984
NA07037
NA07056
NA11829
NA11831
NA11832
NA11840
NA11881
NA11894
NA11918
NA11930
NA11995
NA12005
NA12006
NA12043
NA12044
NA12154
NA12155
NA12234
NA12249
NA12273
NA12275
NA12286
NA12341
NA12347
NA12399
NA12400
NA12413
NA12546
NA12750
NA12751
NA12760
NA12761
NA12762
NA12775
NA12777
NA12814
NA12827
NA12829
NA12830
NA12843
NA12873
NA12874
NA12889
NA12890
NA18489
NA18502
NA18519
NA18520
NA18858
NA18861
NA18873
NA18923
NA19092
NA19093
NA19098
NA19113
NA19130
NA19143
NA19144
NA19146
NA19149
NA19150
NA19153
NA19160
NA19171
NA19172
NA19190
NA19198
NA19201
NA19204
NA19206
NA19207
NA19210
NA19222
NA19223
NA19225
NA19235
NA19236
NA19247
NA19256
NA19257
NA20505
NA20506
NA20508
NA20509
NA20510
NA20513
NA20514
NA20517
NA20521
NA20524
NA20530
NA20531
NA20532
NA20534
NA20535
NA20536
NA20538
NA20539
NA20542
NA20543
NA20544
NA20582
NA20588
NA20589
NA20769
NA20774
NA20800
NA20806
NA20809
NA20813
NA20815
NA20826
2.0
1.5
1.0
1e−02
1e−01
1e+00
1e+01
1e+02
x

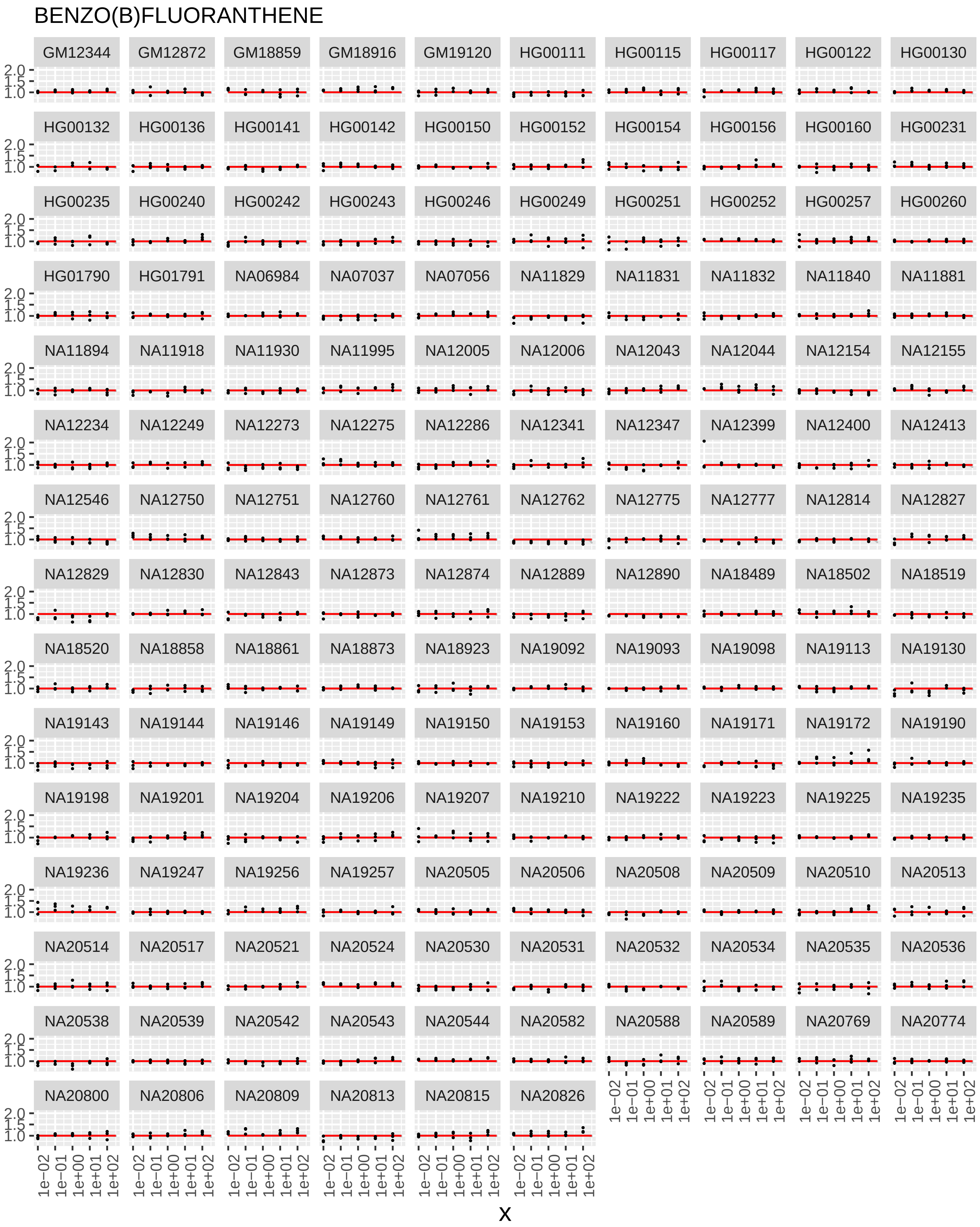
BENZO(B)FLUORANTHENE
GM12344
GM12872
GM18859
GM18916
GM19120
HG00111
HG00115
HG00117
HG00122
HG00130
HG00132
HG00136
HG00141
HG00142
HG00150
HG00152
HG00154
HG00156
HG00160
HG00231
HG00235
HG00240
HG00242
HG00243
HG00246
HG00249
HG00251
HG00252
HG00257
HG00260
HG01790
HG01791
NA06984
NA07037
NA07056
NA11829
NA11831
NA11832
NA11840
NA11881
NA11894
NA11918
NA11930
NA11995
NA12005
NA12006
NA12043
NA12044
NA12154
NA12155
NA12234
NA12249
NA12273
NA12275
NA12286
NA12341
NA12347
NA12399
NA12400
NA12413
NA12546
NA12750
NA12751
NA12760
NA12761
NA12762
NA12775
NA12777
NA12814
NA12827
NA12829
NA12830
NA12843
NA12873
NA12874
NA12889
NA12890
NA18489
NA18502
NA18519
NA18520
NA18858
NA18861
NA18873
NA18923
NA19092
NA19093
NA19098
NA19113
NA19130
NA19143
NA19144
NA19146
NA19149
NA19150
NA19153
NA19160
NA19171
NA19172
NA19190
NA19198
NA19201
NA19204
NA19206
NA19207
NA19210
NA19222
NA19223
NA19225
NA19235
NA19236
NA19247
NA19256
NA19257
NA20505
NA20506
NA20508
NA20509
NA20510
NA20513
NA20514
NA20517
NA20521
NA20524
NA20530
NA20531
NA20532
NA20534
NA20535
NA20536
NA20538
NA20539
NA20542
NA20543
NA20544
NA20582
NA20588
NA20589
NA20769
NA20774
NA20800
NA20806
NA20809
NA20813
NA20815
NA20826
2.0
1.5
1.0
1e−02
1e−01
1e+00
1e+01
1e+02
x

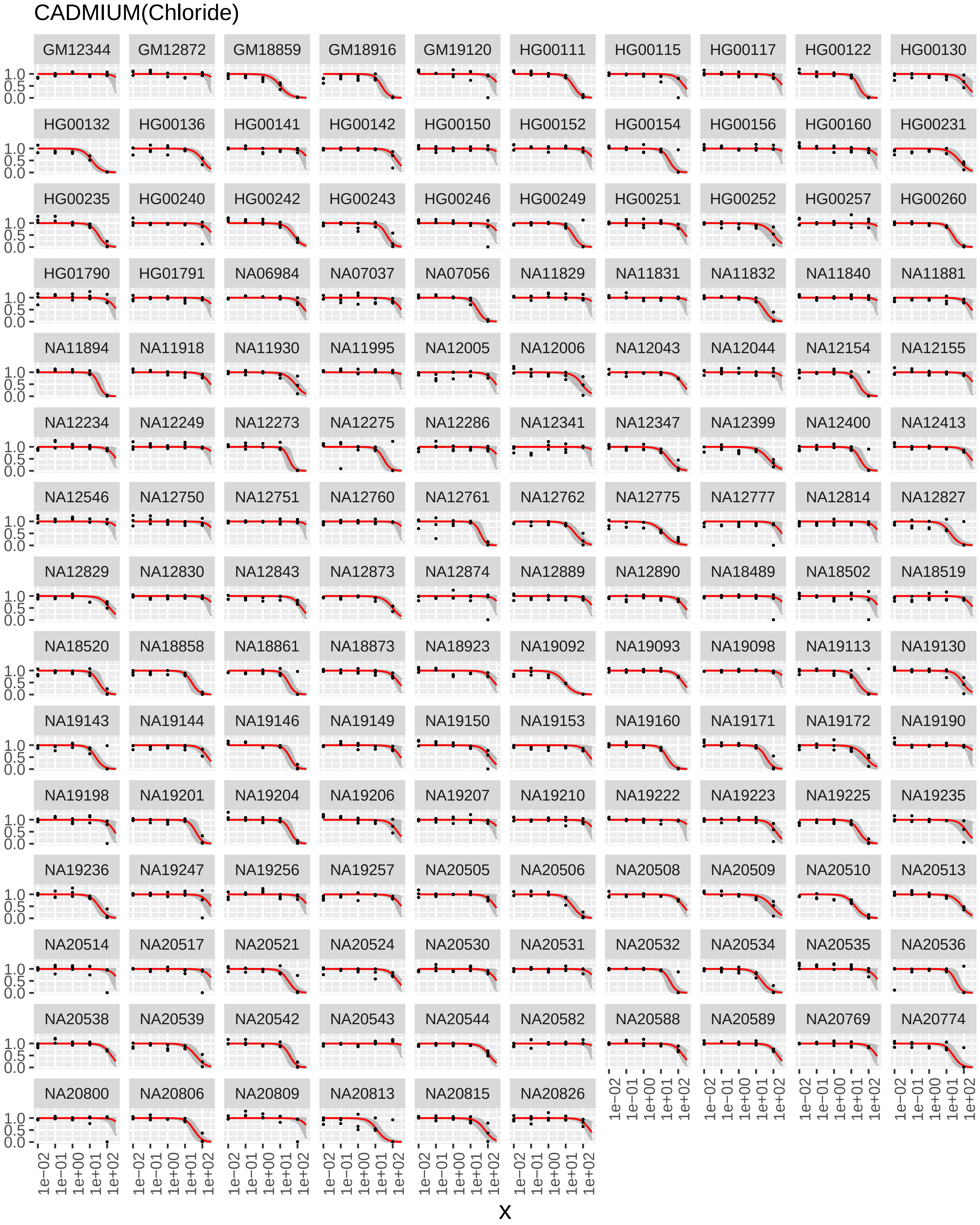
CADMIUM(Chloride)
GM12344
GM12872
GM18859
GM18916
GM19120
HG00111
HG00115
HG00117
HG00122
HG00130
HG00132
HG00136
HG00141
HG00142
HG00150
HG00152
HG00154
HG00156
HG00160
HG00231
HG00235
HG00240
HG00242
HG00243
HG00246
HG00249
HG00251
HG00252
HG00257
HG00260
HG01790
HG01791
NA06984
NA07037
NA07056
NA11829
NA11831
NA11832
NA11840
NA11881
NA11894
NA11918
NA11930
NA11995
NA12005
NA12006
NA12043
NA12044
NA12154
NA12155
NA12234
NA12249
NA12273
NA12275
NA12286
NA12341
NA12347
NA12399
NA12400
NA12413
NA12546
NA12750
NA12751
NA12760
NA12761
NA12762
NA12775
NA12777
NA12814
NA12827
NA12829
NA12830
NA12843
NA12873
NA12874
NA12889
NA12890
NA18489
NA18502
NA18519
NA18520
NA18858
NA18861
NA18873
NA18923
NA19092
NA19093
NA19098
NA19113
NA19130
NA19143
NA19144
NA19146
NA19149
NA19150
NA19153
NA19160
NA19171
NA19172
NA19190
NA19198
NA19201
NA19204
NA19206
NA19207
NA19210
NA19222
NA19223
NA19225
NA19235
NA19236
NA19247
NA19256
NA19257
NA20505
NA20506
NA20508
NA20509
NA20510
NA20513
NA20514
NA20517
NA20521
NA20524
NA20530
NA20531
NA20532
NA20534
NA20535
NA20536
NA20538
NA20539
NA20542
NA20543
NA20544
NA20582
NA20588
NA20589
NA20769
NA20774
NA20800
NA20806
NA20809
NA20813
NA20815
NA20826
1.0
0.5
0.0
1e−02
1e−01
1e+00
1e+01
1e+02
x

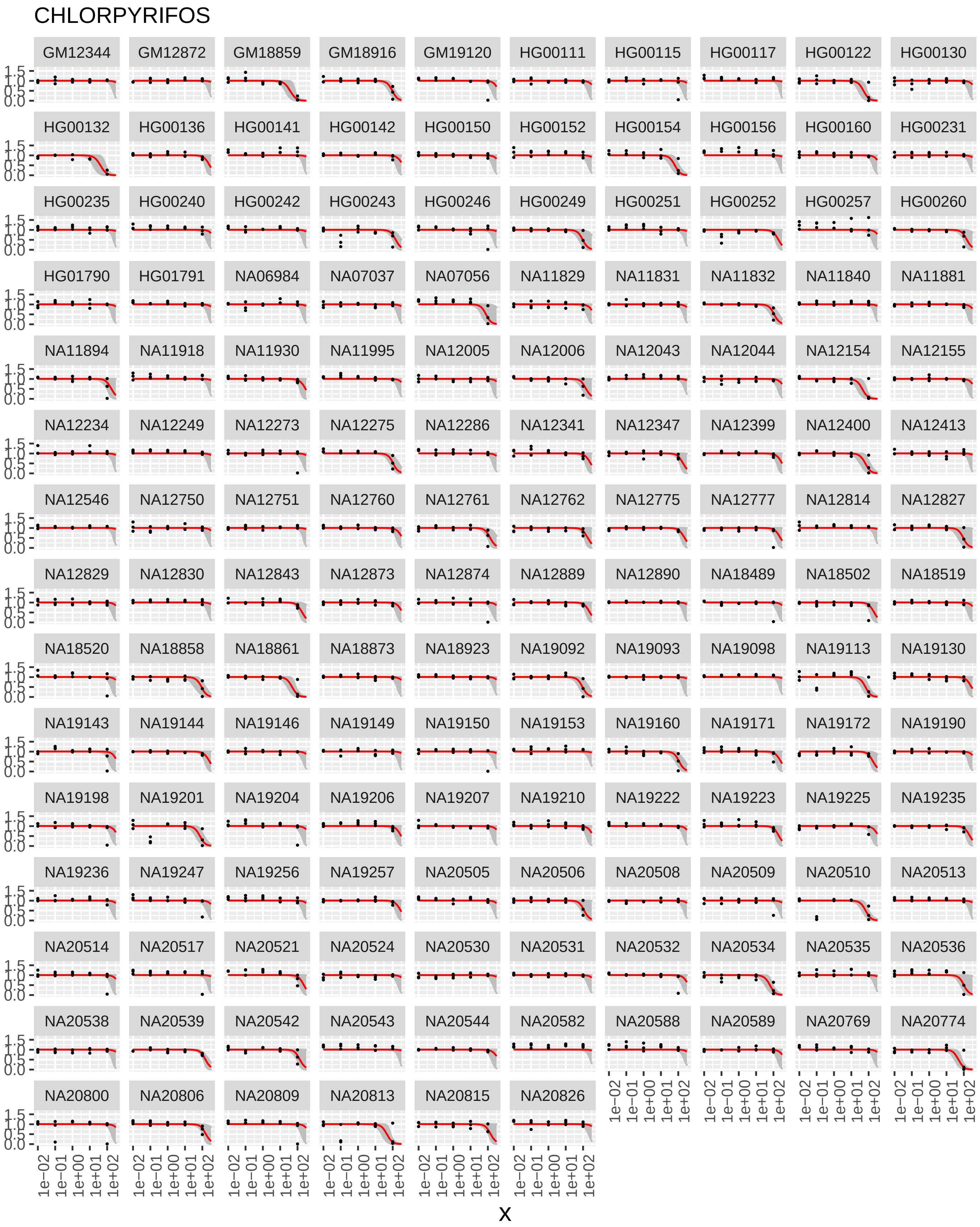

CHLORPYRIFOS
GM12344 GM12872 GM18859 GM18916 GM19120 HG00111 HG00115 HG00117 HG00122 HG00130
HG00132 HG00136 HG00141 HG00142 HG00150 HG00152 HG00154 HG00156 HG00160 HG00231
HG00235 HG00240 HG00242 HG00243 HG00246 HG00249 HG00251 HG00252 HG00257 HG00260
HG01790 HG01791 NA06984 NA07037 NA07056 NA11829 NA11831 NA11832 NA11840 NA11881
NA11894 NA11918 NA11930 NA11995 NA12005 NA12006 NA12043 NA12044 NA12154 NA12155
NA12234 NA12249 NA12273 NA12275 NA12286 NA12341 NA12347 NA12399 NA12400 NA12413
NA12546 NA12750 NA12751 NA12760 NA12761 NA12762 NA12775 NA12777 NA12814 NA12827
NA12829 NA12830 NA12843 NA12873 NA12874 NA12889 NA12890 NA18489 NA18502 NA18519
NA18520 NA18858 NA18861 NA18873 NA18923 NA19092 NA19093 NA19098 NA19113 NA19130
NA19143 NA19144 NA19146 NA19149 NA19150 NA19153 NA19160 NA19171 NA19172 NA19190
NA19198 NA19201 NA19204 NA19206 NA19207 NA19210 NA19222 NA19223 NA19225 NA19235
NA19236 NA19247 NA19256 NA19257 NA20505 NA20506 NA20508 NA20509 NA20510 NA20513
NA20514 NA20517 NA20521 NA20524 NA20530 NA20531 NA20532 NA20534 NA20535 NA20536
NA20538 NA20539 NA20542 NA20543 NA20544 NA20582 NA20588 NA20589 NA20769 NA20774
NA20800 NA20806 NA20809 NA20813 NA20815 NA20826
1.5
1.0
0.5
0.0
1e−02
1e−01
1e+00
1e+01
1e+02
x

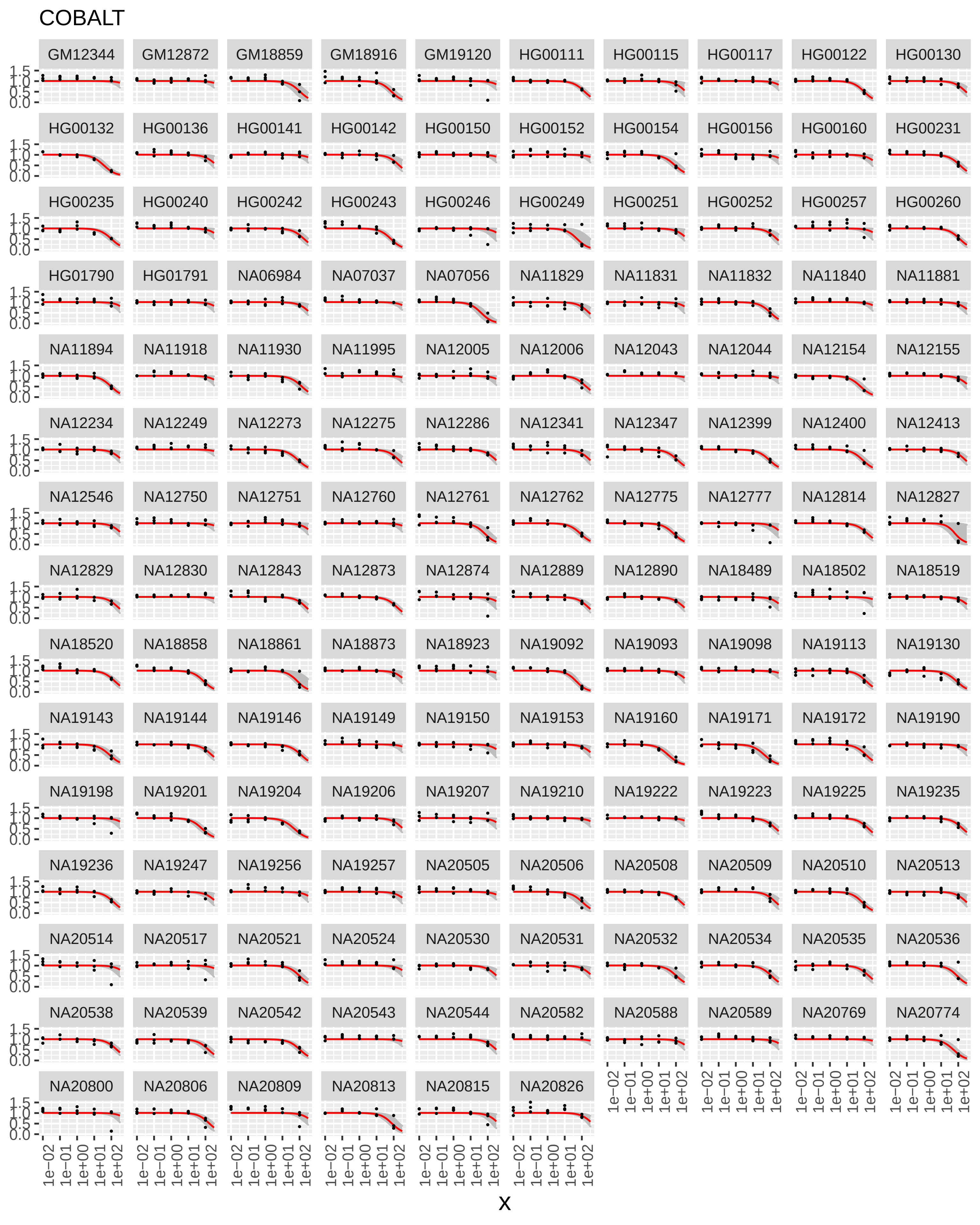

COBALT
GM12344
GM12872
GM18859
GM18916
GM19120
HG00111
HG00115
HG00117
HG00122
HG00130
HG00132
HG00136
HG00141
HG00142
HG00150
HG00152
HG00154
HG00156
HG00160
HG00231
HG00235
HG00240
HG00242
HG00243
HG00246
HG00249
HG00251
HG00252
HG00257
HG00260
HG01790
HG01791
NA06984
NA07037
NA07056
NA11829
NA11831
NA11832
NA11840
NA11881
NA11894
NA11918
NA11930
NA11995
NA12005
NA12006
NA12043
NA12044
NA12154
NA12155
NA12234
NA12249
NA12273
NA12275
NA12286
NA12341
NA12347
NA12399
NA12400
NA12413
NA12546
NA12750
NA12751
NA12760
NA12761
NA12762
NA12775
NA12777
NA12814
NA12827
NA12829
NA12830
NA12843
NA12873
NA12874
NA12889
NA12890
NA18489
NA18502
NA18519
NA18520
NA18858
NA18861
NA18873
NA18923
NA19092
NA19093
NA19098
NA19113
NA19130
NA19143
NA19144
NA19146
NA19149
NA19150
NA19153
NA19160
NA19171
NA19172
NA19190
NA19198
NA19201
NA19204
NA19206
NA19207
NA19210
NA19222
NA19223
NA19225
NA19235
NA19236
NA19247
NA19256
NA19257
NA20505
NA20506
NA20508
NA20509
NA20510
NA20513
NA20514
NA20517
NA20521
NA20524
NA20530
NA20531
NA20532
NA20534
NA20535
NA20536
NA20538
NA20539
NA20542
NA20543
NA20544
NA20582
NA20588
NA20589
NA20769
NA20774
NA20800
NA20806
NA20809
NA20813
NA20815
NA20826
1.5
1.0
0.5
0.0
1e−02
1e−01
1e+00
1e+01
1e+02
x

CRESOL, PARA−

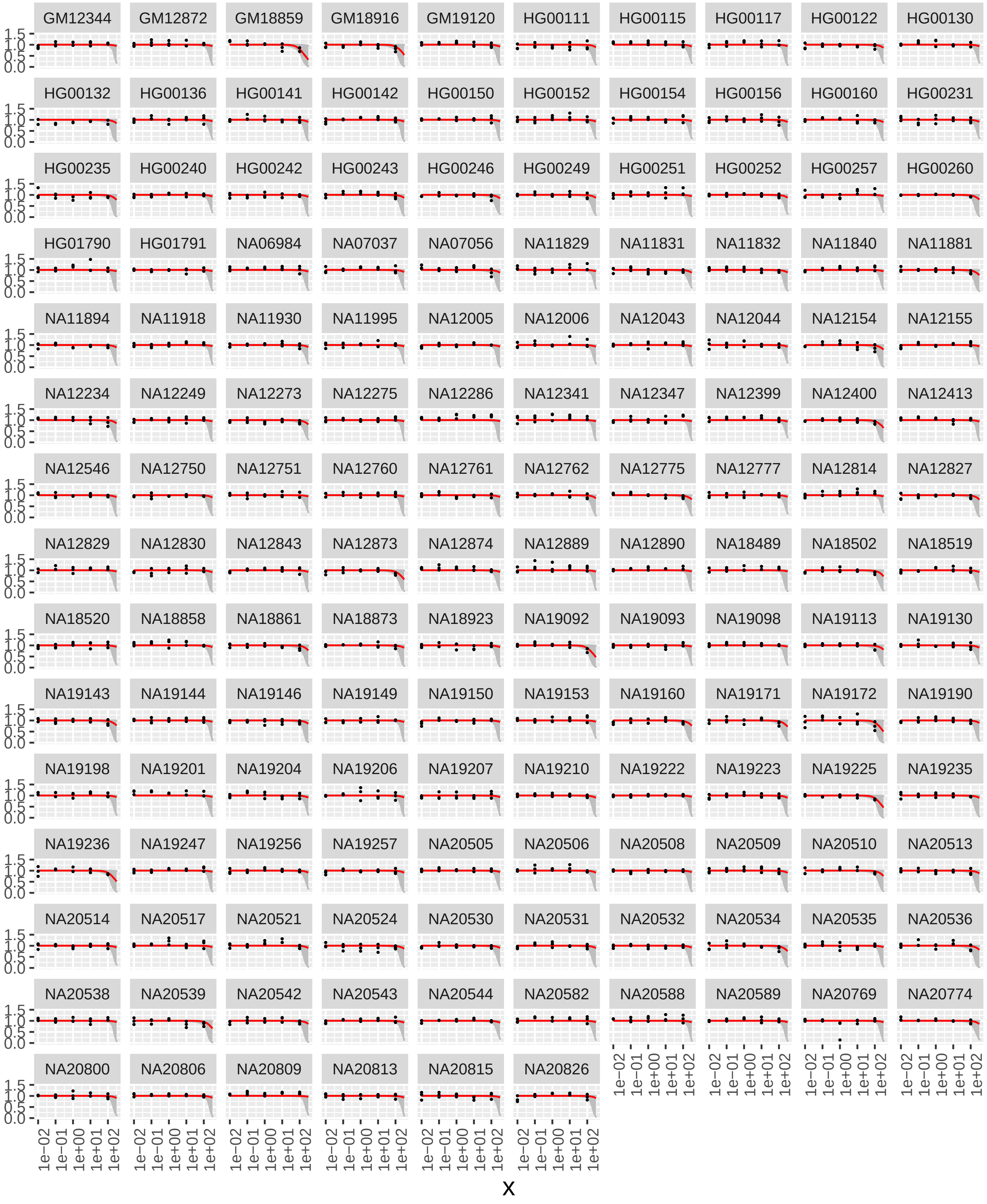
GM12344
GM12872
GM18859
GM18916
GM19120
HG00111
HG00115
HG00117
HG00122
HG00130
HG00132
HG00136
HG00141
HG00142
HG00150
HG00152
HG00154
HG00156
HG00160
HG00231
HG00235
HG00240
HG00242
HG00243
HG00246
HG00249
HG00251
HG00252
HG00257
HG00260
HG01790
HG01791
NA06984
NA07037
NA07056
NA11829
NA11831
NA11832
NA11840
NA11881
NA11894
NA11918
NA11930
NA11995
NA12005
NA12006
NA12043
NA12044
NA12154
NA12155
NA12234
NA12249
NA12273
NA12275
NA12286
NA12341
NA12347
NA12399
NA12400
NA12413
NA12546
NA12750
NA12751
NA12760
NA12761
NA12762
NA12775
NA12777
NA12814
NA12827
NA12829
NA12830
NA12843
NA12873
NA12874
NA12889
NA12890
NA18489
NA18502
NA18519
NA18520
NA18858
NA18861
NA18873
NA18923
NA19092
NA19093
NA19098
NA19113
NA19130
NA19143
NA19144
NA19146
NA19149
NA19150
NA19153
NA19160
NA19171
NA19172
NA19190
NA19198
NA19201
NA19204
NA19206
NA19207
NA19210
NA19222
NA19223
NA19225
NA19235
NA19236
NA19247
NA19256
NA19257
NA20505
NA20506
NA20508
NA20509
NA20510
NA20513
NA20514
NA20517
NA20521
NA20524
NA20530
NA20531
NA20532
NA20534
NA20535
NA20536
NA20538
NA20539
NA20542
NA20543
NA20544
NA20582
NA20588
NA20589
NA20769
NA20774
NA20800
NA20806
NA20809
NA20813
NA20815
NA20826
1.5
1.0
0.5
0.0
1e−02
1e−01
1e+00
1e+01
1e+02
x

DDD, P,P'−

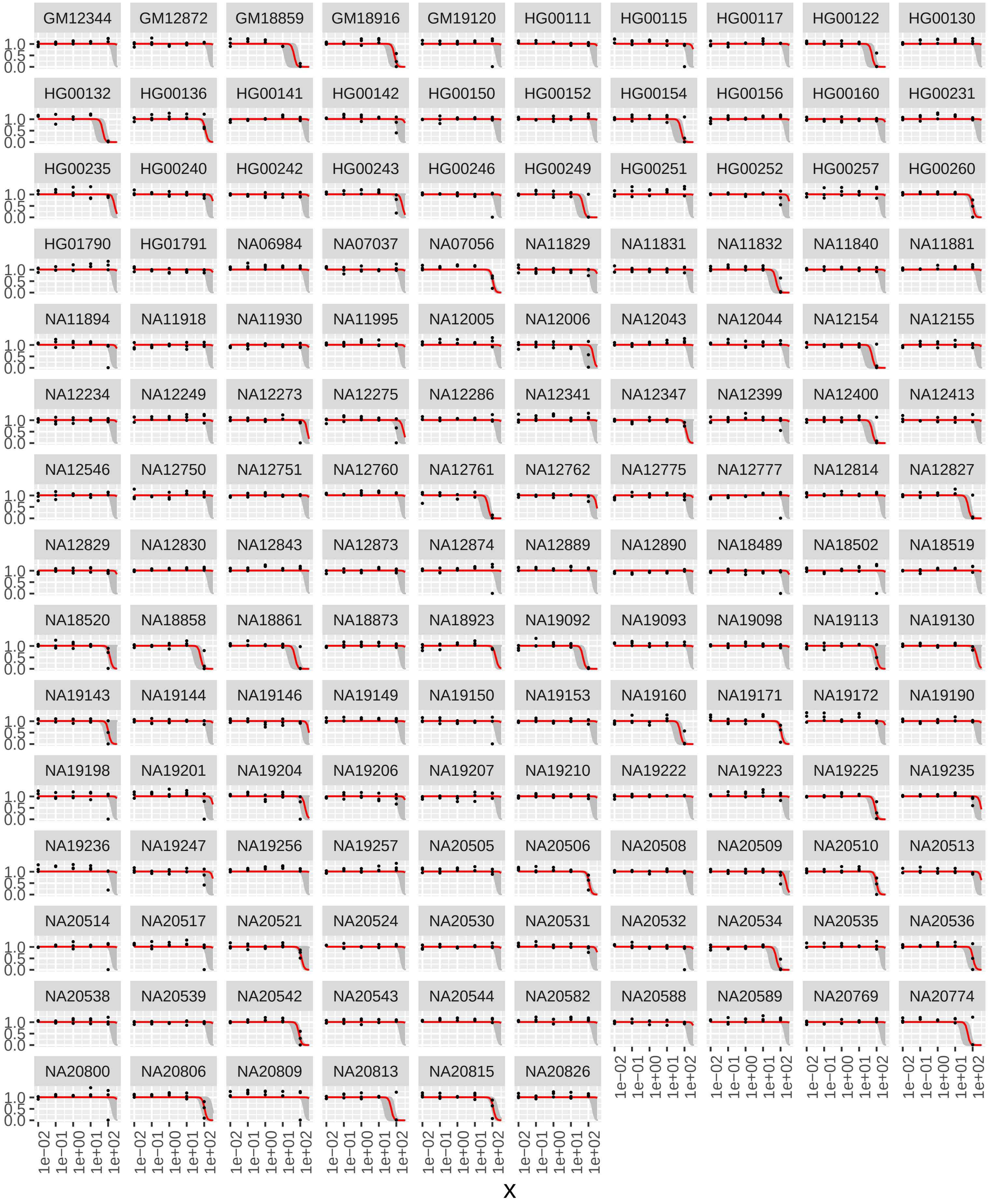
GM12344
GM12872
GM18859
GM18916
GM19120
HG00111
HG00115
HG00117
HG00122
HG00130
HG00132
HG00136
HG00141
HG00142
HG00150
HG00152
HG00154
HG00156
HG00160
HG00231
HG00235
HG00240
HG00242
HG00243
HG00246
HG00249
HG00251
HG00252
HG00257
HG00260
HG01790
HG01791
NA06984
NA07037
NA07056
NA11829
NA11831
NA11832
NA11840
NA11881
NA11894
NA11918
NA11930
NA11995
NA12005
NA12006
NA12043
NA12044
NA12154
NA12155
NA12234
NA12249
NA12273
NA12275
NA12286
NA12341
NA12347
NA12399
NA12400
NA12413
NA12546
NA12750
NA12751
NA12760
NA12761
NA12762
NA12775
NA12777
NA12814
NA12827
NA12829
NA12830
NA12843
NA12873
NA12874
NA12889
NA12890
NA18489
NA18502
NA18519
NA18520
NA18858
NA18861
NA18873
NA18923
NA19092
NA19093
NA19098
NA19113
NA19130
NA19143
NA19144
NA19146
NA19149
NA19150
NA19153
NA19160
NA19171
NA19172
NA19190
NA19198
NA19201
NA19204
NA19206
NA19207
NA19210
NA19222
NA19223
NA19225
NA19235
NA19236
NA19247
NA19256
NA19257
NA20505
NA20506
NA20508
NA20509
NA20510
NA20513
NA20514
NA20517
NA20521
NA20524
NA20530
NA20531
NA20532
NA20534
NA20535
NA20536
NA20538
NA20539
NA20542
NA20543
NA20544
NA20582
NA20588
NA20589
NA20769
NA20774
NA20800
NA20806
NA20809
NA20813
NA20815
NA20826
1.0
0.5
0.0
1e−02
1e−01
1e+00
1e+01
1e+02
x

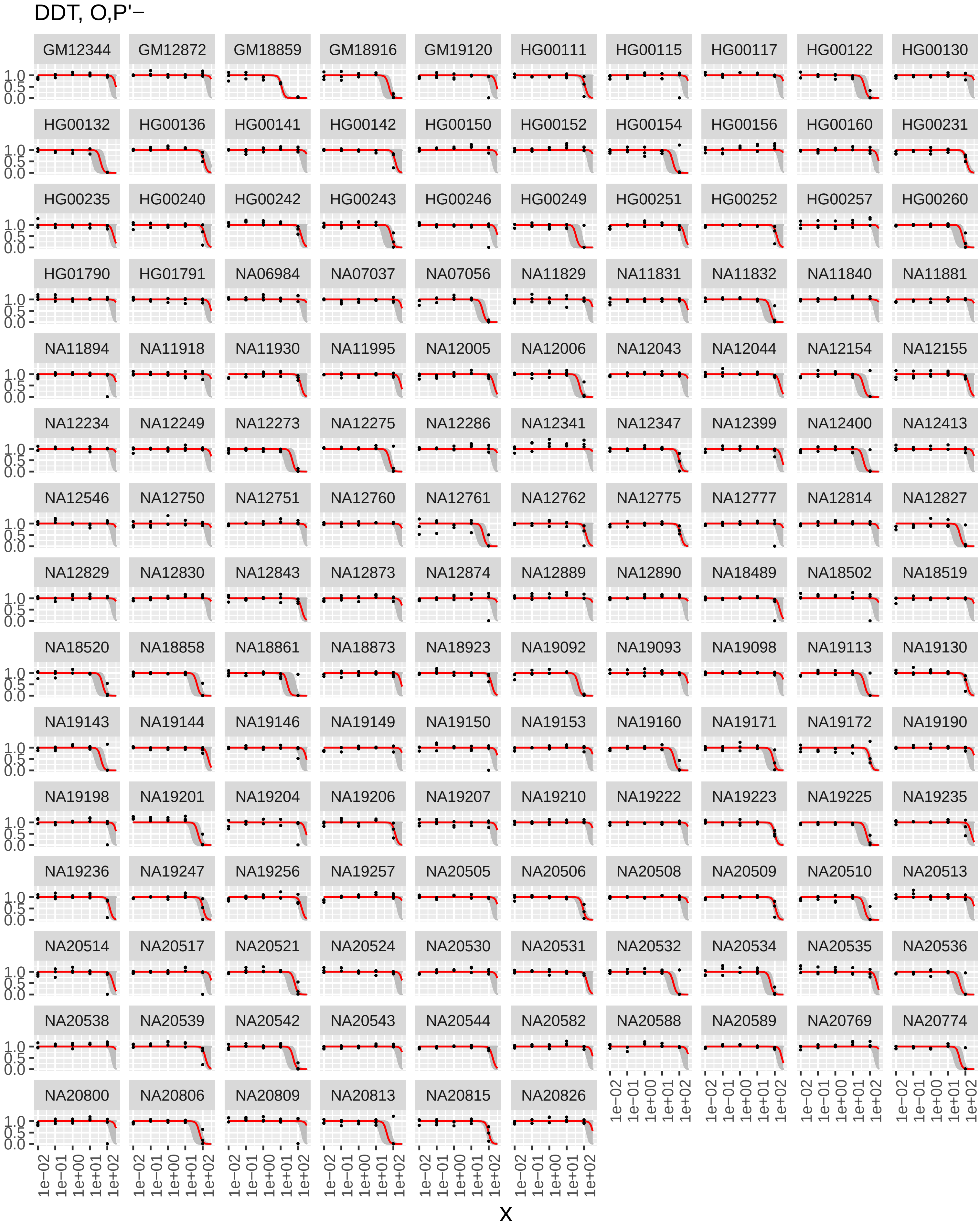
DDT, O,P'−
GM12344 GM12872 GM18859 GM18916 GM19120 HG00111 HG00115 HG00117 HG00122 HG00130
HG00132 HG00136 HG00141 HG00142 HG00150 HG00152 HG00154 HG00156 HG00160 HG00231
HG00235 HG00240 HG00242 HG00243 HG00246 HG00249 HG00251 HG00252 HG00257 HG00260
HG01790 HG01791 NA06984 NA07037 NA07056 NA11829 NA11831 NA11832 NA11840 NA11881
NA11894 NA11918 NA11930 NA11995 NA12005 NA12006 NA12043 NA12044 NA12154 NA12155
NA12234 NA12249 NA12273 NA12275 NA12286 NA12341 NA12347 NA12399 NA12400 NA12413
NA12546 NA12750 NA12751 NA12760 NA12761 NA12762 NA12775 NA12777 NA12814 NA12827
NA12829 NA12830 NA12843 NA12873 NA12874 NA12889 NA12890 NA18489 NA18502 NA18519
NA18520 NA18858 NA18861 NA18873 NA18923 NA19092 NA19093 NA19098 NA19113 NA19130
NA19143 NA19144 NA19146 NA19149 NA19150 NA19153 NA19160 NA19171 NA19172 NA19190
NA19198 NA19201 NA19204 NA19206 NA19207 NA19210 NA19222 NA19223 NA19225 NA19235
NA19236 NA19247 NA19256 NA19257 NA20505 NA20506 NA20508 NA20509 NA20510 NA20513
NA20514 NA20517 NA20521 NA20524 NA20530 NA20531 NA20532 NA20534 NA20535 NA20536
NA20538 NA20539 NA20542 NA20543 NA20544 NA20582 NA20588 NA20589 NA20769 NA20774
NA20800 NA20806 NA20809 NA20813 NA20815 NA20826
0.0
0.5
1.0
1e−02
1e−01
1e+00
1e+01
1e+02
x

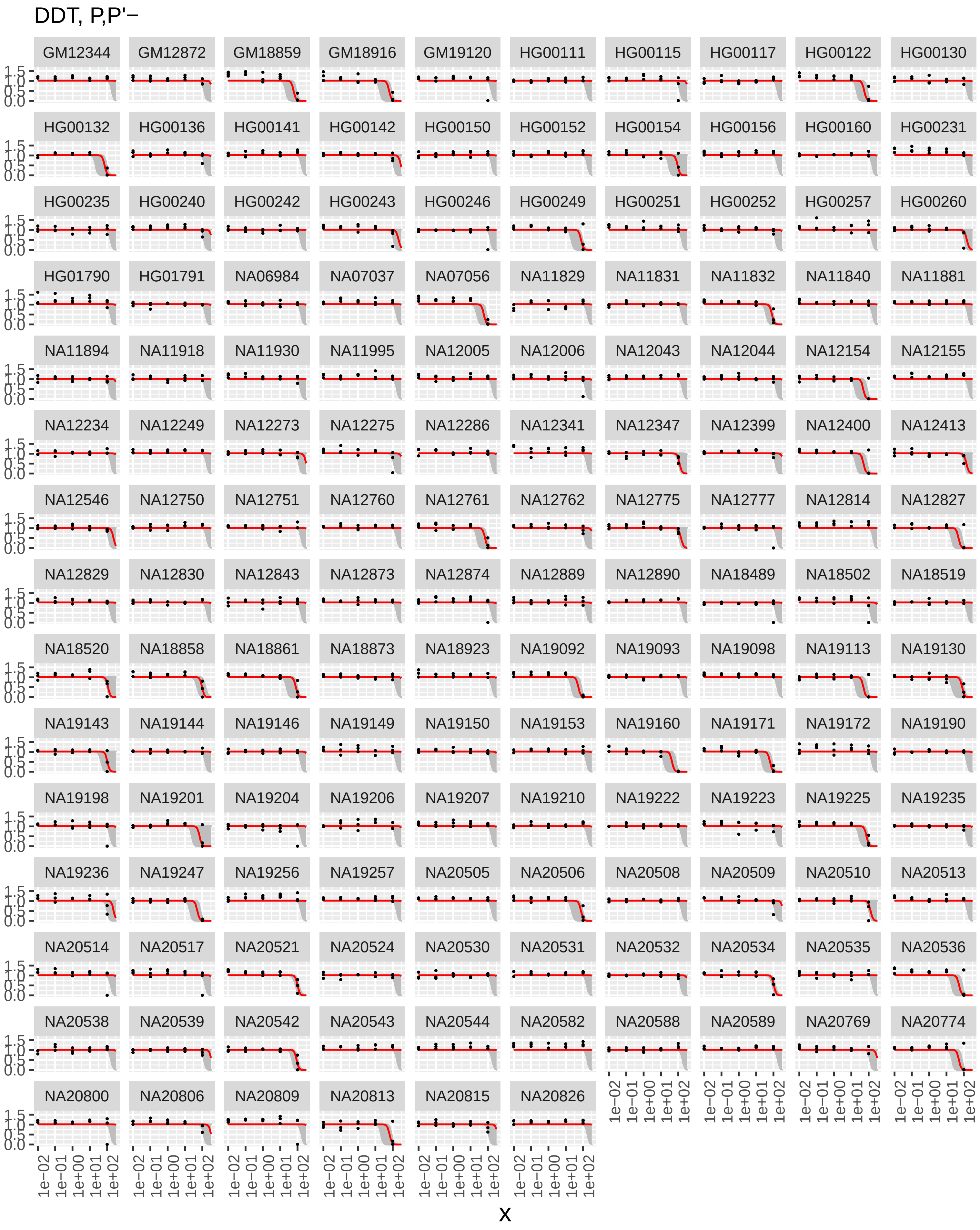
DDT, P,P'−
GM12344
GM12872
GM18859
GM18916
GM19120
HG00111
HG00115
HG00117
HG00122
HG00130
HG00132
HG00136
HG00141
HG00142
HG00150
HG00152
HG00154
HG00156
HG00160
HG00231
HG00235
HG00240
HG00242
HG00243
HG00246
HG00249
HG00251
HG00252
HG00257
HG00260
HG01790
HG01791
NA06984
NA07037
NA07056
NA11829
NA11831
NA11832
NA11840
NA11881
NA11894
NA11918
NA11930
NA11995
NA12005
NA12006
NA12043
NA12044
NA12154
NA12155
NA12234
NA12249
NA12273
NA12275
NA12286
NA12341
NA12347
NA12399
NA12400
NA12413
NA12546
NA12750
NA12751
NA12760
NA12761
NA12762
NA12775
NA12777
NA12814
NA12827
NA12829
NA12830
NA12843
NA12873
NA12874
NA12889
NA12890
NA18489
NA18502
NA18519
NA18520
NA18858
NA18861
NA18873
NA18923
NA19092
NA19093
NA19098
NA19113
NA19130
NA19143
NA19144
NA19146
NA19149
NA19150
NA19153
NA19160
NA19171
NA19172
NA19190
NA19198
NA19201
NA19204
NA19206
NA19207
NA19210
NA19222
NA19223
NA19225
NA19235
NA19236
NA19247
NA19256
NA19257
NA20505
NA20506
NA20508
NA20509
NA20510
NA20513
NA20514
NA20517
NA20521
NA20524
NA20530
NA20531
NA20532
NA20534
NA20535
NA20536
NA20538
NA20539
NA20542
NA20543
NA20544
NA20582
NA20588
NA20589
NA20769
NA20774
NA20800
NA20806
NA20809
NA20813
NA20815
NA20826
1.5
1.0
0.5
0.0
1e−02
1e−01
1e+00
1e+01
1e+02
x

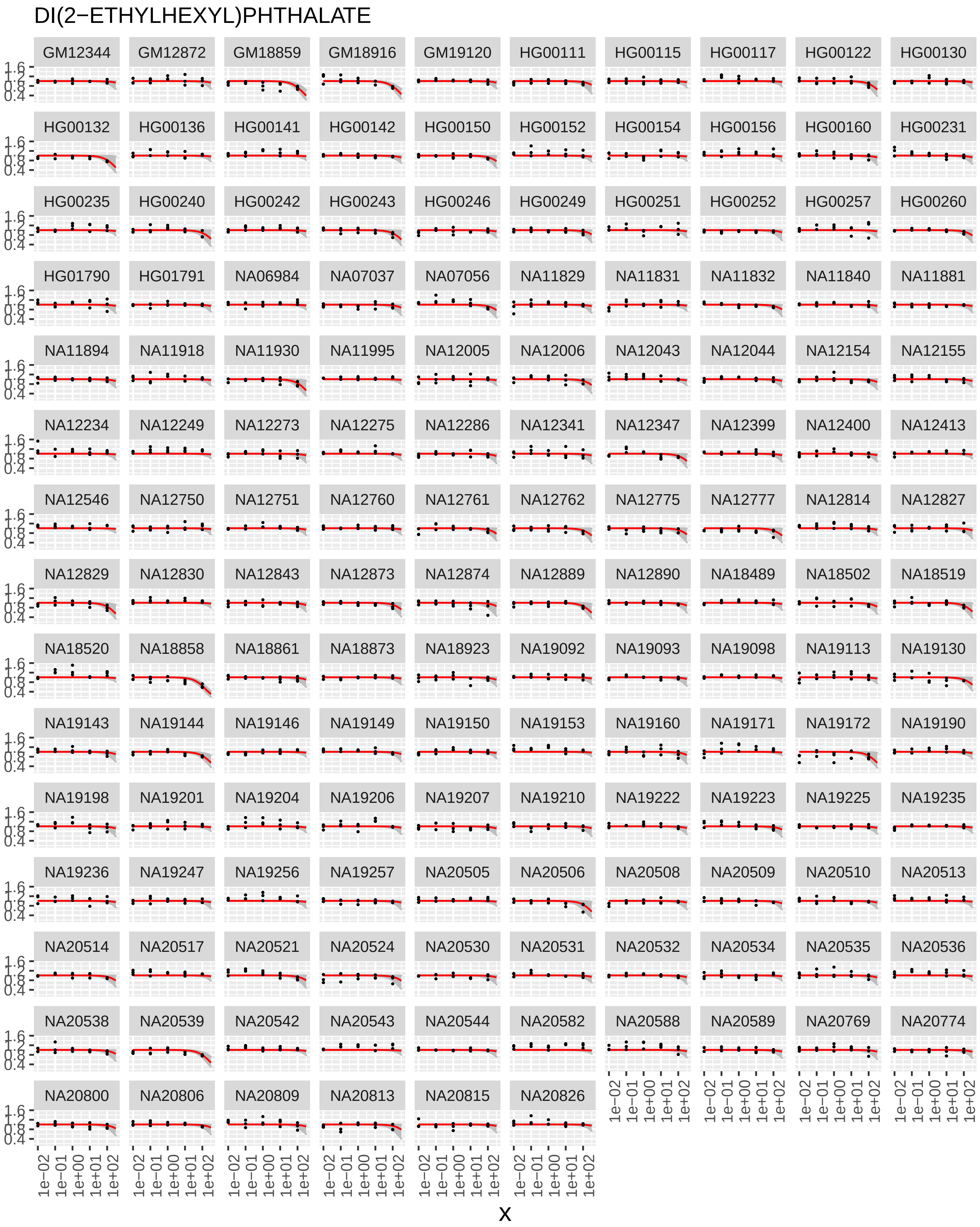
DI(2−ETHYLHEXYL)PHTHALATE
GM12344
GM12872
GM18859
GM18916
GM19120
HG00111
HG00115
HG00117
HG00122
HG00130
HG00132
HG00136
HG00141
HG00142
HG00150
HG00152
HG00154
HG00156
HG00160
HG00231
HG00235
HG00240
HG00242
HG00243
HG00246
HG00249
HG00251
HG00252
HG00257
HG00260
HG01790
HG01791
NA06984
NA07037
NA07056
NA11829
NA11831
NA11832
NA11840
NA11881
NA11894
NA11918
NA11930
NA11995
NA12005
NA12006
NA12043
NA12044
NA12154
NA12155
NA12234
NA12249
NA12273
NA12275
NA12286
NA12341
NA12347
NA12399
NA12400
NA12413
NA12546
NA12750
NA12751
NA12760
NA12761
NA12762
NA12775
NA12777
NA12814
NA12827
NA12829
NA12830
NA12843
NA12873
NA12874
NA12889
NA12890
NA18489
NA18502
NA18519
NA18520
NA18858
NA18861
NA18873
NA18923
NA19092
NA19093
NA19098
NA19113
NA19130
NA19143
NA19144
NA19146
NA19149
NA19150
NA19153
NA19160
NA19171
NA19172
NA19190
NA19198
NA19201
NA19204
NA19206
NA19207
NA19210
NA19222
NA19223
NA19225
NA19235
NA19236
NA19247
NA19256
NA19257
NA20505
NA20506
NA20508
NA20509
NA20510
NA20513
NA20514
NA20517
NA20521
NA20524
NA20530
NA20531
NA20532
NA20534
NA20535
NA20536
NA20538
NA20539
NA20542
NA20543
NA20544
NA20582
NA20588
NA20589
NA20769
NA20774
NA20800
NA20806
NA20809
NA20813
NA20815
NA20826
1.6
1.2
0.8
0.4
1e−02
1e−01
1e+00
1e+01
1e+02
x

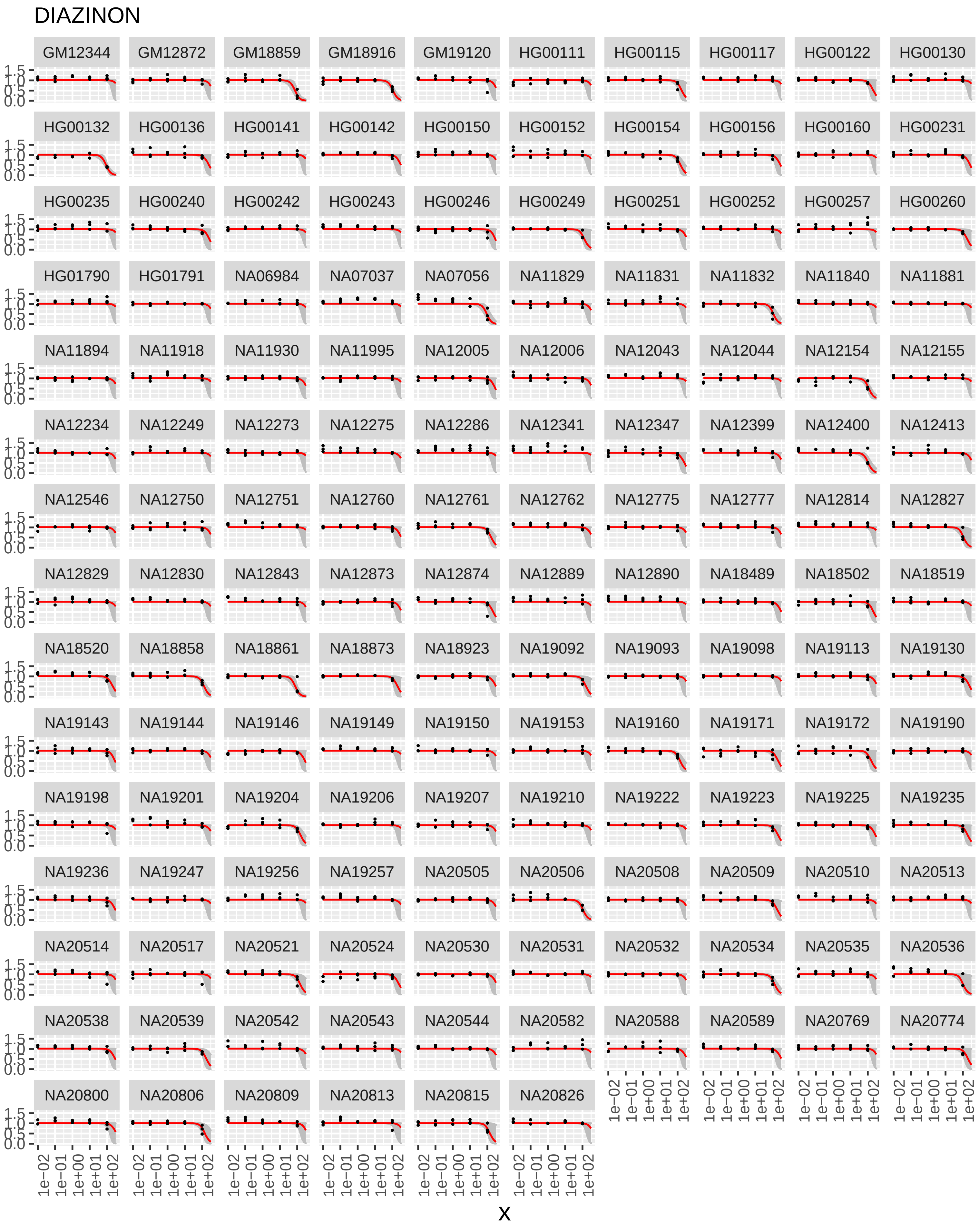
DIAZINON
GM12344
GM12872
GM18859
GM18916
GM19120
HG00111
HG00115
HG00117
HG00122
HG00130
HG00132
HG00136
HG00141
HG00142
HG00150
HG00152
HG00154
HG00156
HG00160
HG00231
HG00235
HG00240
HG00242
HG00243
HG00246
HG00249
HG00251
HG00252
HG00257
HG00260
HG01790
HG01791
NA06984
NA07037
NA07056
NA11829
NA11831
NA11832
NA11840
NA11881
NA11894
NA11918
NA11930
NA11995
NA12005
NA12006
NA12043
NA12044
NA12154
NA12155
NA12234
NA12249
NA12273
NA12275
NA12286
NA12341
NA12347
NA12399
NA12400
NA12413
NA12546
NA12750
NA12751
NA12760
NA12761
NA12762
NA12775
NA12777
NA12814
NA12827
NA12829
NA12830
NA12843
NA12873
NA12874
NA12889
NA12890
NA18489
NA18502
NA18519
NA18520
NA18858
NA18861
NA18873
NA18923
NA19092
NA19093
NA19098
NA19113
NA19130
NA19143
NA19144
NA19146
NA19149
NA19150
NA19153
NA19160
NA19171
NA19172
NA19190
NA19198
NA19201
NA19204
NA19206
NA19207
NA19210
NA19222
NA19223
NA19225
NA19235
NA19236
NA19247
NA19256
NA19257
NA20505
NA20506
NA20508
NA20509
NA20510
NA20513
NA20514
NA20517
NA20521
NA20524
NA20530
NA20531
NA20532
NA20534
NA20535
NA20536
NA20538
NA20539
NA20542
NA20543
NA20544
NA20582
NA20588
NA20589
NA20769
NA20774
NA20800
NA20806
NA20809
NA20813
NA20815
NA20826
0.0
0.5
1.0
1.5
1e−02
1e−01
1e+00
1e+01
1e+02
x

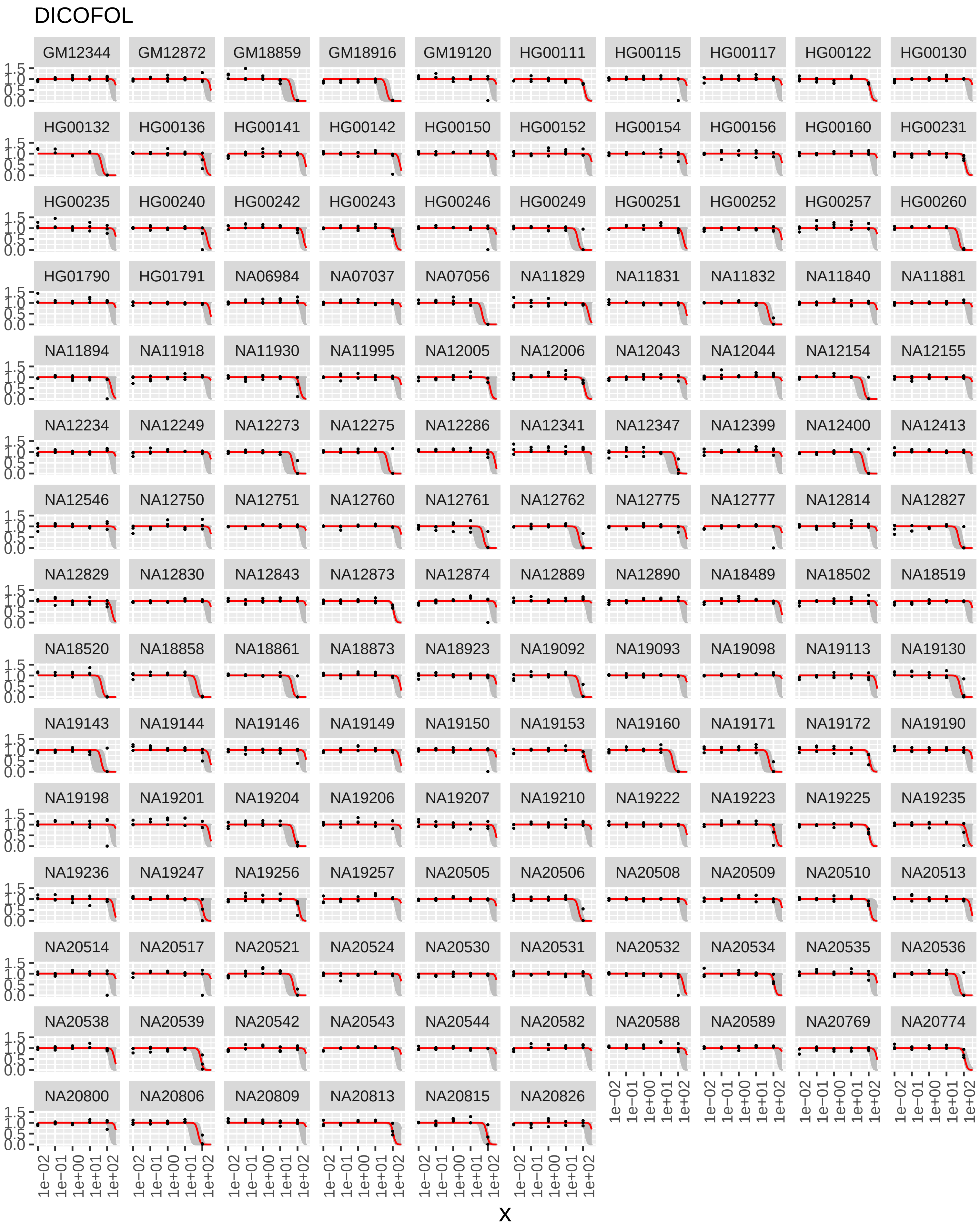
DICOFOL
GM12344
GM12872
GM18859
GM18916
GM19120
HG00111
HG00115
HG00117
HG00122
HG00130
HG00132
HG00136
HG00141
HG00142
HG00150
HG00152
HG00154
HG00156
HG00160
HG00231
HG00235
HG00240
HG00242
HG00243
HG00246
HG00249
HG00251
HG00252
HG00257
HG00260
HG01790
HG01791
NA06984
NA07037
NA07056
NA11829
NA11831
NA11832
NA11840
NA11881
NA11894
NA11918
NA11930
NA11995
NA12005
NA12006
NA12043
NA12044
NA12154
NA12155
NA12234
NA12249
NA12273
NA12275
NA12286
NA12341
NA12347
NA12399
NA12400
NA12413
NA12546
NA12750
NA12751
NA12760
NA12761
NA12762
NA12775
NA12777
NA12814
NA12827
NA12829
NA12830
NA12843
NA12873
NA12874
NA12889
NA12890
NA18489
NA18502
NA18519
NA18520
NA18858
NA18861
NA18873
NA18923
NA19092
NA19093
NA19098
NA19113
NA19130
NA19143
NA19144
NA19146
NA19149
NA19150
NA19153
NA19160
NA19171
NA19172
NA19190
NA19198
NA19201
NA19204
NA19206
NA19207
NA19210
NA19222
NA19223
NA19225
NA19235
NA19236
NA19247
NA19256
NA19257
NA20505
NA20506
NA20508
NA20509
NA20510
NA20513
NA20514
NA20517
NA20521
NA20524
NA20530
NA20531
NA20532
NA20534
NA20535
NA20536
NA20538
NA20539
NA20542
NA20543
NA20544
NA20582
NA20588
NA20589
NA20769
NA20774
NA20800
NA20806
NA20809
NA20813
NA20815
NA20826
1.5
1.0
0.5
0.0
1e−02
1e−01
1e+00
1e+01
1e+02
x

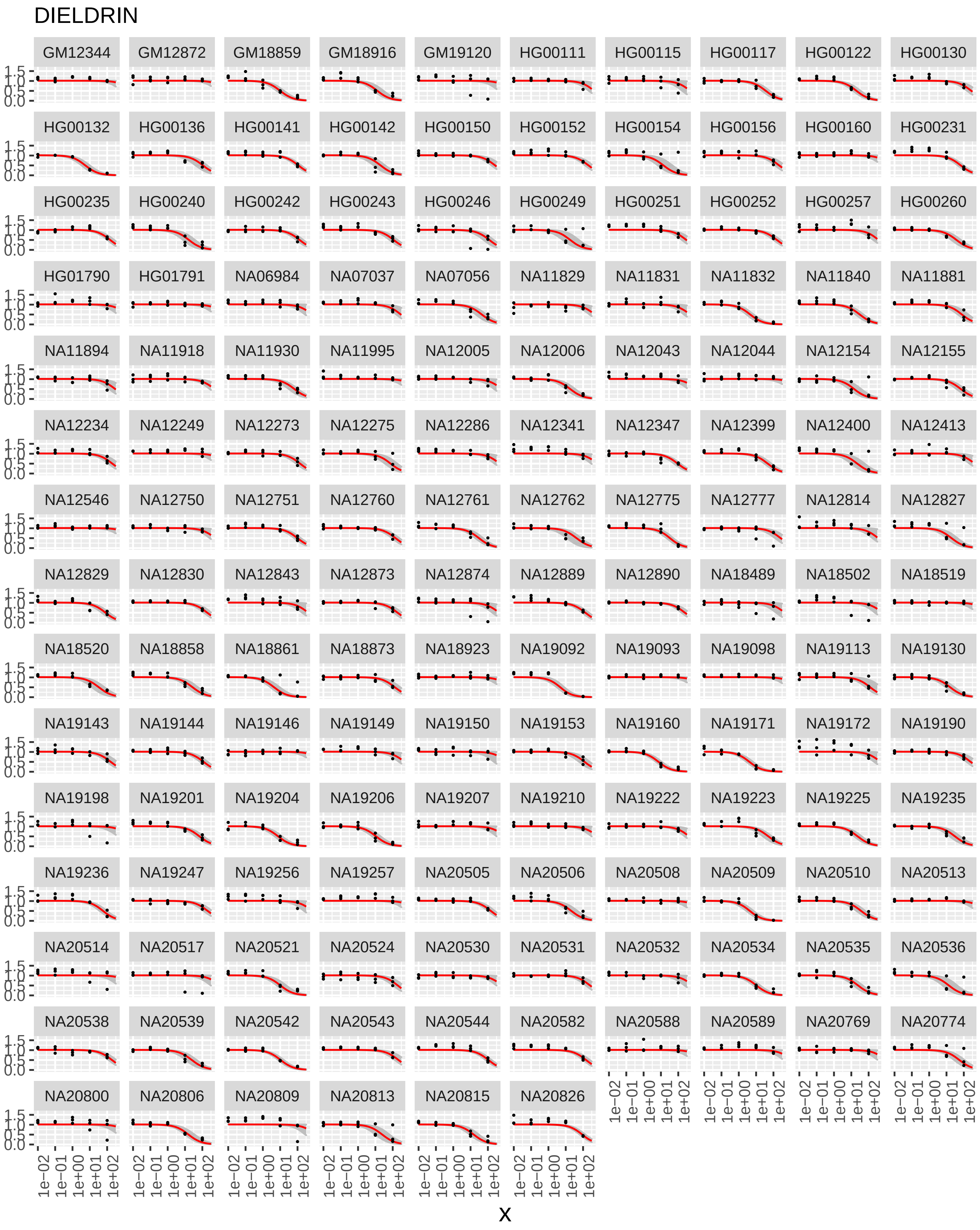
DIELDRIN
GM12344
GM12872
GM18859
GM18916
GM19120
HG00111
HG00115
HG00117
HG00122
HG00130
HG00132
HG00136
HG00141
HG00142
HG00150
HG00152
HG00154
HG00156
HG00160
HG00231
HG00235
HG00240
HG00242
HG00243
HG00246
HG00249
HG00251
HG00252
HG00257
HG00260
HG01790
HG01791
NA06984
NA07037
NA07056
NA11829
NA11831
NA11832
NA11840
NA11881
NA11894
NA11918
NA11930
NA11995
NA12005
NA12006
NA12043
NA12044
NA12154
NA12155
NA12234
NA12249
NA12273
NA12275
NA12286
NA12341
NA12347
NA12399
NA12400
NA12413
NA12546
NA12750
NA12751
NA12760
NA12761
NA12762
NA12775
NA12777
NA12814
NA12827
NA12829
NA12830
NA12843
NA12873
NA12874
NA12889
NA12890
NA18489
NA18502
NA18519
NA18520
NA18858
NA18861
NA18873
NA18923
NA19092
NA19093
NA19098
NA19113
NA19130
NA19143
NA19144
NA19146
NA19149
NA19150
NA19153
NA19160
NA19171
NA19172
NA19190
NA19198
NA19201
NA19204
NA19206
NA19207
NA19210
NA19222
NA19223
NA19225
NA19235
NA19236
NA19247
NA19256
NA19257
NA20505
NA20506
NA20508
NA20509
NA20510
NA20513
NA20514
NA20517
NA20521
NA20524
NA20530
NA20531
NA20532
NA20534
NA20535
NA20536
NA20538
NA20539
NA20542
NA20543
NA20544
NA20582
NA20588
NA20589
NA20769
NA20774
NA20800
NA20806
NA20809
NA20813
NA20815
NA20826
1.5
1.0
0.5
0.0
1e−02
1e−01
1e+00
1e+01
1e+02
x

DI−N−BUTYL PHTHALATE

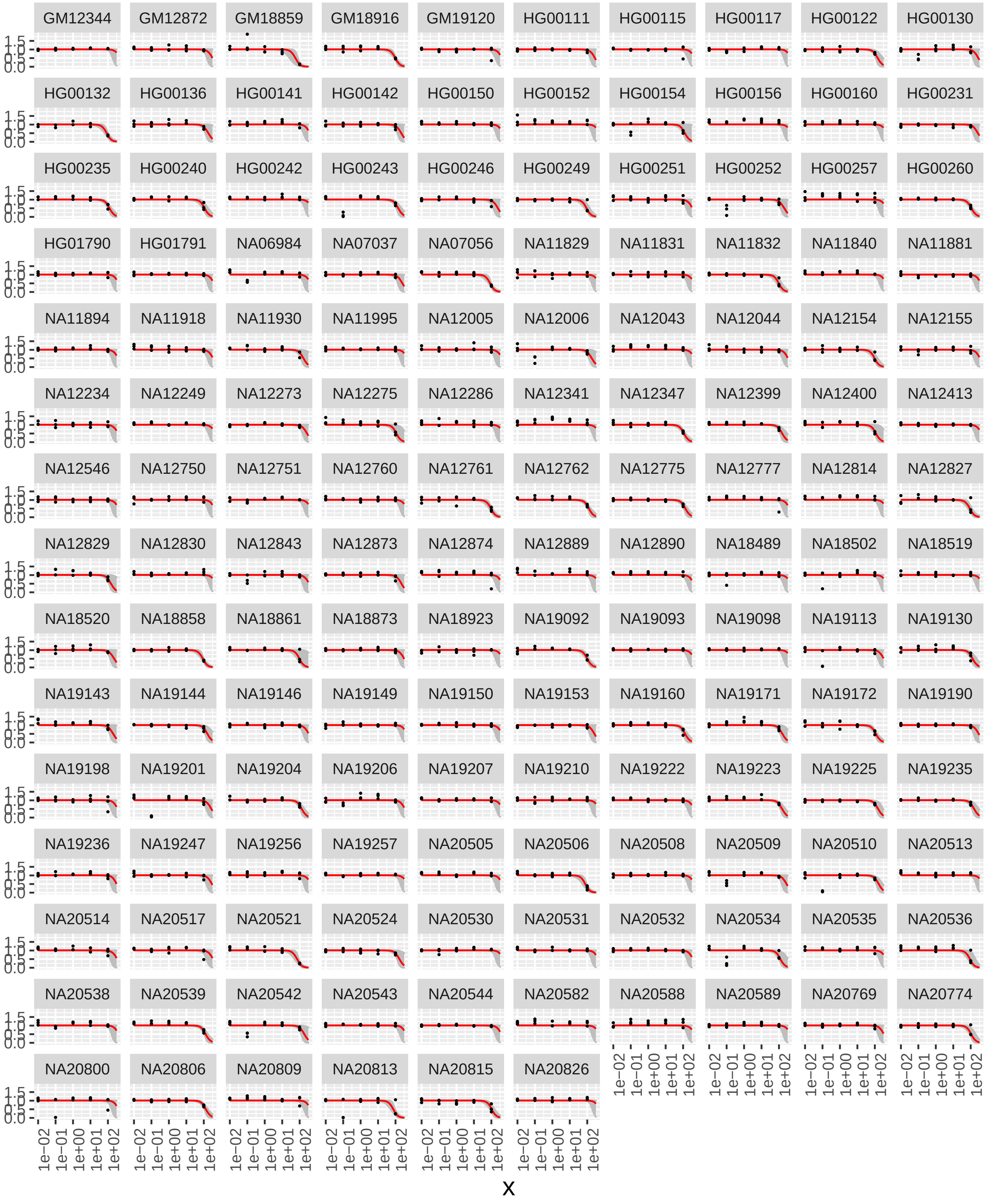

DISULFOTON

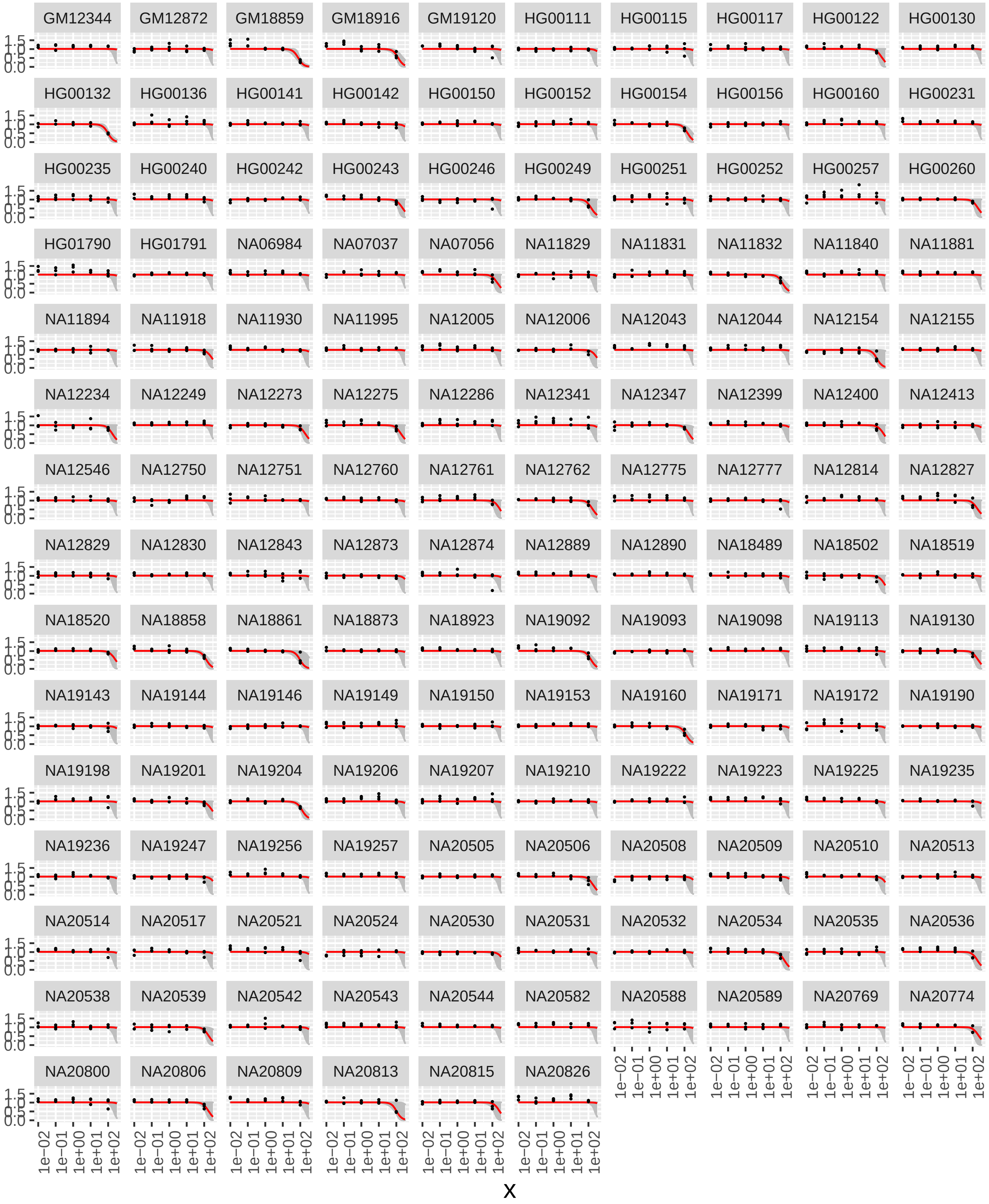
GM12344
GM12872
GM18859
GM18916
GM19120
HG00111
HG00115
HG00117
HG00122
HG00130
HG00132
HG00136
HG00141
HG00142
HG00150
HG00152
HG00154
HG00156
HG00160
HG00231
HG00235
HG00240
HG00242
HG00243
HG00246
HG00249
HG00251
HG00252
HG00257
HG00260
HG01790
HG01791
NA06984
NA07037
NA07056
NA11829
NA11831
NA11832
NA11840
NA11881
NA11894
NA11918
NA11930
NA11995
NA12005
NA12006
NA12043
NA12044
NA12154
NA12155
NA12234
NA12249
NA12273
NA12275
NA12286
NA12341
NA12347
NA12399
NA12400
NA12413
NA12546
NA12750
NA12751
NA12760
NA12761
NA12762
NA12775
NA12777
NA12814
NA12827
NA12829
NA12830
NA12843
NA12873
NA12874
NA12889
NA12890
NA18489
NA18502
NA18519
NA18520
NA18858
NA18861
NA18873
NA18923
NA19092
NA19093
NA19098
NA19113
NA19130
NA19143
NA19144
NA19146
NA19149
NA19150
NA19153
NA19160
NA19171
NA19172
NA19190
NA19198
NA19201
NA19204
NA19206
NA19207
NA19210
NA19222
NA19223
NA19225
NA19235
NA19236
NA19247
NA19256
NA19257
NA20505
NA20506
NA20508
NA20509
NA20510
NA20513
NA20514
NA20517
NA20521
NA20524
NA20530
NA20531
NA20532
NA20534
NA20535
NA20536
NA20538
NA20539
NA20542
NA20543
NA20544
NA20582
NA20588
NA20589
NA20769
NA20774
NA20800
NA20806
NA20809
NA20813
NA20815
NA20826
1.5
1.0
0.5
0.0
1e−02
1e−01
1e+00
1e+01
1e+02
x

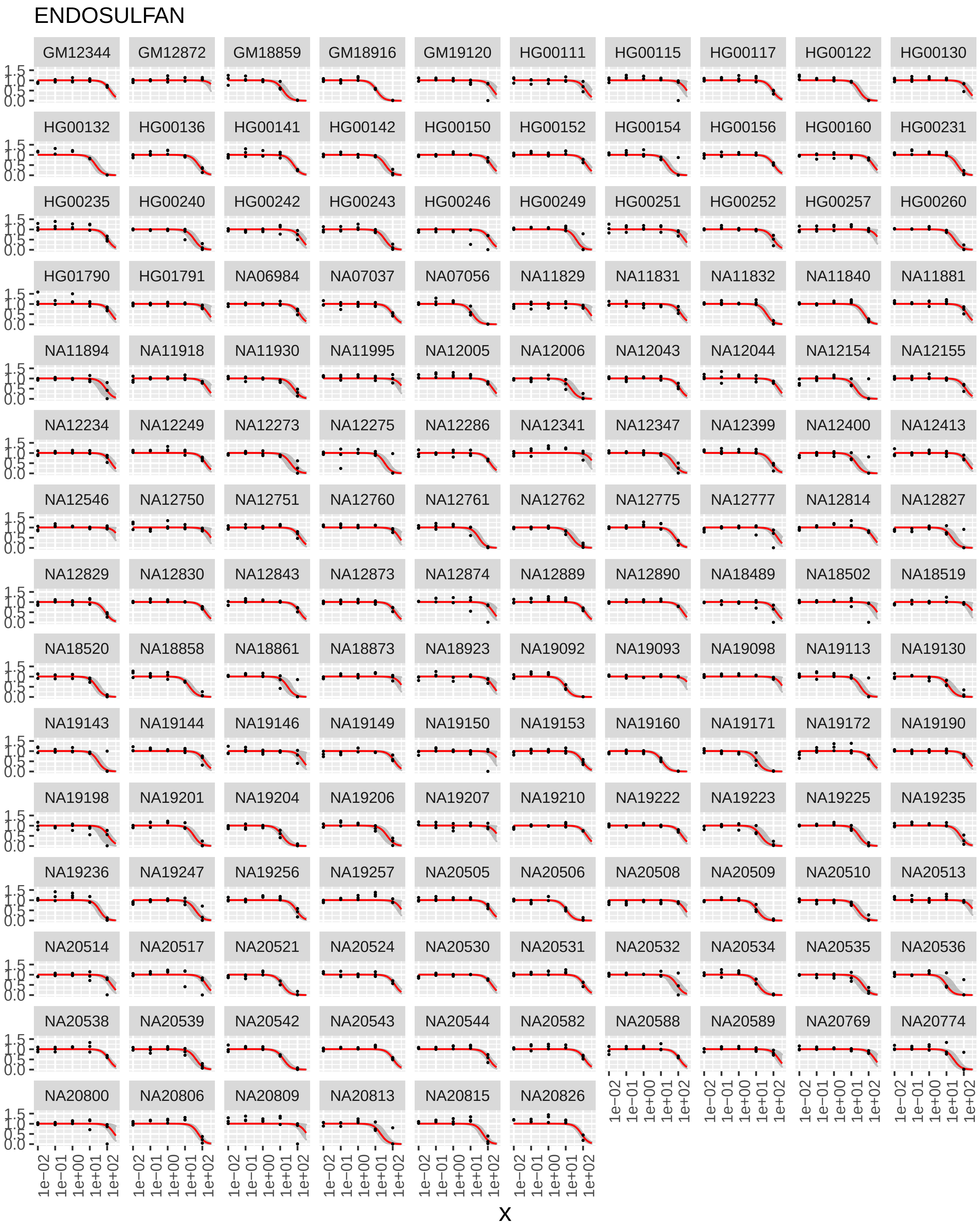

ENDOSULFAN
GM12344
GM12872
GM18859
GM18916
GM19120
HG00111
HG00115
HG00117
HG00122
HG00130
HG00132
HG00136
HG00141
HG00142
HG00150
HG00152
HG00154
HG00156
HG00160
HG00231
HG00235
HG00240
HG00242
HG00243
HG00246
HG00249
HG00251
HG00252
HG00257
HG00260
HG01790
HG01791
NA06984
NA07037
NA07056
NA11829
NA11831
NA11832
NA11840
NA11881
NA11894
NA11918
NA11930
NA11995
NA12005
NA12006
NA12043
NA12044
NA12154
NA12155
NA12234
NA12249
NA12273
NA12275
NA12286
NA12341
NA12347
NA12399
NA12400
NA12413
NA12546
NA12750
NA12751
NA12760
NA12761
NA12762
NA12775
NA12777
NA12814
NA12827
NA12829
NA12830
NA12843
NA12873
NA12874
NA12889
NA12890
NA18489
NA18502
NA18519
NA18520
NA18858
NA18861
NA18873
NA18923
NA19092
NA19093
NA19098
NA19113
NA19130
NA19143
NA19144
NA19146
NA19149
NA19150
NA19153
NA19160
NA19171
NA19172
NA19190
NA19198
NA19201
NA19204
NA19206
NA19207
NA19210
NA19222
NA19223
NA19225
NA19235
NA19236
NA19247
NA19256
NA19257
NA20505
NA20506
NA20508
NA20509
NA20510
NA20513
NA20514
NA20517
NA20521
NA20524
NA20530
NA20531
NA20532
NA20534
NA20535
NA20536
NA20538
NA20539
NA20542
NA20543
NA20544
NA20582
NA20588
NA20589
NA20769
NA20774
NA20800
NA20806
NA20809
NA20813
NA20815
NA20826
1.5
1.0
0.5
0.0
1e−02
1e−01
1e+00
1e+01
1e+02
x

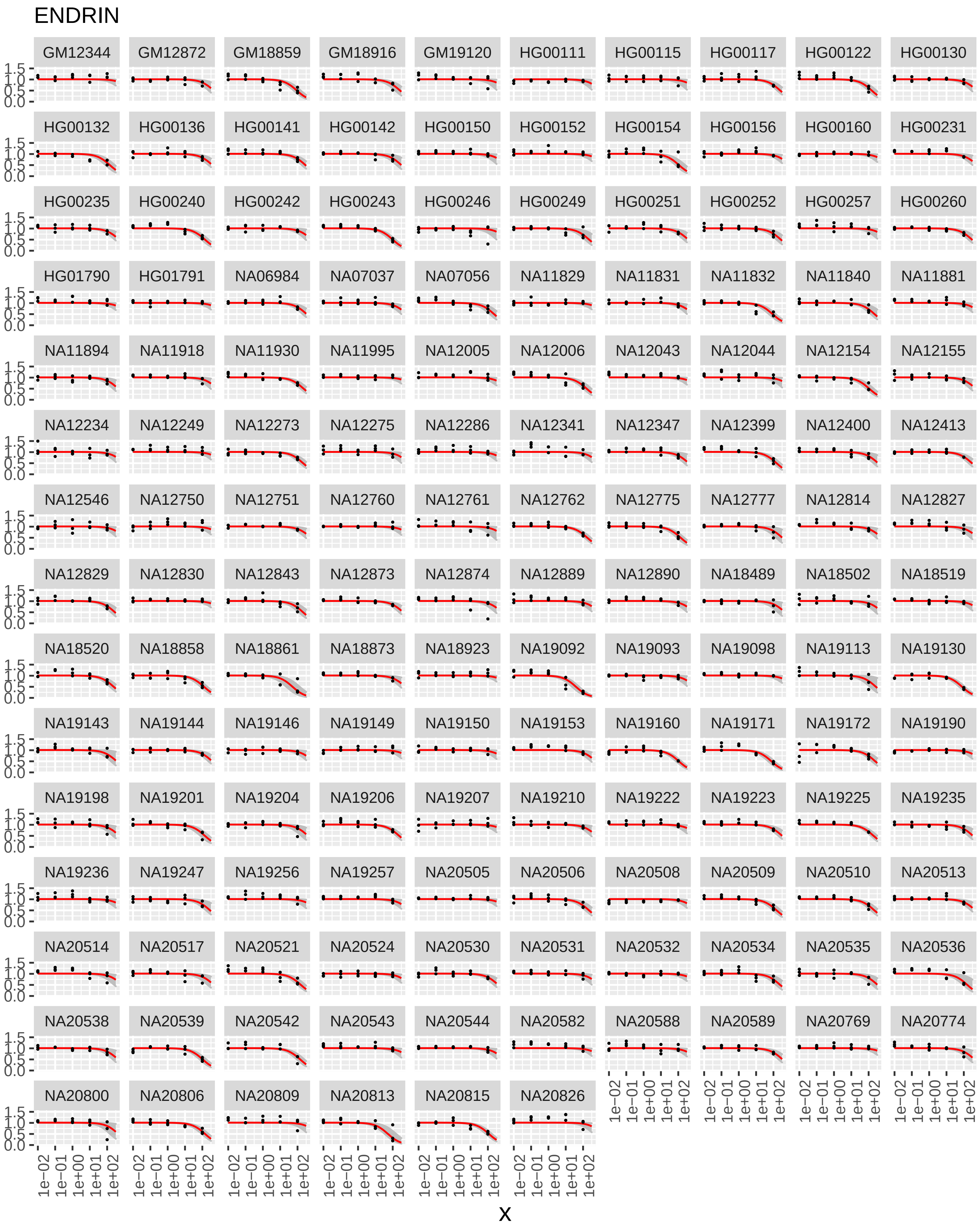
ENDRIN
GM12344
GM12872
GM18859
GM18916
GM19120
HG00111
HG00115
HG00117
HG00122
HG00130
HG00132
HG00136
HG00141
HG00142
HG00150
HG00152
HG00154
HG00156
HG00160
HG00231
HG00235
HG00240
HG00242
HG00243
HG00246
HG00249
HG00251
HG00252
HG00257
HG00260
HG01790
HG01791
NA06984
NA07037
NA07056
NA11829
NA11831
NA11832
NA11840
NA11881
NA11894
NA11918
NA11930
NA11995
NA12005
NA12006
NA12043
NA12044
NA12154
NA12155
NA12234
NA12249
NA12273
NA12275
NA12286
NA12341
NA12347
NA12399
NA12400
NA12413
NA12546
NA12750
NA12751
NA12760
NA12761
NA12762
NA12775
NA12777
NA12814
NA12827
NA12829
NA12830
NA12843
NA12873
NA12874
NA12889
NA12890
NA18489
NA18502
NA18519
NA18520
NA18858
NA18861
NA18873
NA18923
NA19092
NA19093
NA19098
NA19113
NA19130
NA19143
NA19144
NA19146
NA19149
NA19150
NA19153
NA19160
NA19171
NA19172
NA19190
NA19198
NA19201
NA19204
NA19206
NA19207
NA19210
NA19222
NA19223
NA19225
NA19235
NA19236
NA19247
NA19256
NA19257
NA20505
NA20506
NA20508
NA20509
NA20510
NA20513
NA20514
NA20517
NA20521
NA20524
NA20530
NA20531
NA20532
NA20534
NA20535
NA20536
NA20538
NA20539
NA20542
NA20543
NA20544
NA20582
NA20588
NA20589
NA20769
NA20774
NA20800
NA20806
NA20809
NA20813
NA20815
NA20826
1.5
1.0
0.5
0.0
1e−02
1e−01
1e+00
1e+01
1e+02
x

ETHION

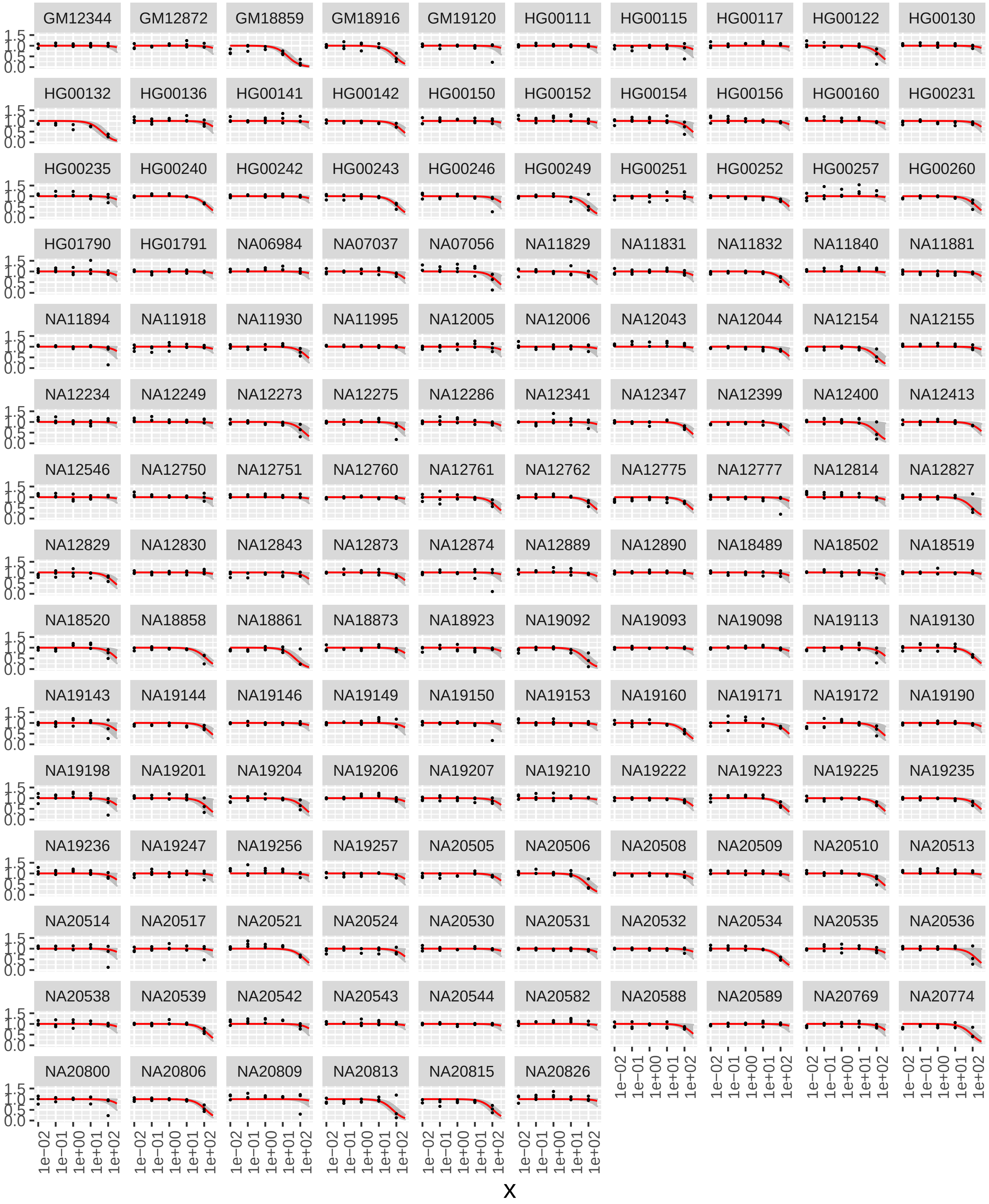
GM12344
GM12872
GM18859
GM18916
GM19120
HG00111
HG00115
HG00117
HG00122
HG00130
HG00132
HG00136
HG00141
HG00142
HG00150
HG00152
HG00154
HG00156
HG00160
HG00231
HG00235
HG00240
HG00242
HG00243
HG00246
HG00249
HG00251
HG00252
HG00257
HG00260
HG01790
HG01791
NA06984
NA07037
NA07056
NA11829
NA11831
NA11832
NA11840
NA11881
NA11894
NA11918
NA11930
NA11995
NA12005
NA12006
NA12043
NA12044
NA12154
NA12155
NA12234
NA12249
NA12273
NA12275
NA12286
NA12341
NA12347
NA12399
NA12400
NA12413
NA12546
NA12750
NA12751
NA12760
NA12761
NA12762
NA12775
NA12777
NA12814
NA12827
NA12829
NA12830
NA12843
NA12873
NA12874
NA12889
NA12890
NA18489
NA18502
NA18519
NA18520
NA18858
NA18861
NA18873
NA18923
NA19092
NA19093
NA19098
NA19113
NA19130
NA19143
NA19144
NA19146
NA19149
NA19150
NA19153
NA19160
NA19171
NA19172
NA19190
NA19198
NA19201
NA19204
NA19206
NA19207
NA19210
NA19222
NA19223
NA19225
NA19235
NA19236
NA19247
NA19256
NA19257
NA20505
NA20506
NA20508
NA20509
NA20510
NA20513
NA20514
NA20517
NA20521
NA20524
NA20530
NA20531
NA20532
NA20534
NA20535
NA20536
NA20538
NA20539
NA20542
NA20543
NA20544
NA20582
NA20588
NA20589
NA20769
NA20774
NA20800
NA20806
NA20809
NA20813
NA20815
NA20826
1.5
1.0
0.5
0.0
1e−02
1e−01
1e+00
1e+01
1e+02
x

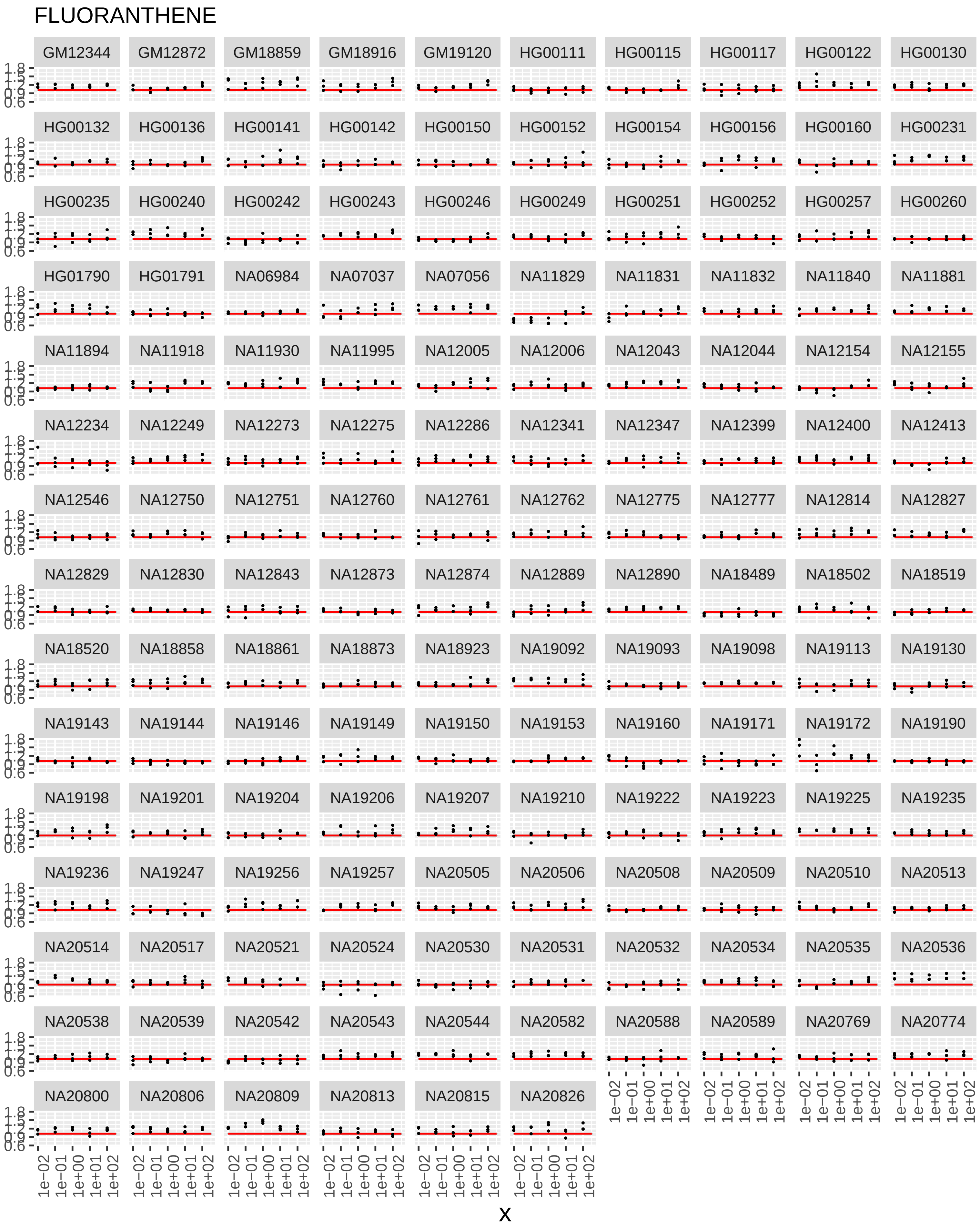
FLUORANTHENE
GM12344
GM12872
GM18859
GM18916
GM19120
HG00111
HG00115
HG00117
HG00122
HG00130
HG00132
HG00136
HG00141
HG00142
HG00150
HG00152
HG00154
HG00156
HG00160
HG00231
HG00235
HG00240
HG00242
HG00243
HG00246
HG00249
HG00251
HG00252
HG00257
HG00260
HG01790
HG01791
NA06984
NA07037
NA07056
NA11829
NA11831
NA11832
NA11840
NA11881
NA11894
NA11918
NA11930
NA11995
NA12005
NA12006
NA12043
NA12044
NA12154
NA12155
NA12234
NA12249
NA12273
NA12275
NA12286
NA12341
NA12347
NA12399
NA12400
NA12413
NA12546
NA12750
NA12751
NA12760
NA12761
NA12762
NA12775
NA12777
NA12814
NA12827
NA12829
NA12830
NA12843
NA12873
NA12874
NA12889
NA12890
NA18489
NA18502
NA18519
NA18520
NA18858
NA18861
NA18873
NA18923
NA19092
NA19093
NA19098
NA19113
NA19130
NA19143
NA19144
NA19146
NA19149
NA19150
NA19153
NA19160
NA19171
NA19172
NA19190
NA19198
NA19201
NA19204
NA19206
NA19207
NA19210
NA19222
NA19223
NA19225
NA19235
NA19236
NA19247
NA19256
NA19257
NA20505
NA20506
NA20508
NA20509
NA20510
NA20513
NA20514
NA20517
NA20521
NA20524
NA20530
NA20531
NA20532
NA20534
NA20535
NA20536
NA20538
NA20539
NA20542
NA20543
NA20544
NA20582
NA20588
NA20589
NA20769
NA20774
NA20800
NA20806
NA20809
NA20813
NA20815
NA20826
1.8
1.5
1.2
0.9
0.6
1e−02
1e−01
1e+00
1e+01
1e+02
x

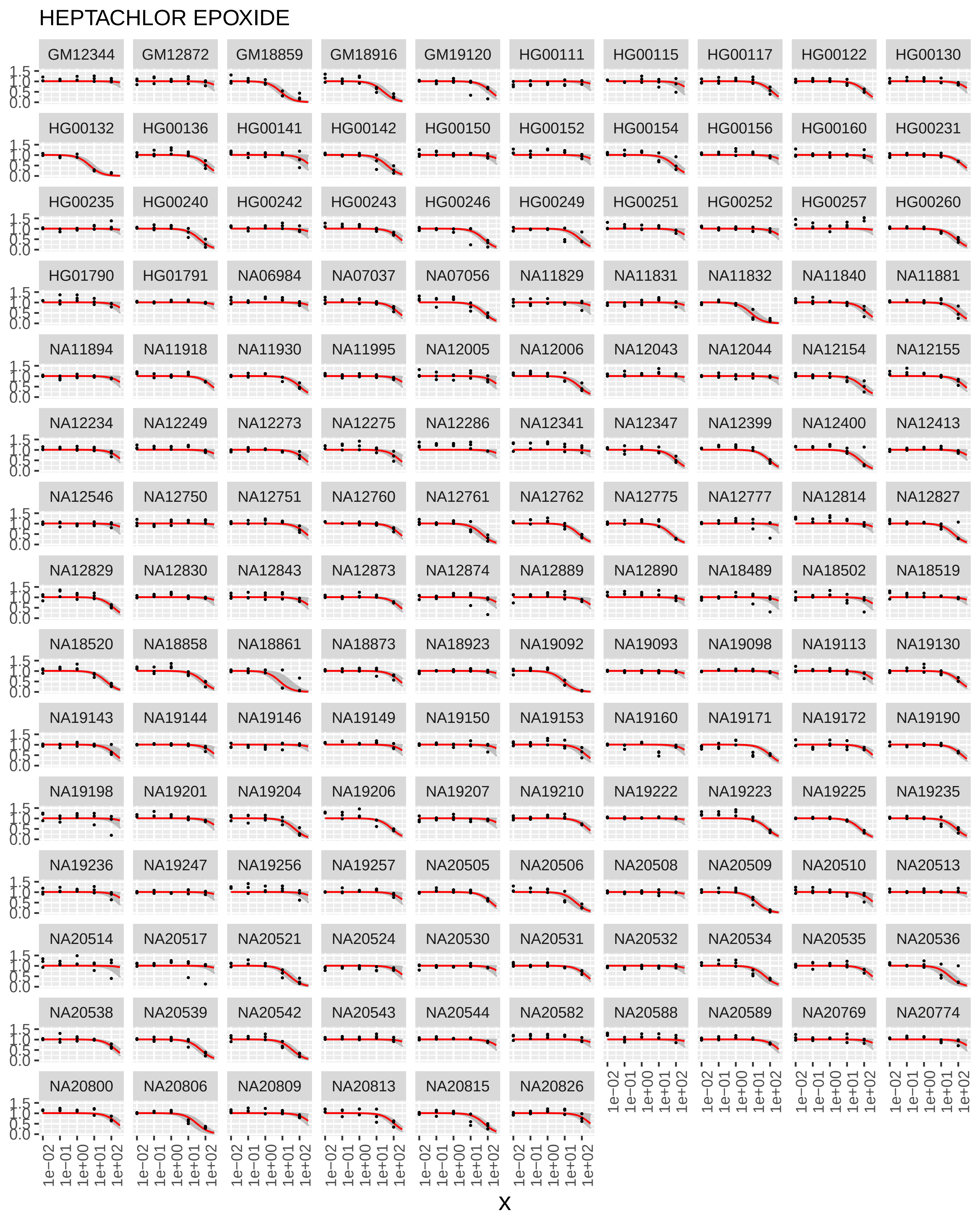
HEPTACHLOR EPOXIDE
x

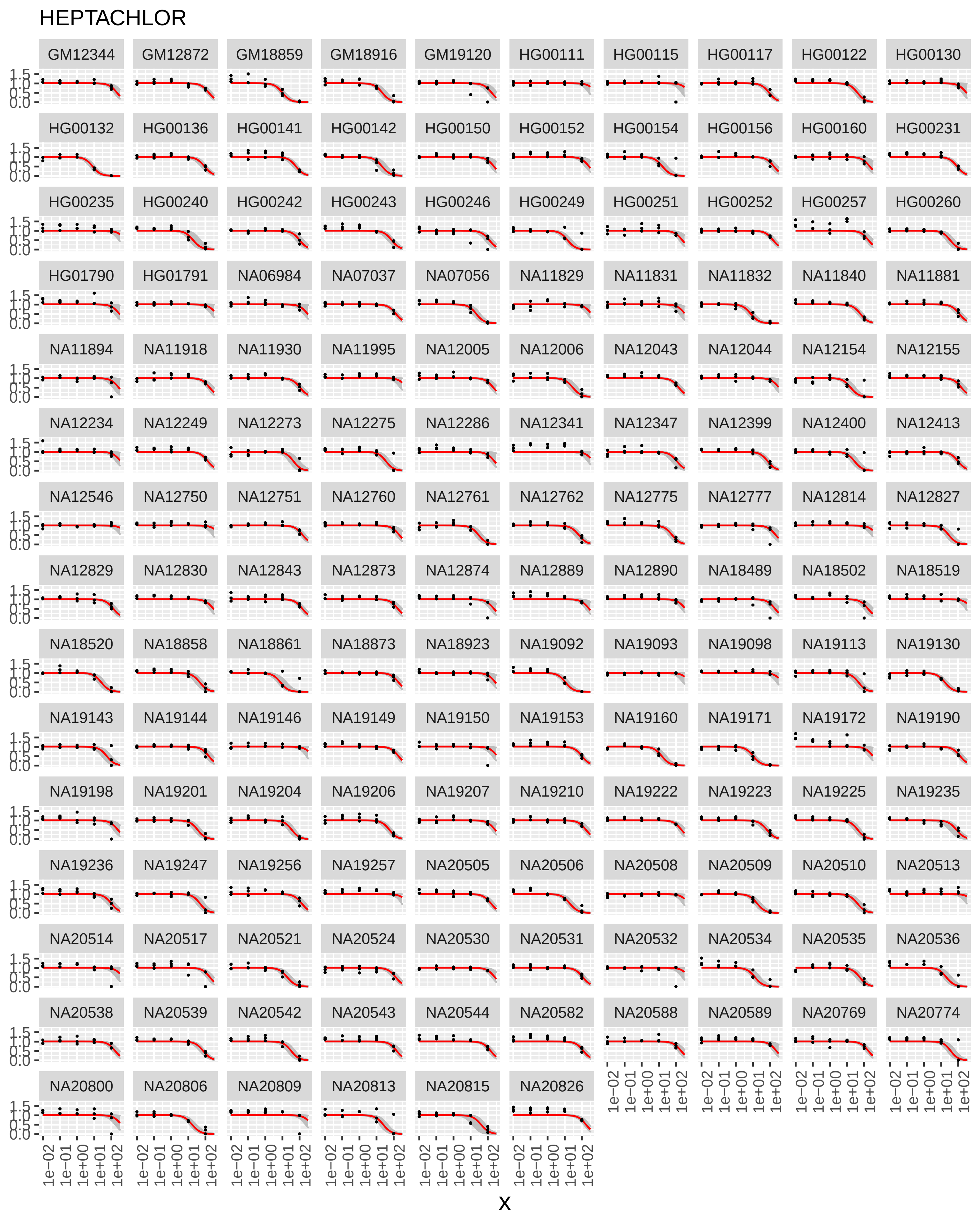
HEPTACHLOR
GM12344 GM12872 GM18859 GM18916 GM19120 HG00111 HG00115 HG00117 HG00122 HG00130
HG00132 HG00136 HG00141 HG00142 HG00150 HG00152 HG00154 HG00156 HG00160 HG00231
HG00235 HG00240 HG00242 HG00243 HG00246 HG00249 HG00251 HG00252 HG00257 HG00260
HG01790 HG01791 NA06984 NA07037 NA07056 NA11829 NA11831 NA11832 NA11840 NA11881
NA11894 NA11918 NA11930 NA11995 NA12005 NA12006 NA12043 NA12044 NA12154 NA12155
NA12234 NA12249 NA12273 NA12275 NA12286 NA12341 NA12347 NA12399 NA12400 NA12413
NA12546 NA12750 NA12751 NA12760 NA12761 NA12762 NA12775 NA12777 NA12814 NA12827
NA12829 NA12830 NA12843 NA12873 NA12874 NA12889 NA12890 NA18489 NA18502 NA18519
NA18520 NA18858 NA18861 NA18873 NA18923 NA19092 NA19093 NA19098 NA19113 NA19130
NA19143 NA19144 NA19146 NA19149 NA19150 NA19153 NA19160 NA19171 NA19172 NA19190
NA19198 NA19201 NA19204 NA19206 NA19207 NA19210 NA19222 NA19223 NA19225 NA19235
NA19236 NA19247 NA19256 NA19257 NA20505 NA20506 NA20508 NA20509 NA20510 NA20513
NA20514 NA20517 NA20521 NA20524 NA20530 NA20531 NA20532 NA20534 NA20535 NA20536
NA20538 NA20539 NA20542 NA20543 NA20544 NA20582 NA20588 NA20589 NA20769 NA20774
NA20800 NA20806 NA20809 NA20813 NA20815 NA20826
1.5
1.0
0.5
0.0
1e−02
1e−01
1e+00
1e+01
1e+02
x

HEXACHLOROCYCLOHEXANE, GAMMA−

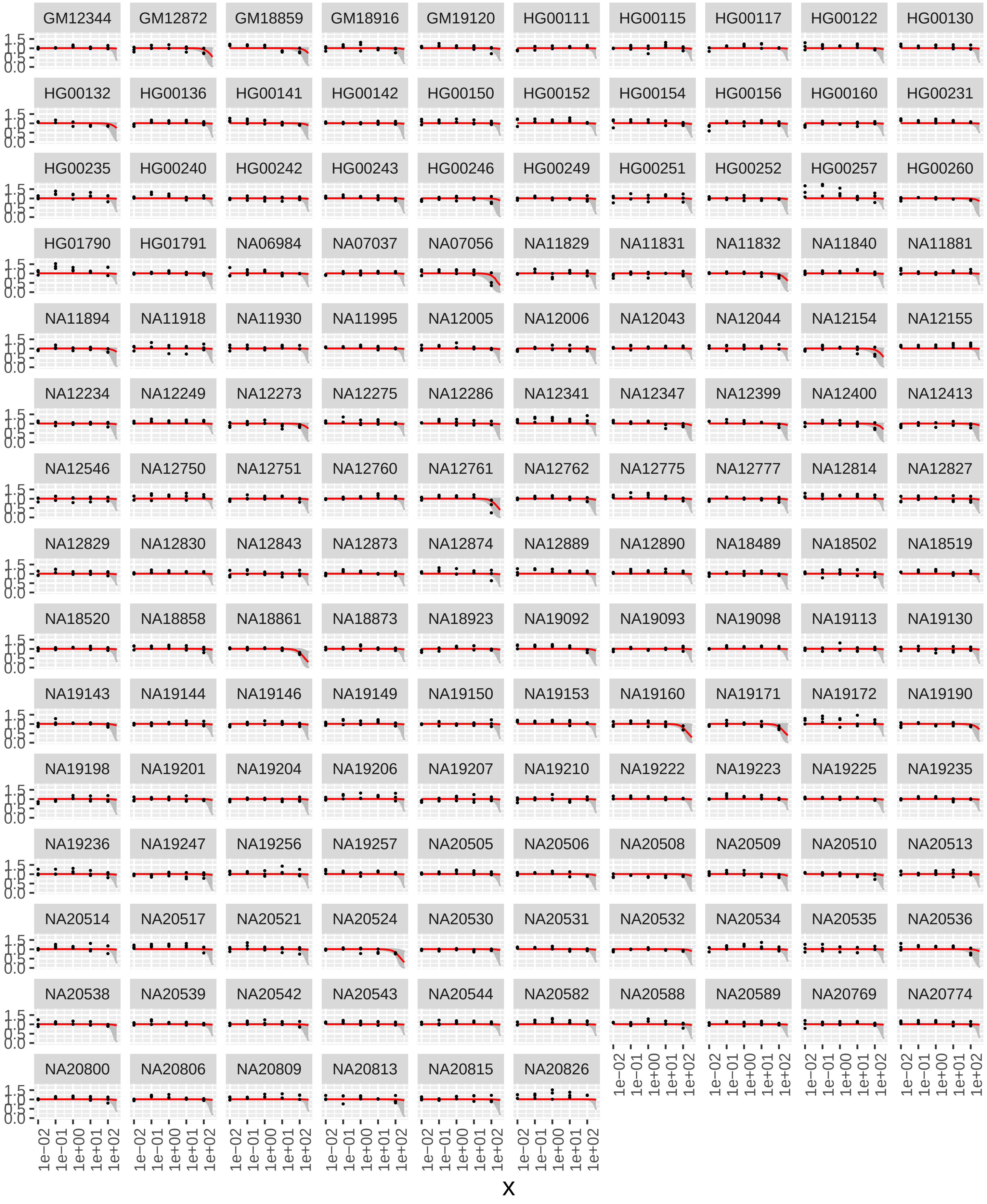

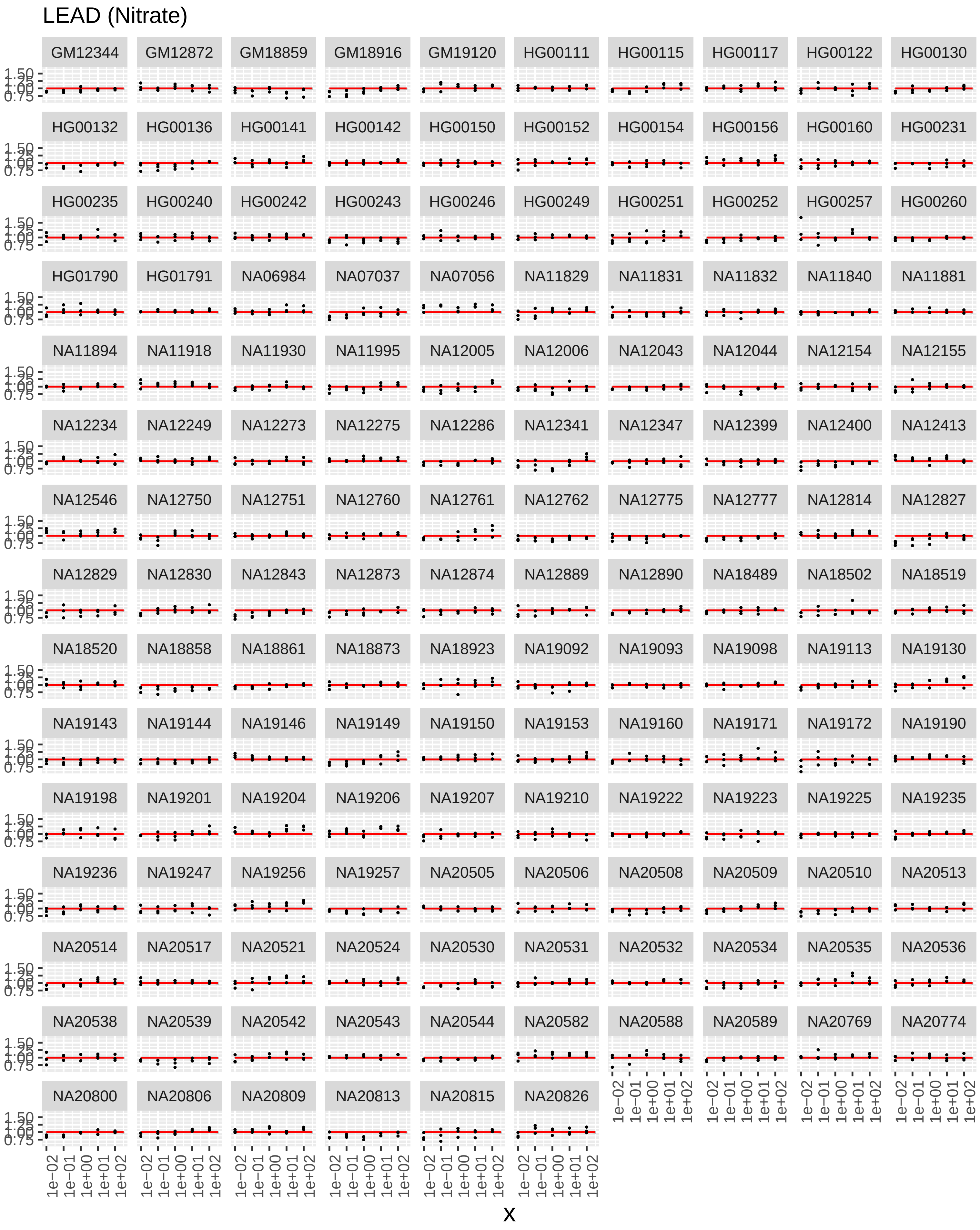
LEAD (Nitrate)
GM12344
GM12872
GM18859
GM18916
GM19120
HG00111
HG00115
HG00117
HG00122
HG00130
HG00132
HG00136
HG00141
HG00142
HG00150
HG00152
HG00154
HG00156
HG00160
HG00231
HG00235
HG00240
HG00242
HG00243
HG00246
HG00249
HG00251
HG00252
HG00257
HG00260
HG01790
HG01791
NA06984
NA07037
NA07056
NA11829
NA11831
NA11832
NA11840
NA11881
NA11894
NA11918
NA11930
NA11995
NA12005
NA12006
NA12043
NA12044
NA12154
NA12155
NA12234
NA12249
NA12273
NA12275
NA12286
NA12341
NA12347
NA12399
NA12400
NA12413
NA12546
NA12750
NA12751
NA12760
NA12761
NA12762
NA12775
NA12777
NA12814
NA12827
NA12829
NA12830
NA12843
NA12873
NA12874
NA12889
NA12890
NA18489
NA18502
NA18519
NA18520
NA18858
NA18861
NA18873
NA18923
NA19092
NA19093
NA19098
NA19113
NA19130
NA19143
NA19144
NA19146
NA19149
NA19150
NA19153
NA19160
NA19171
NA19172
NA19190
NA19198
NA19201
NA19204
NA19206
NA19207
NA19210
NA19222
NA19223
NA19225
NA19235
NA19236
NA19247
NA19256
NA19257
NA20505
NA20506
NA20508
NA20509
NA20510
NA20513
NA20514
NA20517
NA20521
NA20524
NA20530
NA20531
NA20532
NA20534
NA20535
NA20536
NA20538
NA20539
NA20542
NA20543
NA20544
NA20582
NA20588
NA20589
NA20769
NA20774
NA20800
NA20806
NA20809
NA20813
NA20815
NA20826
1.50
1.25
1.00
0.75
1e−02
1e−01
1e+00
1e+01
1e+02
x

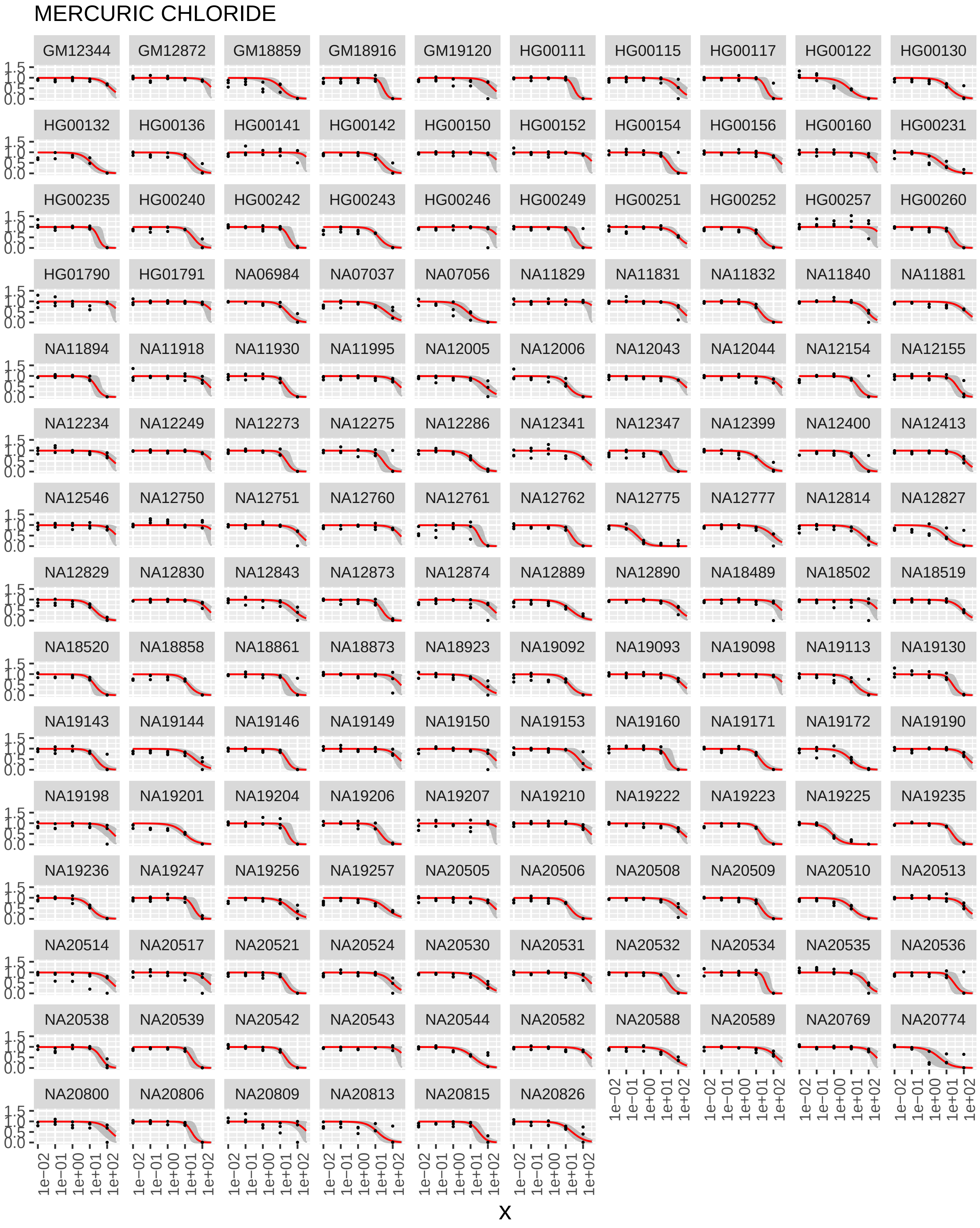
MERCURIC CHLORIDE
GM12344
GM12872
GM18859
GM18916
GM19120
HG00111
HG00115
HG00117
HG00122
HG00130
HG00132
HG00136
HG00141
HG00142
HG00150
HG00152
HG00154
HG00156
HG00160
HG00231
HG00235
HG00240
HG00242
HG00243
HG00246
HG00249
HG00251
HG00252
HG00257
HG00260
HG01790
HG01791
NA06984
NA07037
NA07056
NA11829
NA11831
NA11832
NA11840
NA11881
NA11894
NA11918
NA11930
NA11995
NA12005
NA12006
NA12043
NA12044
NA12154
NA12155
NA12234
NA12249
NA12273
NA12275
NA12286
NA12341
NA12347
NA12399
NA12400
NA12413
NA12546
NA12750
NA12751
NA12760
NA12761
NA12762
NA12775
NA12777
NA12814
NA12827
NA12829
NA12830
NA12843
NA12873
NA12874
NA12889
NA12890
NA18489
NA18502
NA18519
NA18520
NA18858
NA18861
NA18873
NA18923
NA19092
NA19093
NA19098
NA19113
NA19130
NA19143
NA19144
NA19146
NA19149
NA19150
NA19153
NA19160
NA19171
NA19172
NA19190
NA19198
NA19201
NA19204
NA19206
NA19207
NA19210
NA19222
NA19223
NA19225
NA19235
NA19236
NA19247
NA19256
NA19257
NA20505
NA20506
NA20508
NA20509
NA20510
NA20513
NA20514
NA20517
NA20521
NA20524
NA20530
NA20531
NA20532
NA20534
NA20535
NA20536
NA20538
NA20539
NA20542
NA20543
NA20544
NA20582
NA20588
NA20589
NA20769
NA20774
NA20800
NA20806
NA20809
NA20813
NA20815
NA20826
1.5
1.0
0.5
0.0
1e−02
1e−01
1e+00
1e+01
1e+02
x

MERCURIC CHLORIDE−2

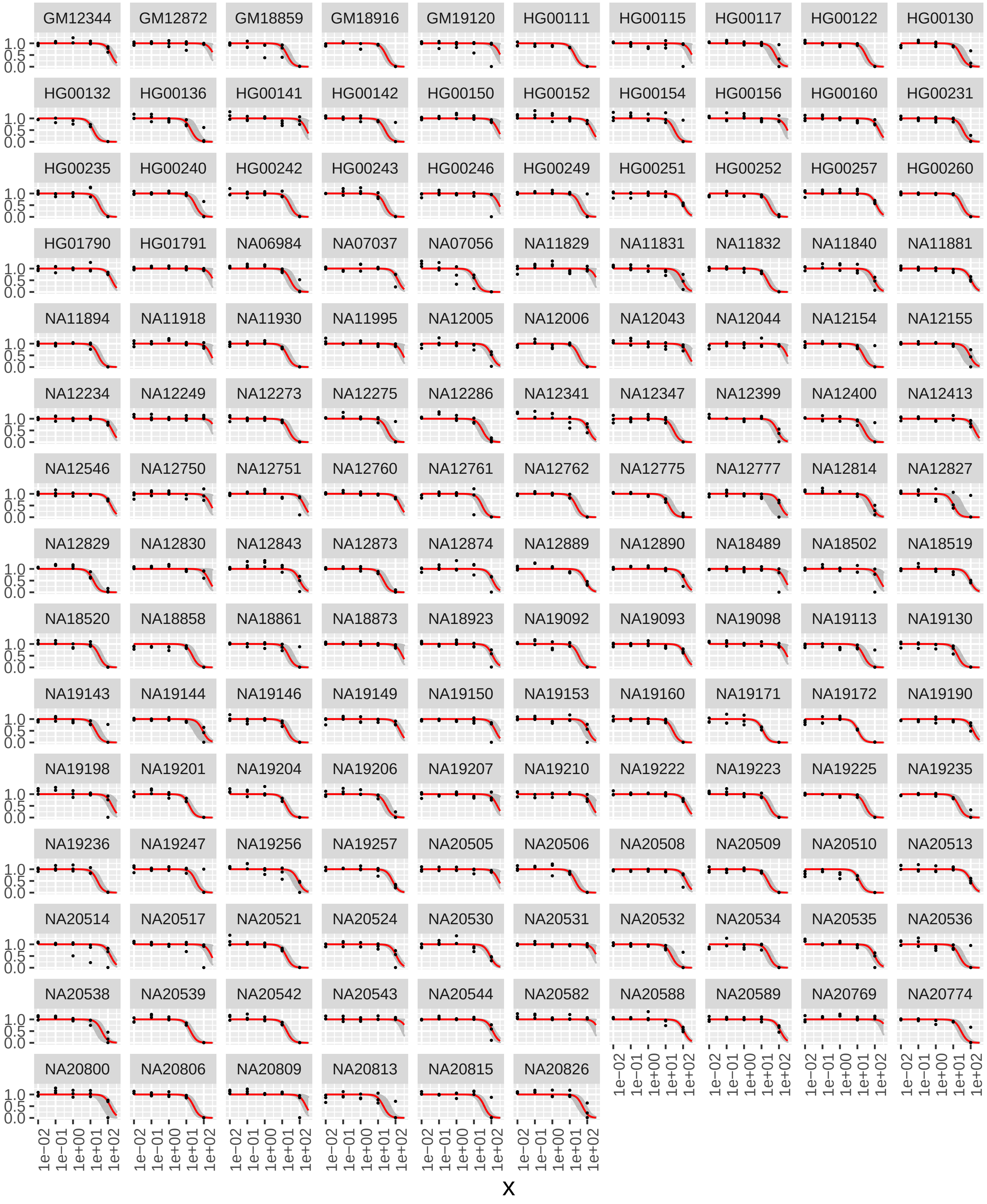

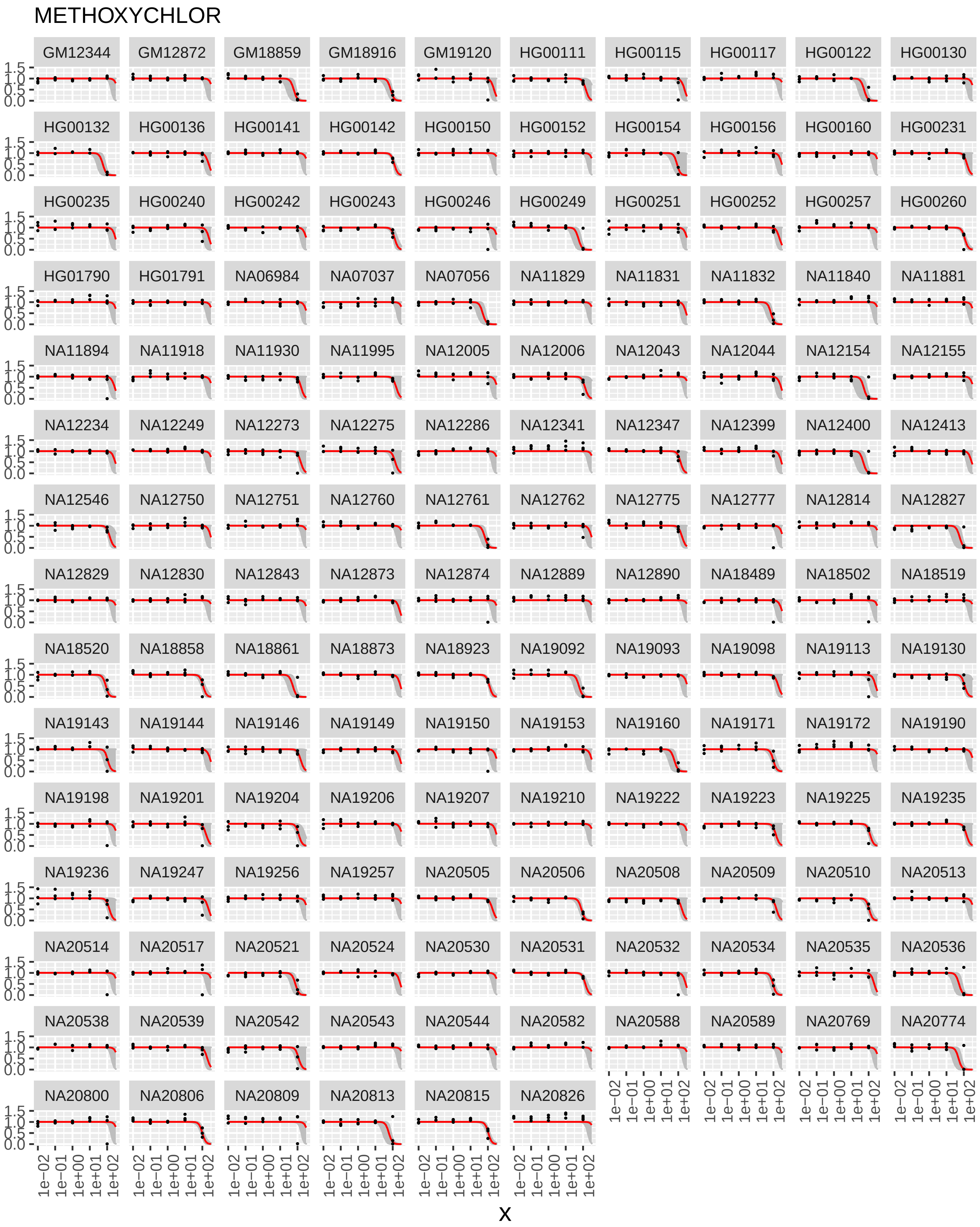
METHOXYCHLOR
GM12344
GM12872
GM18859
GM18916
GM19120
HG00111
HG00115
HG00117
HG00122
HG00130
HG00132
HG00136
HG00141
HG00142
HG00150
HG00152
HG00154
HG00156
HG00160
HG00231
HG00235
HG00240
HG00242
HG00243
HG00246
HG00249
HG00251
HG00252
HG00257
HG00260
HG01790
HG01791
NA06984
NA07037
NA07056
NA11829
NA11831
NA11832
NA11840
NA11881
NA11894
NA11918
NA11930
NA11995
NA12005
NA12006
NA12043
NA12044
NA12154
NA12155
NA12234
NA12249
NA12273
NA12275
NA12286
NA12341
NA12347
NA12399
NA12400
NA12413
NA12546
NA12750
NA12751
NA12760
NA12761
NA12762
NA12775
NA12777
NA12814
NA12827
NA12829
NA12830
NA12843
NA12873
NA12874
NA12889
NA12890
NA18489
NA18502
NA18519
NA18520
NA18858
NA18861
NA18873
NA18923
NA19092
NA19093
NA19098
NA19113
NA19130
NA19143
NA19144
NA19146
NA19149
NA19150
NA19153
NA19160
NA19171
NA19172
NA19190
NA19198
NA19201
NA19204
NA19206
NA19207
NA19210
NA19222
NA19223
NA19225
NA19235
NA19236
NA19247
NA19256
NA19257
NA20505
NA20506
NA20508
NA20509
NA20510
NA20513
NA20514
NA20517
NA20521
NA20524
NA20530
NA20531
NA20532
NA20534
NA20535
NA20536
NA20538
NA20539
NA20542
NA20543
NA20544
NA20582
NA20588
NA20589
NA20769
NA20774
NA20800
NA20806
NA20809
NA20813
NA20815
NA20826
1.5
1.0
0.5
0.0
1e−02
1e−01
1e+00
1e+01
1e+02
x

NAPHTHALENE

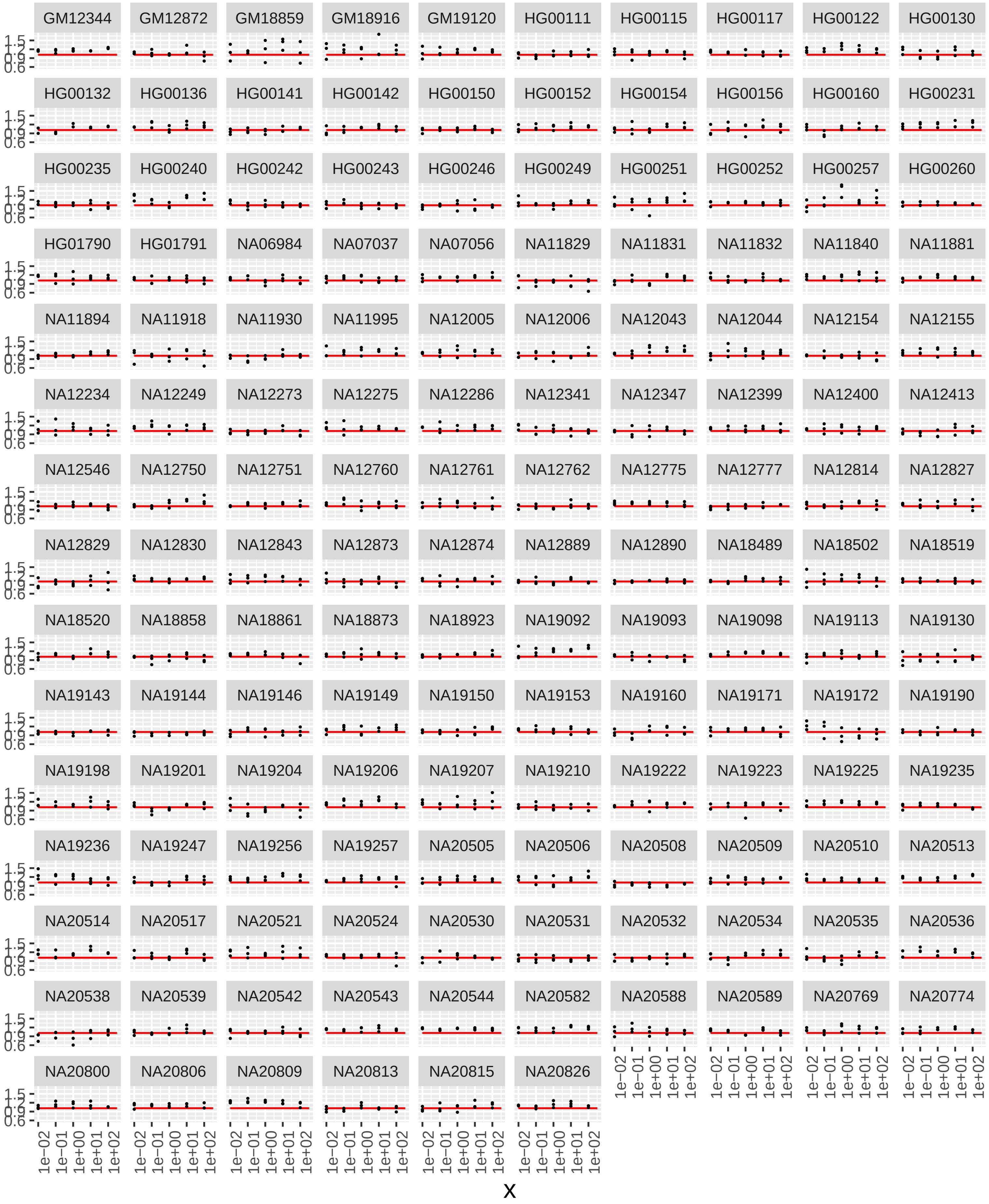

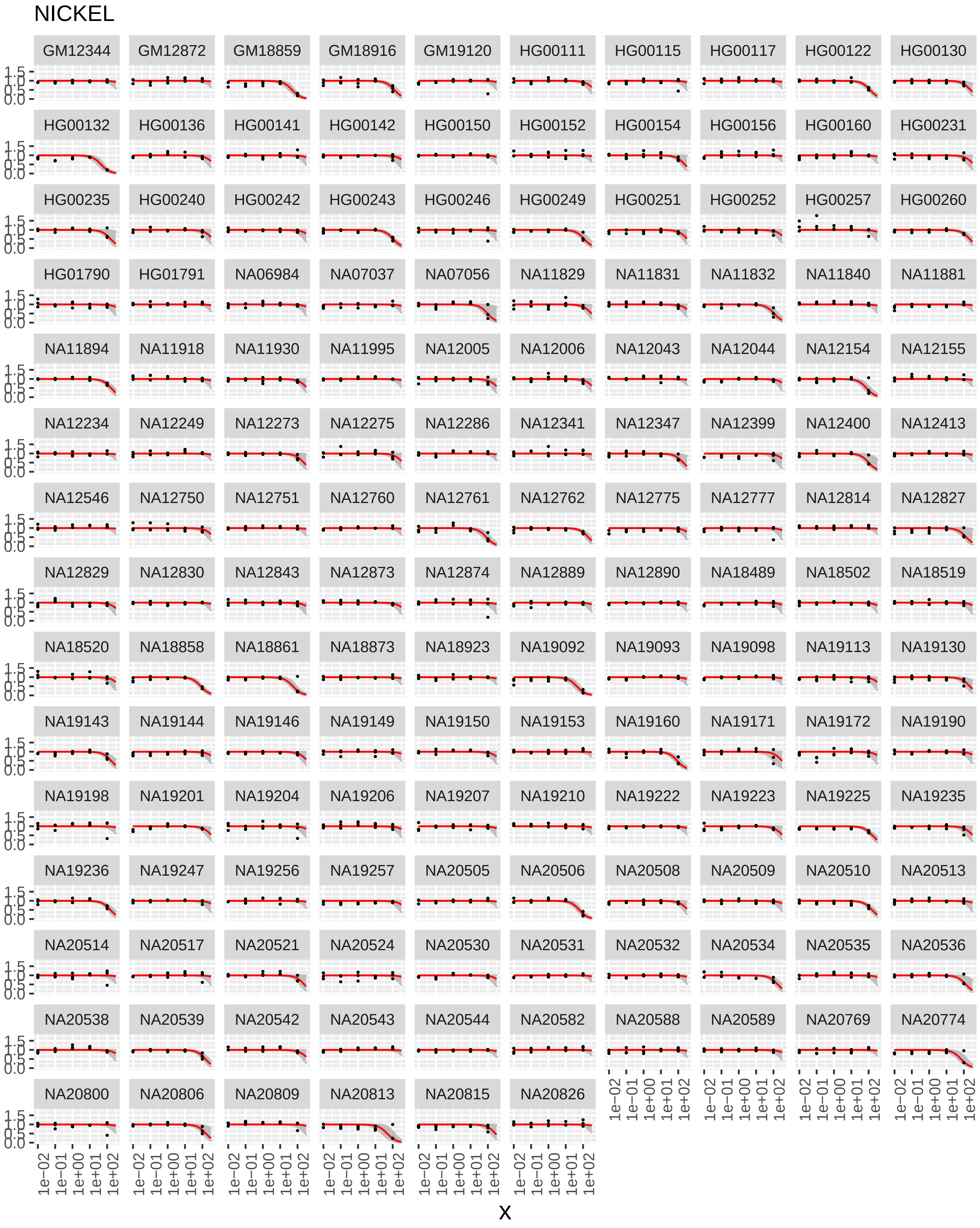
NICKEL
GM12344 GM12872 GM18859 GM18916 GM19120 HG00111 HG00115 HG00117 HG00122 HG00130
HG00132 HG00136 HG00141 HG00142 HG00150 HG00152 HG00154 HG00156 HG00160 HG00231
HG00235 HG00240 HG00242 HG00243 HG00246 HG00249 HG00251 HG00252 HG00257 HG00260
HG01790 HG01791 NA06984 NA07037 NA07056 NA11829 NA11831 NA11832 NA11840 NA11881
NA11894 NA11918 NA11930 NA11995 NA12005 NA12006 NA12043 NA12044 NA12154 NA12155
NA12234 NA12249 NA12273 NA12275 NA12286 NA12341 NA12347 NA12399 NA12400 NA12413
NA12546 NA12750 NA12751 NA12760 NA12761 NA12762 NA12775 NA12777 NA12814 NA12827
NA12829 NA12830 NA12843 NA12873 NA12874 NA12889 NA12890 NA18489 NA18502 NA18519
NA18520 NA18858 NA18861 NA18873 NA18923 NA19092 NA19093 NA19098 NA19113 NA19130
NA19143 NA19144 NA19146 NA19149 NA19150 NA19153 NA19160 NA19171 NA19172 NA19190
NA19198 NA19201 NA19204 NA19206 NA19207 NA19210 NA19222 NA19223 NA19225 NA19235
NA19236 NA19247 NA19256 NA19257 NA20505 NA20506 NA20508 NA20509 NA20510 NA20513
NA20514 NA20517 NA20521 NA20524 NA20530 NA20531 NA20532 NA20534 NA20535 NA20536
NA20538 NA20539 NA20542 NA20543 NA20544 NA20582 NA20588 NA20589 NA20769 NA20774
NA20800 NA20806 NA20809 NA20813 NA20815 NA20826
0.0 0.5 1.0 1.5
1e−02 1e−01 1e+00 1e+01 1e+02
x

PARATHION

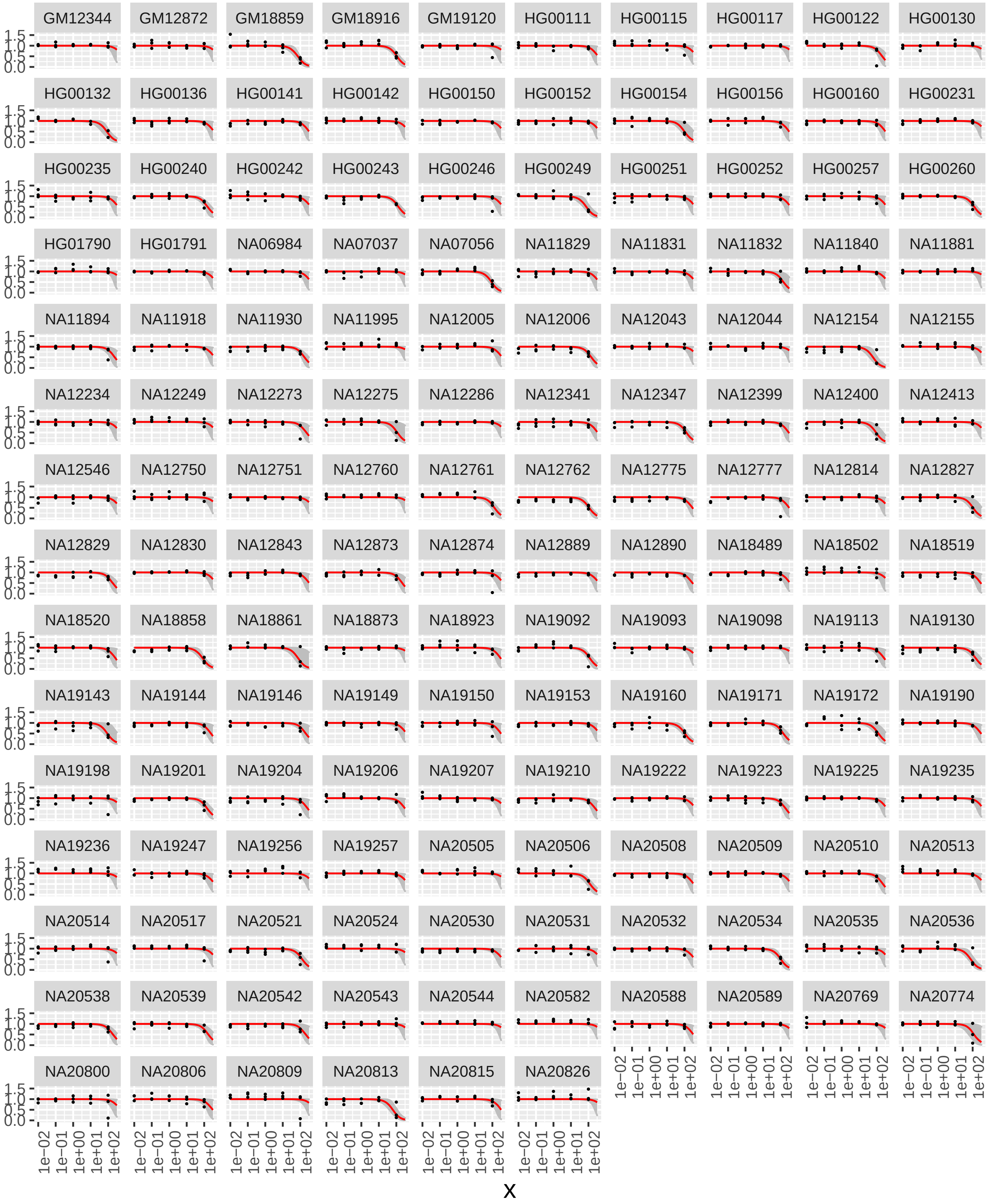
GM12344
GM12872
GM18859
GM18916
GM19120
HG00111
HG00115
HG00117
HG00122
HG00130
HG00132
HG00136
HG00141
HG00142
HG00150
HG00152
HG00154
HG00156
HG00160
HG00231
HG00235
HG00240
HG00242
HG00243
HG00246
HG00249
HG00251
HG00252
HG00257
HG00260
HG01790
HG01791
NA06984
NA07037
NA07056
NA11829
NA11831
NA11832
NA11840
NA11881
NA11894
NA11918
NA11930
NA11995
NA12005
NA12006
NA12043
NA12044
NA12154
NA12155
NA12234
NA12249
NA12273
NA12275
NA12286
NA12341
NA12347
NA12399
NA12400
NA12413
NA12546
NA12750
NA12751
NA12760
NA12761
NA12762
NA12775
NA12777
NA12814
NA12827
NA12829
NA12830
NA12843
NA12873
NA12874
NA12889
NA12890
NA18489
NA18502
NA18519
NA18520
NA18858
NA18861
NA18873
NA18923
NA19092
NA19093
NA19098
NA19113
NA19130
NA19143
NA19144
NA19146
NA19149
NA19150
NA19153
NA19160
NA19171
NA19172
NA19190
NA19198
NA19201
NA19204
NA19206
NA19207
NA19210
NA19222
NA19223
NA19225
NA19235
NA19236
NA19247
NA19256
NA19257
NA20505
NA20506
NA20508
NA20509
NA20510
NA20513
NA20514
NA20517
NA20521
NA20524
NA20530
NA20531
NA20532
NA20534
NA20535
NA20536
NA20538
NA20539
NA20542
NA20543
NA20544
NA20582
NA20588
NA20589
NA20769
NA20774
NA20800
NA20806
NA20809
NA20813
NA20815
NA20826
1.5
1.0
0.5
0.0
1e−02
1e−01
1e+00
1e+01
1e+02
x

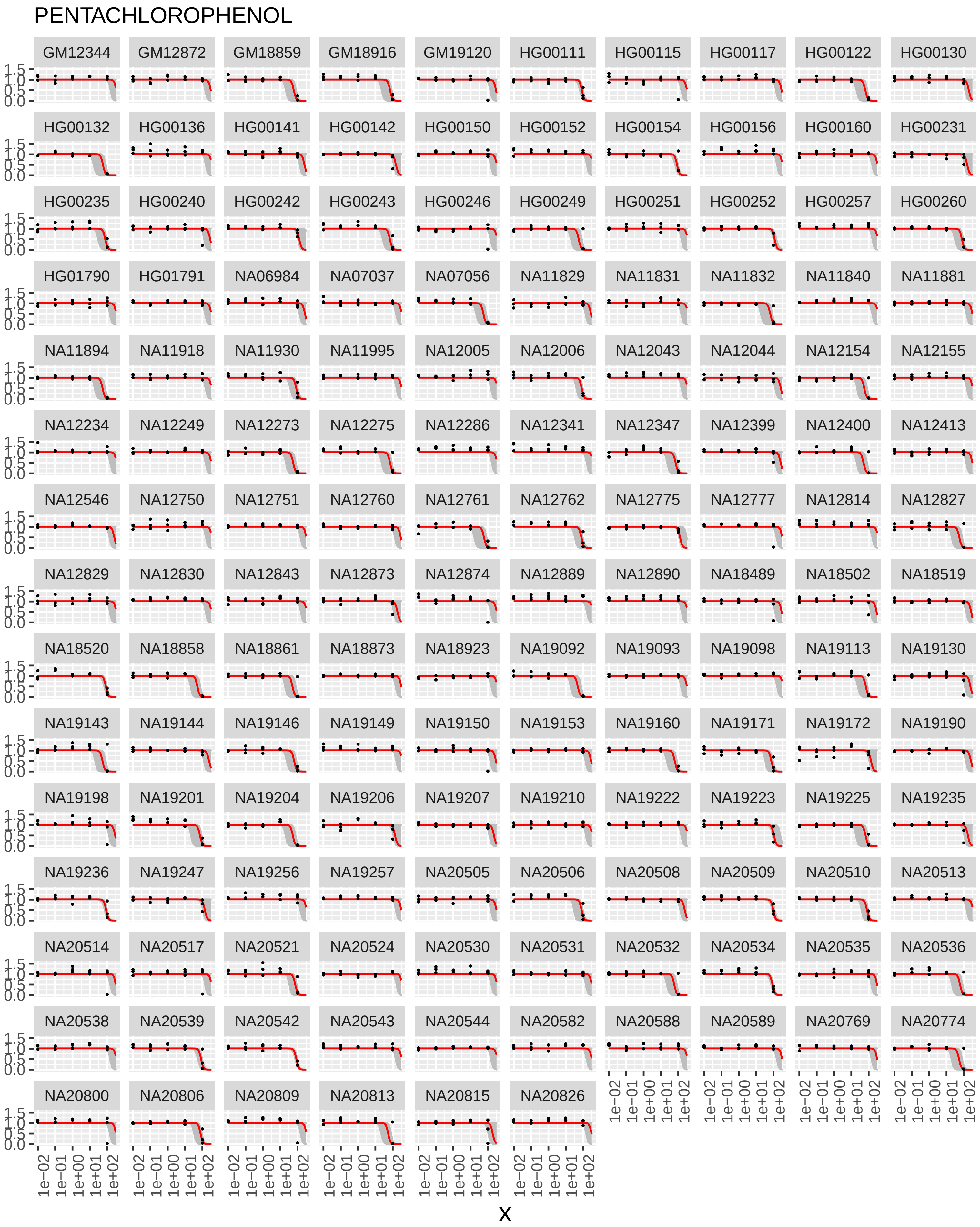
PENTACHLOROPHENOL
GM12344
GM12872
GM18859
GM18916
GM19120
HG00111
HG00115
HG00117
HG00122
HG00130
HG00132
HG00136
HG00141
HG00142
HG00150
HG00152
HG00154
HG00156
HG00160
HG00231
HG00235
HG00240
HG00242
HG00243
HG00246
HG00249
HG00251
HG00252
HG00257
HG00260
HG01790
HG01791
NA06984
NA07037
NA07056
NA11829
NA11831
NA11832
NA11840
NA11881
NA11894
NA11918
NA11930
NA11995
NA12005
NA12006
NA12043
NA12044
NA12154
NA12155
NA12234
NA12249
NA12273
NA12275
NA12286
NA12341
NA12347
NA12399
NA12400
NA12413
NA12546
NA12750
NA12751
NA12760
NA12761
NA12762
NA12775
NA12777
NA12814
NA12827
NA12829
NA12830
NA12843
NA12873
NA12874
NA12889
NA12890
NA18489
NA18502
NA18519
NA18520
NA18858
NA18861
NA18873
NA18923
NA19092
NA19093
NA19098
NA19113
NA19130
NA19143
NA19144
NA19146
NA19149
NA19150
NA19153
NA19160
NA19171
NA19172
NA19190
NA19198
NA19201
NA19204
NA19206
NA19207
NA19210
NA19222
NA19223
NA19225
NA19235
NA19236
NA19247
NA19256
NA19257
NA20505
NA20506
NA20508
NA20509
NA20510
NA20513
NA20514
NA20517
NA20521
NA20524
NA20530
NA20531
NA20532
NA20534
NA20535
NA20536
NA20538
NA20539
NA20542
NA20543
NA20544
NA20582
NA20588
NA20589
NA20769
NA20774
NA20800
NA20806
NA20809
NA20813
NA20815
NA20826
1.5
1.0
0.5
0.0
1e−02
1e−01
1e+00
1e+01
1e+02
x

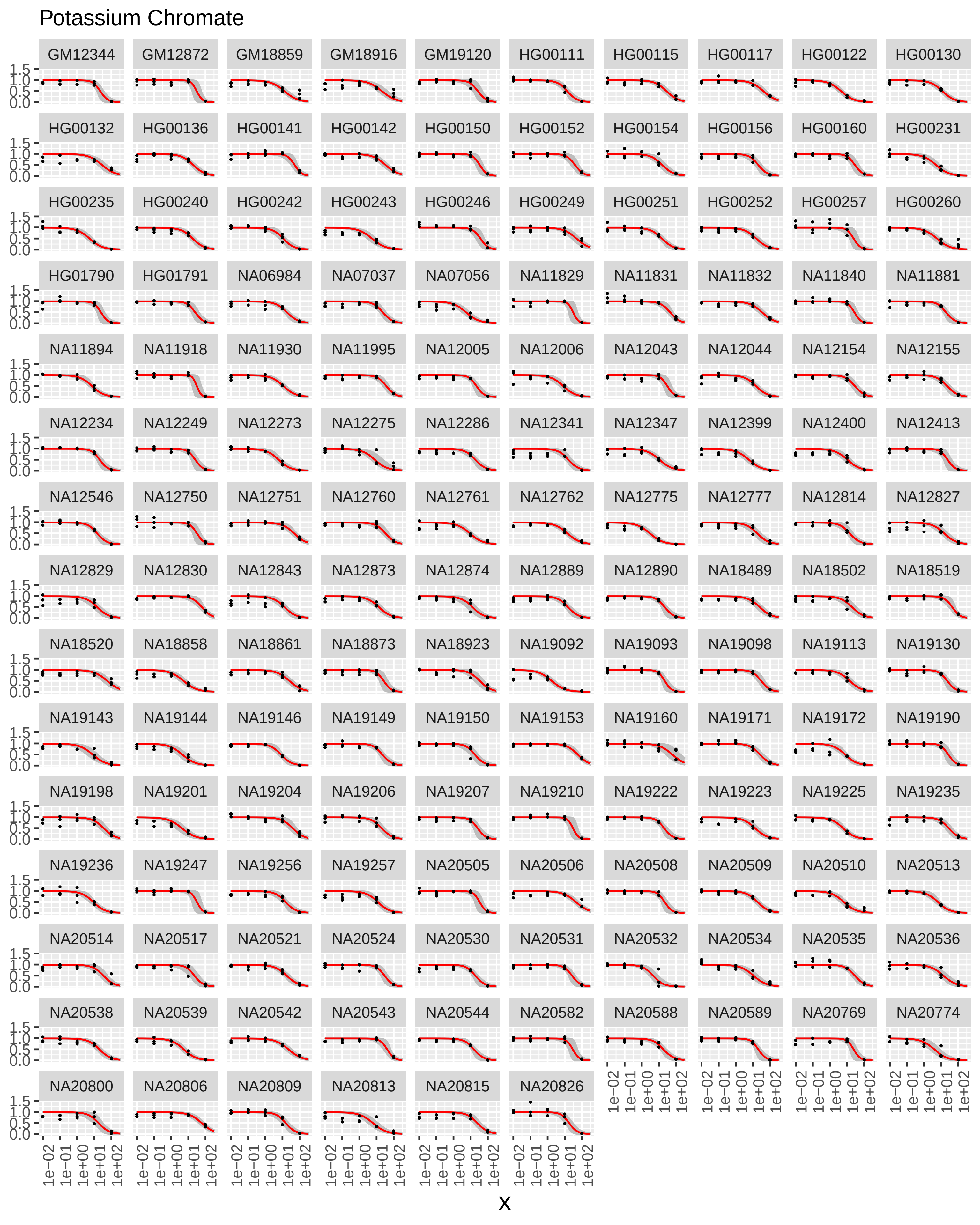

Potassium Chromate
GM12344 GM12872 GM18859 GM18916 GM19120 HG00111 HG00115 HG00117 HG00122 HG00130
HG00132 HG00136 HG00141 HG00142 HG00150 HG00152 HG00154 HG00156 HG00160 HG00231
HG00235 HG00240 HG00242 HG00243 HG00246 HG00249 HG00251 HG00252 HG00257 HG00260
HG01790 HG01791 NA06984 NA07037 NA07056 NA11829 NA11831 NA11832 NA11840 NA11881
NA11894 NA11918 NA11930 NA11995 NA12005 NA12006 NA12043 NA12044 NA12154 NA12155
NA12234 NA12249 NA12273 NA12275 NA12286 NA12341 NA12347 NA12399 NA12400 NA12413
NA12546 NA12750 NA12751 NA12760 NA12761 NA12762 NA12775 NA12777 NA12814 NA12827
NA12829 NA12830 NA12843 NA12873 NA12874 NA12889 NA12890 NA18489 NA18502 NA18519
NA18520 NA18858 NA18861 NA18873 NA18923 NA19092 NA19093 NA19098 NA19113 NA19130
NA19143 NA19144 NA19146 NA19149 NA19150 NA19153 NA19160 NA19171 NA19172 NA19190
NA19198 NA19201 NA19204 NA19206 NA19207 NA19210 NA19222 NA19223 NA19225 NA19235
NA19236 NA19247 NA19256 NA19257 NA20505 NA20506 NA20508 NA20509 NA20510 NA20513
NA20514 NA20517 NA20521 NA20524 NA20530 NA20531 NA20532 NA20534 NA20535 NA20536
NA20538 NA20539 NA20542 NA20543 NA20544 NA20582 NA20588 NA20589 NA20769 NA20774
NA20800 NA20806 NA20809 NA20813 NA20815 NA20826
0.0 0.5 1.0 1.5
1e−02 1e−01 1e+00 1e+01 1e+02
x

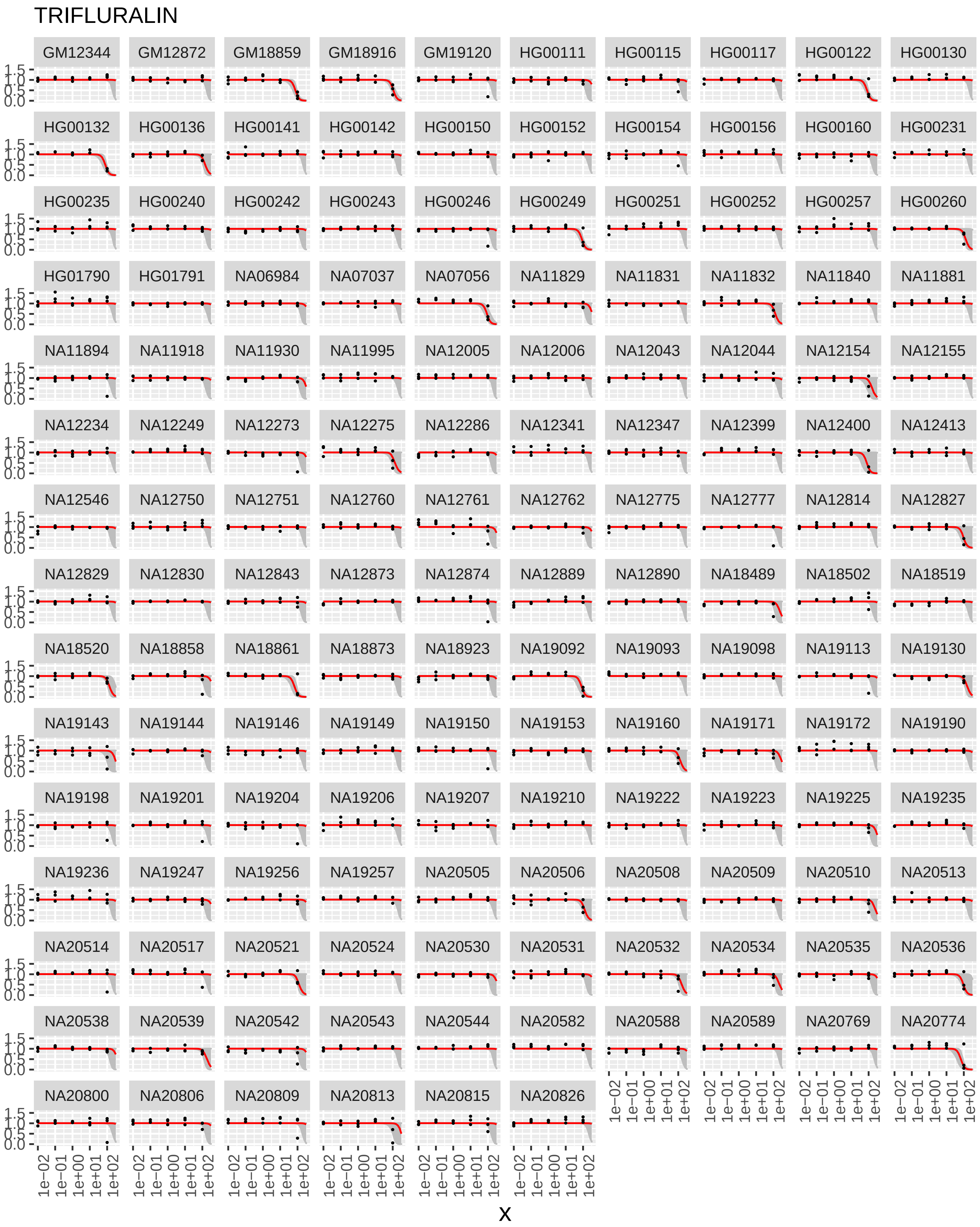
TRIFLURALIN
GM12344 GM12872 GM18859 GM18916 GM19120 HG00111 HG00115 HG00117 HG00122 HG00130
HG00132 HG00136 HG00141 HG00142 HG00150 HG00152 HG00154 HG00156 HG00160 HG00231
HG00235 HG00240 HG00242 HG00243 HG00246 HG00249 HG00251 HG00252 HG00257 HG00260
HG01790 HG01791 NA06984 NA07037 NA07056 NA11829 NA11831 NA11832 NA11840 NA11881
NA11894 NA11918 NA11930 NA11995 NA12005 NA12006 NA12043 NA12044 NA12154 NA12155
NA12234 NA12249 NA12273 NA12275 NA12286 NA12341 NA12347 NA12399 NA12400 NA12413
NA12546 NA12750 NA12751 NA12760 NA12761 NA12762 NA12775 NA12777 NA12814 NA12827
NA12829 NA12830 NA12843 NA12873 NA12874 NA12889 NA12890 NA18489 NA18502 NA18519
NA18520 NA18858 NA18861 NA18873 NA18923 NA19092 NA19093 NA19098 NA19113 NA19130
NA19143 NA19144 NA19146 NA19149 NA19150 NA19153 NA19160 NA19171 NA19172 NA19190
NA19198 NA19201 NA19204 NA19206 NA19207 NA19210 NA19222 NA19223 NA19225 NA19235
NA19236 NA19247 NA19256 NA19257 NA20505 NA20506 NA20508 NA20509 NA20510 NA20513
NA20514 NA20517 NA20521 NA20524 NA20530 NA20531 NA20532 NA20534 NA20535 NA20536
NA20538 NA20539 NA20542 NA20543 NA20544 NA20582 NA20588 NA20589 NA20769 NA20774
NA20800 NA20806 NA20809 NA20813 NA20815 NA20826
1.5
1.0
0.5
0.0
1e−02
1e−01
1e+00
1e+01
1e+02
x

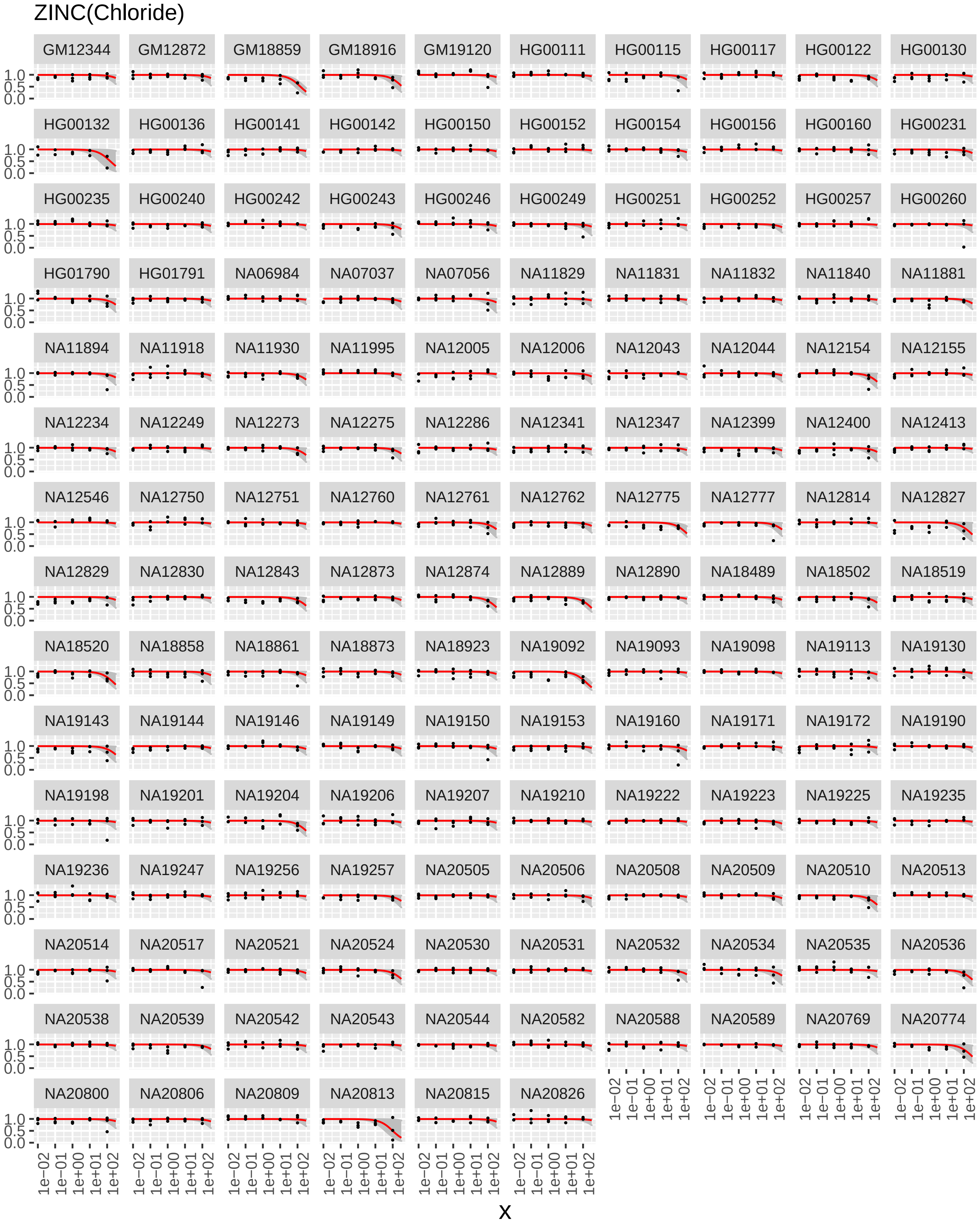
ZINC(Chloride)
GM12344
GM12872
GM18859
GM18916
GM19120
HG00111
HG00115
HG00117
HG00122
HG00130
HG00132
HG00136
HG00141
HG00142
HG00150
HG00152
HG00154
HG00156
HG00160
HG00231
HG00235
HG00240
HG00242
HG00243
HG00246
HG00249
HG00251
HG00252
HG00257
HG00260
HG01790
HG01791
NA06984
NA07037
NA07056
NA11829
NA11831
NA11832
NA11840
NA11881
NA11894
NA11918
NA11930
NA11995
NA12005
NA12006
NA12043
NA12044
NA12154
NA12155
NA12234
NA12249
NA12273
NA12275
NA12286
NA12341
NA12347
NA12399
NA12400
NA12413
NA12546
NA12750
NA12751
NA12760
NA12761
NA12762
NA12775
NA12777
NA12814
NA12827
NA12829
NA12830
NA12843
NA12873
NA12874
NA12889
NA12890
NA18489
NA18502
NA18519
NA18520
NA18858
NA18861
NA18873
NA18923
NA19092
NA19093
NA19098
NA19113
NA19130
NA19143
NA19144
NA19146
NA19149
NA19150
NA19153
NA19160
NA19171
NA19172
NA19190
NA19198
NA19201
NA19204
NA19206
NA19207
NA19210
NA19222
NA19223
NA19225
NA19235
NA19236
NA19247
NA19256
NA19257
NA20505
NA20506
NA20508
NA20509
NA20510
NA20513
NA20514
NA20517
NA20521
NA20524
NA20530
NA20531
NA20532
NA20534
NA20535
NA20536
NA20538
NA20539
NA20542
NA20543
NA20544
NA20582
NA20588
NA20589
NA20769
NA20774
NA20800
NA20806
NA20809
NA20813
NA20815
NA20826
1.0
0.5
0.0
1e−02
1e−01
1e+00
1e+01
1e+02
x

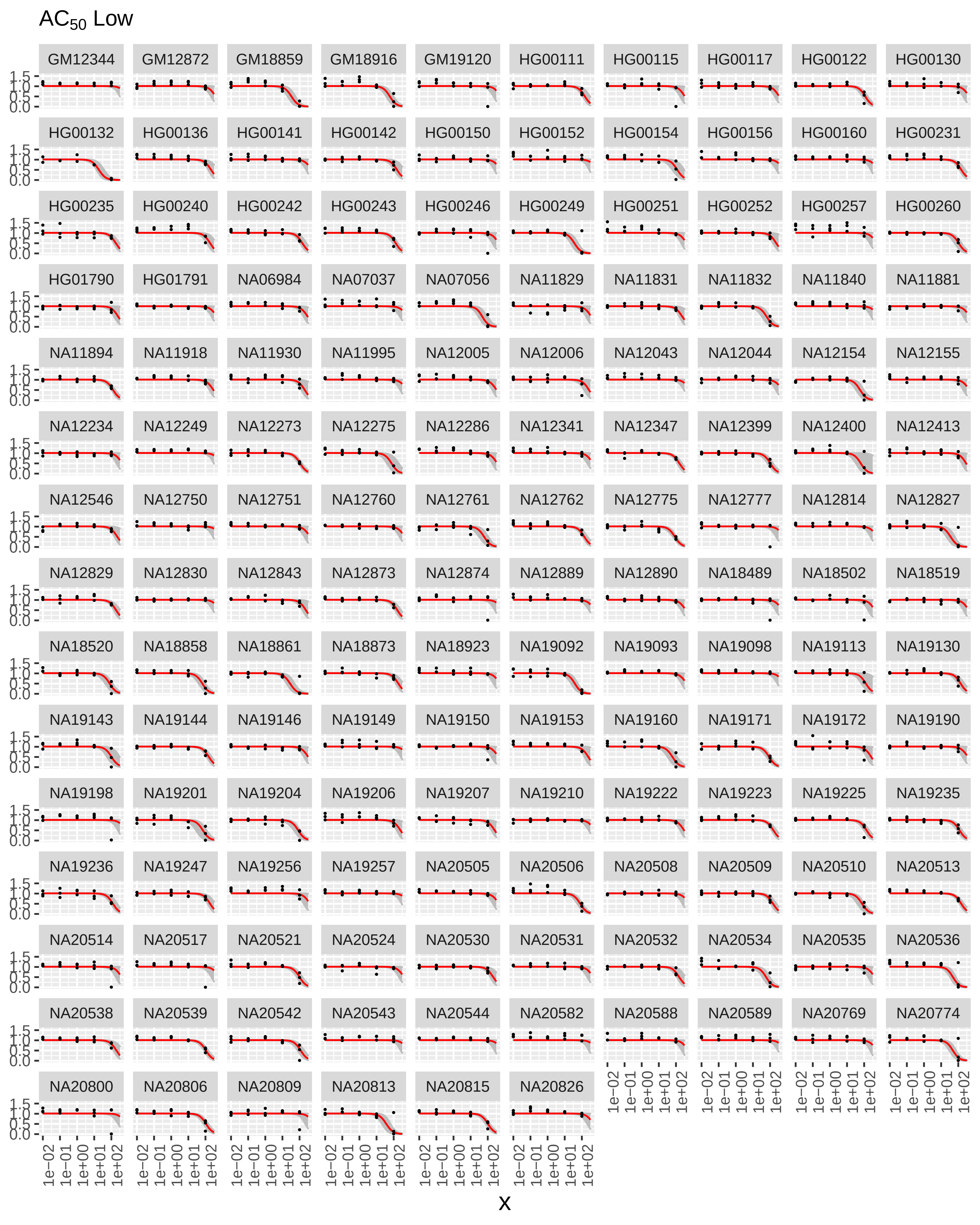

AC50 Low
GM12344
GM12872
GM18859
GM18916
GM19120
HG00111
HG00115
HG00117
HG00122
HG00130
HG00132
HG00136
HG00141
HG00142
HG00150
HG00152
HG00154
HG00156
HG00160
HG00231
HG00235
HG00240
HG00242
HG00243
HG00246
HG00249
HG00251
HG00252
HG00257
HG00260
HG01790
HG01791
NA06984
NA07037
NA07056
NA11829
NA11831
NA11832
NA11840
NA11881
NA11894
NA11918
NA11930
NA11995
NA12005
NA12006
NA12043
NA12044
NA12154
NA12155
NA12234
NA12249
NA12273
NA12275
NA12286
NA12341
NA12347
NA12399
NA12400
NA12413
NA12546
NA12750
NA12751
NA12760
NA12761
NA12762
NA12775
NA12777
NA12814
NA12827
NA12829
NA12830
NA12843
NA12873
NA12874
NA12889
NA12890
NA18489
NA18502
NA18519
NA18520
NA18858
NA18861
NA18873
NA18923
NA19092
NA19093
NA19098
NA19113
NA19130
NA19143
NA19144
NA19146
NA19149
NA19150
NA19153
NA19160
NA19171
NA19172
NA19190
NA19198
NA19201
NA19204
NA19206
NA19207
NA19210
NA19222
NA19223
NA19225
NA19235
NA19236
NA19247
NA19256
NA19257
NA20505
NA20506
NA20508
NA20509
NA20510
NA20513
NA20514
NA20517
NA20521
NA20524
NA20530
NA20531
NA20532
NA20534
NA20535
NA20536
NA20538
NA20539
NA20542
NA20543
NA20544
NA20582
NA20588
NA20589
NA20769
NA20774
NA20800
NA20806
NA20809
NA20813
NA20815
NA20826
1.5
1.0
0.5
0.0
1e−02
1e−01
1e+00
1e+01
1e+02
x

AC_{50} High

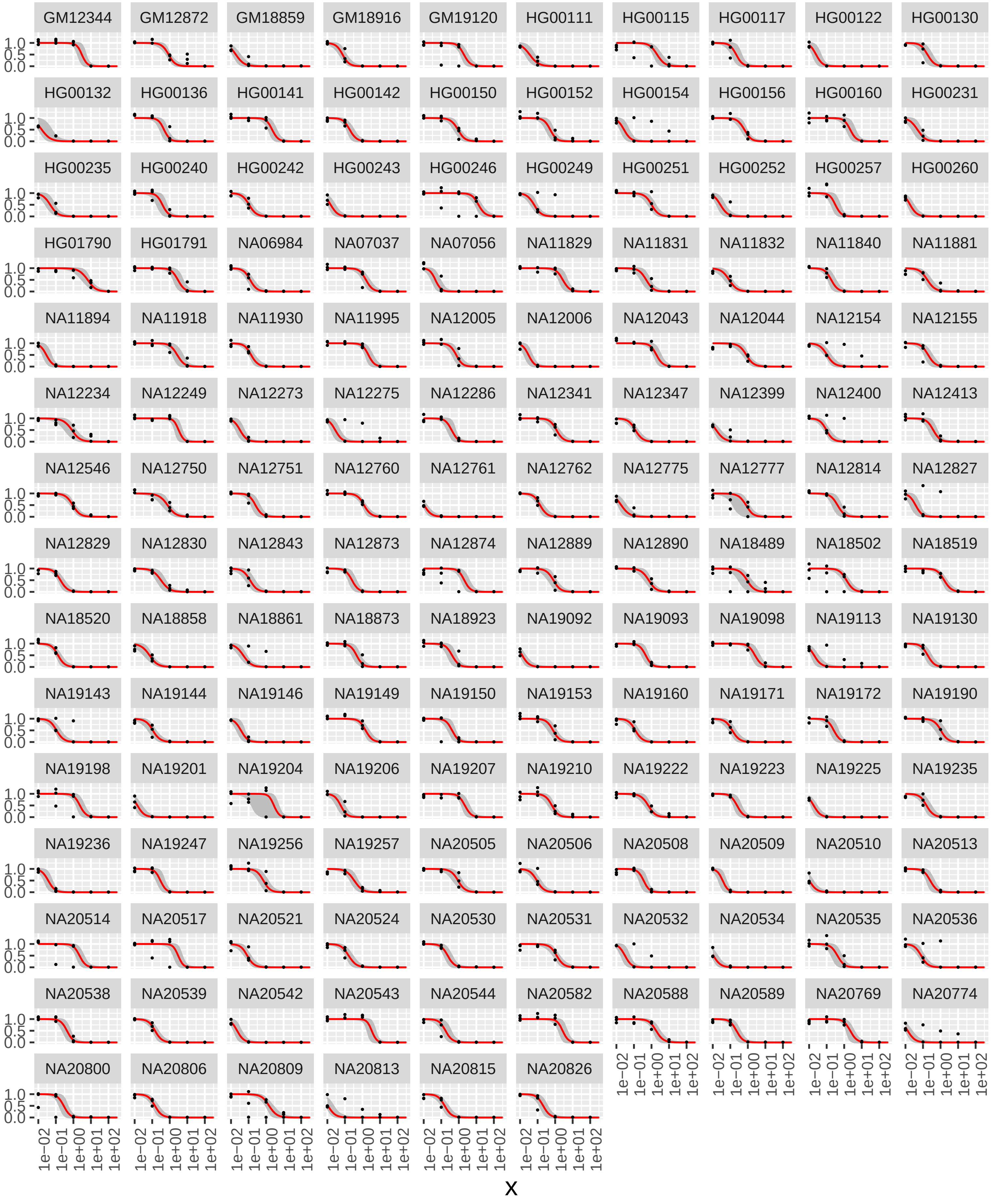
GM12344
GM12872
GM18859
GM18916
GM19120
HG00111
HG00115
HG00117
HG00122
HG00130
HG00132
HG00136
HG00141
HG00142
HG00150
HG00152
HG00154
HG00156
HG00160
HG00231
HG00235
HG00240
HG00242
HG00243
HG00246
HG00249
HG00251
HG00252
HG00257
HG00260
HG01790
HG01791
NA06984
NA07037
NA07056
NA11829
NA11831
NA11832
NA11840
NA11881
NA11894
NA11918
NA11930
NA11995
NA12005
NA12006
NA12043
NA12044
NA12154
NA12155
NA12234
NA12249
NA12273
NA12275
NA12286
NA12341
NA12347
NA12399
NA12400
NA12413
NA12546
NA12750
NA12751
NA12760
NA12761
NA12762
NA12775
NA12777
NA12814
NA12827
NA12829
NA12830
NA12843
NA12873
NA12874
NA12889
NA12890
NA18489
NA18502
NA18519
NA18520
NA18858
NA18861
NA18873
NA18923
NA19092
NA19093
NA19098
NA19113
NA19130
NA19143
NA19144
NA19146
NA19149
NA19150
NA19153
NA19160
NA19171
NA19172
NA19190
NA19198
NA19201
NA19204
NA19206
NA19207
NA19210
NA19222
NA19223
NA19225
NA19235
NA19236
NA19247
NA19256
NA19257
NA20505
NA20506
NA20508
NA20509
NA20510
NA20513
NA20514
NA20517
NA20521
NA20524
NA20530
NA20531
NA20532
NA20534
NA20535
NA20536
NA20538
NA20539
NA20542
NA20543
NA20544
NA20582
NA20588
NA20589
NA20769
NA20774
NA20800
NA20806
NA20809
NA20813
NA20815
NA20826
1.0
0.5
0.0
1e−02
1e−01
1e+00
1e+01
1e+02
x

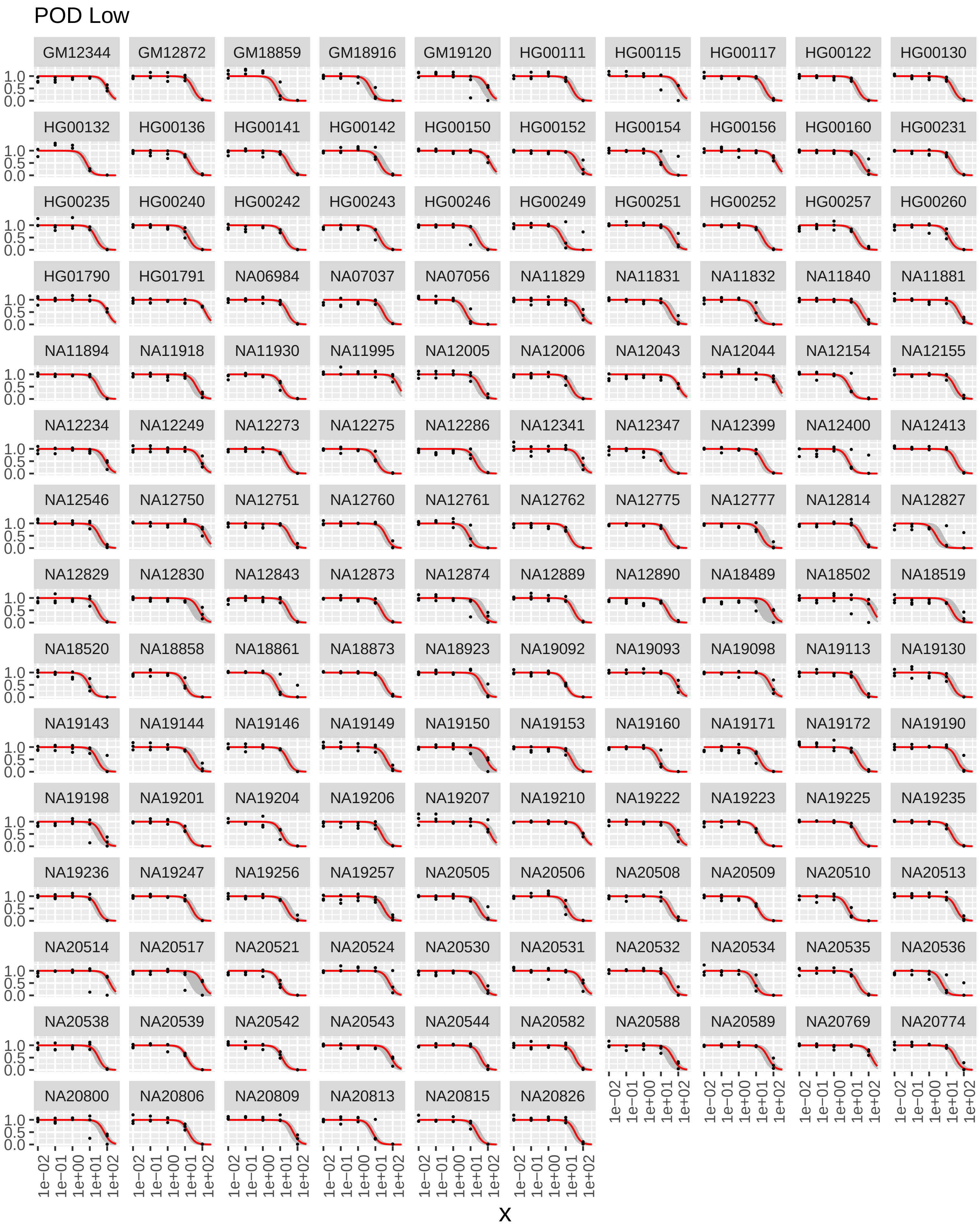

POD Low
GM12344
GM12872
GM18859
GM18916
GM19120
HG00111
HG00115
HG00117
HG00122
HG00130
HG00132
HG00136
HG00141
HG00142
HG00150
HG00152
HG00154
HG00156
HG00160
HG00231
HG00235
HG00240
HG00242
HG00243
HG00246
HG00249
HG00251
HG00252
HG00257
HG00260
HG01790
HG01791
NA06984
NA07037
NA07056
NA11829
NA11831
NA11832
NA11840
NA11881
NA11894
NA11918
NA11930
NA11995
NA12005
NA12006
NA12043
NA12044
NA12154
NA12155
NA12234
NA12249
NA12273
NA12275
NA12286
NA12341
NA12347
NA12399
NA12400
NA12413
NA12546
NA12750
NA12751
NA12760
NA12761
NA12762
NA12775
NA12777
NA12814
NA12827
NA12829
NA12830
NA12843
NA12873
NA12874
NA12889
NA12890
NA18489
NA18502
NA18519
NA18520
NA18858
NA18861
NA18873
NA18923
NA19092
NA19093
NA19098
NA19113
NA19130
NA19143
NA19144
NA19146
NA19149
NA19150
NA19153
NA19160
NA19171
NA19172
NA19190
NA19198
NA19201
NA19204
NA19206
NA19207
NA19210
NA19222
NA19223
NA19225
NA19235
NA19236
NA19247
NA19256
NA19257
NA20505
NA20506
NA20508
NA20509
NA20510
NA20513
NA20514
NA20517
NA20521
NA20524
NA20530
NA20531
NA20532
NA20534
NA20535
NA20536
NA20538
NA20539
NA20542
NA20543
NA20544
NA20582
NA20588
NA20589
NA20769
NA20774
NA20800
NA20806
NA20809
NA20813
NA20815
NA20826
1.0
0.5
0.0
1e−02
1e−01
1e+00
1e+01
1e+02
x

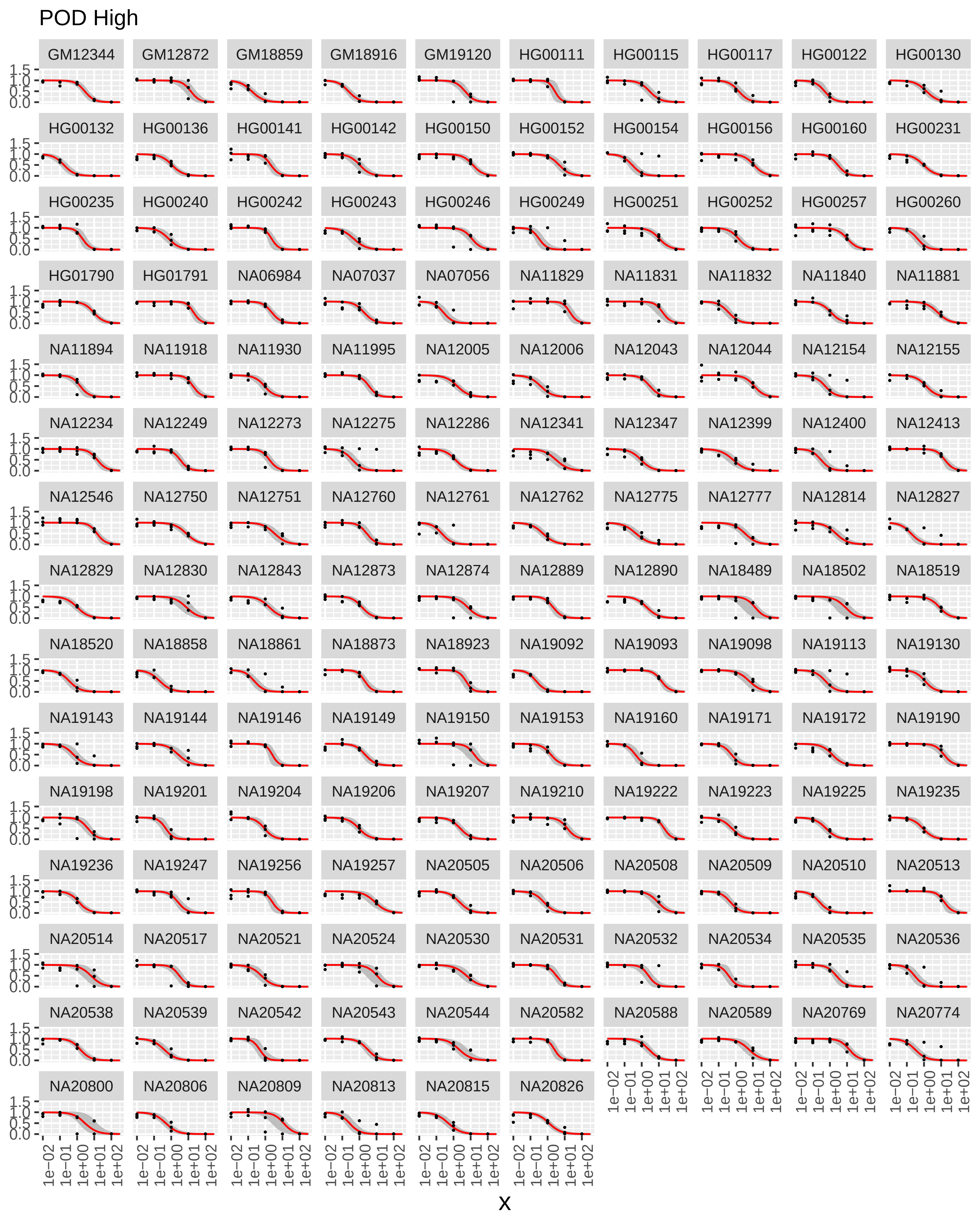
POD High
GM12344
GM12872
GM18859
GM18916
GM19120
HG00111
HG00115
HG00117
HG00122
HG00130
HG00132
HG00136
HG00141
HG00142
HG00150
HG00152
HG00154
HG00156
HG00160
HG00231
HG00235
HG00240
HG00242
HG00243
HG00246
HG00249
HG00251
HG00252
HG00257
HG00260
HG01790
HG01791
NA06984
NA07037
NA07056
NA11829
NA11831
NA11832
NA11840
NA11881
NA11894
NA11918
NA11930
NA11995
NA12005
NA12006
NA12043
NA12044
NA12154
NA12155
NA12234
NA12249
NA12273
NA12275
NA12286
NA12341
NA12347
NA12399
NA12400
NA12413
NA12546
NA12750
NA12751
NA12760
NA12761
NA12762
NA12775
NA12777
NA12814
NA12827
NA12829
NA12830
NA12843
NA12873
NA12874
NA12889
NA12890
NA18489
NA18502
NA18519
NA18520
NA18858
NA18861
NA18873
NA18923
NA19092
NA19093
NA19098
NA19113
NA19130
NA19143
NA19144
NA19146
NA19149
NA19150
NA19153
NA19160
NA19171
NA19172
NA19190
NA19198
NA19201
NA19204
NA19206
NA19207
NA19210
NA19222
NA19223
NA19225
NA19235
NA19236
NA19247
NA19256
NA19257
NA20505
NA20506
NA20508
NA20509
NA20510
NA20513
NA20514
NA20517
NA20521
NA20524
NA20530
NA20531
NA20532
NA20534
NA20535
NA20536
NA20538
NA20539
NA20542
NA20543
NA20544
NA20582
NA20588
NA20589
NA20769
NA20774
NA20800
NA20806
NA20809
NA20813
NA20815
NA20826
1.5
1.0
0.5
0.0
1e−02
1e−01
1e+00
1e+01
1e+02
x

Expo Low

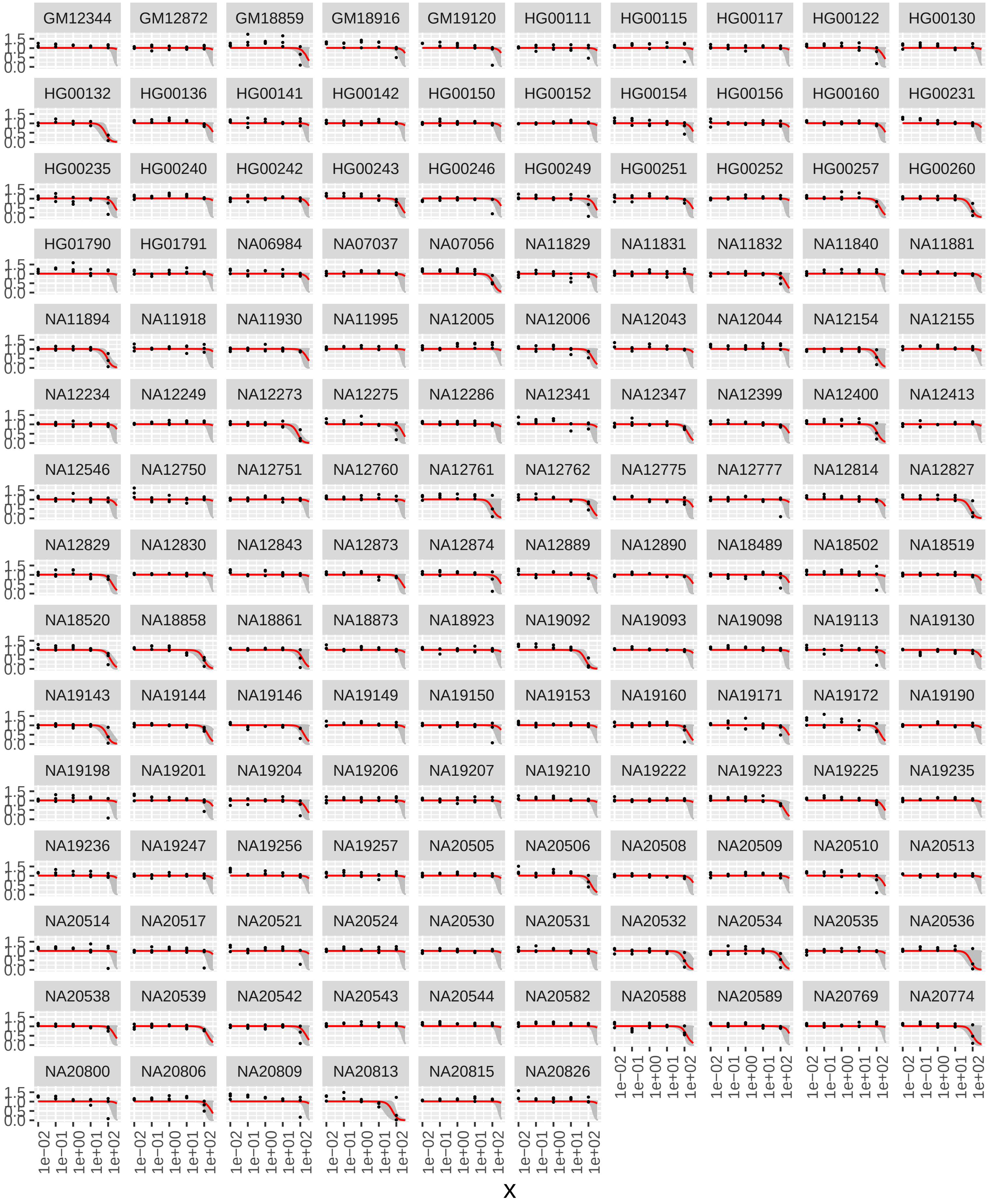

GM12344
GM12872
GM18859
GM18916
GM19120
HG00111
HG00115
HG00117
HG00122
HG00130
HG00132
HG00136
HG00141
HG00142
HG00150
HG00152
HG00154
HG00156
HG00160
HG00231
HG00235
HG00240
HG00242
HG00243
HG00246
HG00249
HG00251
HG00252
HG00257
HG00260
HG01790
HG01791
NA06984
NA07037
NA07056
NA11829
NA11831
NA11832
NA11840
NA11881
NA11894
NA11918
NA11930
NA11995
NA12005
NA12006
NA12043
NA12044
NA12154
NA12155
NA12234
NA12249
NA12273
NA12275
NA12286
NA12341
NA12347
NA12399
NA12400
NA12413
NA12546
NA12750
NA12751
NA12760
NA12761
NA12762
NA12775
NA12777
NA12814
NA12827
NA12829
NA12830
NA12843
NA12873
NA12874
NA12889
NA12890
NA18489
NA18502
NA18519
NA18520
NA18858
NA18861
NA18873
NA18923
NA19092
NA19093
NA19098
NA19113
NA19130
NA19143
NA19144
NA19146
NA19149
NA19150
NA19153
NA19160
NA19171
NA19172
NA19190
NA19198
NA19201
NA19204
NA19206
NA19207
NA19210
NA19222
NA19223
NA19225
NA19235
NA19236
NA19247
NA19256
NA19257
NA20505
NA20506
NA20508
NA20509
NA20510
NA20513
NA20514
NA20517
NA20521
NA20524
NA20530
NA20531
NA20532
NA20534
NA20535
NA20536
NA20538
NA20539
NA20542
NA20543
NA20544
NA20582
NA20588
NA20589
NA20769
NA20774
NA20800
NA20806
NA20809
NA20813
NA20815
NA20826
1.5
1.0
0.5
0.0
1e−02
1e−01
1e+00
1e+01
1e+02
x

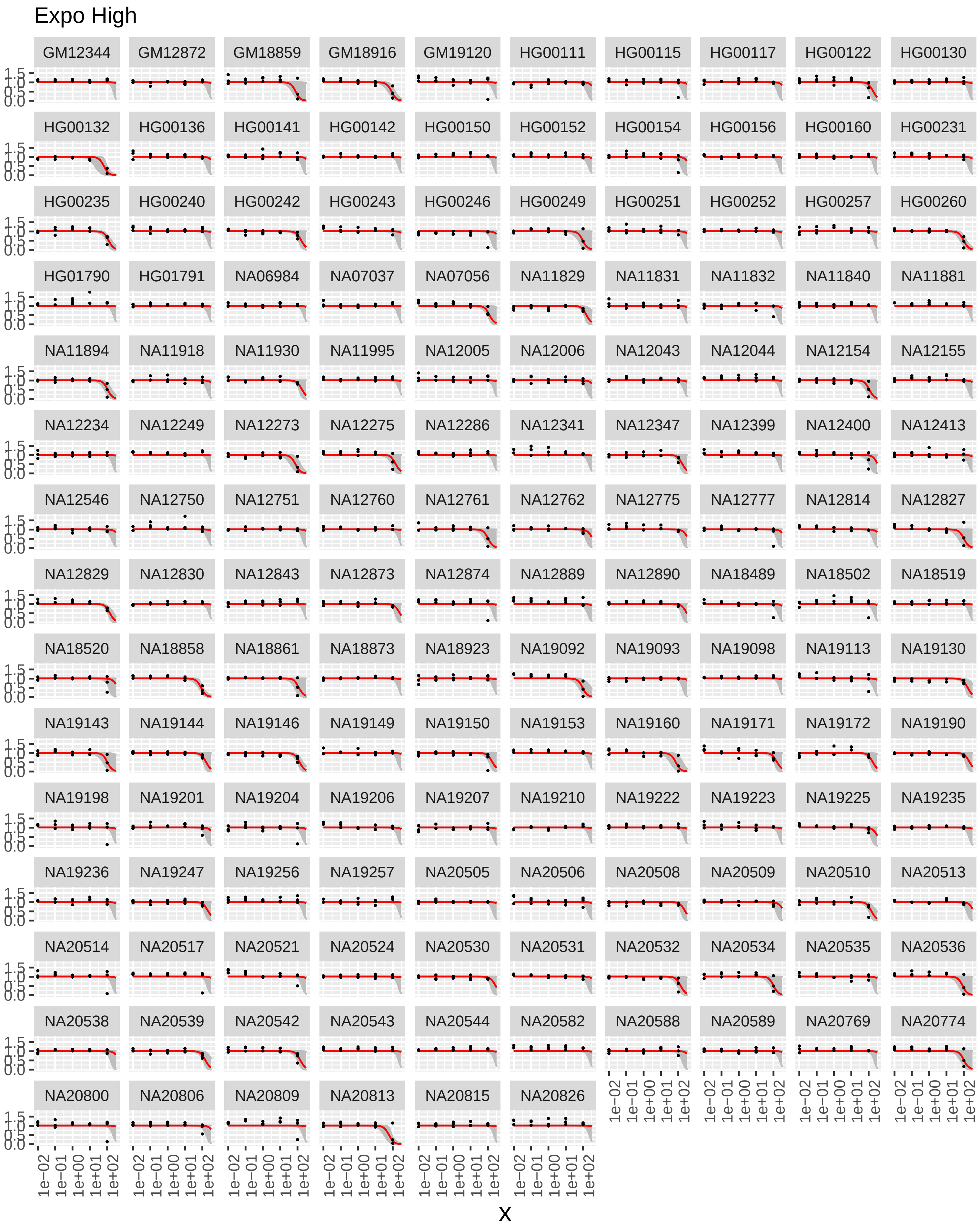

Expo High
GM12344
GM12872
GM18859
GM18916
GM19120
HG00111
HG00115
HG00117
HG00122
HG00130
HG00132
HG00136
HG00141
HG00142
HG00150
HG00152
HG00154
HG00156
HG00160
HG00231
HG00235
HG00240
HG00242
HG00243
HG00246
HG00249
HG00251
HG00252
HG00257
HG00260
HG01790
HG01791
NA06984
NA07037
NA07056
NA11829
NA11831
NA11832
NA11840
NA11881
NA11894
NA11918
NA11930
NA11995
NA12005
NA12006
NA12043
NA12044
NA12154
NA12155
NA12234
NA12249
NA12273
NA12275
NA12286
NA12341
NA12347
NA12399
NA12400
NA12413
NA12546
NA12750
NA12751
NA12760
NA12761
NA12762
NA12775
NA12777
NA12814
NA12827
NA12829
NA12830
NA12843
NA12873
NA12874
NA12889
NA12890
NA18489
NA18502
NA18519
NA18520
NA18858
NA18861
NA18873
NA18923
NA19092
NA19093
NA19098
NA19113
NA19130
NA19143
NA19144
NA19146
NA19149
NA19150
NA19153
NA19160
NA19171
NA19172
NA19190
NA19198
NA19201
NA19204
NA19206
NA19207
NA19210
NA19222
NA19223
NA19225
NA19235
NA19236
NA19247
NA19256
NA19257
NA20505
NA20506
NA20508
NA20509
NA20510
NA20513
NA20514
NA20517
NA20521
NA20524
NA20530
NA20531
NA20532
NA20534
NA20535
NA20536
NA20538
NA20539
NA20542
NA20543
NA20544
NA20582
NA20588
NA20589
NA20769
NA20774
NA20800
NA20806
NA20809
NA20813
NA20815
NA20826
1.5
1.0
0.5
0.0
1e−02
1e−01
1e+00
1e+01
1e+02
x

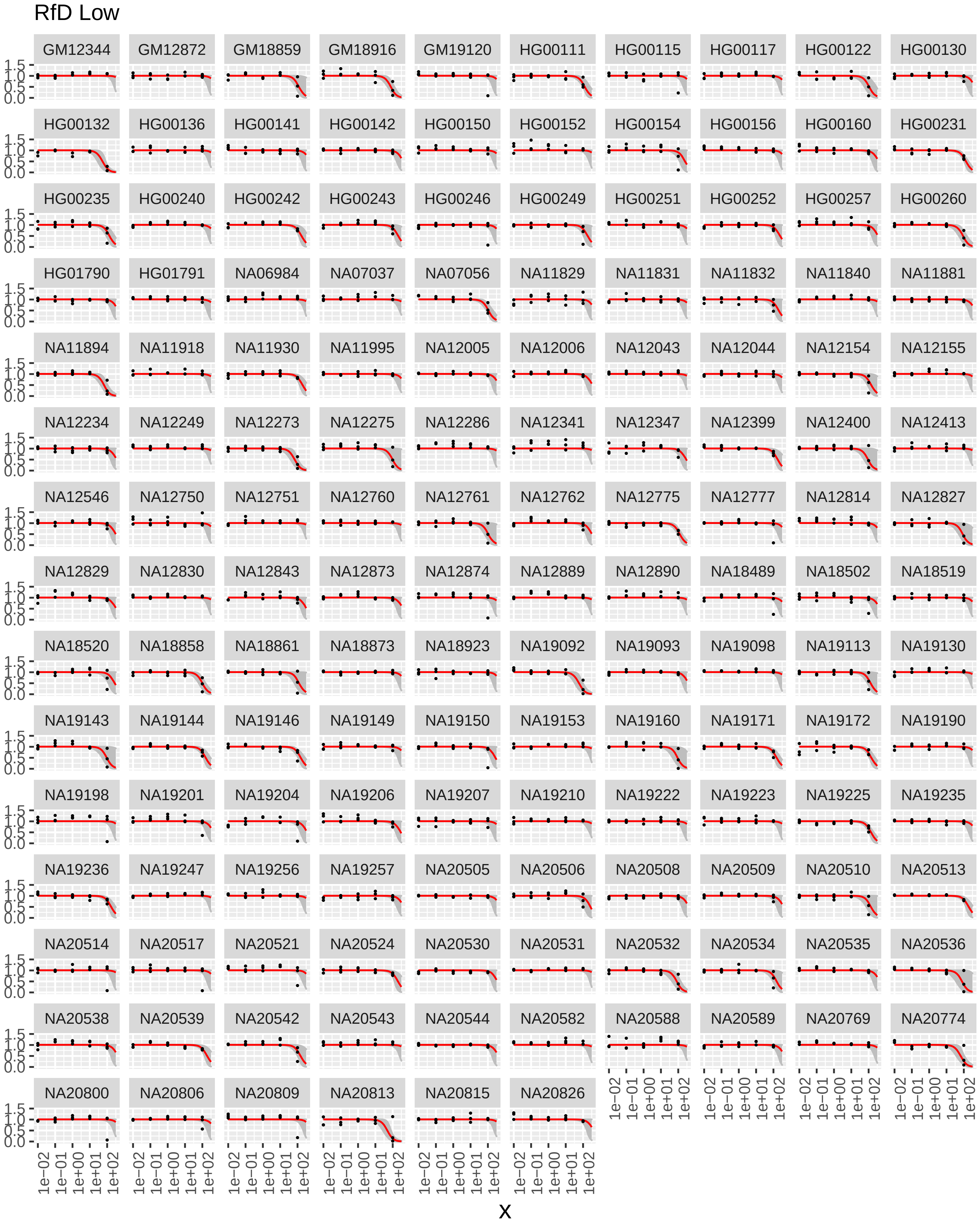
RfD Low
GM12344 GM12872 GM18859 GM18916 GM19120 HG00111 HG00115 HG00117 HG00122 HG00130
HG00132 HG00136 HG00141 HG00142 HG00150 HG00152 HG00154 HG00156 HG00160 HG00231
HG00235 HG00240 HG00242 HG00243 HG00246 HG00249 HG00251 HG00252 HG00257 HG00260
HG01790 HG01791 NA06984 NA07037 NA07056 NA11829 NA11831 NA11832 NA11840 NA11881
NA11894 NA11918 NA11930 NA11995 NA12005 NA12006 NA12043 NA12044 NA12154 NA12155
NA12234 NA12249 NA12273 NA12275 NA12286 NA12341 NA12347 NA12399 NA12400 NA12413
NA12546 NA12750 NA12751 NA12760 NA12761 NA12762 NA12775 NA12777 NA12814 NA12827
NA12829 NA12830 NA12843 NA12873 NA12874 NA12889 NA12890 NA18489 NA18502 NA18519
NA18520 NA18858 NA18861 NA18873 NA18923 NA19092 NA19093 NA19098 NA19113 NA19130
NA19143 NA19144 NA19146 NA19149 NA19150 NA19153 NA19160 NA19171 NA19172 NA19190
NA19198 NA19201 NA19204 NA19206 NA19207 NA19210 NA19222 NA19223 NA19225 NA19235
NA19236 NA19247 NA19256 NA19257 NA20505 NA20506 NA20508 NA20509 NA20510 NA20513
NA20514 NA20517 NA20521 NA20524 NA20530 NA20531 NA20532 NA20534 NA20535 NA20536
NA20538 NA20539 NA20542 NA20543 NA20544 NA20582 NA20588 NA20589 NA20769 NA20774
NA20800 NA20806 NA20809 NA20813 NA20815 NA20826
0.0 0.5 1.0 1.5
1e−02 1e−01 1e+00 1e+01 1e+02
x

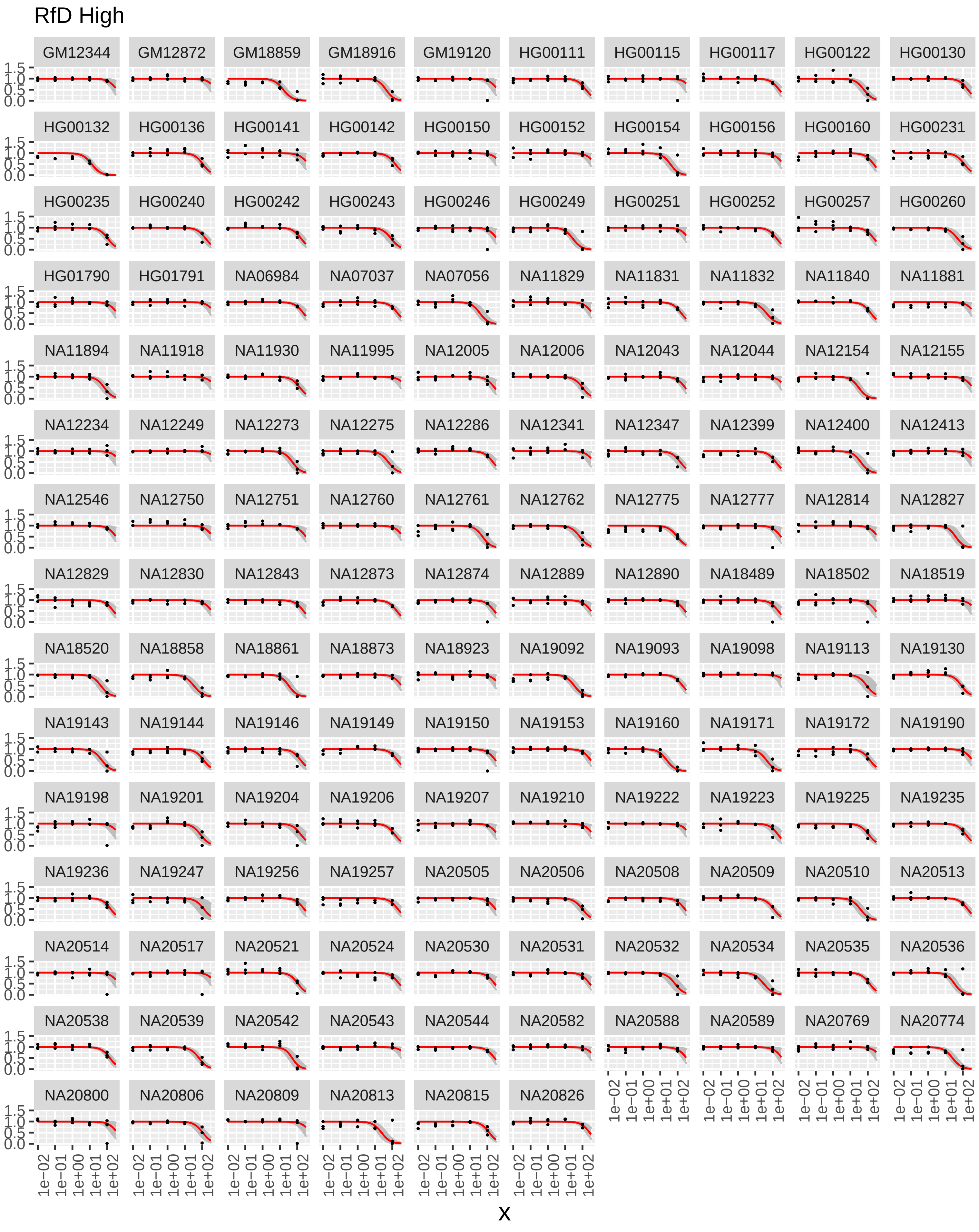
RfD High
GM12344
GM12872
GM18859
GM18916
GM19120
HG00111
HG00115
HG00117
HG00122
HG00130
HG00132
HG00136
HG00141
HG00142
HG00150
HG00152
HG00154
HG00156
HG00160
HG00231
HG00235
HG00240
HG00242
HG00243
HG00246
HG00249
HG00251
HG00252
HG00257
HG00260
HG01790
HG01791
NA06984
NA07037
NA07056
NA11829
NA11831
NA11832
NA11840
NA11881
NA11894
NA11918
NA11930
NA11995
NA12005
NA12006
NA12043
NA12044
NA12154
NA12155
NA12234
NA12249
NA12273
NA12275
NA12286
NA12341
NA12347
NA12399
NA12400
NA12413
NA12546
NA12750
NA12751
NA12760
NA12761
NA12762
NA12775
NA12777
NA12814
NA12827
NA12829
NA12830
NA12843
NA12873
NA12874
NA12889
NA12890
NA18489
NA18502
NA18519
NA18520
NA18858
NA18861
NA18873
NA18923
NA19092
NA19093
NA19098
NA19113
NA19130
NA19143
NA19144
NA19146
NA19149
NA19150
NA19153
NA19160
NA19171
NA19172
NA19190
NA19198
NA19201
NA19204
NA19206
NA19207
NA19210
NA19222
NA19223
NA19225
NA19235
NA19236
NA19247
NA19256
NA19257
NA20505
NA20506
NA20508
NA20509
NA20510
NA20513
NA20514
NA20517
NA20521
NA20524
NA20530
NA20531
NA20532
NA20534
NA20535
NA20536
NA20538
NA20539
NA20542
NA20543
NA20544
NA20582
NA20588
NA20589
NA20769
NA20774
NA20800
NA20806
NA20809
NA20813
NA20815
NA20826
1.5
1.0
0.5
0.0
1e−02
1e−01
1e+00
1e+01
1e+02
x
