# Supplementary material for: A Population-Based Human In Vitro Approach to Quantify Inter-Individual Variability in Responses to Chemical Mixtures
Source: Toxics. 2022 Aug 1;10(8):441. doi: 10.3390/toxics10080441 (PMC9413237; doi:10.3390/toxics10080441)
Supplement: Supplementary file 1 [file toxics-10-00441-s001.zip › Figure S5 Chemical and mixture-specific Manhattan plot visualizations.pdf]

2,4,5-TRICHLOROPHENOL

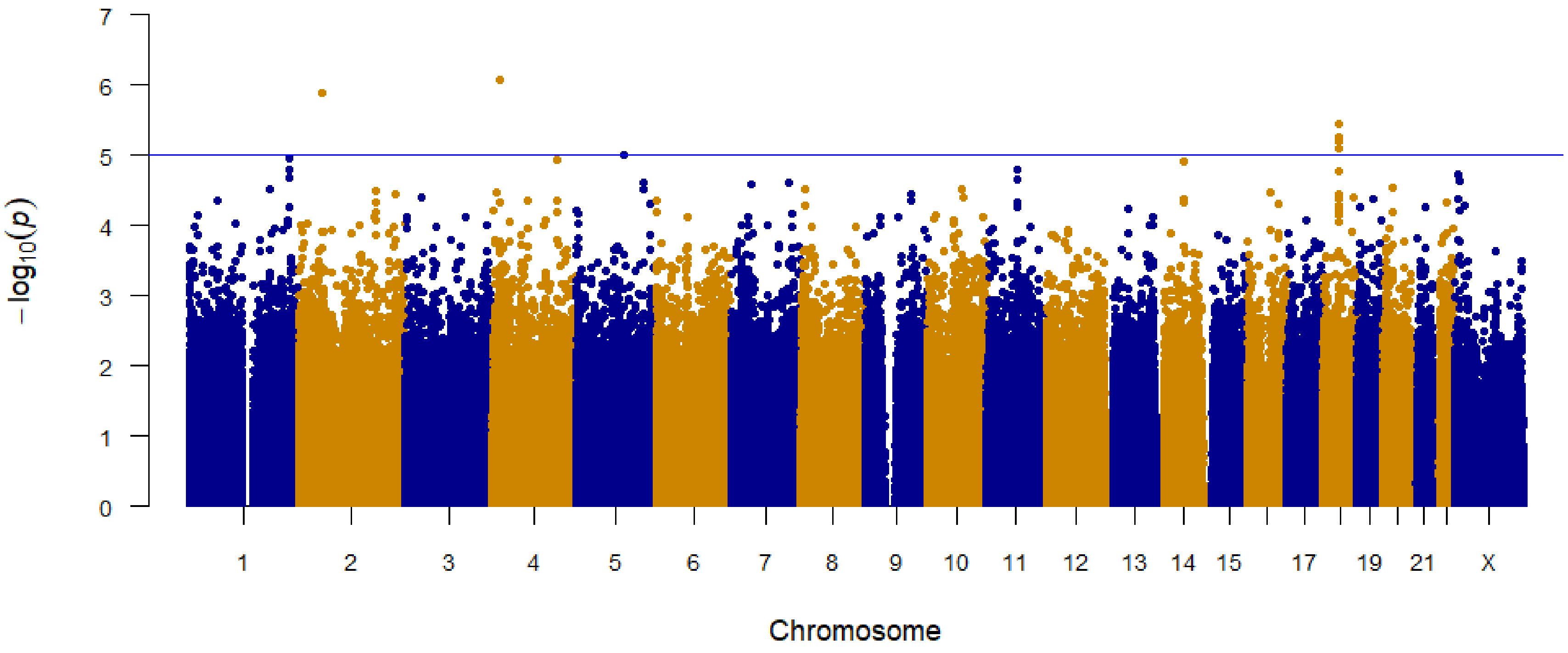

2,4,5-TRICHLOROPHENOL-2

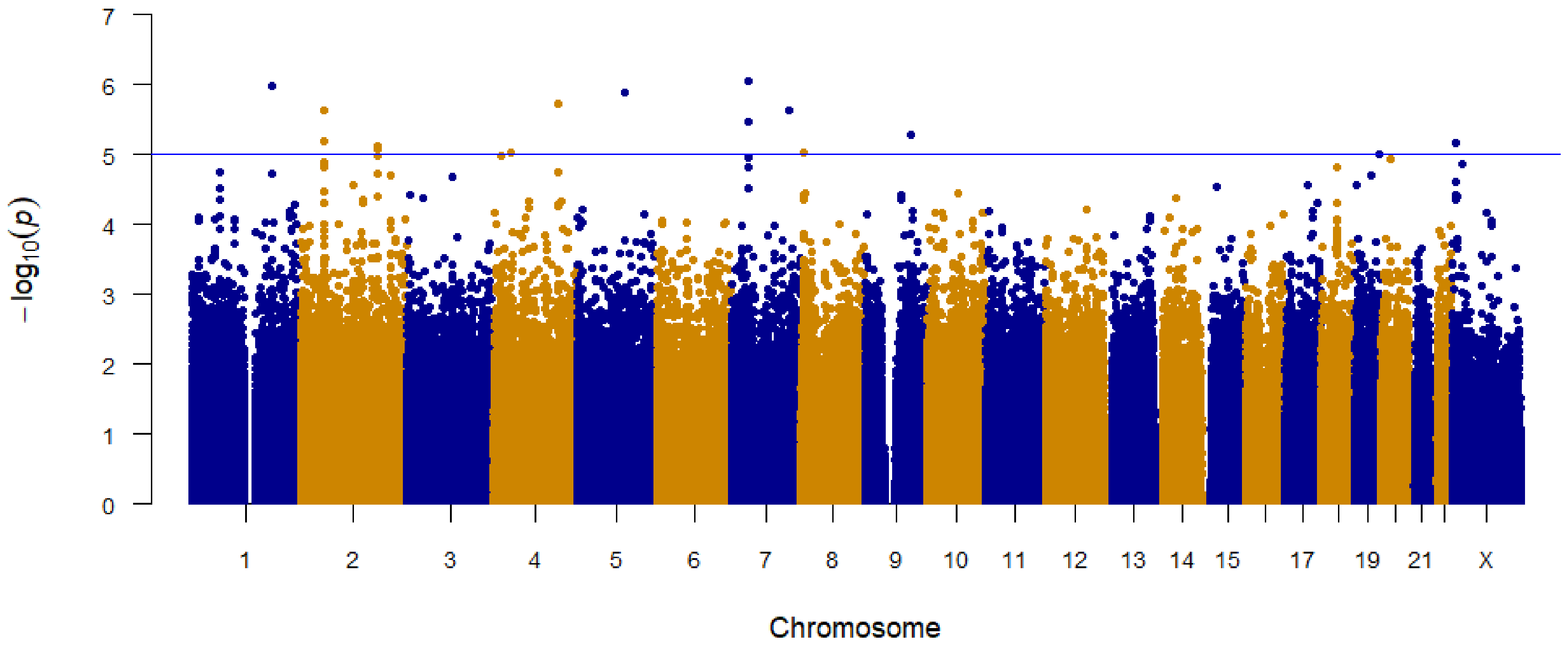

4,6-DINITRO-O-CRESOL

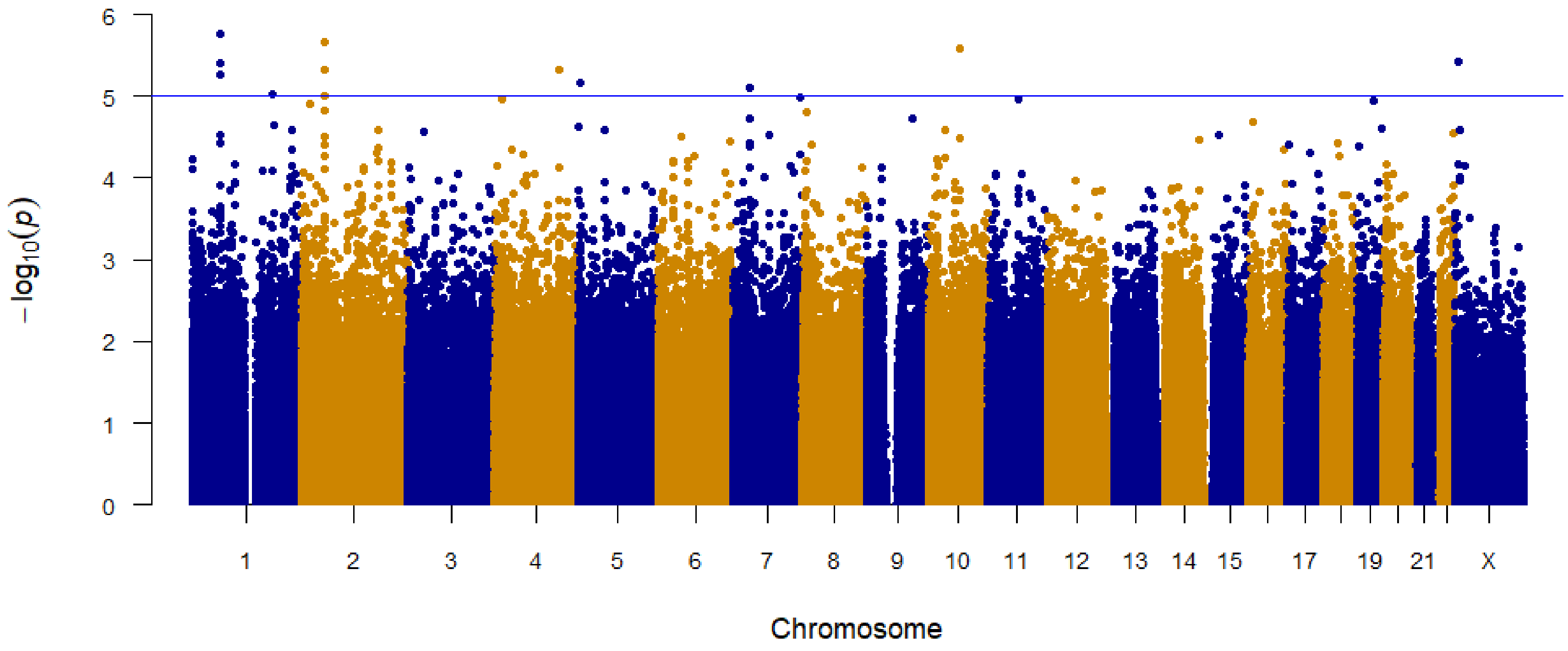

ALDRIN

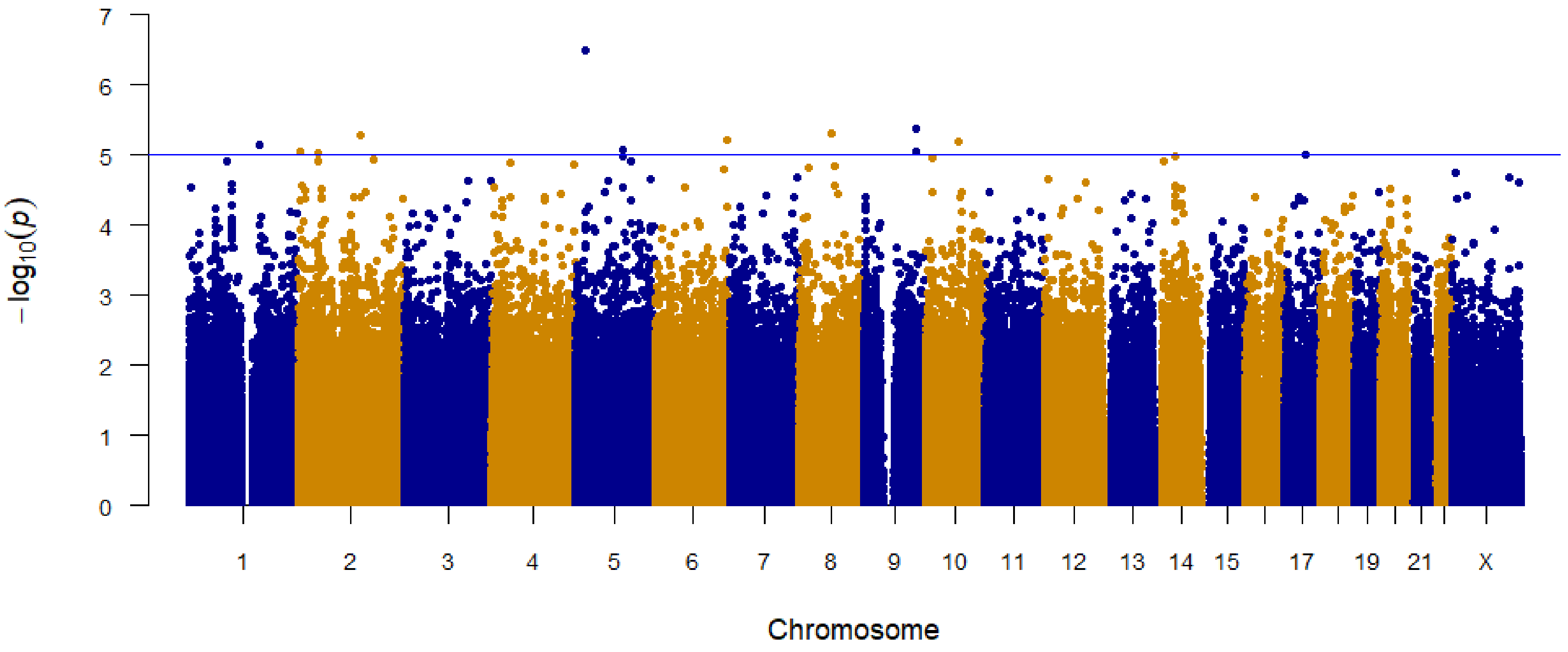

7
6
5
4
3
2
1
0
−log10(p)
1
2
3
4
5
6
7
8
9
10
11
12
13
14
15
17
19
21
X
Chromosome

AZINPHOS-METHYL

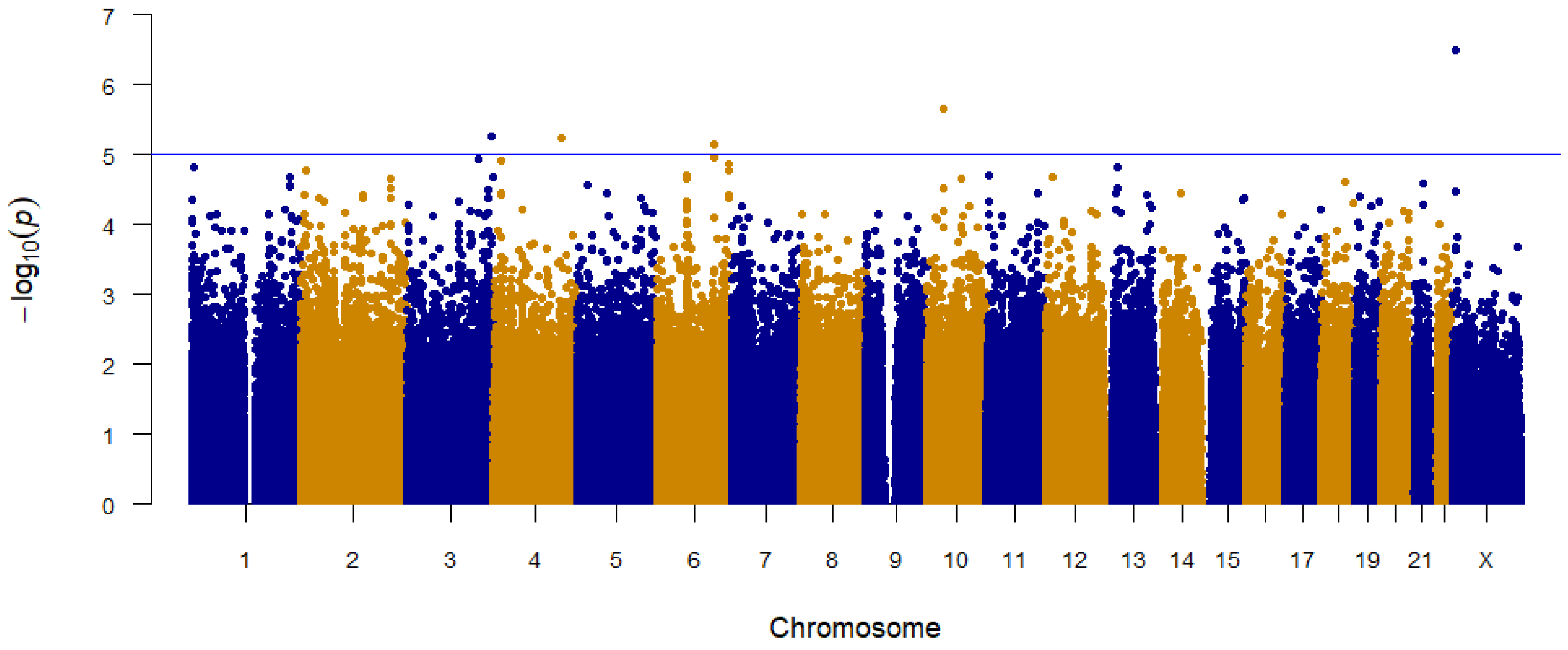

CADMIUM(Chloride)

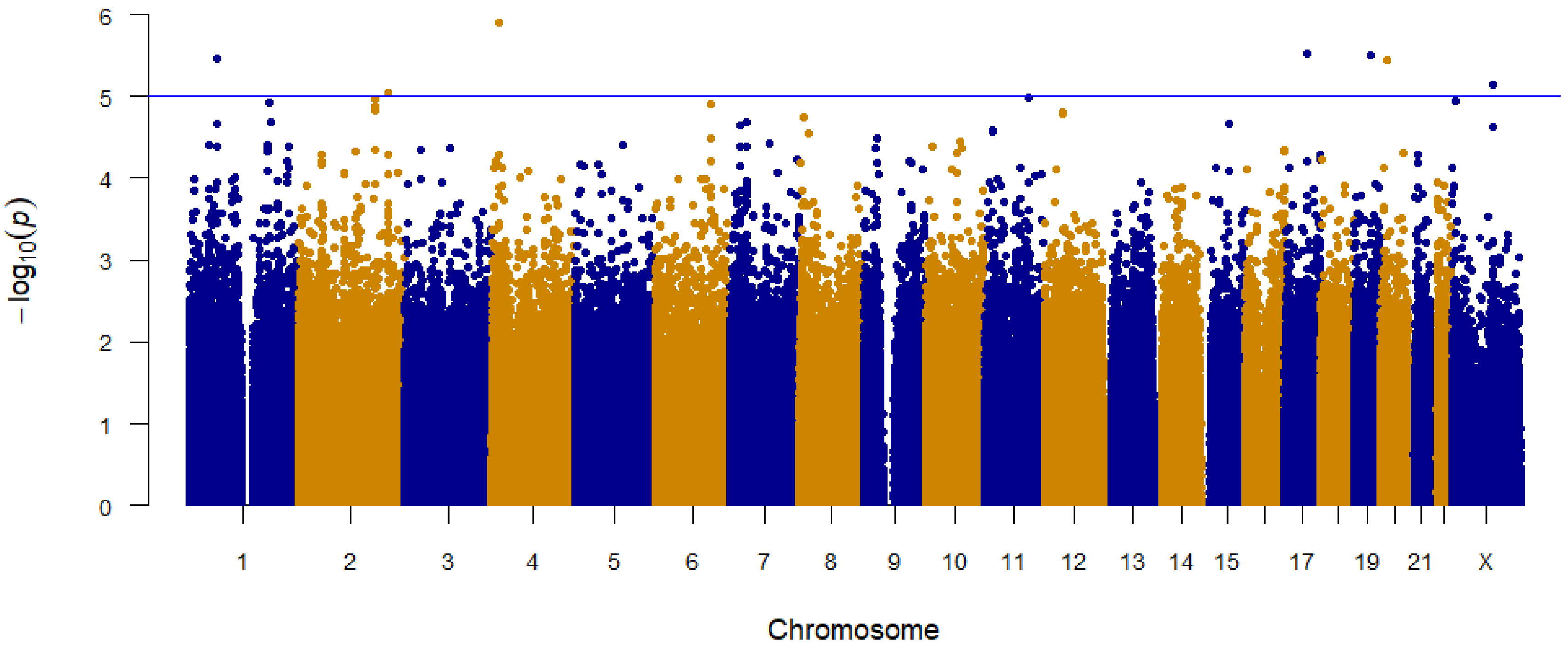

CHLORPYRIFOS

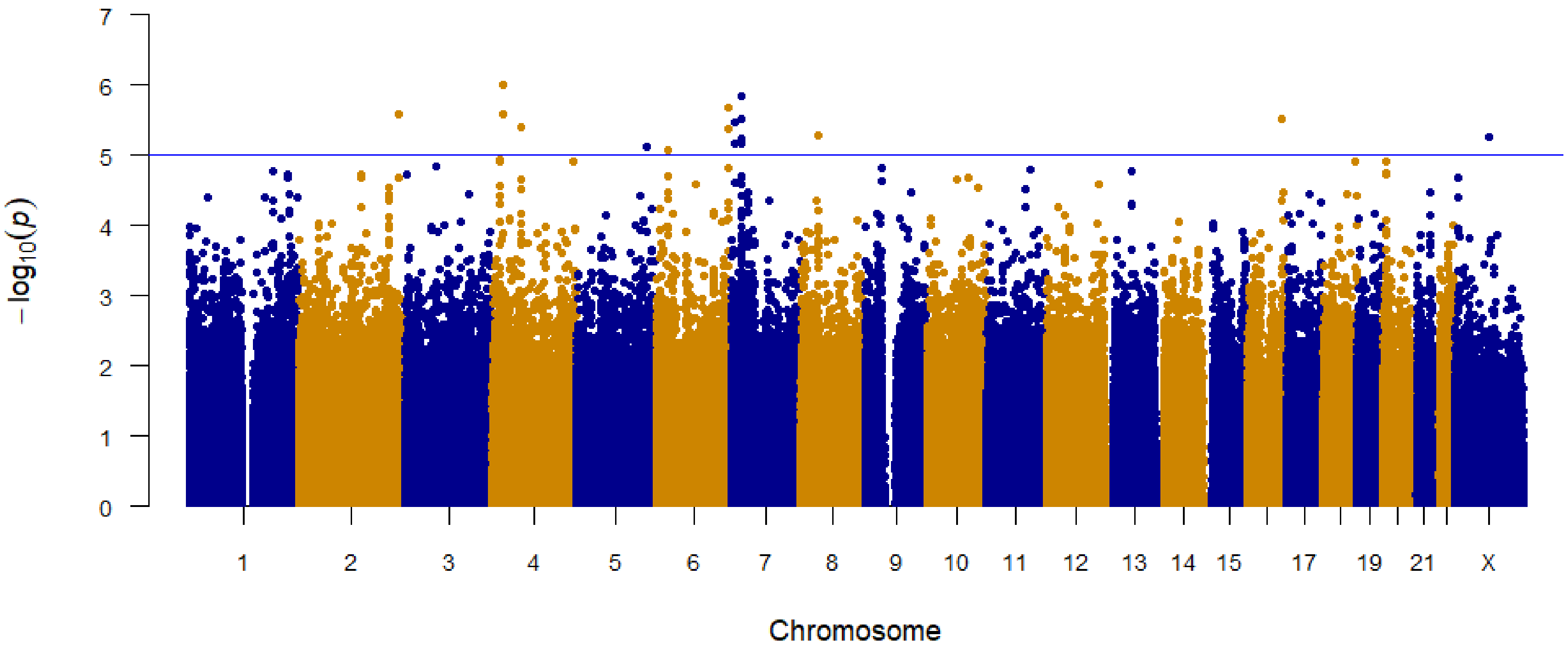

COBALT

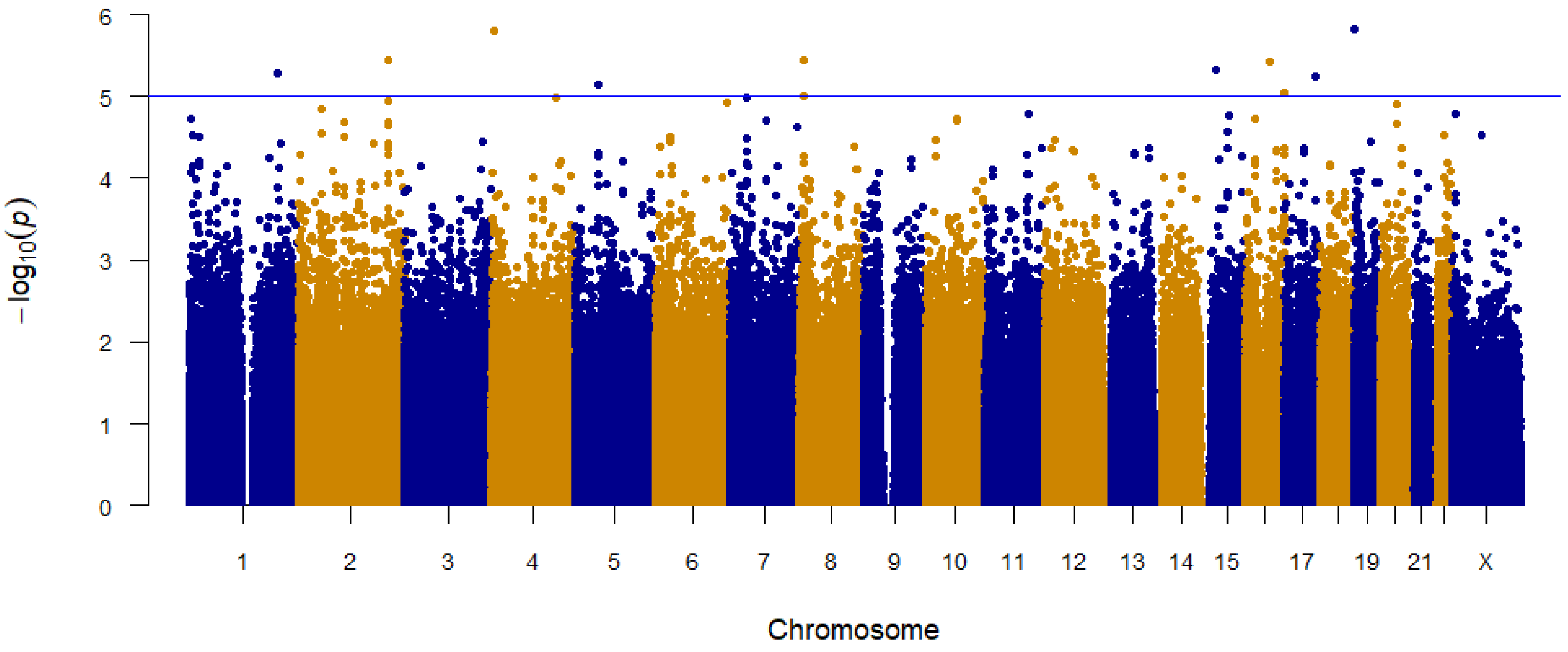

6
5
4
3
2
1
0
$-\log_{10}(p)$
1
2
3
4
5
6
7
8
9
10
11
12
13
14
15
17
19
21
X
Chromosome

DDD, P,P'-

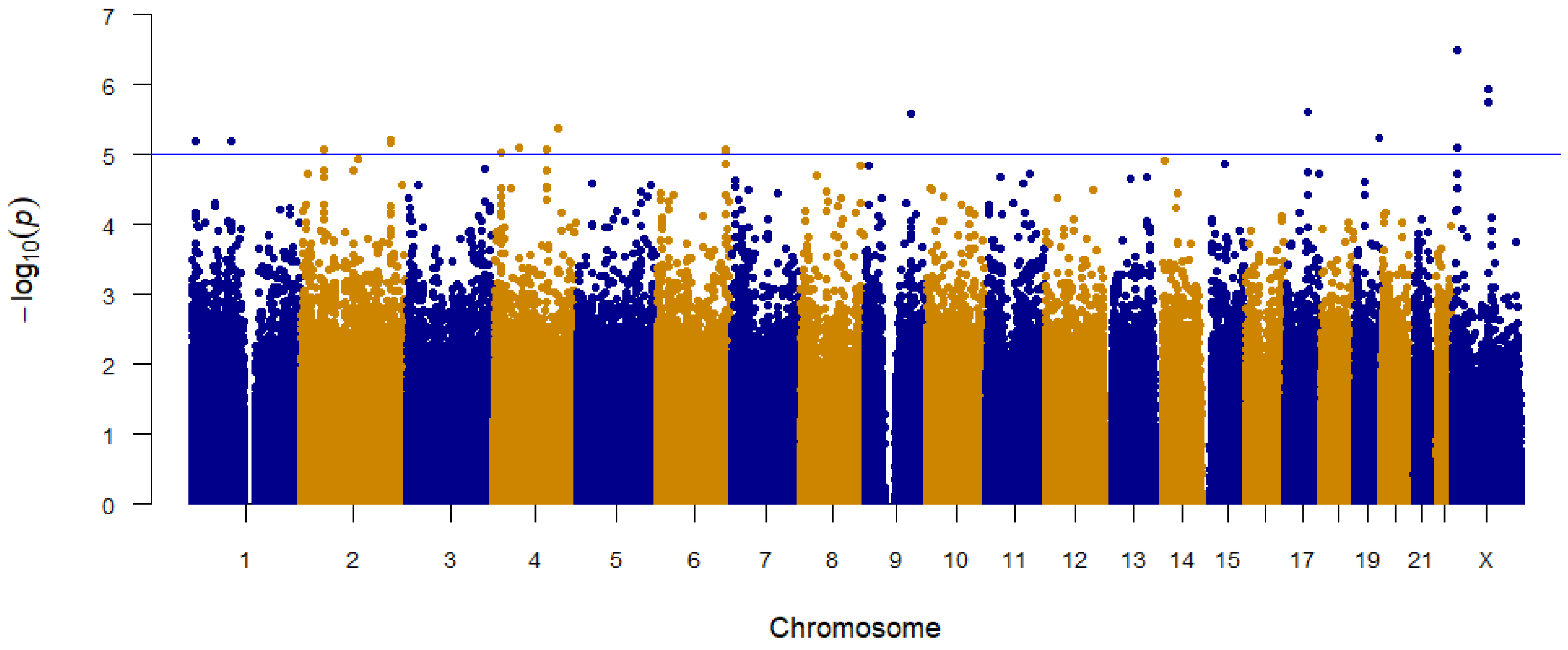
7
6
5
4
3
2
1
0
−log10(p)
1
2
3
4
5
6
7
8
9
10
11
12
13
14
15
17
19
21
X
Chromosome

DDT, O,P'-

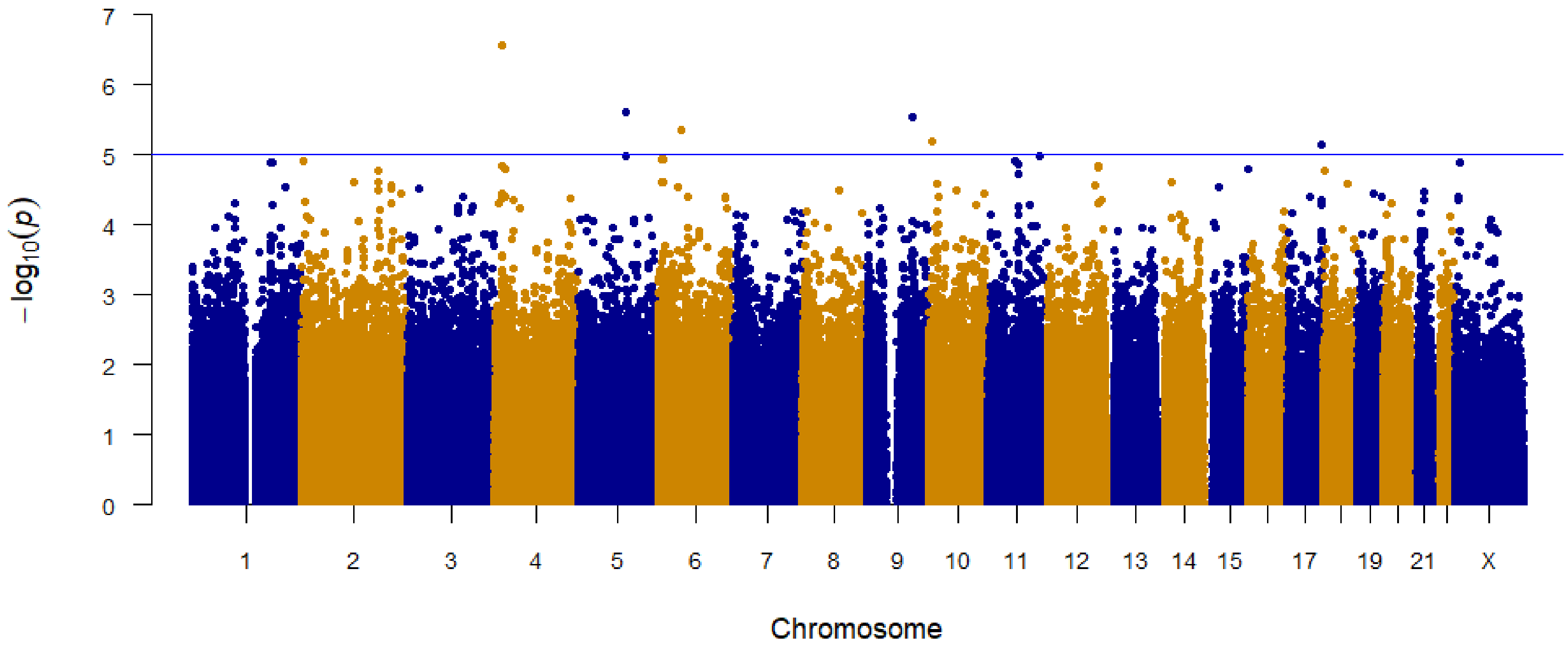

7
6
5
4
3
2
1
0
$-\log_{10}(p)$
1
2
3
4
5
6
7
8
9
10
11
12
13
14
15
17
19
21
X
Chromosome

DDT, P,P'-

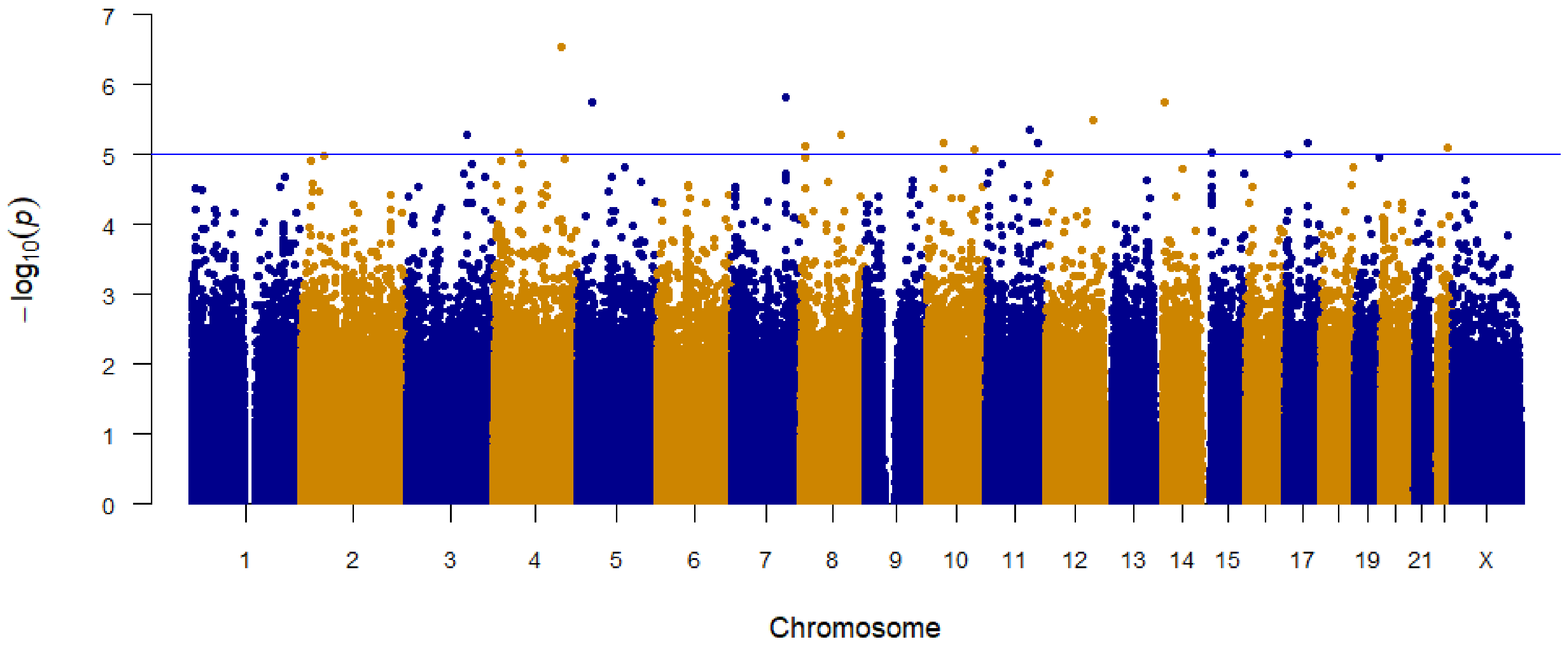

7
6
5
4
3
2
1
0
$-\log_{10}(p)$
1
2
3
4
5
6
7
8
9
10
11
12
13
14
15
17
19
21
X
Chromosome

DI-N-BUTYL PHTHALATE

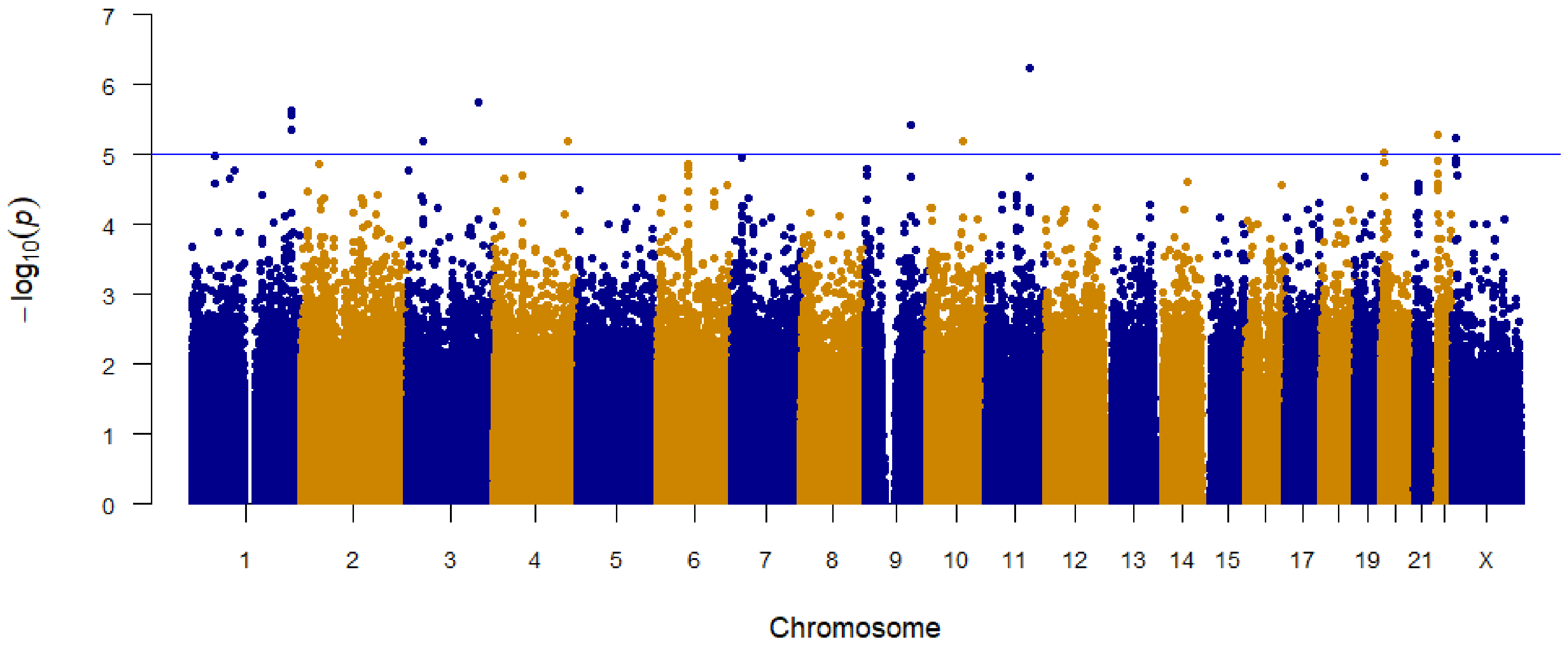

DIAZINON

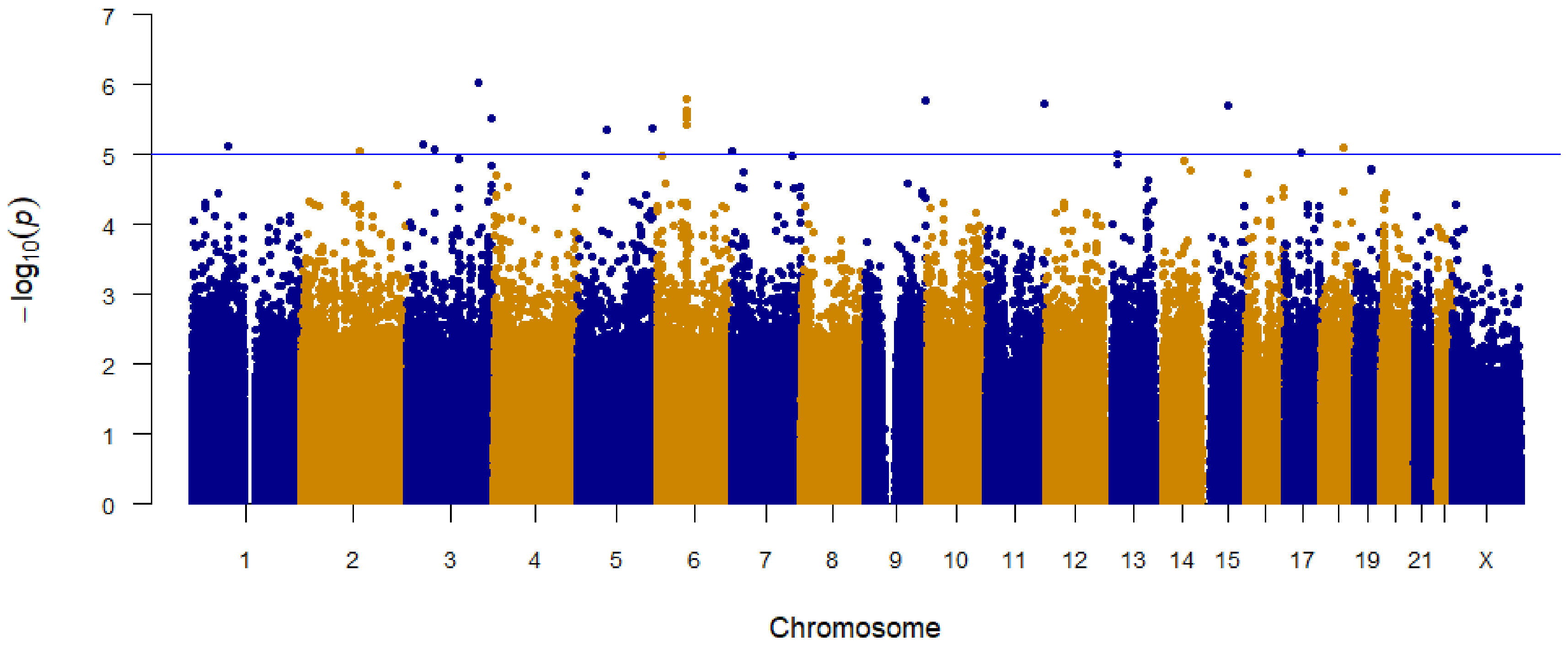

7
6
5
4
3
2
1
0
−log10(p)
1
2
3
4
5
6
7
8
9
10
11
12
13
14
15
17
19
21
X
Chromosome

DICOFOL

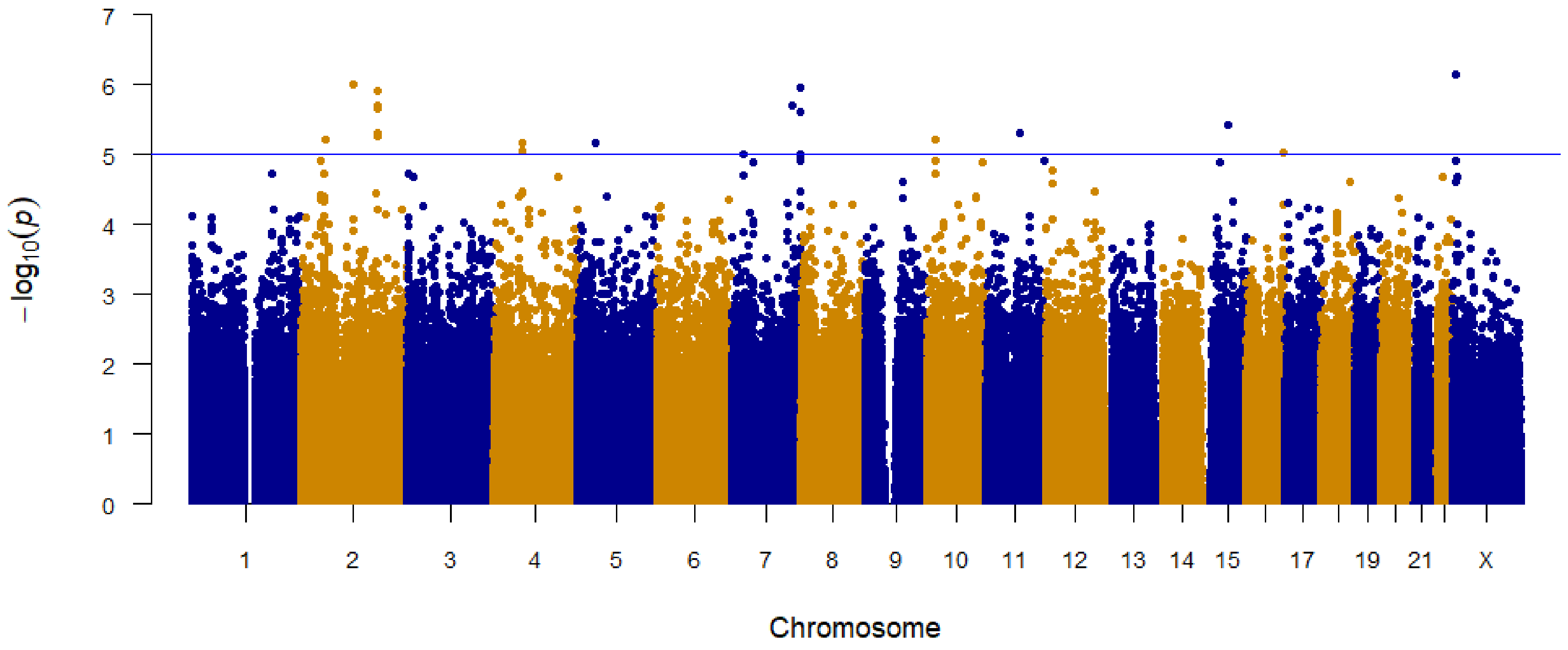

7
6
5
4
3
2
1
0
$-\log_{10}(p)$
1
2
3
4
5
6
7
8
9
10
11
12
13
14
15
17
19
21
X
Chromosome

DIELDRIN

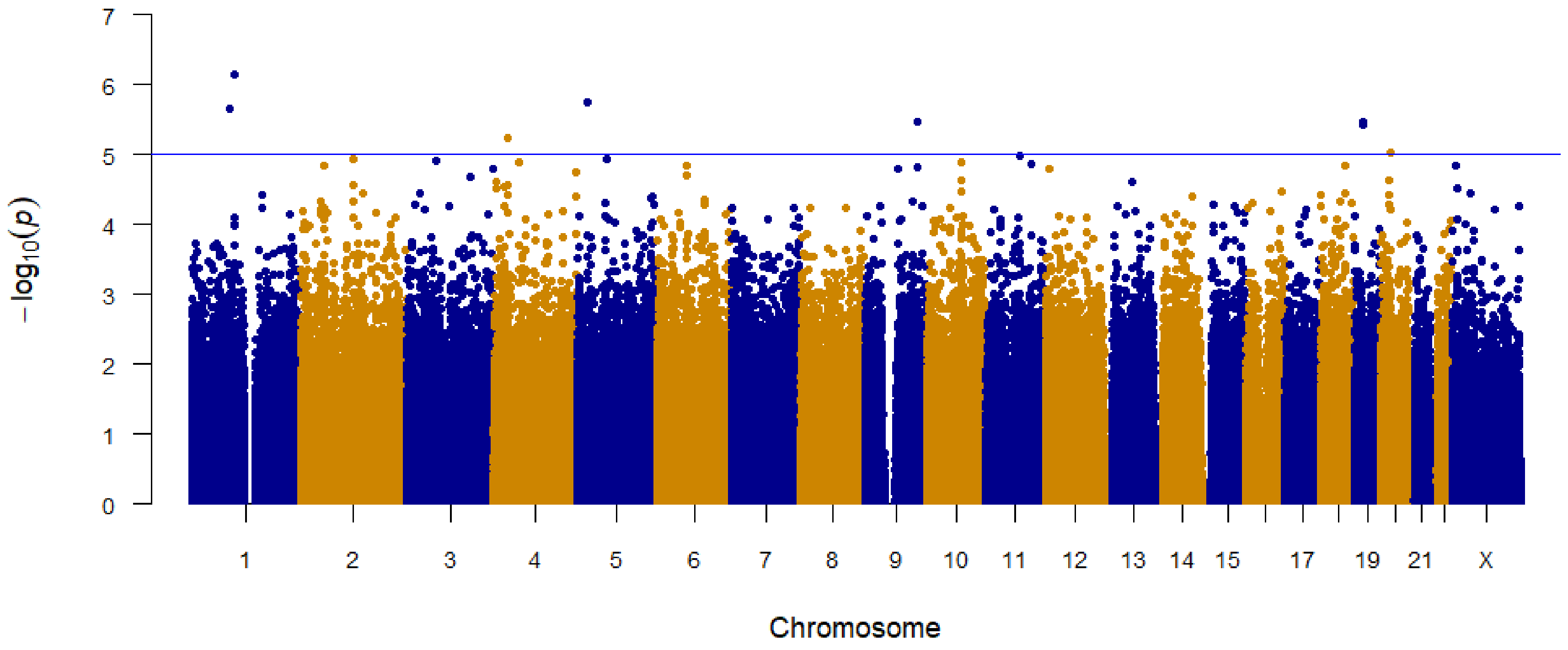
7
6
5
4
3
2
1
0
$-\log_{10}(p)$
1
2
3
4
5
6
7
8
9
10
11
12
13
14
15
17
19
21
X
Chromosome

DISULFOTON

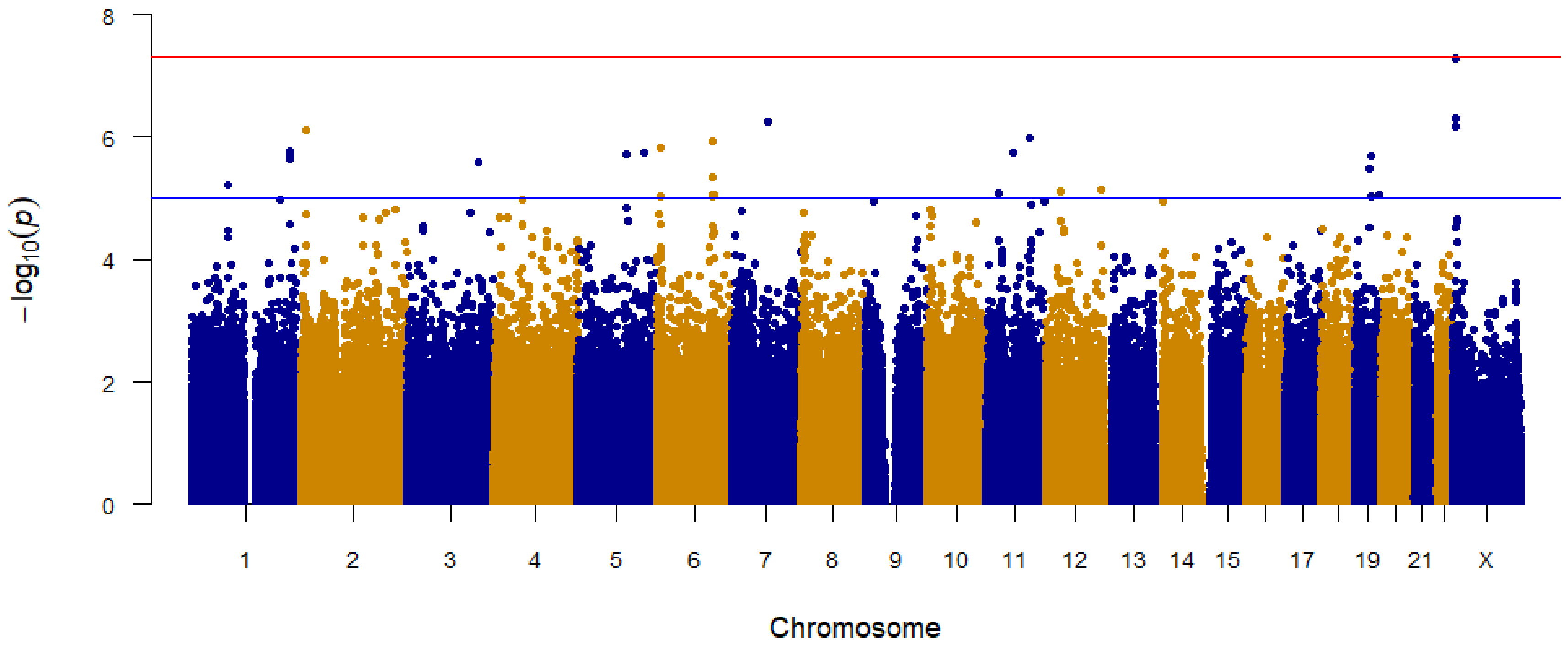

ENDOSULFAN

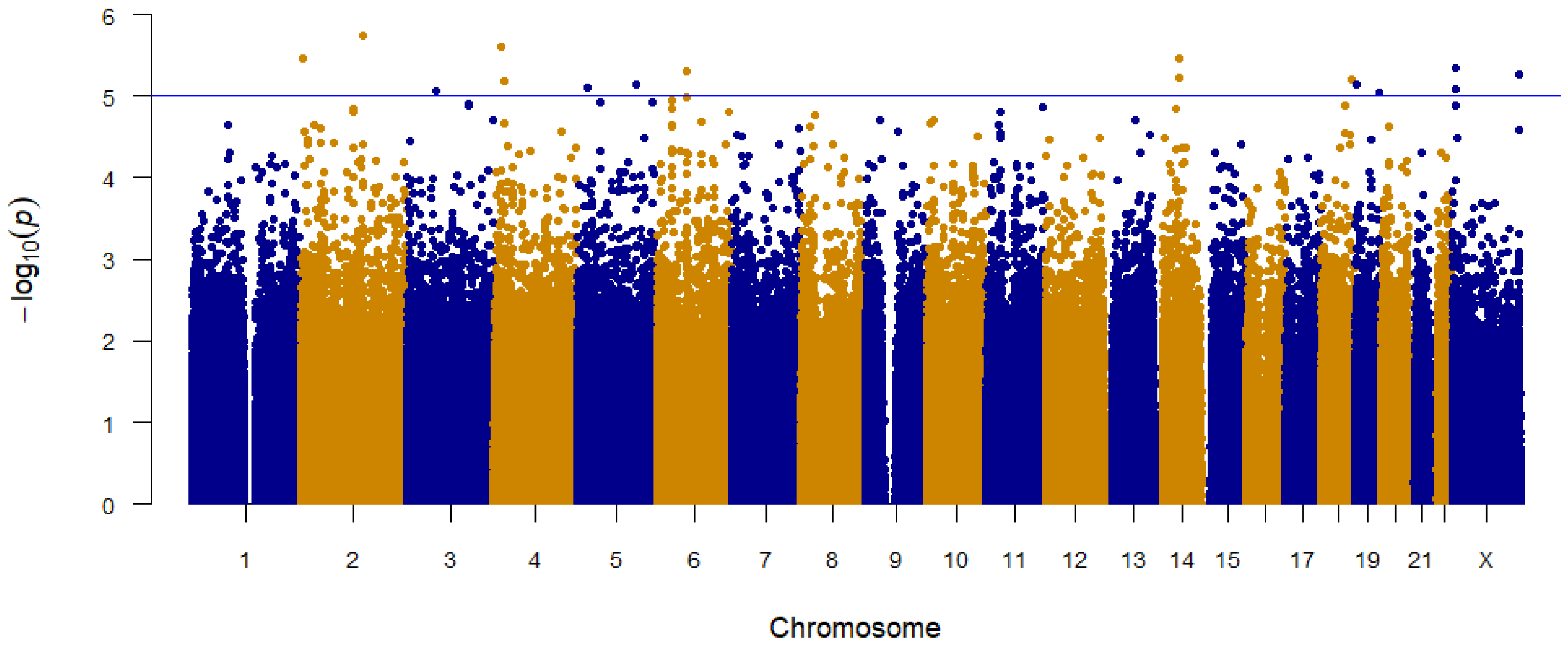
6
5
4
3
2
1
0
$-\log_{10}(p)$
1
2
3
4
5
6
7
8
9
10
11
12
13
14
15
17
19
21
X
Chromosome

ENDRIN

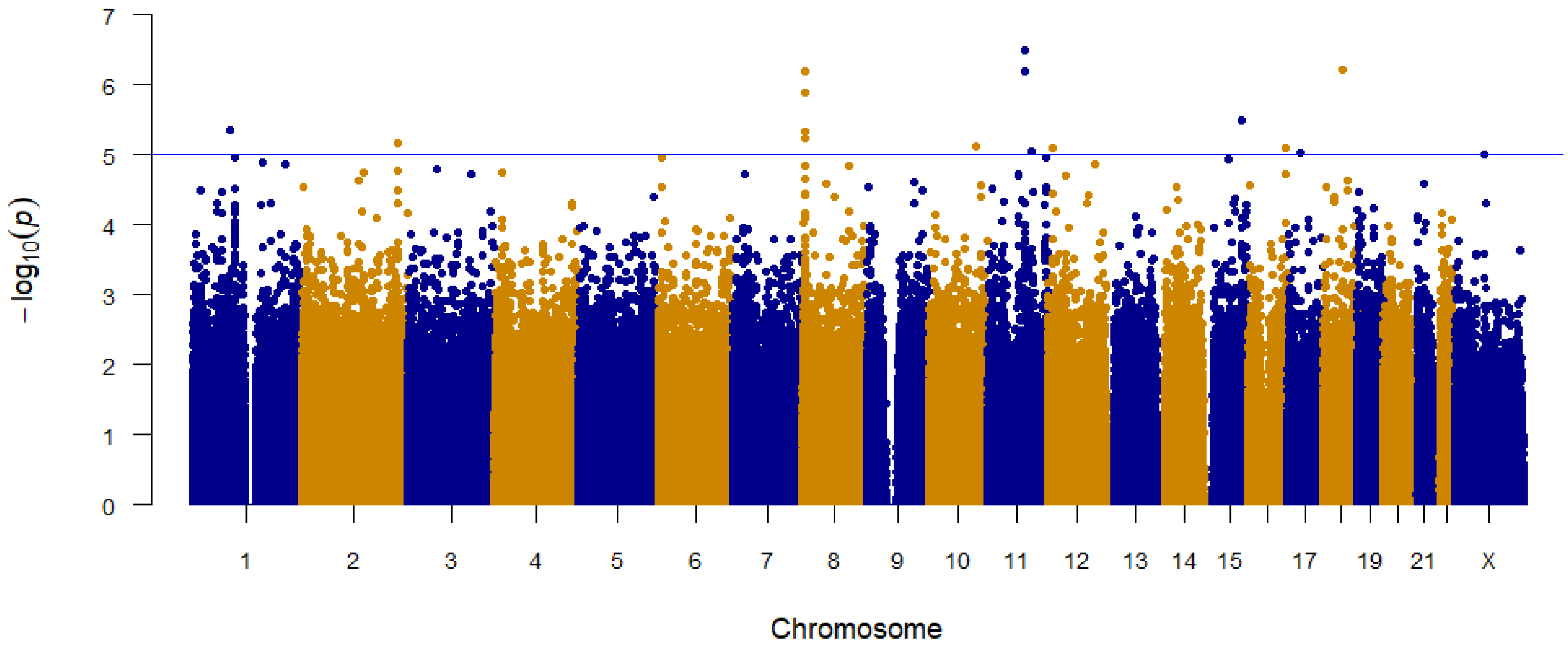

7
6
5
4
3
2
1
0
−log10(p)
1
2
3
4
5
6
7
8
9
10
11
12
13
14
15
17
19
21
X
Chromosome

ETHION

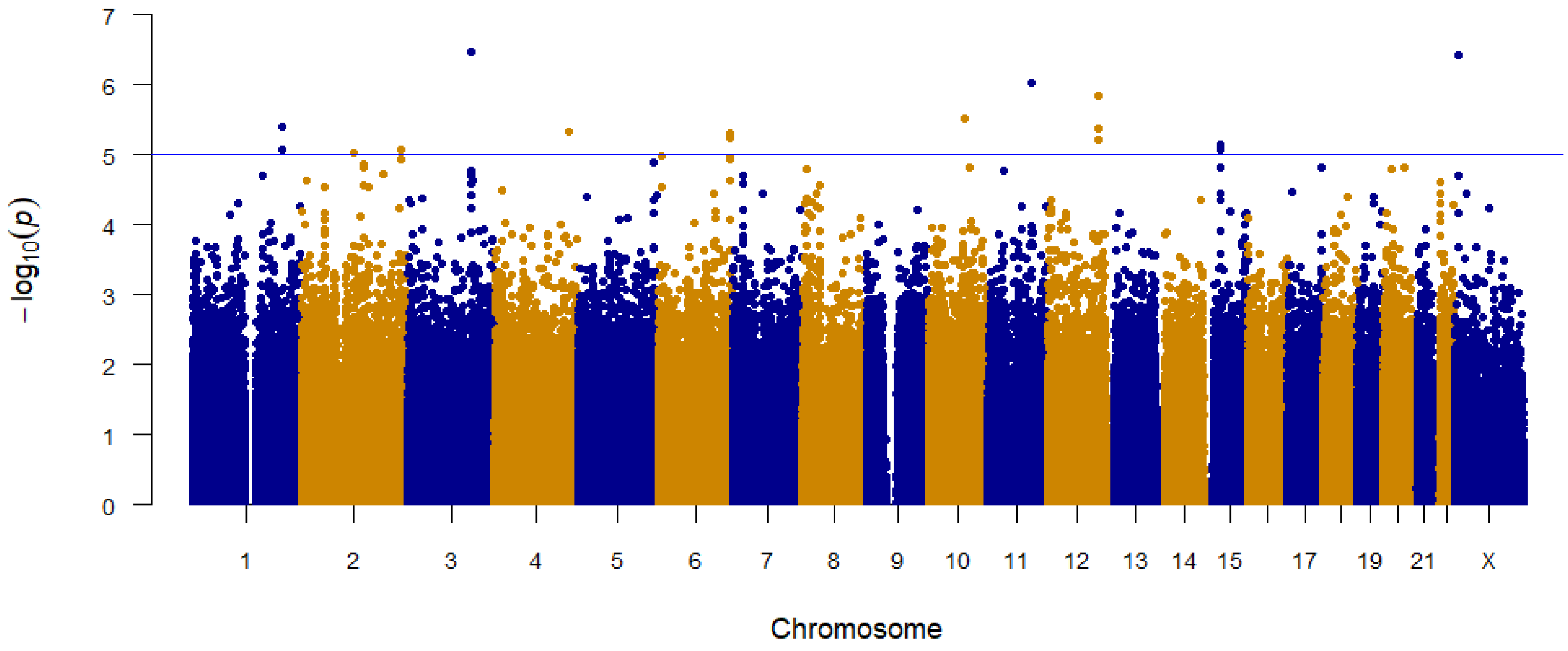

7
6
5
4
3
2
1
0
$-\log_{10}(p)$
1
2
3
4
5
6
7
8
9
10
11
12
13
14
15
17
19
21
X
Chromosome

HEPTACHLOR

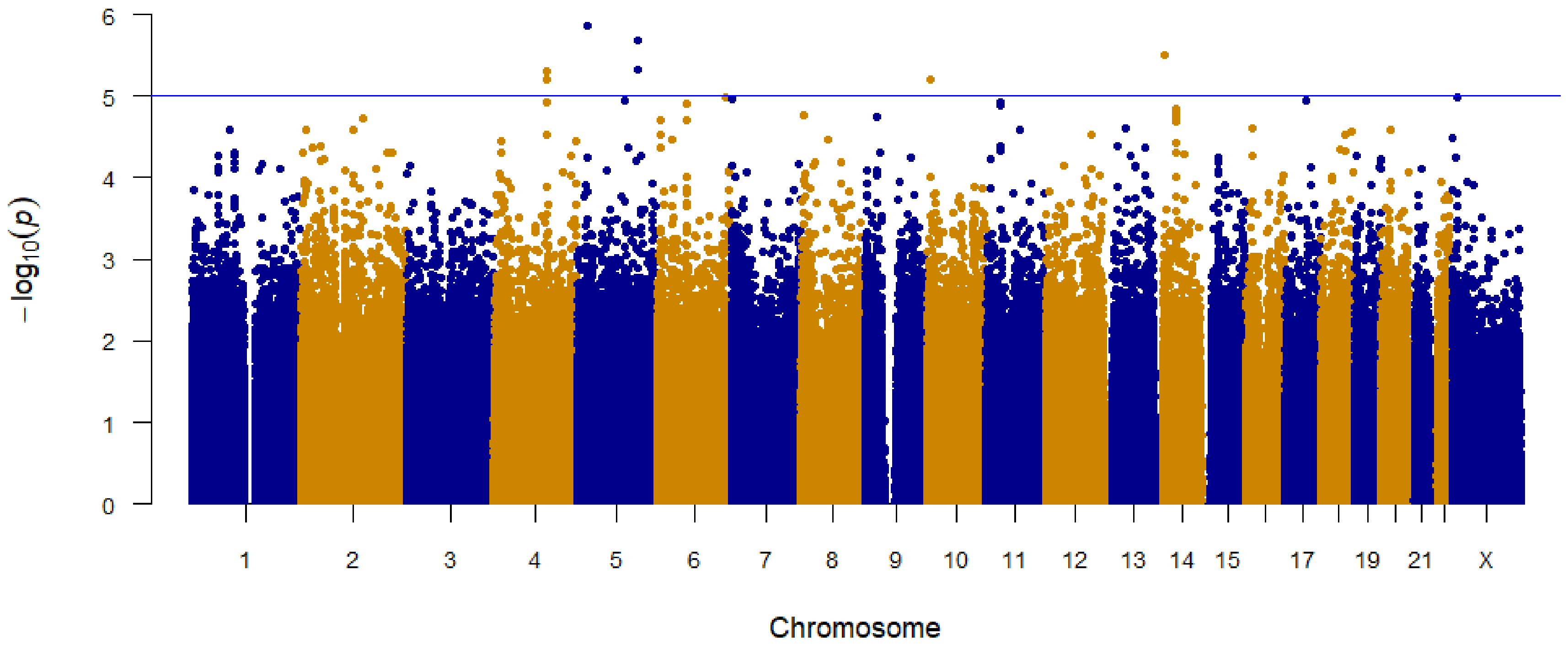

6
5
4
3
2
1
0
$-\log_{10}(p)$
1
2
3
4
5
6
7
8
9
10
11
12
13
14
15
17
19
21
X
Chromosome

HEPTACHLOR EPOXIDE

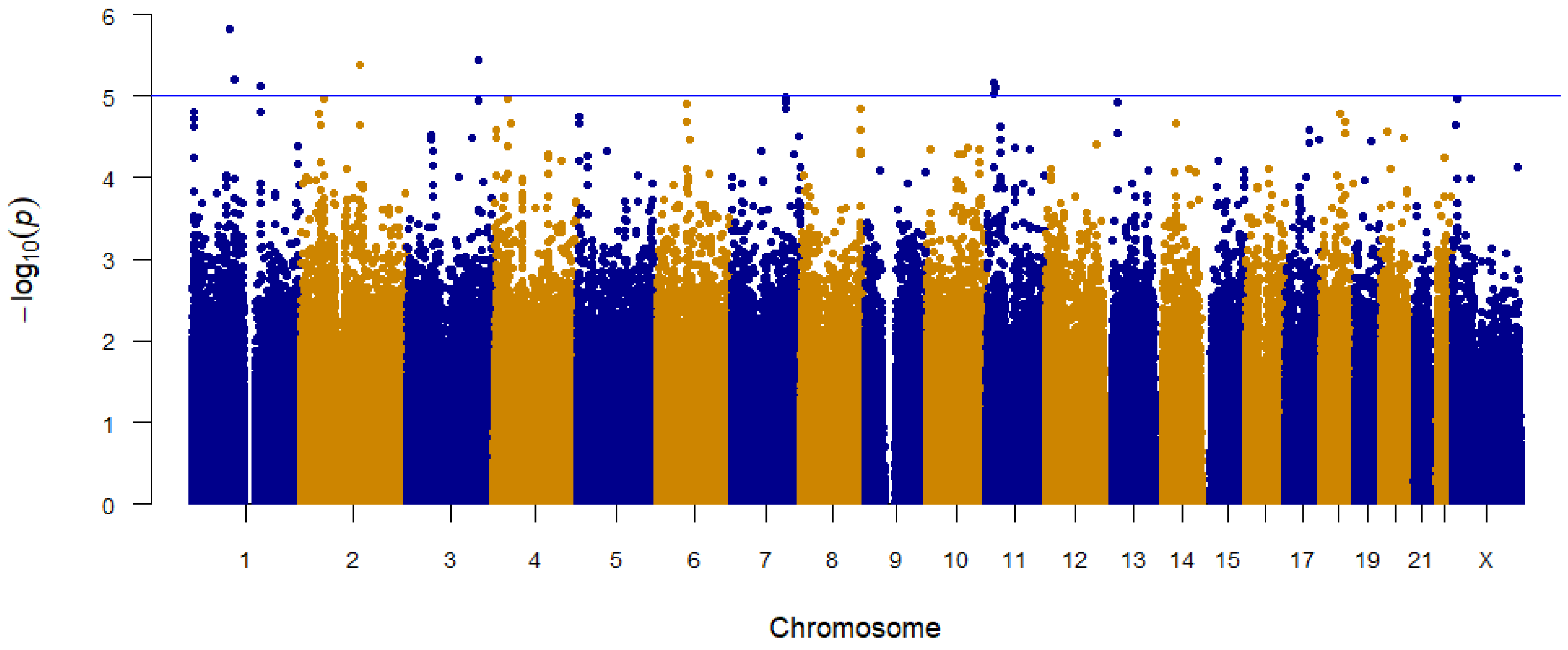

MERCURIC CHLORIDE

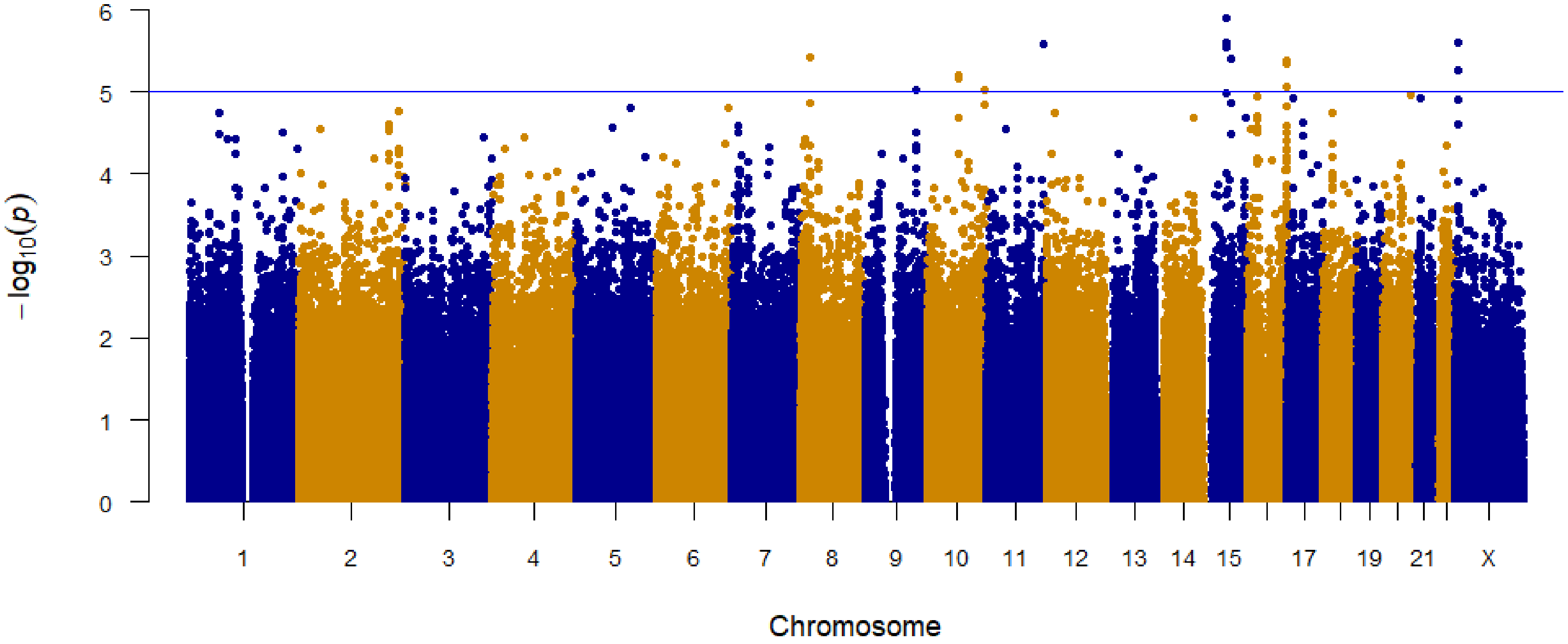

MERCURIC CHLORIDE-2

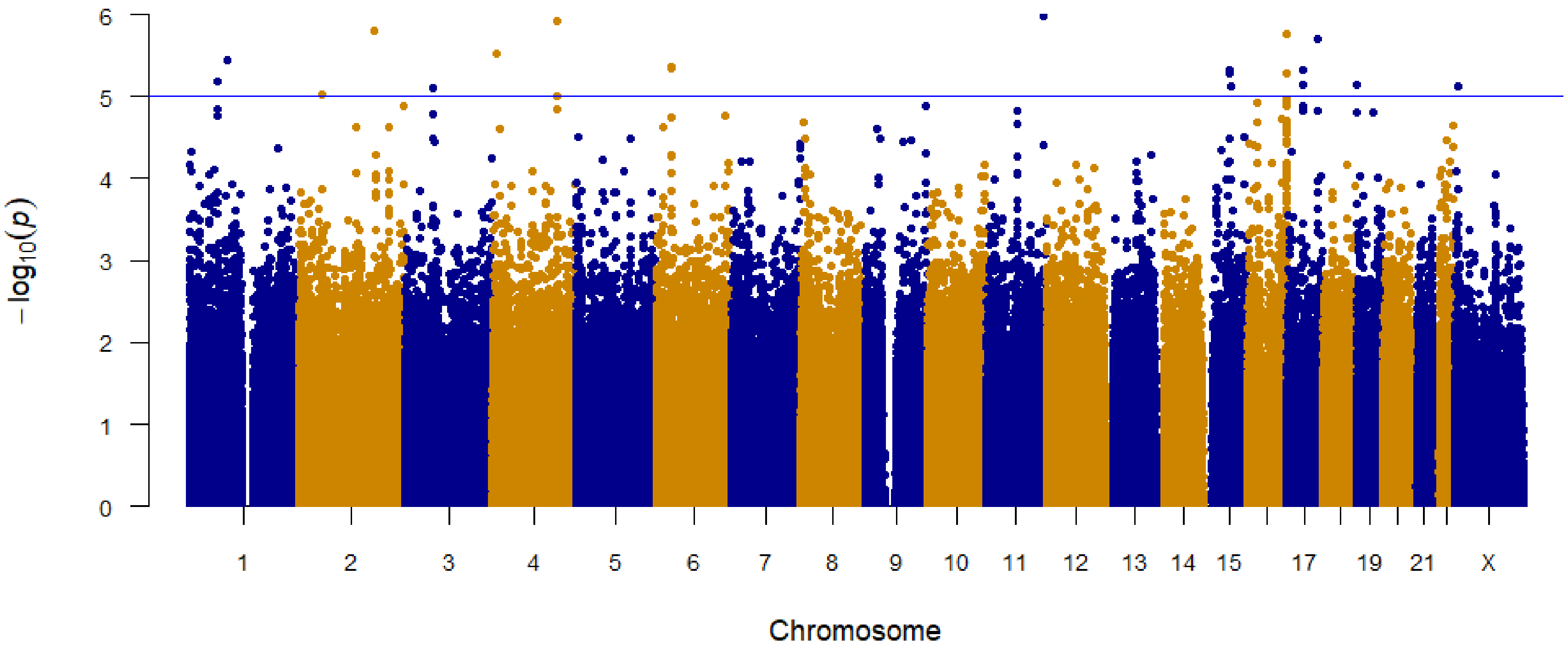

METHOXYCHLOR

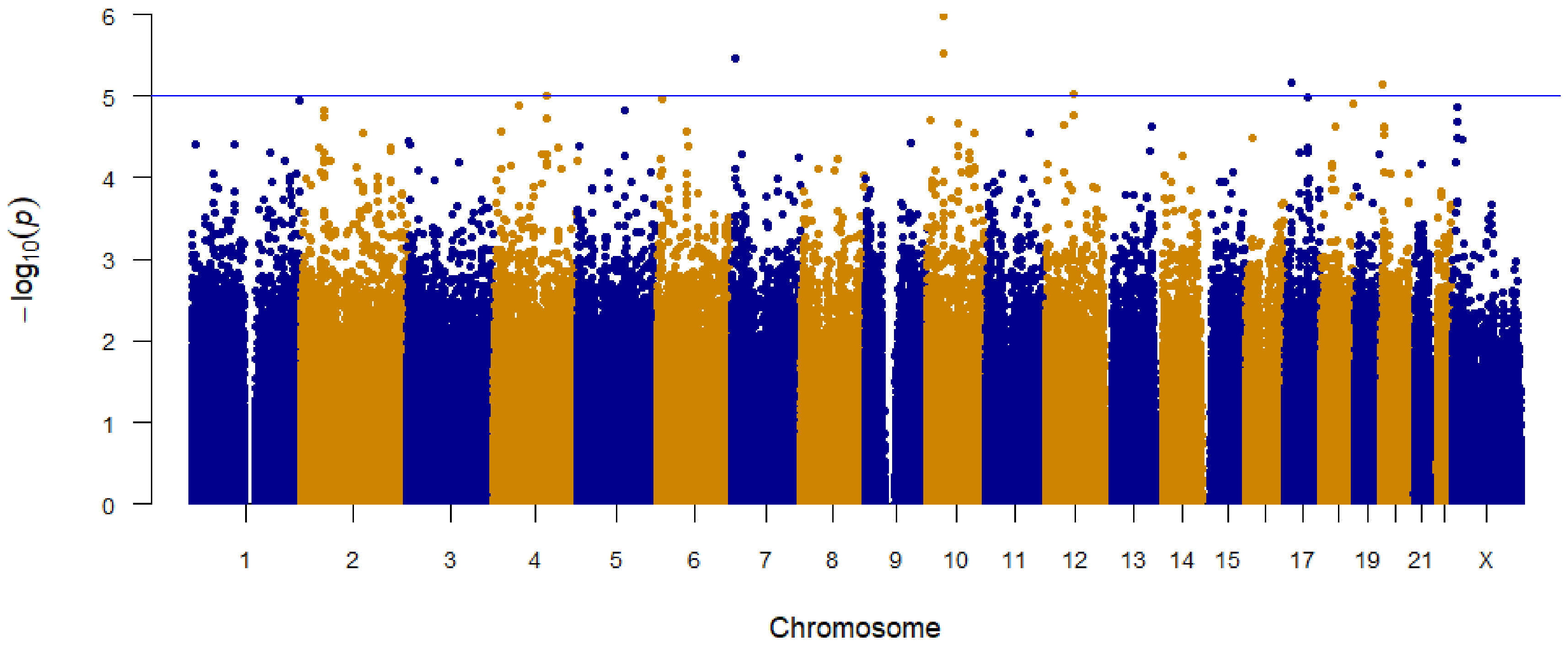

NICKEL

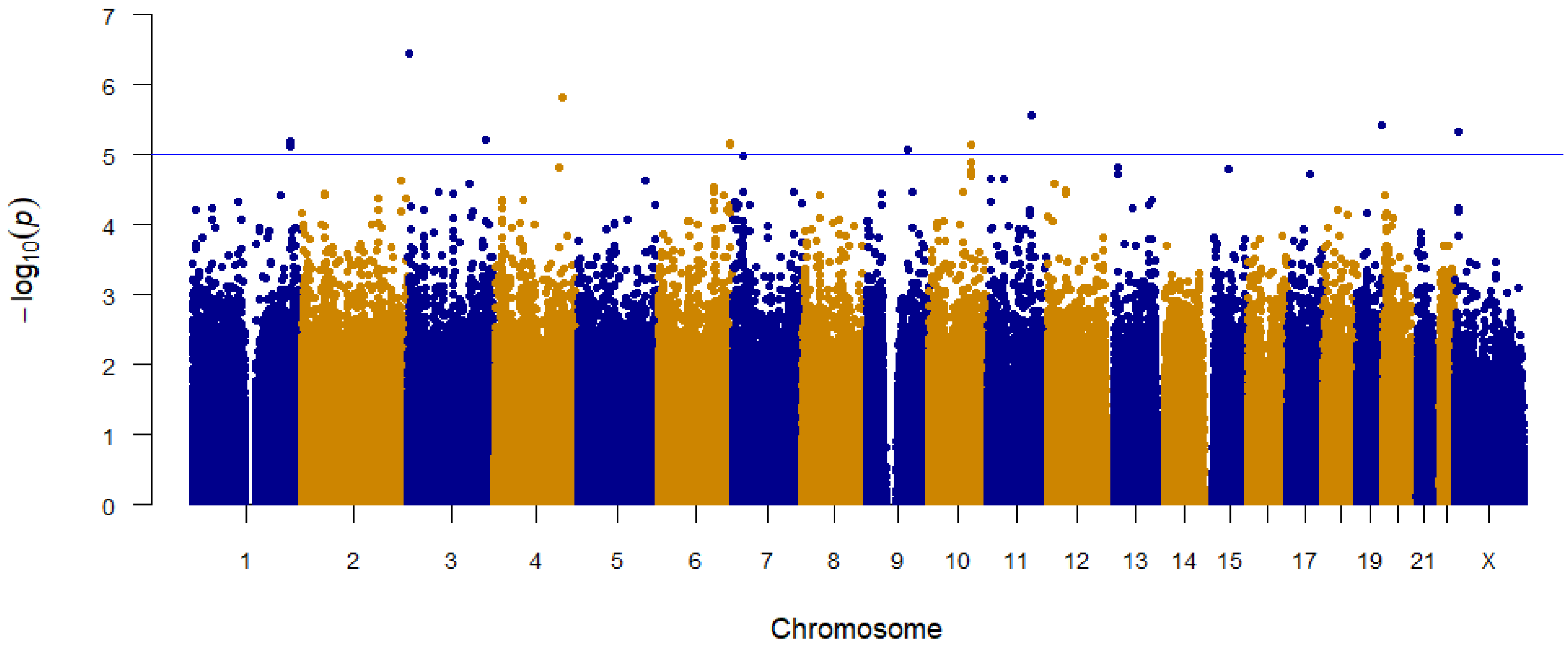

7
6
5
4
3
2
1
0
−log10(p)
1
2
3
4
5
6
7
8
9
10
11
12
13
14
15
17
19
21
X
Chromosome

PARATHION

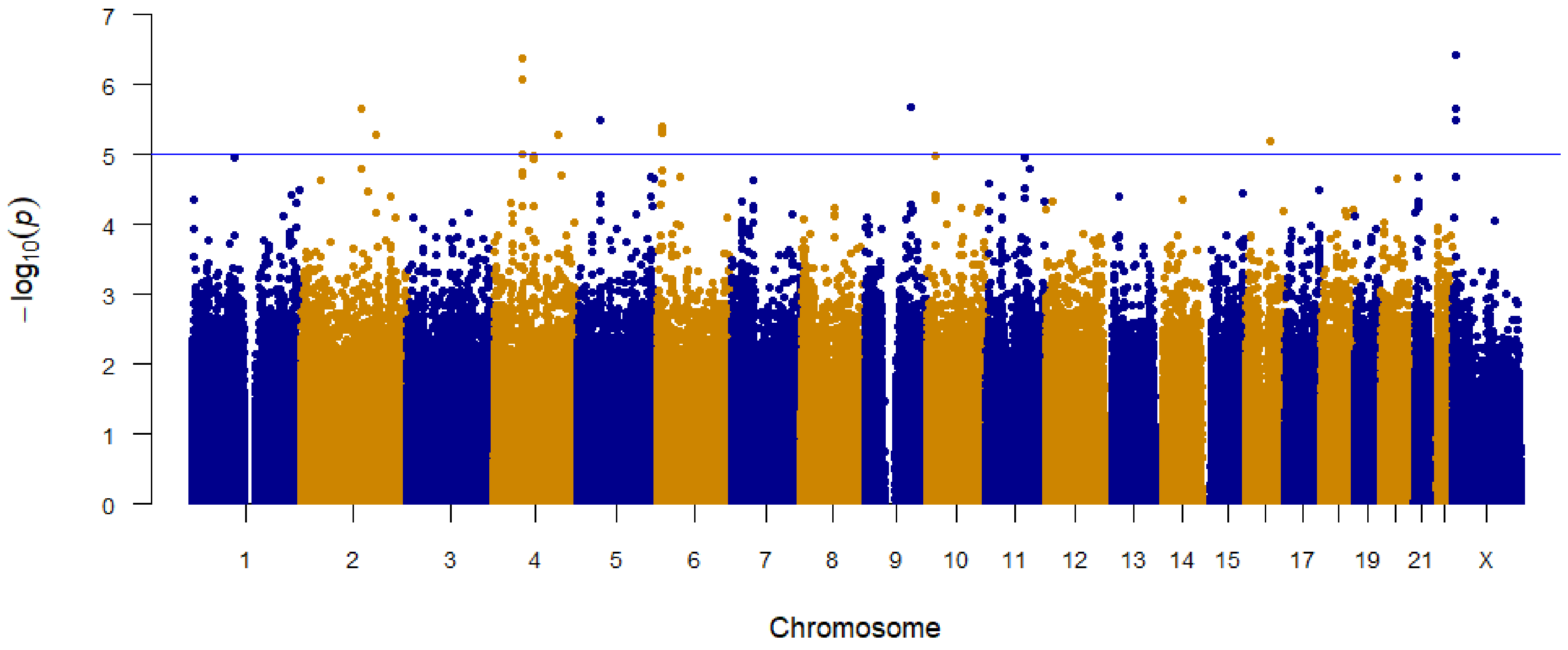
7
6
5
4
3
2
1
0
$-\log_{10}(p)$
1
2
3
4
5
6
7
8
9
10
11
12
13
14
15
17
19
21
X
Chromosome

PENTACHLOROPHENOL

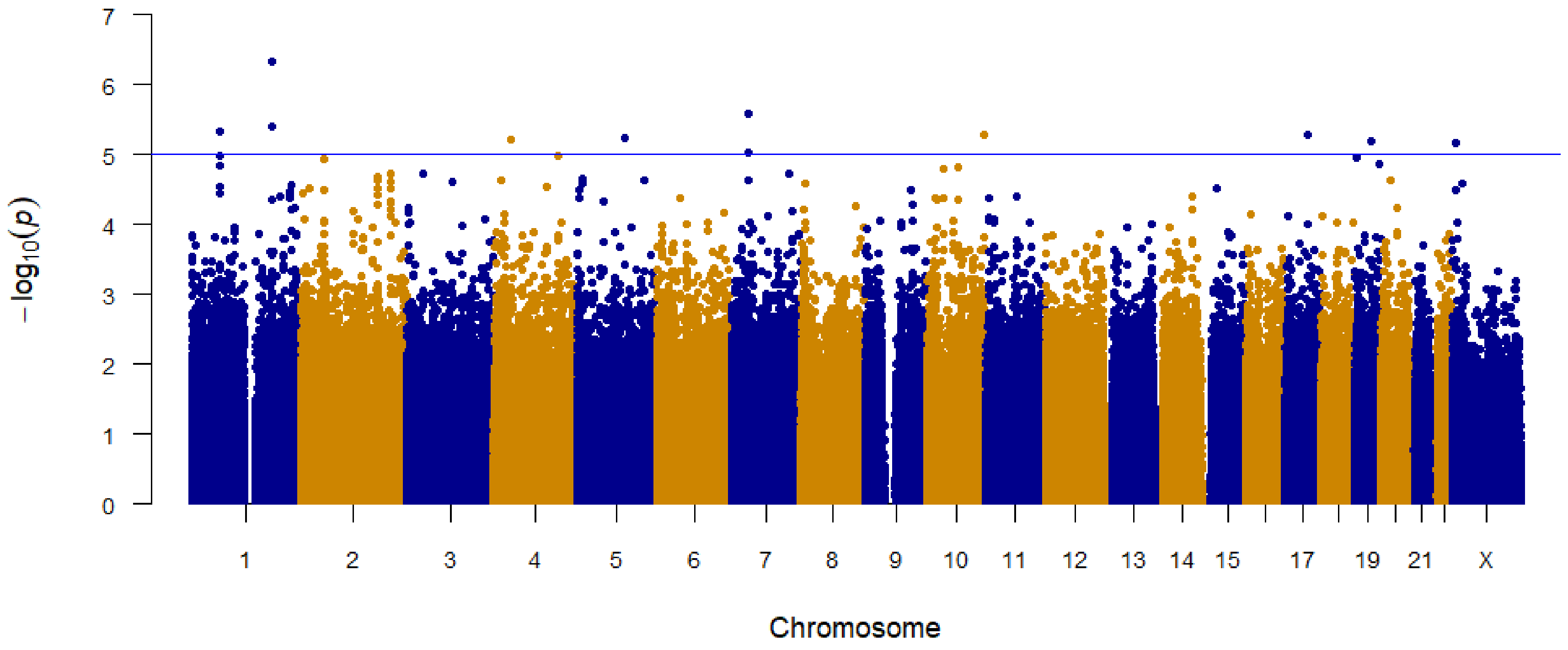

Potassium Chromate

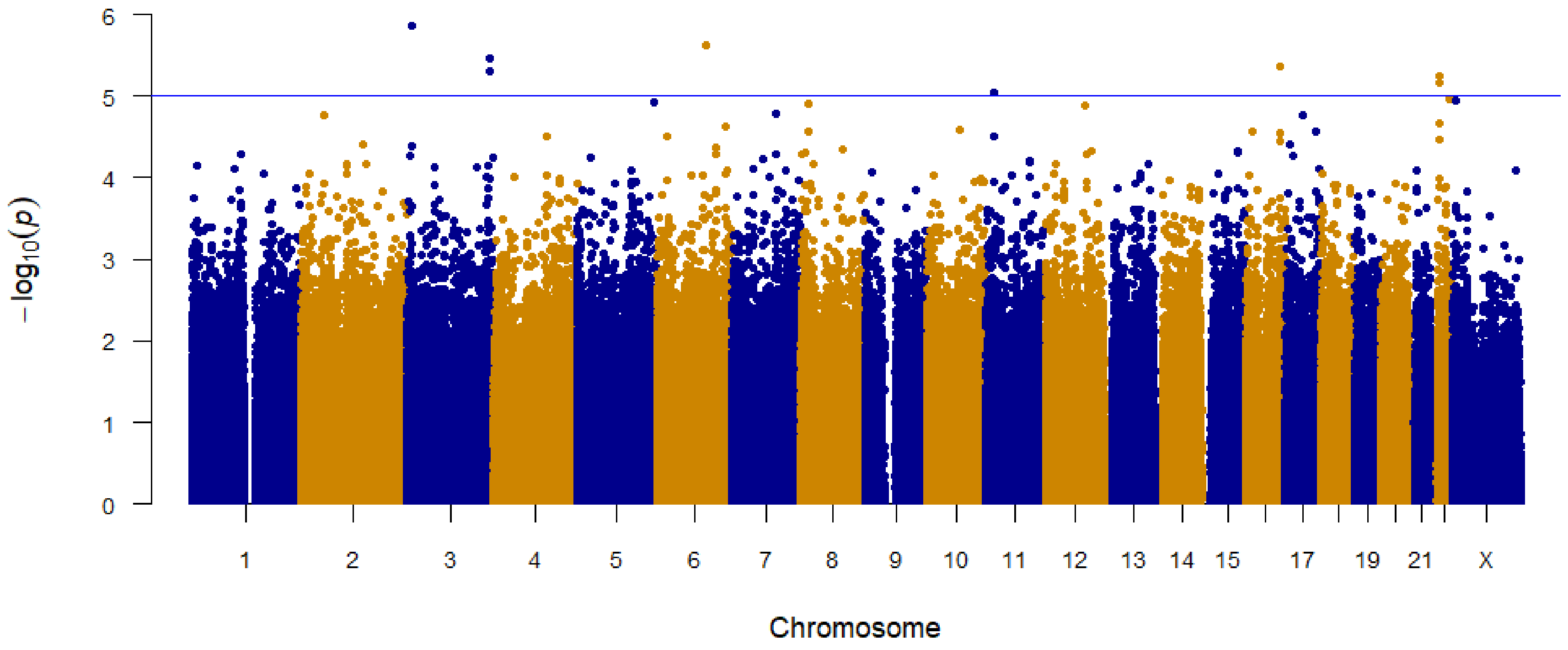

AC_{50} Low

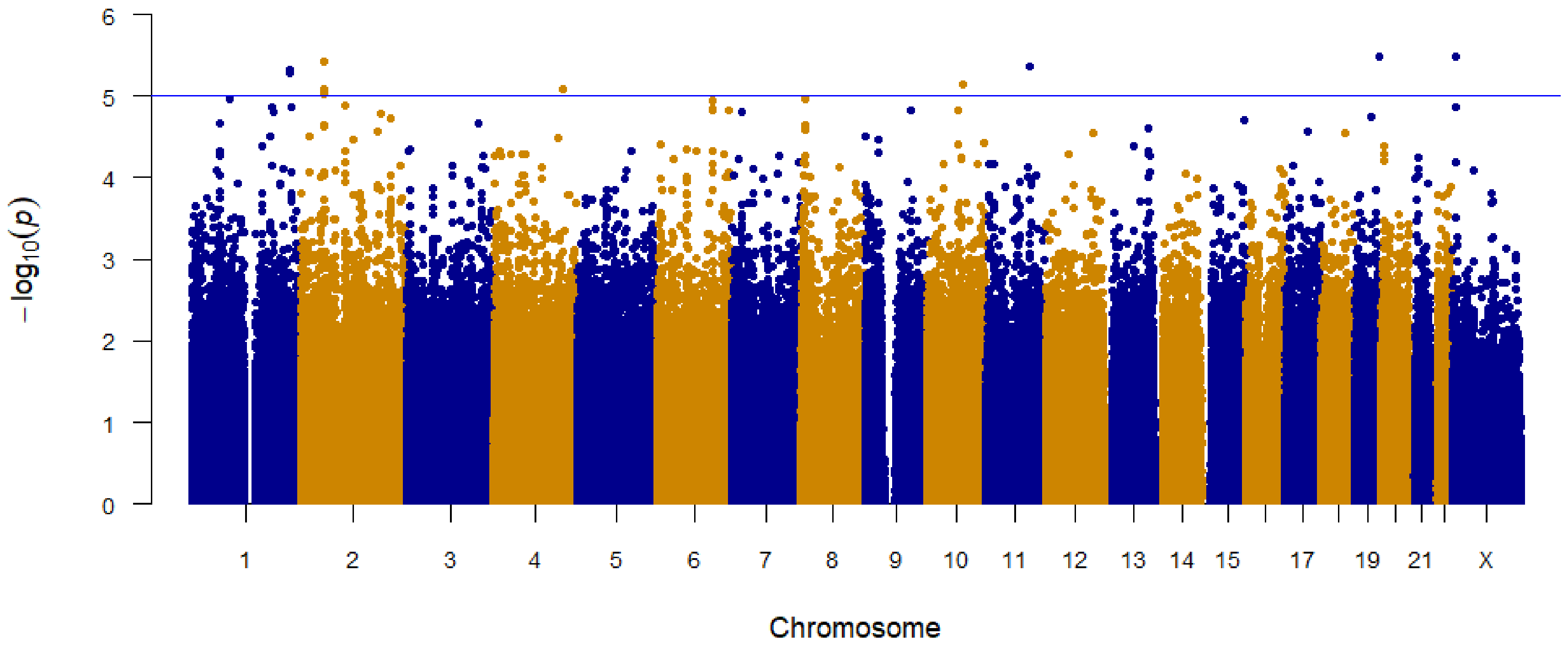
6
5
4
3
2
1
0
$-\log_{10}(p)$
1
2
3
4
5
6
7
8
9
10
11
12
13
14
15
17
19
21
X
Chromosome

AC_{50} High

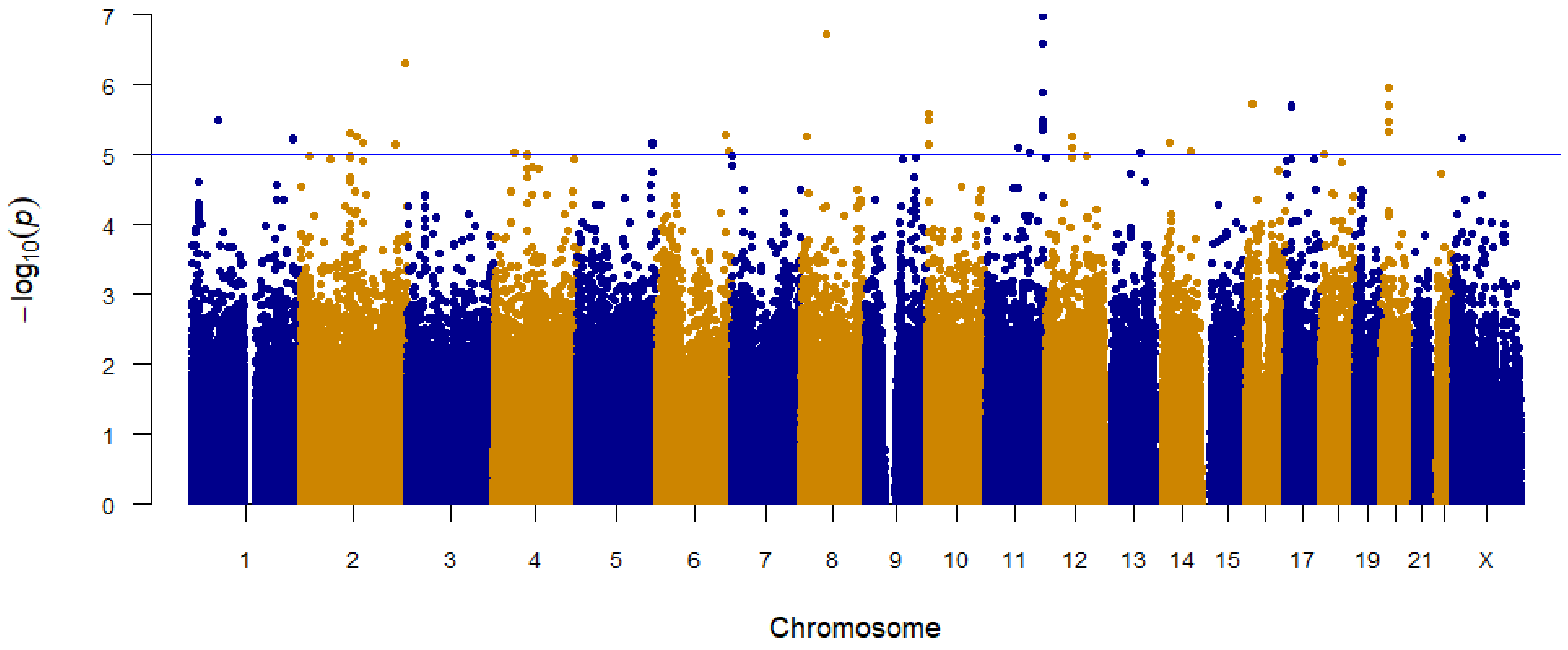

7
6
5
4
3
2
1
0
$-\log_{10}(p)$
1
2
3
4
5
6
7
8
9
10
11
12
13
14
15
17
19
21
X
Chromosome

POD Low

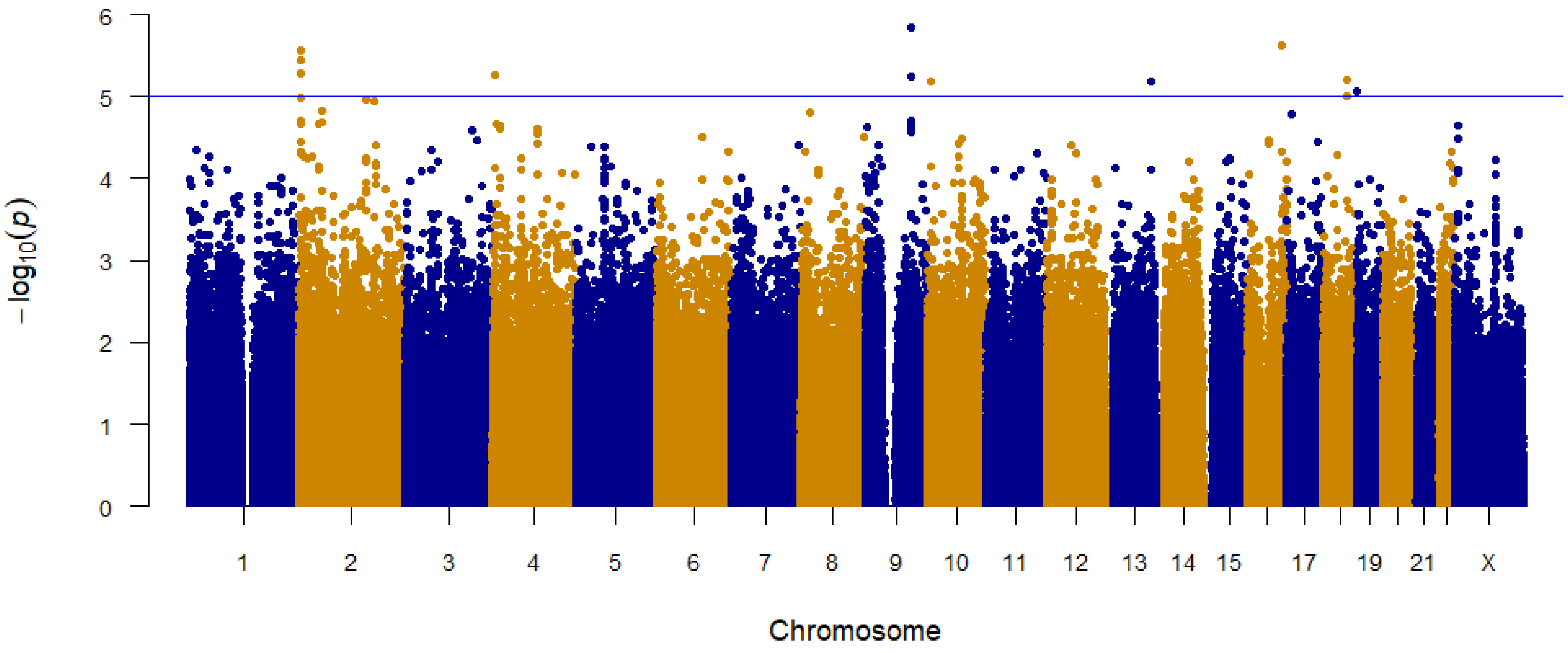

6
5
4
3
2
1
0
−log10(p)
1
2
3
4
5
6
7
8
9
10
11
12
13
14
15
17
19
21
X
Chromosome

POD High

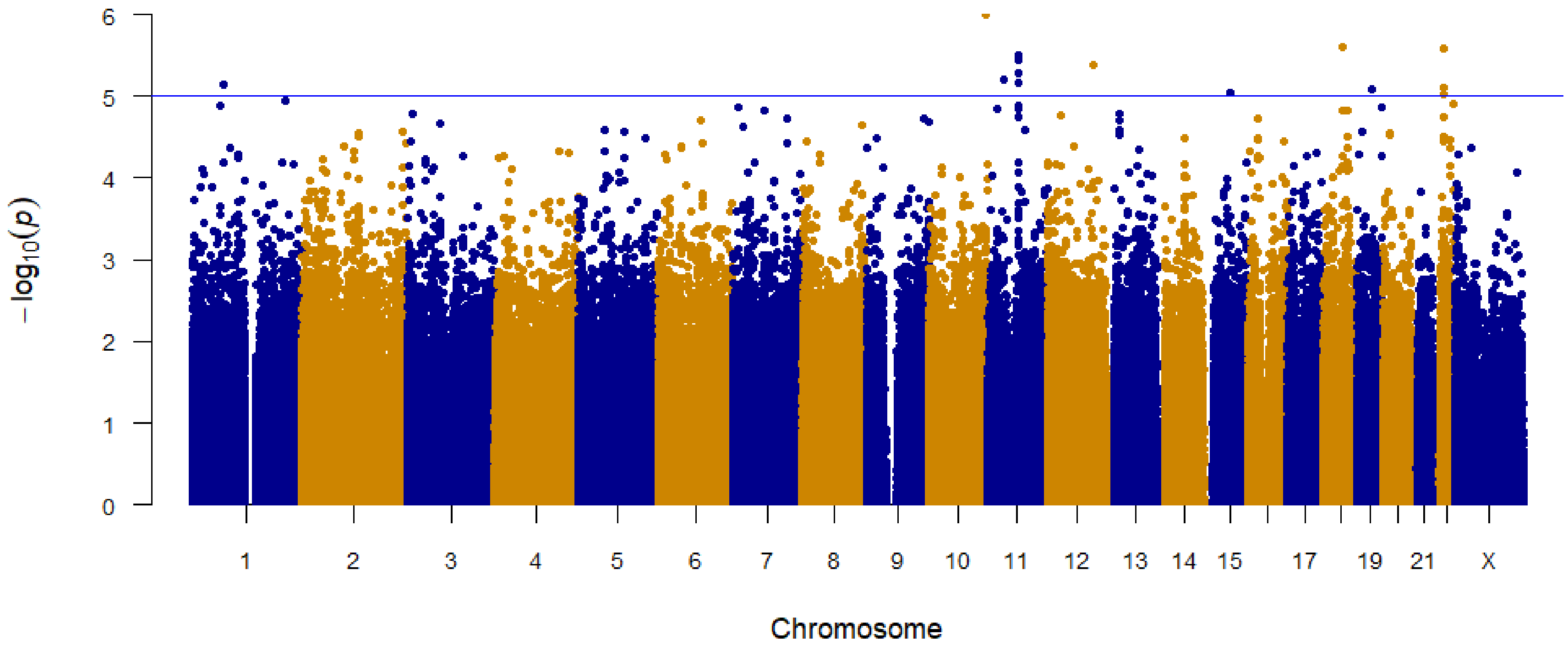

6
5
4
3
2
1
0
−log10(p)
1
2
3
4
5
6
7
8
9
10
11
12
13
14
15
17
19
21
X
Chromosome

Expo Low

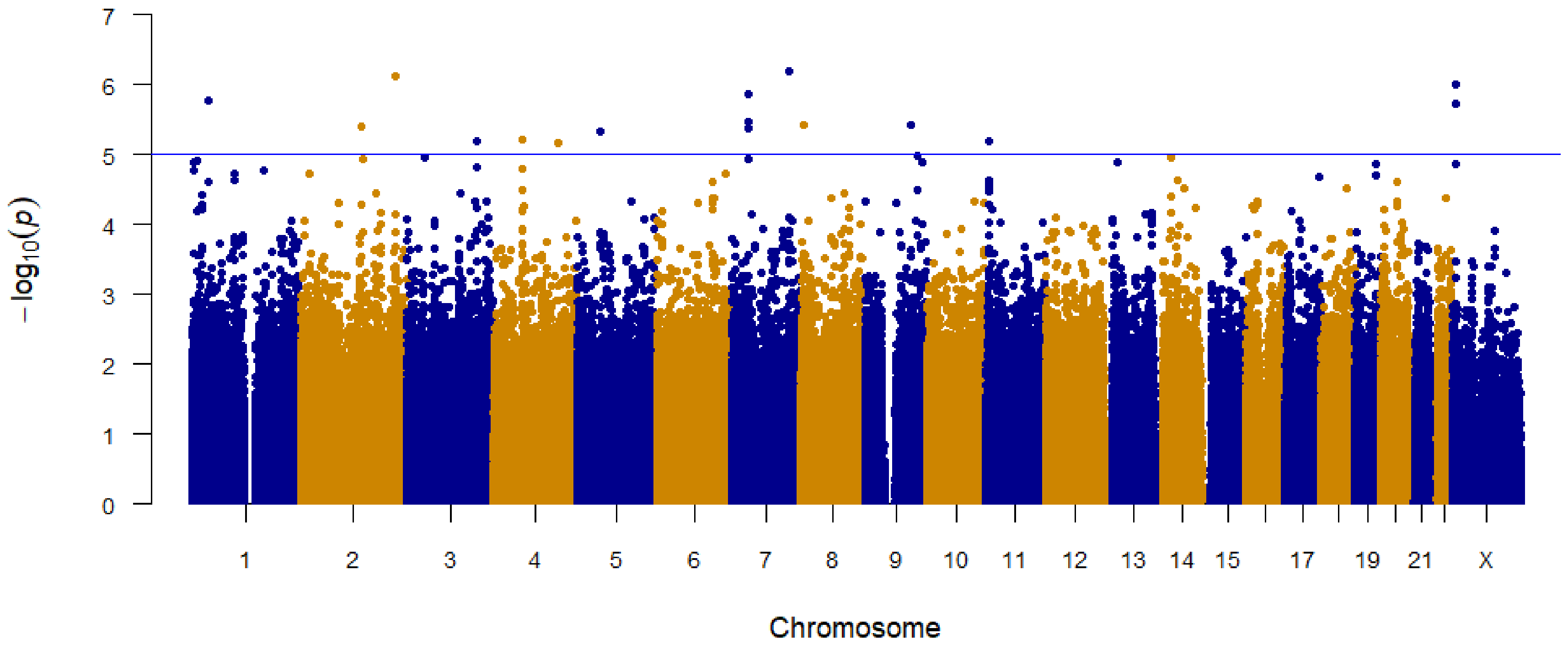

7
6
5
4
3
2
1
0
−log10(p)
1
2
3
4
5
6
7
8
9
10
11
12
13
14
15
17
19
21
X
Chromosome

Expo High

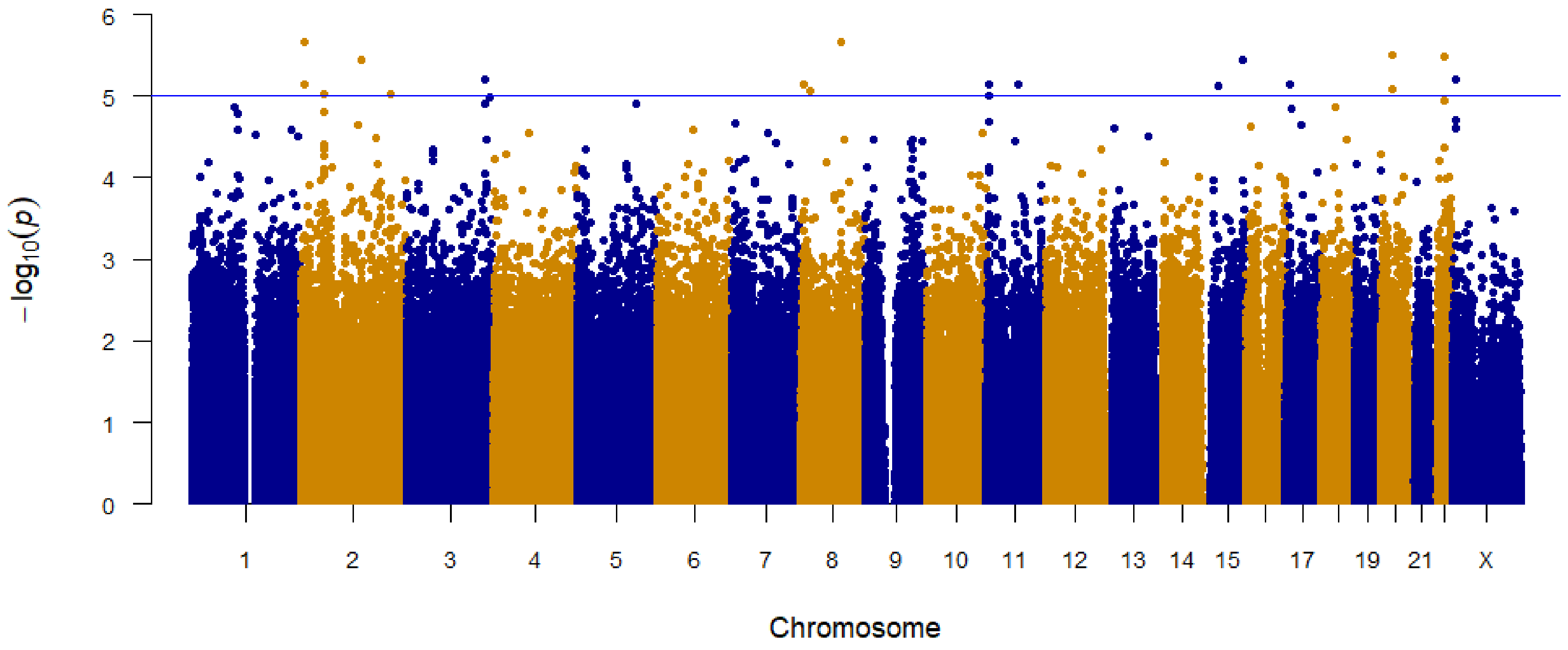

6
5
4
3
2
1
0
−log10(p)
1
2
3
4
5
6
7
8
9
10
11
12
13
14
15
17
19
21
X
Chromosome

RfD Low

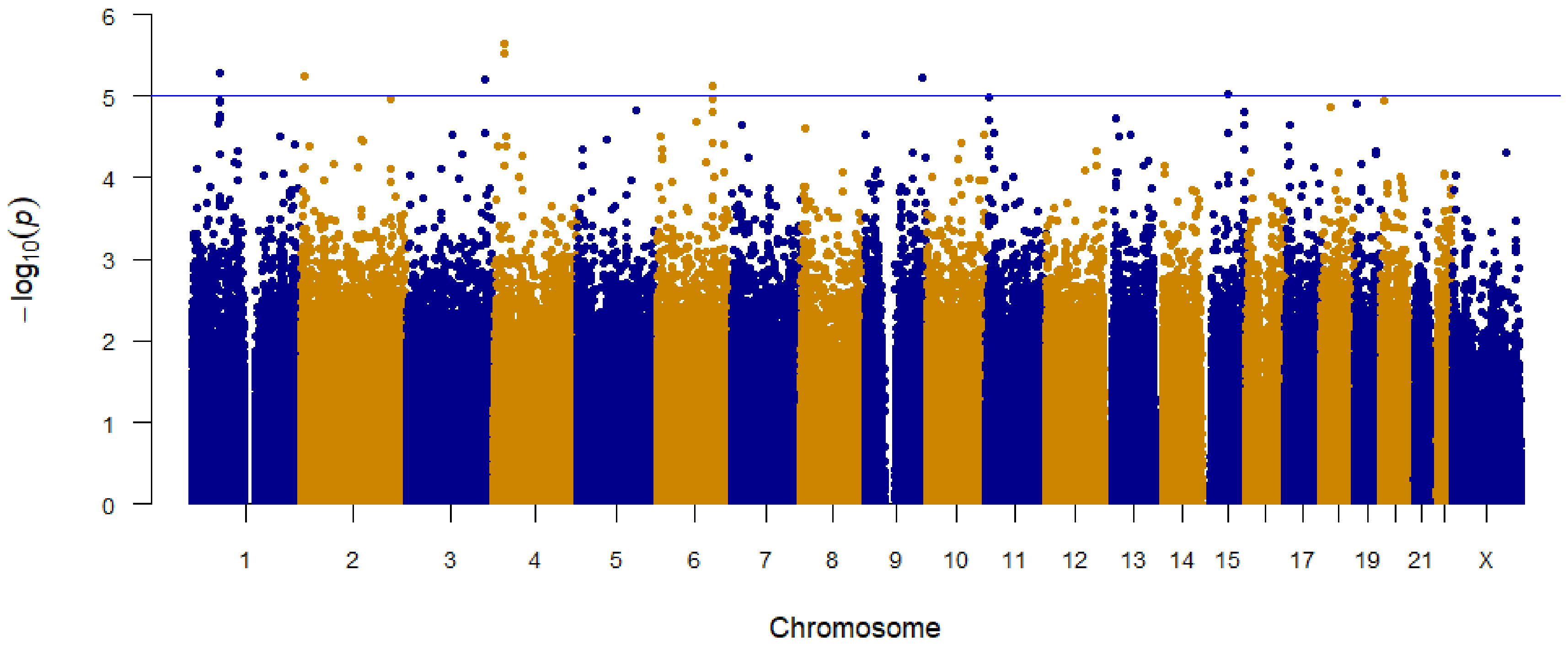
6
5
4
3
2
1
0
−log10(p)
1
2
3
4
5
6
7
8
9
10
11
12
13
14
15
17
19
21
X
Chromosome

RfD High

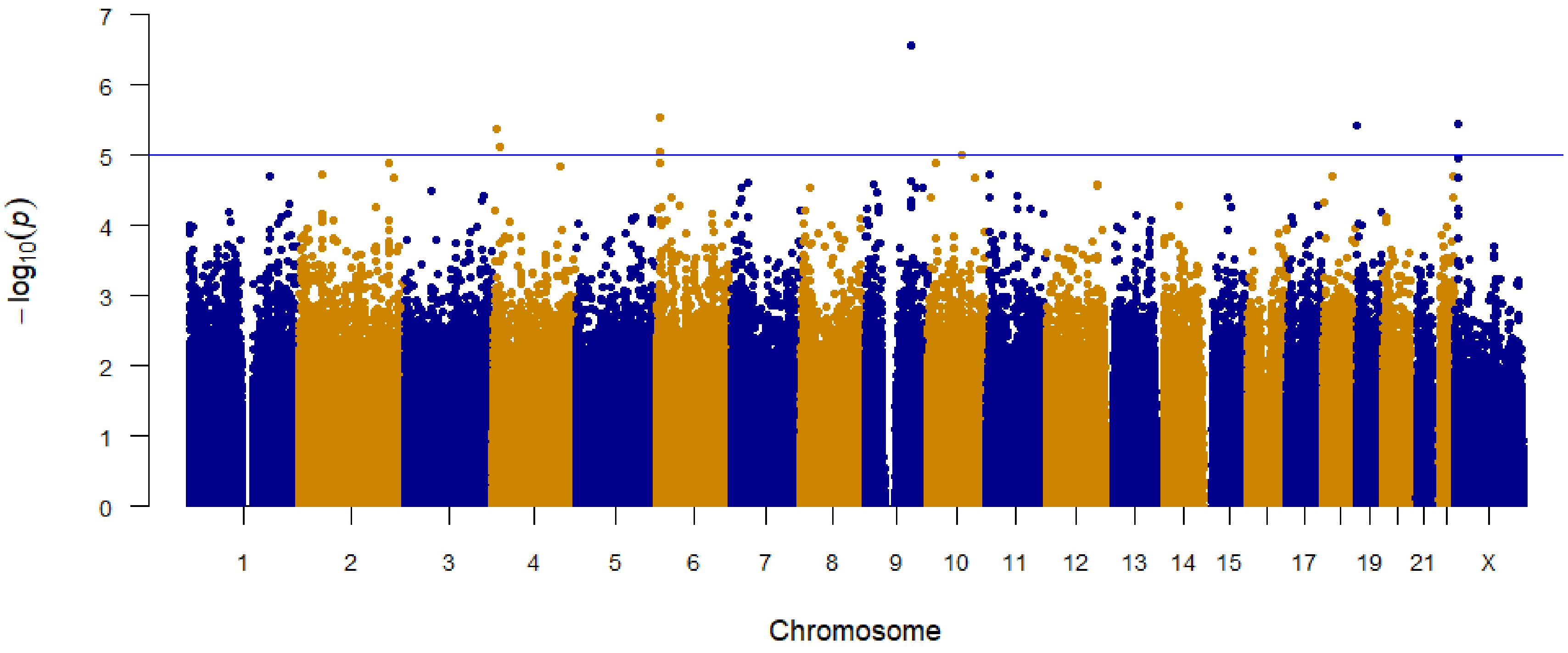

−log10(p)
0
1
2
3
4
5
6
7
1
2
3
4
5
6
7
8
9
10
11
12
13
14
15
17
19
21
X
Chromosome
