# Supplementary material for: A Population-Based Human In Vitro Approach to Quantify Inter-Individual Variability in Responses to Chemical Mixtures
Source: Toxics. 2022 Aug 1;10(8):441. doi: 10.3390/toxics10080441 (PMC9413237; doi:10.3390/toxics10080441)
Supplement: Supplementary file 1 [file toxics-10-00441-s001.zip › Supplemental Figure Legends .pdf]

Figure S1. Plate design layout. Plate design includes all chemicals and mixtures in concentration response as well as positive and negative controls and intraplate replicates (mercuric chloride and 2,4,5-trichlorophenol).

Figure S2. QQplots. Chemical and mixture-specific QQplots reported for both before and after PC correction.

Figure S3. Manhattan plots with mixed model analyses. LocusZoom output visualizations for chemicals (with cytotoxic effects) and all mixtures, showing the chromosomal locations of the SNPs with the corresponding –log10(p). Horizontal line at 10^{-5} represents the corrected genome-wide significance threshold for all SNPs and phenotypes. Chemical and mixture-specific lambda values reported.

Figure S4. Cell line-specific concentration response curves. Concentration response curves for individual cell lines across for chemicals and defined mixtures.

Figure S5. Chemical and mixture-specific Manhattan plot visualizations. LocusZoom output visualizations for chemicals (with cytotoxic effects) and all mixtures, showing the chromosomal locations of the SNPs with the corresponding –log10(p). Horizontal line at 10^{-5} represents the corrected genome-wide significance threshold for all SNPs and phenotypes.

Figure S6. Cytotoxicity comparisons across various *in vitro* models. Distribution of EC_{10} values for chemicals (A) and mixtures (B) across various *in vitro* models. Box-and-whisker plots show the interquartile range (boxes) and 10th to 90th percentile (whiskers) of the chemical and mixture distribution of median EC_{10} values.
