# Supplementary material for: A Population-Based Human In Vitro Approach to Quantify Inter-Individual Variability in Responses to Chemical Mixtures
Source: Toxics. 2022 Aug 1;10(8):441. doi: 10.3390/toxics10080441 (PMC9413237; doi:10.3390/toxics10080441)
Supplement: Supplementary file 1 [file toxics-10-00441-s001.zip › Supplemental Table Legend.pdf]

Supplemental Tables:

Table S1. Chemical identification information. Chemical characteristics including: name, CAS-RN, molecular weight, chemical formula, vendor, catalog number, and chemical class.

Table S2. Individual lymphoblast cell line identification. Individual cell line information including: screening batch number, cell line ID, sex, geographical background for subpopulation identification.

Table S3. Defined mixture information. Individual chemical information (name, CAS-RN, chemical class, and molecular weight) and the concentrations of individual constituents in each of the 8 defined mixtures.

Table S4. Pearson correlation coefficients for intraplate replicates across all cell lines. Pearson correlation coefficient values (and r-squared values) and corresponding p-values for mercuric chloride and 2,4,5-trichlorophenol across all individual lymphoblast cell lines and by batch.

Table S5. Pearson correlation coefficients for intraplate replicates across all cell lines. Interplate replicate Pearson correlation coefficient (and r-squared) and p-values for all individual cell lines.

Table S6: SNPID and corresponding p-values. SNPIDs from the GWAS plots for chemicals (with cytotoxic effect) and mixtures that had a p-value above the suggestive significance threshold of $p<10^{-5}$.

Table S7. Chemical and mixture raw and normalized cytotoxicity data across all cell lines. Raw and normalized cytotoxicity data for 146 individual cell lines, including the concentration-response and intra- and inter-plate replicate data for all 42 chemicals and 8 defined mixtures.

Table S8. Median EC_{10} values for individual chemicals and mixtures. EC_{10} values for all individual cell lines for individual chemicals (with effect) (sheet 1) and defined mixtures (sheet 2).

Table S9. Chemical and mixtures toxicodynamic variability factor summary data. $TDVF_{05}$ summary statistics including: median, 25th, 75th, 5th, 95th percentile, minimum and maximum values for all chemicals (with cytotoxic effects) (sheet 1) and defined mixtures (sheet 2).

Table S10. Genome-wide association study candidate genes and corresponding p-values. Candidate gene identification including gene abbreviation, gene name, function, chromosomal location, number of gene hits across chemicals and mixtures and identification of those particular and identification of chemicals/mixtures.

Table S11: Genome-wide association study SNPs with beta coefficients. SNP IDs with the corresponding beta coefficients for all chemicals (with cytotoxic effects) and mixtures.
