# Supplementary figures and images for: A Population-Based Human In Vitro Approach to Quantify Inter-Individual Variability in Responses to Chemical Mixtures
Source: Toxics. 2022 Aug 1;10(8):441. doi: 10.3390/toxics10080441 (PMC9413237; doi:10.3390/toxics10080441)

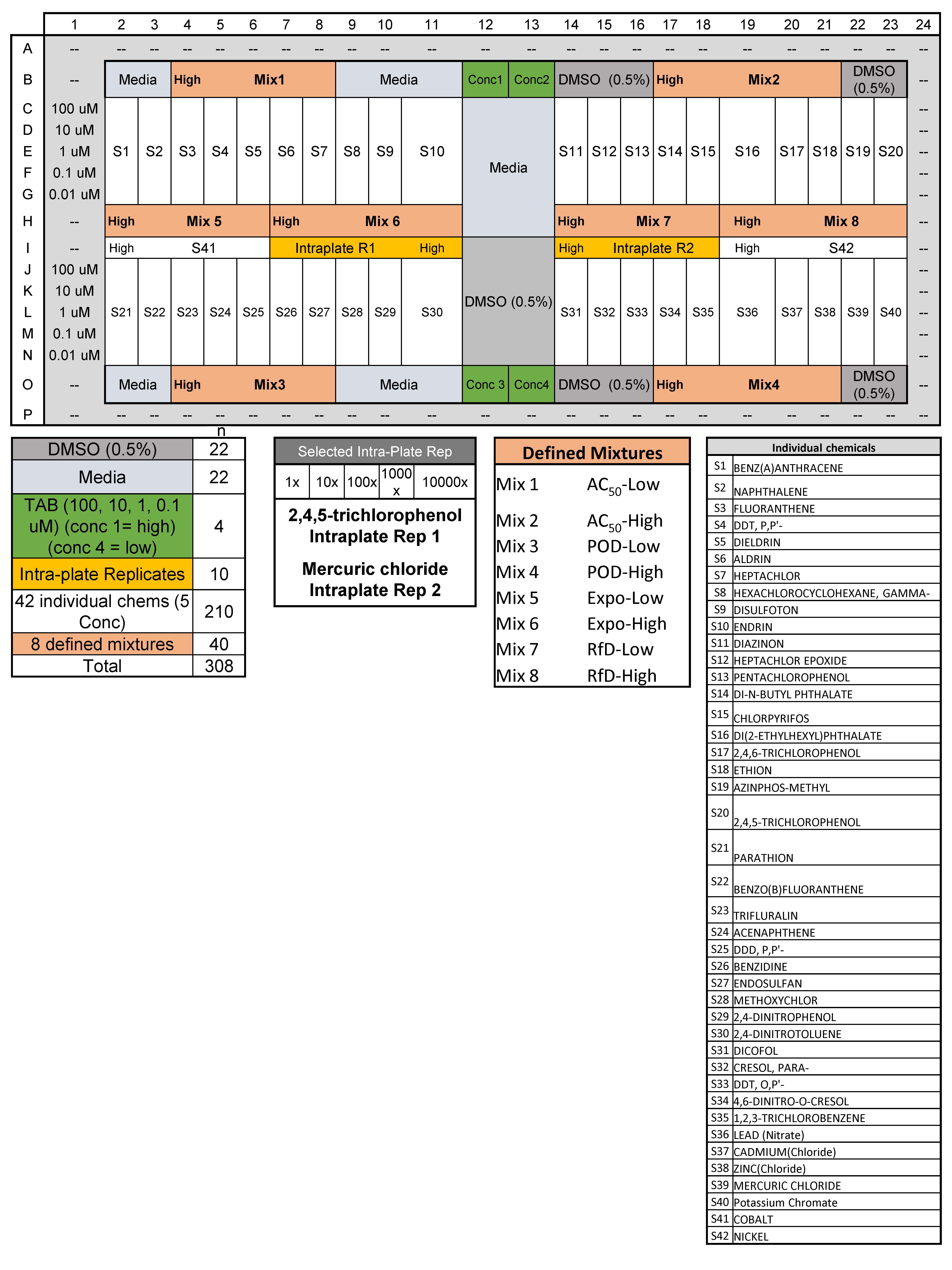

Supplement: Supplementary file 1 [file toxics-10-00441-s001.zip › Figure S1 Plate design layout PNG.png]

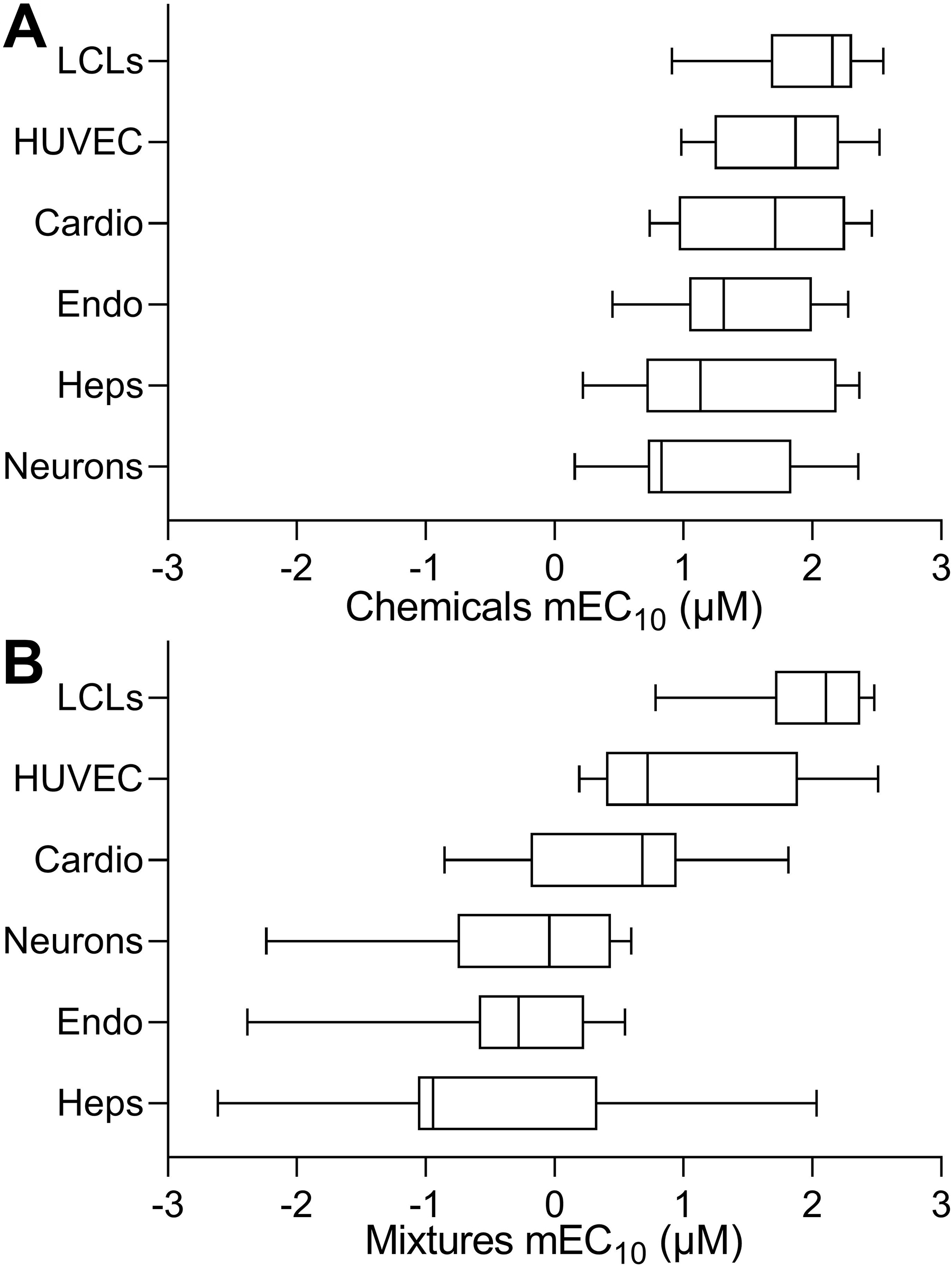

Supplement: Supplementary file 1 [file toxics-10-00441-s001.zip › Figure S6 Cytotoxicity comparisons across various in vitro models PNG.png]
